# Synthesis of Benzo[*c*]cinnolinium Salts from 2-Azobiaryls by Copper(II) or Electrochemical Oxidation

**DOI:** 10.1021/acs.orglett.4c00213

**Published:** 2024-02-15

**Authors:** Huan-Chang Hsiao, Meng-Che Li, Guganchandar Vedarethinam, Pei-Lin Chen, Shih-Ching Chuang

**Affiliations:** Department of Applied Chemistry, National Yang Ming Chiao Tung University, Hsinchu 30010, Taiwan

## Abstract

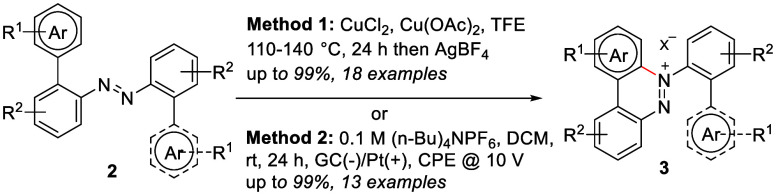

Synthesis of benzo[*c*]cinnolinium salts by copper(II)-promoted or electrochemical oxidation of 2-azobiaryls is described. A variety of diversely functionalized benzo[*c*]cinnolinium salts were easily constructed by this strategy with excellent functional group tolerance and high efficiency. An interesting fluorescence centered at 571 nm is revealed by a benzo[*c*]cinnolinium salt with electron push–pull substitutions. The mechanism is proposed to go through single-electron transfer driven by oxidant and intramolecular cyclization via nucleophilic addition, followed by an anion exchange.

The benzo[*c*]cinnoline is a privileged molecule and often appears in the framework of functional compounds, such as a ligand to construct complexes,^1^ biosensors,^2^ antiandrogenic activity,^3^ anticancer agents,^4^ organic field-effect transistors,^5^ and as a group of fluorophores for biological studies.^6^ In addition, benzo[*c*]cinnoline has a considerable influence on the photophysical properties. For example, compounds with the structure of benzo[*c*]cinnoline have the property of broad absorption bands and exhibit large Stoke shifts.^6^ Furthermore, the quantum yield of the triplet formation of benzo[*c*]cinnoline is about unity by measuring the heat energy coming from the excited state of benzo[*c*]cinnoline together with the fluorescence quantum yield measurement, and a significant short triplet lifetime occurs from the nonradiative relaxation process from the T1 state to the ground state.^7^ Consequently, the synthesis of benzo[*c*]cinnolines and benzo[*c*]cinnolinium salts has been extensively reported due to the aforementioned diverse functionalities. The synthesis of benzo[*c*]cinnolines were reported through an electrochemical reduction from nitroaryls,^8^ transition-metal catalysis from aryl halides and aryl hydrazide or nitrobenzene,^9^ and chemical oxidation/reduction of 2,2′-diamine^10^ or 2,2′-dinitro biphenyls.^11^ However, the method for the preparation of benzo[*c*]cinnolinium salts is rather limited: the preparation of benzo[*c*]cinnolinium salts could start from triphenyldiazenium with moderate yield through dehydrogenative coupling (Scheme 1a),^12^ a multistep synthesis from arylamines and arylboronic acids followed by *N*-alkylation as a key step (Scheme 1b)^6^ or cyclization of azobenzenes with aryl iodonium salts catalyzed by iridium (Scheme 1c).^13^ Therefore, we were inspired to report the efficient synthesis of benzo[*c*]cinnolinium salts by copper(II)-promoted or electrochemical oxidation that could facilitate the intramolecular cyclization of 2-azobiaryls (Scheme 1d).

**Scheme 1:**
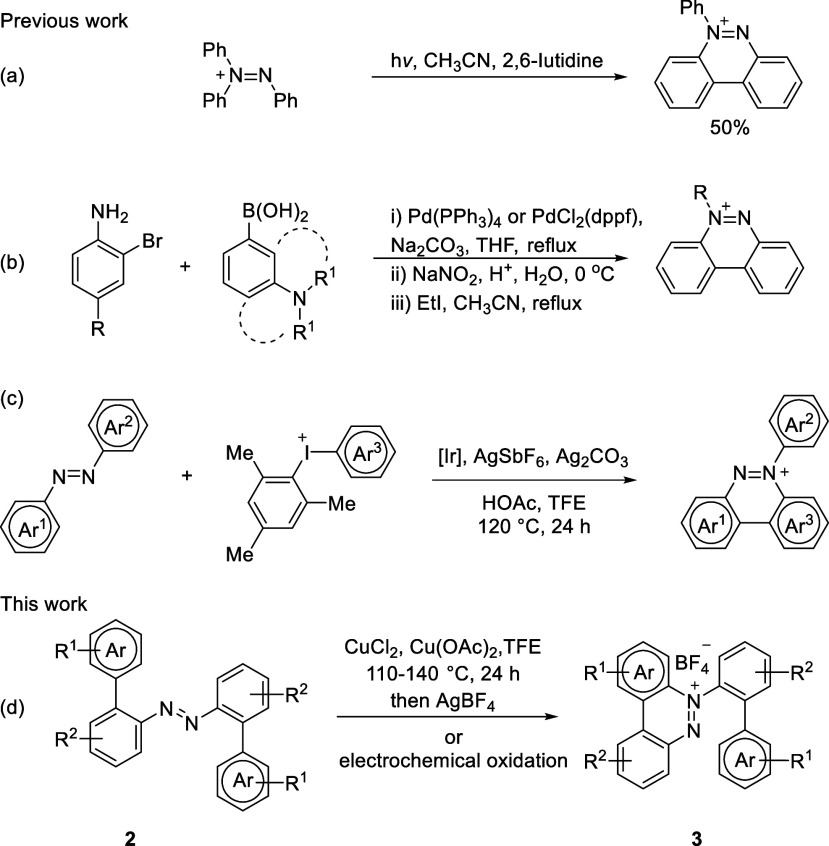
Syntheses of Benzo[*c*]cinnolinium Salts

We started our investigation using 2-azobiaryl **2a** as the model substrate to optimize reaction conditions for intramolecular cyclization, and the observed results are summarized in Table 1. After considerable experimentation, the desired benzo[*c*]cinnolinium salts **3a** could be isolated in 55% yield with Pd(OAc)_2_ (10 mol %) as the catalyst, Cu(OAc)_2_ (3.0 equiv) and CuCl_2_ (3.0 equiv) as the oxidants in TFE (1.5 mL) at 110 °C for 24 h followed by using AgBF_4_ (1.5 equiv) for anion exchange (entry 1).^14^ To confirm individual reagent dependence, reactions were performed without Pd(OAc)_2_ or Cu(OAc)_2_ or CuCl_2_, respectively (entries 2–4). Surprisingly, the yield of benzo[*c*]cinnolinium salts **3a** was significantly increased without Pd(OAc)_2_ (99%, entry 2). In the test of the oxidizing agent, the reaction yield tended to drop significantly when CuCl_2_ or Cu(OAc)_2_ was not used (entries 3 and 4). The use of co-oxidants such as ceric ammonium nitrate, selenium dioxide, or iodobenzene diacetate could improve the yield of product **3a** (entries 6–8). However, reactions using hexafluoroisopropanol as a solvent had slightly lower efficiency (entry 9). Next, we found that adding AgBF_4_ as a coadditive with Cu(OAc)_2_ simultaneously to reaction did not directly exchange the anion of the product into tetrafluoroborate. Therefore, we speculated that adding AgBF_4_ after the reaction is a necessary step (entry 10). In the screening of oxidants, we observed that both CuCl_2_ and Cu(OAc)_2_ are indispensable reagents (entries 11–13). Furthermore, we found that CuCl_2_ is the key reagent to promote the reaction since less products were isolated under the conditions with less amounts of CuCl_2_ or Cu(OAc)_2_ (entries 14–17). An arguably possible concept by just using 3 equiv of CAN, SeO_2_, or PhI(OAc)_2_ gave poor yields (entries 18–20), indicating further oxidation and stabilization with suitable counteranion would be necessary.

**Table 1:**
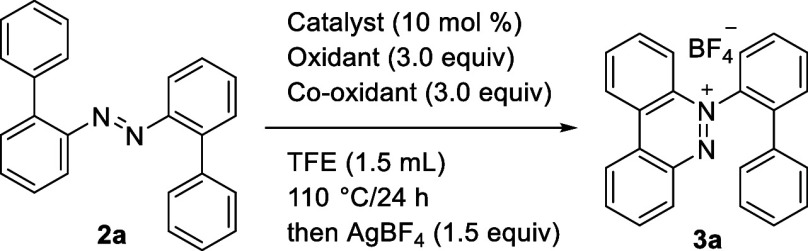
Optimization Studies for the Intramolecular Cyclization of 2-Azobiaryls^a^

entry	catalyst (10 mol %)	oxidant (equiv)	co-oxidant (3.0 equiv)	yield (%)^b^
1	Pd(OAc)_2_	CuCl_2_ (3.0)	Cu(OAc)_2_	55
2	–	CuCl_2_ (3.0)	Cu(OAc)_2_	99
3	Pd(OAc)_2_	–	Cu(OAc)_2_	trace
4	Pd(OAc)_2_	CuCl_2_ (3.0)	–	40
5	–	AgBF_4_ (1.5)	Cu(OAc)_2_	27
6	–	CuCl_2_ (3.0)	CAN	88
7	–	CuCl_2_ (3.0)	SeO_2_	85
8	–	CuCl_2_ (3.0)	PhI(OAc)_2_	99
9^c^	–	CuCl_2_ (3.0)	Cu(OAc)_2_	95
10	–	CuCl_2_ (3.0)	Cu(OAc)_2_/AgBF_4_ (3.0/1.5)	99
11	–	CuCl_2_ (3.0)	–	15
12	–	–	Cu(OAc)_2_	N.D.
13	–	–	AgBF_4_	N.D.
14	–	CuCl_2_ (0.5)	Cu(OAc)_2_	30^d^
15	–	CuCl_2_ (1.0)	Cu(OAc)_2_	66^d^
16	–	CuCl_2_ (2.0)	Cu(OAc)_2_	72^d^
17	–	CuCl_2_ (2.0)	Cu(OAc)_2_^e^	45
18	–	–	CAN	32
19	–	–	SeO_2_	2
20	–	–	PhI(OAc)_2_	39

a Reaction conditions: 2-azobiaryl **2a** (0.1 mmol), TFE (1.5 mL), catalyst, oxidant, co-oxidant and additive at 110 °C for 24 h under aerobic conditions.

b Yields were determined by ^1^H NMR spectroscopic method using 1,3,5-trimethoxybenzene as an internal standard unless otherwise noted.

c Hexafluoroisopropanol (1.5 mL) was used as solvent.

d Yields were obtained after purification and anion exchange with AgBF_4_.

e 1.0 equiv of Cu(OAc)_2_.

With the optimal reaction conditions in hand, we explored the scope and the generality of this reaction, employing a wide range of substrates (Scheme 2). 2-Azobiaryls bearing a methyl group at the C4′, C3′, or C2′ position were tolerated. The corresponding products (**3b** and **3c**) were afforded in 91 and 99% yields. However, the reaction of the C2′-methyl substituted substrate giving **3d** became sluggish, where the yield was decreased to 33% containing two diastereomers. It is presumed that the factor of steric hindrance leads to the decrease of the yield and the isomers are formed due to the inability to rotate freely about C1–C1′. Substrates **2** with a methoxy group at C4′ and C3′ afforded the corresponding products (**3e** and **3f**) in 69–71% yields due to poor solubility, resulting in some starting materials recovered. Notably, introducing a fluorine group at the C4′ or C3′ position of the phenyl ring largely retarded the reaction, leading to **3g** and **3h** in 32% and 27% yields, respectively. The reaction was found to be sensitive to the electronic nature of the substituents in the internal ring. The reaction of substrate bearing a methyl group at the internal ring of C4-position gave the desired **3i** in 70% yield, which was supported by X-ray crystallographic analysis (Supporting Information). However, the result of substitution with a fluorine group at the C4-position was 49% (**3j**). Furthermore, reactions with poor yields were obtained when the C4 is substituted with methyl and C4′ is substituted with methyl or fluoro group, giving 44% and 30% of **3k** and **3l**, respectively. Based on this phenomenon, we speculated that the tendency for the formation of the desired radical intermediate via the oxidation step would be reduced by functional group substitution at the internal ring. Moreover, the external aryl ring replaced by a thienyl group furnished the expected products **3m** in 35% yield, as established by single crystal X-ray diffraction analysis. In addition, reactions employing α- or β-naphthalene at the external ring and their corresponding products **3n** and **3o** were obtained in 20% and 22% yield, likely due to steric hindrance. Notably, asymmetric 2-azobiaryl with methyl and fluoro groups at two C4′ results in 69% yield of **3p**, asserting that oxidation took place at the electron-rich ring. However, a diazo **2q** equipped with a *p*-CF_3_ moiety did not yield the corresponding salt **3q** by using chemical or electrochemical oxidation.

**Scheme 2:**
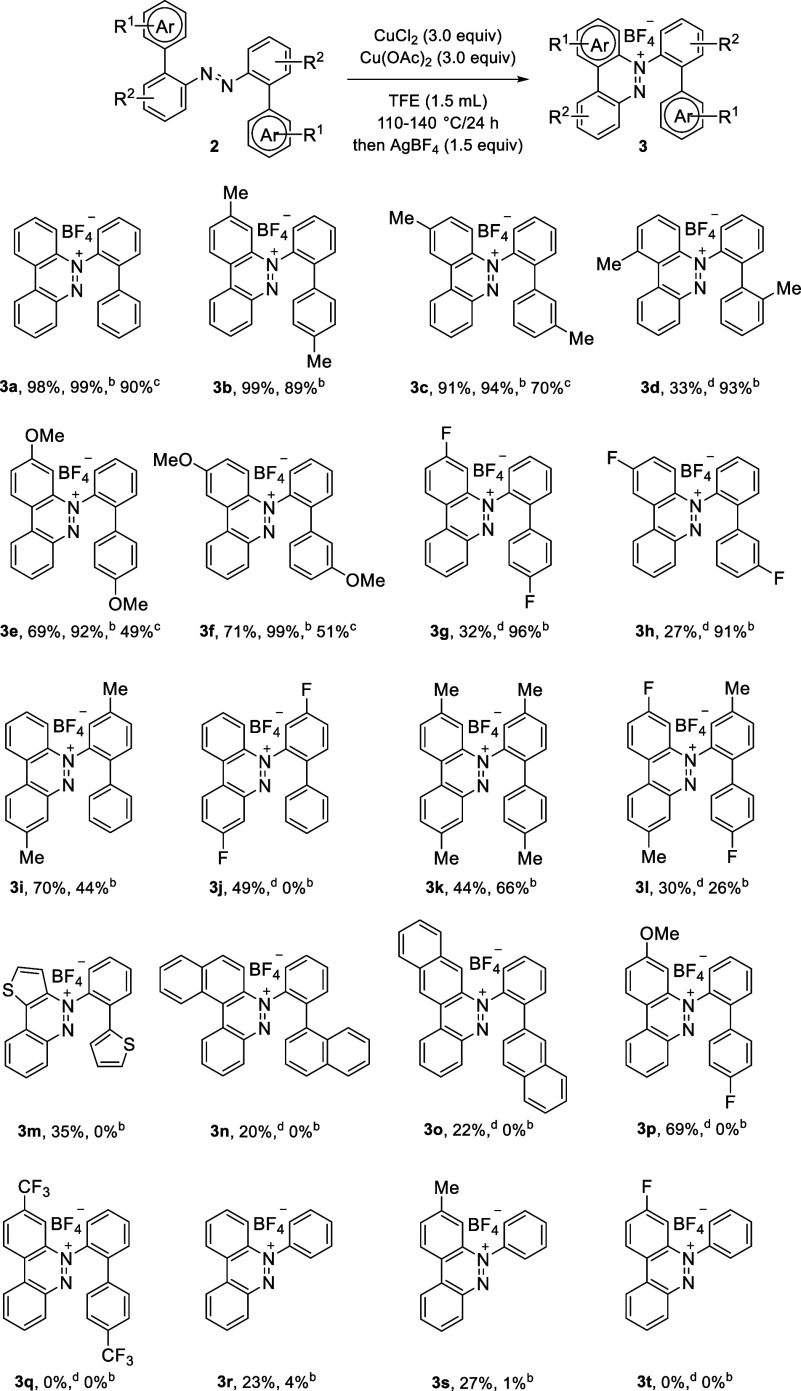
Substrate Scope of Benzo[*c*]cinnolinium Salts **3** Reactions were carried out with substrate **2** (0.10 mmol), CuCl_2_ (3.0 equiv), Cu(OAc)_2_ (3.0 equiv) in 1.5 mL TFE at 110 °C for 24 h under aerobic conditions, then anion exchange with AgBF_4_ was performed, unless otherwise noted. Method A: Substrate **2** (0.025 mmol), *n*Bu_4_NPF_6_ (0.3 mmol), DCM (3 mL), undivided cell with Pt electrode (1 × 2 cm^2^) and glassy carbon electrode, with constant voltage of 10.0 V at room temperature for 24 h. Method B: Substrate **2** (0.025 mmol), KPF_6_ (2.0 equiv), 3.0 mL THF, undivided cell with Pt electrodes (1 × 2 cm^2^) and carbon electrodes, constant voltage of 10.0 V, room temperature, 24 h. Yields were determined by ^1^H NMR spectroscopy using 1,3,5-trimethoxybenzene as an internal standard. 140 °C.

To further extend the strategy of product preparation, we studied the synthesis of benzo[*c*]cinnolinium salts **3** by electrochemical oxidation at room temperature. With the optimized reaction conditions in hand (Table S1, method A), the substrates with an external phenyl ring substituted with electron-donating groups, such as methyl (**2b**–**2d**) and methoxy (**2e**–**2f**), successfully participated in electrolysis, giving the corresponding products (**3b**–**3f**) in good yields. Reactions with the substitution of fluorine were also tolerated (**2g**–**2h**). However, in the case of methyl group substitution at internal ring C4, the yield would be significantly reduced to 44% (**3i**). Surprisingly, a fluoro substitution at C4 did not yield any product formation of **3j**. A notable comparison of the reactivity toward electrochemical oxidation lies in the cases with a methyl or fluoro group substituted at C4′, which gave 66% and 26% yields of **3k** and **3l**, respectively. This outspoke that the reaction was disfavored with an electron-withdrawing substitution on the external ring. Furthermore, the reactions of 2-azo biaryls with 2-thienyl, 1-naphthyl, and 2-naphthyl did not give any products upon electrochemical oxidation, nor did the asymmetric 2-azobiaryl **2p**. An eminently poor reactivity on **2q** was observed, with negative results by chemical and electrochemical oxidation, due to its very low-lying HOMO energy level. Interestingly, the diazo substrates **2r**–**2t** with one aryl and one biaryl exhibit relatively poor reactivity, reflected from their low-lying HOMOs (see Figure S6a). This presents a limited applicability for preparing such salts by using electrochemical methods. Owing to that the desired products and *n*-Bu_4_NPF_6_ could not be well-separated, we carried out the electrochemical oxidation with KPF_6_ (Method B) as electrolytes for further purification. Further yield-checking of selected examples with method B had also been completed, and these results showed moderate to good yields (**3a**, **3c**, **3e**, and **3f**). The poor reactivity of **2d**, **2g**, **2h**, and **2j** toward chemical oxidation is consistent with their low-lying HOMO (Figure S6a), and that electrochemical oxidation could provide sufficient energy to overcome the first single-electron oxidation step. With a *meta*-fluoro substitution, however, substrate **2j** may exhibit less nucleophilicity toward cyclization, leading it to be not only difficult to oxidize but also difficult to cyclize. Furthermore, reactions of **2m**–**2p** show distinct differences on the formation of **3m**–**3p** between chemical and electrochemical oxidations. In addition to the smaller HOMO/LUMO gaps of **2m**–**2p** between 3.33 and 3.36 eV, the LUMO energy levels of **3m**–**3p** (Figure S6b), bearing thienyl, naphthyl moiety, and electron push–pull pair, respectively, are relatively higher among the studied products, indicating their possible further reductions at the cathode in the undivided cell—this is reflected from the recovery of these substrates with an appreciable amount after the reactions. The poor yields for **3n**–**3o** also possibly resulted from the steric hindrance for biaryl formation.

The substituted benzo[*c*]cinnolinium salts **3**, featuring methyl, methoxy, fluoro, thienyl, 2-naphthyl substitutions and unsymmetrical azobenzenes, showed promising photophysical properties and exhibited typical absorption bands from 300 to 550 nm (Figures S1, S2). Among them, a strong absorption band at 350–410 nm and a weak absorption band spanning 400 to 550 nm can be identified. Notably, the red shift of the weak absorption band can be observed when the methoxy group substituted at benzo[*c*]cinnolinium salts (**3e** and **3p**). Based on TD-DFT calculations on **3a**–**3t** cations and summarized absorption spectra of these cations, oscillator strengths, relevant electronic transitions and MOs in Figures S7–S26, the transitions that could involve intramolecular charge transfer are highlighted in red color. We conclude that although the absorptions at longer wavelengths are derived from intramolecular charge transfer, their oscillator strengths are very small. However, other shorter wavelength transitions could consist of some extent of intramolecular charge transfer absorptions. Last, only compound **3p** showed fluorescence emission at 571 nm (Figure S3), and other compounds do not show obvious fluorescence. Its computed fluorescent emission is centered at 557 nm with an oscillator strength of 0.0370.

To clarify these reactions in greater detail, we first examined 2-ethylazobenzene **2u** and 2-cyclopropylazobenzene **2v** and found that the desired products were not obtained under standard conditions (Scheme S1a,b). Moreover, we also tested the reaction of reactant **2v** with diphenylacetylene under standard conditions, and no expected product was produced nor did the transition metal-catalyzed intermolecular cyclization product take place (Scheme S1c). The results suggest that the reaction proceeded via oxidation of the external aryl ring rather than transition-metal-catalyzed C–H bond activation. We further confirmed that the reaction mechanism involved the generation of a free radical intermediate through the oxidation step, collaborated by the decreased yield when the free radical inhibitor TEMPO was added (Scheme S1d). Intramolecular cyclization of the parent 2-azobiaryl **2a** with 1.0 mmol in standard conditions and electrochemical oxidation (Method A) were accomplished in 99% and 56% yield, respectively (Scheme S1e,f).

We selected **2a**, **2b**, **2g**, **2q**, and **2s** for cyclic voltammetry study, summarized in Table S3 and Figure S5. The 2-azobiaryl substrates equipped with an electron-donating moiety exhibit higher-lying HOMOs (**2b**) and those with an electron-withdrawing moiety show low-lying HOMOs (**2g** and **2q**). It is important to note that the diazo with a biaryl and an aryl group displays a lower lying HOMO (**2b** versus **2s**), indicating its lower reactivity toward oxidation.

According to the experimental findings and literature reports,^13,15^ a plausible mechanism is proposed in Scheme 3a. Initially, copper(II) directed by the azo group oxidizes the external aromatic ring by single electron transfer (SET) and forms the radical cationic intermediate **I**. Subsequently, an intramolecular cyclization reaction takes place via nucleophilic addition of the azo group, delivering intermediate **II**, followed by second SET through copper(II) acetate to oxidize the C–H bond at the C2′ position and removal of one molecule of acetic acid to afford the intermediate **3-X**. Finally, benzo[*c*]cinnoline salt **3a** was formed by exchanging anions in the presence of silver tetrafluoroborate.

**Scheme 3:**
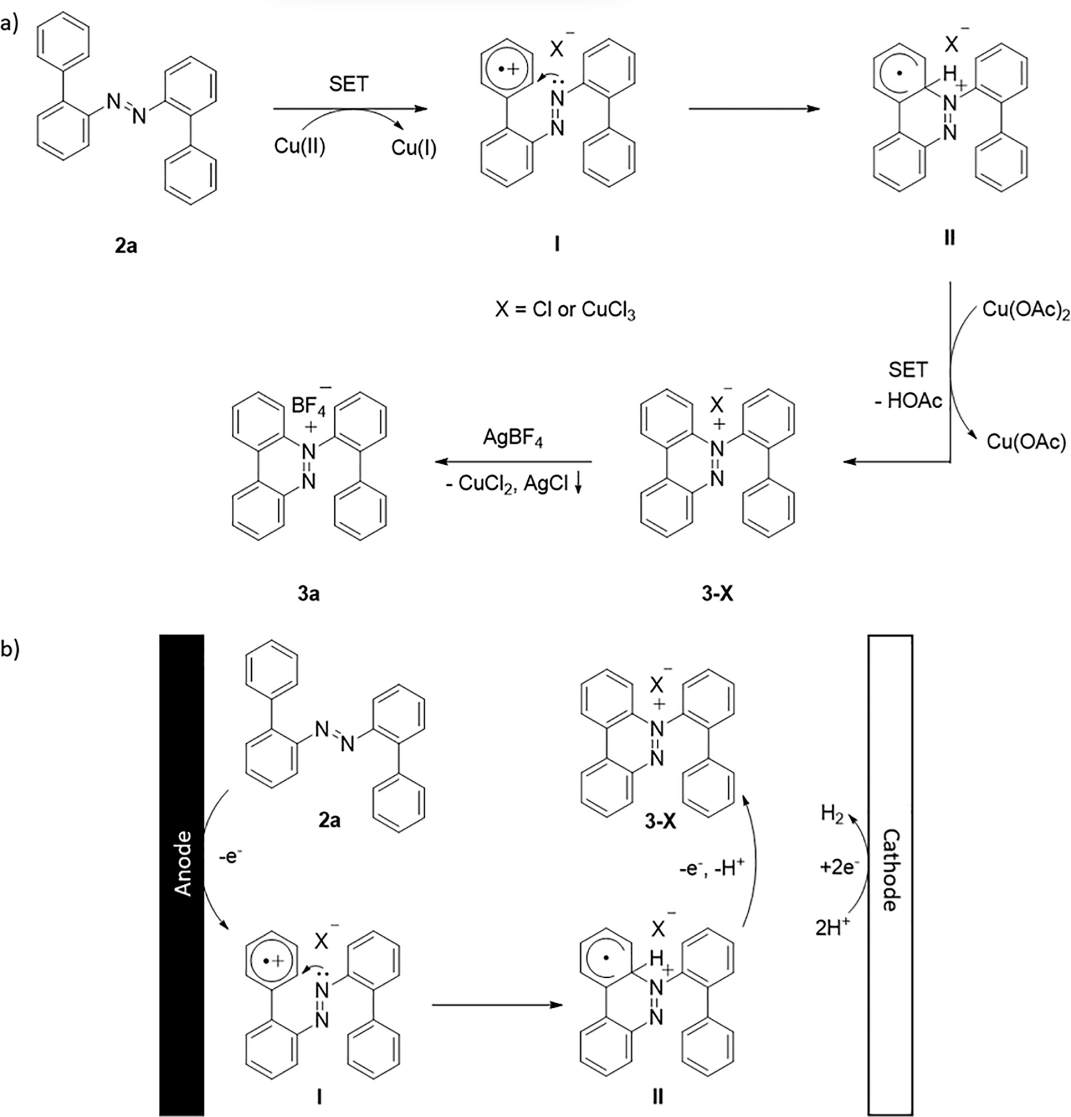
Proposed Reaction Mechanism

In the case of the electrochemical strategy (Scheme 3b), the reaction is initiated by the generation of radical cationic intermediate **I** through anodic oxidation. Next, **I** could undergo intramolecular cyclization via nucleophilic addition to generate intermediate **II**. Finally, single electron oxidation and deprotonation of **II** could afford **3-X** and a proton followed by releasing hydrogen through cathodic reduction.^16^

In summary, we have developed an efficient method for the synthesis of benzo[*c*]cinnolinium salts via intramolecular cyclization promoted by copper(II) or electrochemical oxidation. A variety of diversely functionalized benzo[*c*]cinnolinium salts were easily constructed by this strategy with excellent functional group tolerance and high efficiency. An interesting fluorescence centered at 571 nm is revealed by a benzo[*c*]cinnolinium salt with electron push–pull substitutions. The mechanism is proposed to go through single electron transfer driven by oxidant and intramolecular cyclization via nucleophilic addition, followed by exchanging anions. Copper(II) promoted or electrochemical oxidation can be a simpler and more convenient alternative to traditional methods for the synthesis of benzo[*c*]cinnolinium salts.

## Data Availability

The data underlying this study are available in the published article and its Supporting Information.

## Supporting Information Available

Detailed experimental procedures, spectral data, and ^1^H and ^13^C spectra for compounds (PDF)
FAIR Data including the primary NMR FID files for compounds **2d**–**g**, **2j**–**l**, **2p**, **2v**, and **3a**–**p** (ZIP)

## References

[ref1] aSaito T.; Nishiyama H.; Kawakita K.; Nechayev M.; Kriegel B.; Tsurugi H.; Arnold J.; Mashima K. Reduction of (tBuN = )NbCl_3_(py)_2_ in a Salt-Free Manner for Generating Nb(IV) Dinuclear Complexes and Their Reactivity toward Benzo[*c*]cinnoline. Inorg. Chem. 2015, 54, 6004–6009. 10.1021/acs.inorgchem.5b00812. 26017157

[ref2] Çelik A. C.; Öztürk F.; Erden P. E.; Kaçar C.; Kılıç E. Amperometric Lactate Biosensor Based on Carbon Paste Electrode Modified with Benzo[*c*]cinnoline and Multiwalled Carbon Nanotubes. Electroanalysis 2015, 27, 2820–2828. 10.1002/elan.201500180.

[ref3] Takahashi H.; Ishioka T.; Koiso Y.; Sodeoka M.; Hashimoto Y. Anti-Androgenic Activity of Substituted Azo- and Azoxy-Benzene Derivatives. Biol. Pharm. Bull. 2000, 23, 1387–1390. 10.1248/bpb.23.1387. 11085374

[ref4] Yu Y.; Singh S. K.; Liu A.; Li T.-K.; Liu L. F.; LaVoie E. J. Substituted dibenzo[c,h]cinnolines: topoisomerase I-targeting anticancer agents. Bioorg. Med. Chem. 2003, 11, 1475–1491. 10.1016/S0968-0896(02)00604-1. 12628673

[ref5] aLee S.-W.; Chien S.-H.; Chen J.-C.; Wang S.-H.; Wang L.-Y.; Lai B.-H.; Wang C.-L. Synthesis and characterization of heterocyclic conjugated polymers containing planar benzo[*c*]cinnoline and tetraazapyrene structures for organic field-effect transistor application. Org. Electron. 2019, 66, 136–147. 10.1016/j.orgel.2018.12.027.

[ref6] Shen Y.; Shang Z.; Yang Y.; Zhu S.; Qian X.; Shi P.; Zheng J.; Yang Y. Structurally Rigid 9-Amino-benzo[*c*]cinnoliniums Make Up a Class of Compact and Large Stokes-Shift Fluorescent Dyes for Cell-Based Imaging Applications. J. Org. Chem. 2015, 80, 5906–5911. 10.1021/acs.joc.5b00242. 25951429

[ref7] Takezaki M.; Hirota N.; Terazima M. Excited State Dynamics of 9,10-Diazaphenanthrene Studied by the Time-Resolved Transient Grating Method. J. Phys. Chem. 1996, 100, 10015–10020. 10.1021/jp9602540.

[ref8] aMeng R.; Li F.; Li D.; Jin B. A Green and Efficient Synthesis Method of Benzo[*c*]cinnolines: Electrochemical Reduction of 2,2’-Dinitrobiphenyl in the Presence of CO_2_. ChemElectroChem 2022, 9, e202101381 10.1002/celc.202101381.

[ref9] aXie R.; Lv H.; Ye X.; Kong X.; Li S. Cu-Catalyzed tandem N-arylation of phthalhydrazides with cyclic iodoniums to yield dihydrobenzo[*c*]cinnolines. Org. Biomol. Chem. 2020, 18, 4824–4830. 10.1039/D0OB00894J. 32608470

[ref10] aLee D. S.; Chatterjee T.; Ban J.; Rhee H.; Cho E. J. Simple Synthetic Method for the Functionalized Benzo[*c*]cinnolines. ChemistrySelect 2018, 3, 2092–2095. 10.1002/slct.201800278.

[ref11] aSakai N.; Asama S.; Anai S.; Konakahara T. One-pot preparation of azobenzenes from nitrobenzenes by the combination of an indium-catalyzed reductive coupling and a subsequent oxidation. Tetrahedron 2014, 70, 2027–2033. 10.1016/j.tet.2014.01.048.

[ref12] Cauquis G.; Reverdy G. Etude electrochimique de la cyclodeshydrogenation photochimique du cation triphenyldiazenium. Tetrahedron Lett. 1977, 18, 3267–3270. 10.1016/S0040-4039(01)83214-7.

[ref13] Liu Z.; Xian Y.; Lan J.; Luo Y.; Ma W.; You J. Fusion of Aromatic Ring to Azoarenes: One-Pot Access to 5,6-Phenanthroliniums for Mitochondria-Targeted Far-Red/NIR Fluorescent Probes. Org. Lett. 2019, 21, 1037–1041. 10.1021/acs.orglett.8b04072. 30681876

[ref14] Jayakumar J.; Vedarethinam G.; Hsiao H.-C.; Sun S.-Y.; Chuang S.-C. Cascade One-Pot Synthesis of Orange-Red-Fluorescent Polycyclic Cinnolino[2,3-f]phenanthridin-9-ium Salts by Palladium(II)-Catalyzed C–H Bond Activation of 2-Azobiaryl Compounds and Alkenes. Angew. Chem., Int. Ed. 2020, 59, 689–694. 10.1002/anie.201910959. 31617286

[ref15] Cho S. H.; Yoon J.; Chang S. Intramolecular Oxidative C–N Bond Formation for the Synthesis of Carbazoles: Comparison of Reactivity between the Copper-Catalyzed and Metal-Free Conditions. J. Am. Chem. Soc. 2011, 133, 5996–6005. 10.1021/ja111652v. 21446710

[ref16] Ohno Y.; Ando S.; Furusho D.; Hifumi R.; Nagata Y.; Tomita I.; Inagi S. Synthesis of Cationic Azatriphenylene Derivatives by Electrochemical Intramolecular Pyridination and Characterization of Their Optoelectronic Properties. Org. Lett. 2023, 25, 3951–3955. 10.1021/acs.orglett.3c01341. 37222538 PMC10243103

