# Supplementary material for: Synthesis of Benzo[c]cinnolinium Salts from 2-Azobiaryls by Copper(II) or Electrochemical Oxidation
Source: Org Lett. 2024 Feb 15;26(8):1694–8. doi: 10.1021/acs.orglett.4c00213 (PMC10913080; doi:10.1021/acs.orglett.4c00213)
Supplement: Supplementary file 1 — ol4c00213_si_001.pdf [file ol4c00213_si_001.pdf]

Supporting Information

Synthesis of Benzo[c]cinnolinium Salts from 2-Azobiaryls by Copper(II) or Electrochemical Oxidation

Huan-Chang Hsiao, Meng-Che Li, Guganchandar Vedarethinam, Pei-Lin Chen and Shih-Ching Chuang*

Department of Applied Chemistry, National Yang Ming Chiao Tung University, Hsinchu, Taiwan

E-mail: jscchuang@nycu.edu.tw

Table of contents **Page no**

General information....3

General procedure for the synthesis of 2-aminobiaryls (1) 3

Procedure for the synthesis of 2-cyclopropylaniline 3

General procedure for the synthesis of 2-azobiaryls (2)....4

General procedure for the synthesis of 2-azobiaryls (2r-2t)....4

General procedure for the synthesis of benzo[c]cinnolinium salts (3) 5

General procedure for the synthesis of benzo[c]cinnolinium salts 3 with 1.0 mmol 5

General procedure for the synthesis of benzo[c]cinnolinium salt (3) by electrochemical oxidation (Method A) 6

General procedure for the synthesis of benzo[c]cinnolinium salt (3) by electrochemical oxidation with KPF_6 as electrolytes (Method B)....6

General procedure for the synthesis of benzo[c]cinnolinium salt 3a with 1.0 mmol scale by method A 7

Table S1. Optimization of the electrochemical intramolecular cyclization of 2-azobiaryls....7

Spectral data of 2-azobiaryls 8

Spectral data of salt products 3....13

Photophysical properties....21

Figure S1. UV–vis spectra Benzo[c]cinnolinium salts of 3a-3h. 21

Figure S2. UV–vis absorption spectra of Benzo[c]cinnolinium salts 3i-3p, 3r and 3s....21

Table S2. Photophysical properties of benzo[c]cinnolinium salts 3a and 3p 22

Figure S3. Benzo[c]cinnolinium salts 3a and 3p fluorescence emission spectra. 22

Figure S4. Fluorescence emission of benzo[c]cinnolinium salts 3a (right) and 3p (left). 22

^{1}H and ^{13}C NMR spectra of compounds....23

Cyclic voltammetry 54

Table S3. Cyclic voltammetry study of representative substrates 2a-b, 2g and 2q-s in 0.1 M Bu_4NPF_6/DCM at 23 ºC 55

Figure S5. Cyclic voltammograms use the polarographic plotting convention. Cyclic voltammograms of 2a, 2b, 2g, 2q, 2s and blank (3.0 mM) in 0.1 M Bu_4NPF_6/DCM at 23 ºC started at 0 V. The scan rates were 100 mV/s. 55

Figure S6a. Computed HOMO and LUMO energy levels of 2a-2t 56

Figure S6b. Computed HOMO and LUMO energy levels of 3a-3t cations 56

Computed UV-vis spectra, MOs, and energy levels of 3a-3t cation 57

Atomic coordinates of optimized structures 77

X-ray Crystallographic Analysis 127

Table S4. Crystal data and structure refinement for compound 3i 127

Table S5. Crystal data and structure refinement for compound 3m 128

Scheme S1. Control experiments..129

Reference 129

General information

All commercially available reagents or chemicals, such as $Cu(OAc)_2$, $CuCl_2$, $Pd(OAc)_2$, aromatic amine and alkynes, were used as purchased without further purification unless otherwise noted. All the obtained benzo[c]cinnolinium salts were characterized by melting points (m.p.), ^{1}H NMR, ^{13}C NMR, infrared spectra (IR), high-resolution mass spectrometry (HR-MS), UV-visible absorption spectra and fluorescence spectra. Melting points were performed on an Electrothermal MEL-TEMP melting point apparatus; ^{1}H NMR and ^{13}C NMR spectra were recorded on Agilent 400 MHz and chemical shifts were reported in parts per million (ppm, δ) with chloroform, DMSO or MeOH as the internal standard unless otherwise mentioned. Proton coupling patterns are described as singlet (s), doublet (d), triplet (t), multiplet (m); coupling constants *J* are given in Hz. Carbon-13 nuclear magnetic resonance (^{13}C NMR) data were acquired at 100 MHz unless otherwise mentioned. Yields of synthesized compounds in this work were calculated using 1,3,5-trimethoxybenzene as an internal standard by NMR spectroscopy. IR spectra were recorded on a BRUKER spectrometer. UV-vis absorption and emission spectra in dichloromethane were recorded at room temperature on U-3010 spectrophotometer and F-7000 fluorescence spectrophotometer, respectively. The molar absorption coefficient (ε) of **3** is calculated by Beer's law. The path length is 1 cm, and the concentration of the light-absorbing solution is 1.0×10^{-5} M in dichloromethane. Cyclic voltammograms were obtained on a CHI 621C potentiostat. We perform the computational study using Gaussian 16, Revision B.01 software. Geometry optimizations and frequency calculations are carried out with B3LYP/6-31G+(d,p) for all atoms, and single point energy calculations are performed with B3LYP/6-311G++(2df, 2p) for all atoms.

General procedure for the synthesis of 2-aminobiaryls (1)[1]

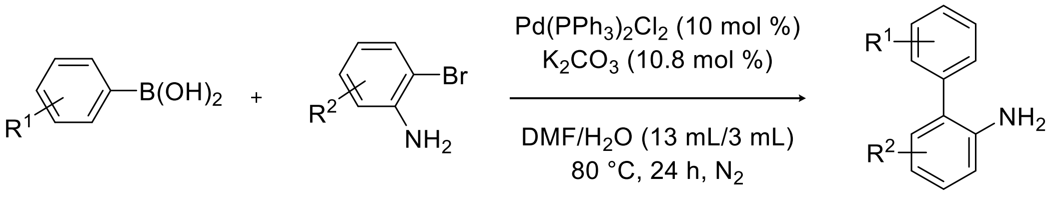

1

To a two-neck round bottom flask containing aryl boronic acid (3.2 mmol), 2-bromoanilines (2.7 mmol), K_2CO_3 (10.8 mmol) and $Pd(PPh_3)_2Cl_2$ (0.27 mmol) in 13 mL/3 mL of DMF/H_2O was stirred at 80 °C for 24 h under nitrogen. The resulting reaction mixture was cooled to room temperature and filter by Celite with ethyl acetate followed by extraction for several time with ethyl acetate and brine. The organic layer was collected and dried over anhydrous Na_2SO_4. After the solution was concentrated in vacuum, the residue was purified by flash column chromatography (2% EA in hexanes) to produce 2-aminobiaryls **1** in good yields.

Procedure for the synthesis of 2-cyclopropylaniline

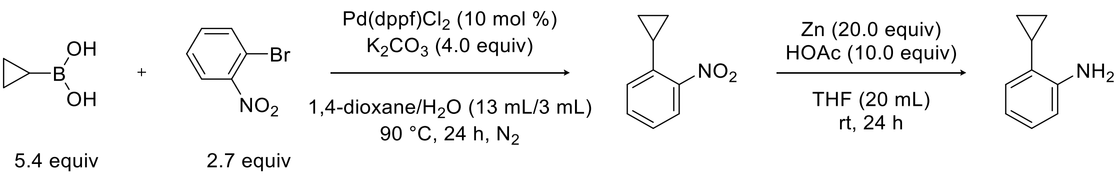

To a two-neck round bottom flask containing cyclopropaneboronic acid (5.4 mmol), 1-bromo-2-nitrobenzene (2.7 mmol), K_2CO_3 (10.8 mmol) and Pd(dppf)Cl_2 (0.27 mmol) in 13 mL/3 mL of 1,4-Dioxane/H_2O was stirred at 90 °C for 24 h under nitrogen. The resulting reaction mixture was cooled to room temperature and filter by Celite with ethyl acetate followed by the extraction several time with ethyl acetate and brine water. The organic layer was collected and dried over anhydrous Na_2SO_4. After the solution was concentrated in vacuum, the residue was purified by flash column chromatography (Hexanes) to produce 2-cyclopropyl-1-nitrobenzene in good yields. Next, to a reaction tube containing 2-cyclopropyl-1-nitrobenzene (1.5 mmol) and Zn (30 mmol) in 20 mL of THF, followed by the dropwise addition of HOAc (15 mmol), was stirred at room temperature for 24 h. The resulting reaction mixture was filtered by Celite with ethyl acetate followed by the extraction three time with ethyl acetate and saturated solution of sodium bicarbonate. The organic layer was collected and dried over Na_2SO_4. After the solution was concentrated in vacuum, the residue was purified by flash column chromatography (Hexanes) to produce 2-cyclopropylaniline in good yields.

General procedure for the synthesis of 2-azobiaryls (2)[2, 3]

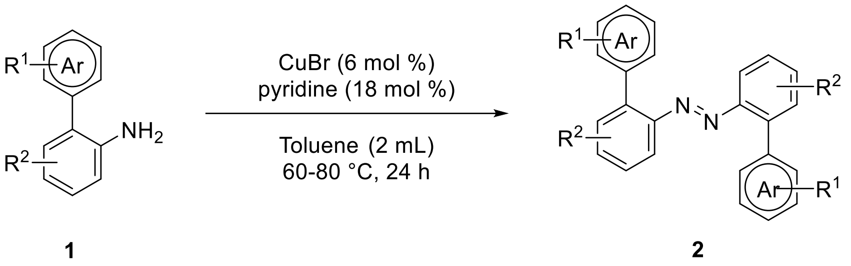

To a glass reaction tube containing 2-aminobiaryls (**1**, 0.5 mmol) and CuBr (4.3 mg, 0.03 mmol) in 2 mL of toluene was added pyridine (3 drops, 0.09 mmol). The resulting mixture was stirred at 80 °C for 24 h. After completion of the reaction, the mixture was cooled to room temperature and concentrated in vacuum. Then the residue was purified by flash column chromatography (hexanes/ethyl acetate) to provide 2-azobiaryls **2** in moderate yields.

General procedure for the synthesis of 2-azobiaryls (2r-2t)[4, 5]

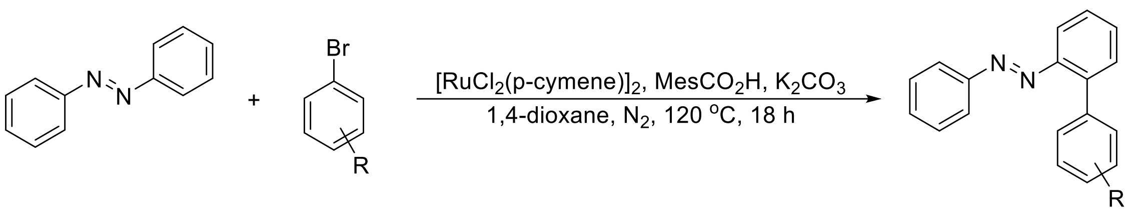

To a screw-capped sealed tube, a mixture of azobenzene (91.1 mg, 0.5 mmol), [RuCl_2(p-cymene)]$_2$ (15.3 mg, 5.0 mol %), MesCO_2H (24.6 mg, 30 mol %), K_2CO_3 (138 mg, 1.0 mmol) and bromobenzene (39.3 mg, 0.25 mmol) in 1,4-dioxane (2.0 mL) was stirred at 120 °C for 18 h under a N_2 atmosphere. After the reaction was completed, the reaction mixture was filtered through Celite and concentrated in vacuum. Then, the crude product was purified by column chromatography on silica gel (n-hexane/EA:99/1) to provide **2r**.

General procedure for the synthesis of benzo[c]cinnolinium salts (3)

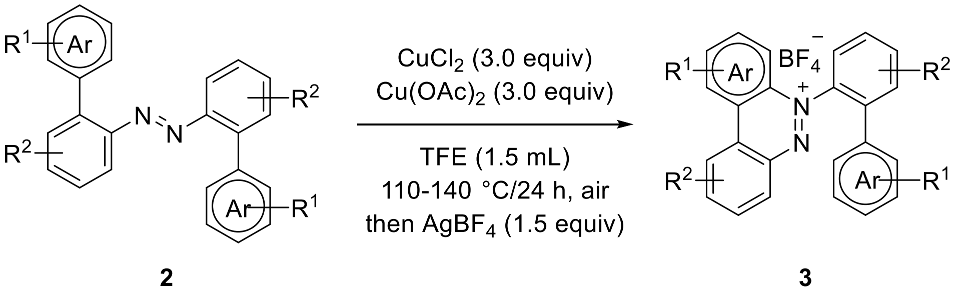

To a pressure-affordable sealed tube with 35 cm^3 inner volume containing 2-azobiaryls (**2**, 0.1 mmol), $CuCl_2$ (40.3 mg, 0.3 mmol) and $Cu(OAc)_2$ (54.5 mg, 0.3 mmol) in 1.5 mL of trifluoroethanol (TFE) was heated for 24 h at 110-140°C under aerobic condition. **CAUTION!** Due to the operating temperature higher than the boiling point of TFE (78 °C), an explosion may occur. A thick-walled and pressure-affordable tube must be used. The resulting mixture was cooled to room temperature and filtered by Celite with CH_2Cl_2, and the solution was directly purified by flash column chromatography (ethyl acetate/methanol or CH_2Cl_2/methanol) to produce compound **3-X**. Then, Compound **3-X** was treated with $AgBF_4$ (30 mg, 0.15 mmol) in 3-5 mL of CH_2Cl_2. The resulting solution was stirred at room temperature for 30 minutes. After the anion exchange was completed, the resulting solution was filtered by Celite and reprecipitated by CH_2Cl_2 and n-hexane to afford the desired quaternary ammonium salts **3** in moderate to good yields.

General procedure for the synthesis of benzo[c]cinnolinium salts 3 with 1.0 mmol

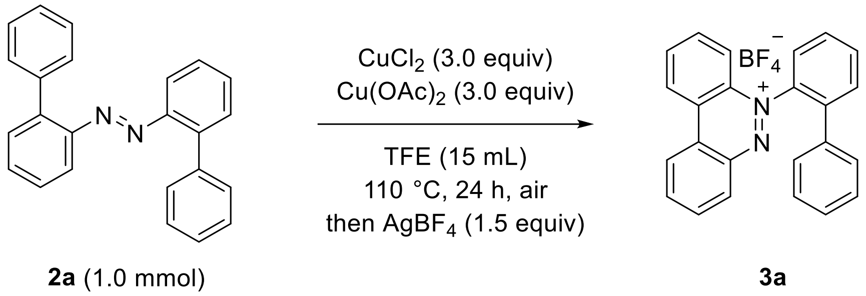

To a pressure-affordable sealed tube with 35 cm^3 inner volume containing 2-azobiphenyls (**2a**, 334.4 mg, 1.0 mmol), $CuCl_2$ (403.4 mg, 3.0 mmol) and $Cu(OAc)_2$ (544.9 mg, 3.0 mmol) in 15 mL of trifluoroethanol (TFE) was heated for 24 h at 110°C under aerobic condition. The resulting mixture was cooled to room temperature and filtered by Celite with CH_2Cl_2, and the solution was directly purified by flash column chromatography (CH_2Cl_2/methanol) to produce compound **3-X**. Then, Compound **3-X** was treated with $AgBF_4$ (300 mg, 1.5 mmol) in 30-50 mL of CH_2Cl_2. The resulting solution was stirred at room temperature for 30 minutes. After the anion exchange was completed, the resulting solution was filtered by Celite and reprecipitated by CH_2Cl_2 and n-hexane to afford the desired quaternary ammonium salts **3a** in 99% yield (416 mg).

General procedure for the synthesis of benzo[c]cinnolinium salt (3) by electrochemical oxidation (Method A)

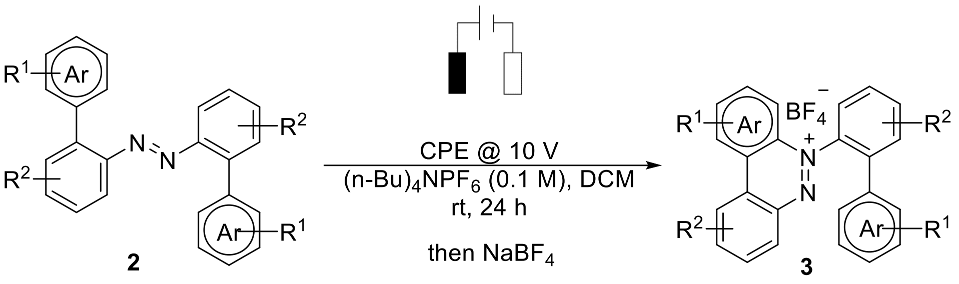

A 15 mL tubular vial was charged with substrate **2** (0.025 mmol), (n-Bu)$_4$NPF$_6$ (0.1 M), DCM (3.0 mL) and a magnetic stir bar. The vial was equipped with platinum electrodes (2.0 cm × 1.0 cm × 0.1 mm) as an anode and a glassy carbon as a cathode. The whole cell was an undivided cell. The reaction mixture was stirred and electrolyzed at a constant voltage of 10.0 V without reference electrode under room temperature for 24 h. After completion of the reaction, the mixture was concentrated in vacuo followed by anion exchange. Then appropriate amounts of internal standard were added for checking yields by ^{1}H NMR spectroscopy.

General procedure for the synthesis of benzo[c]cinnolinium salt (3) by electrochemical oxidation with KPF$_6$ as electrolytes (Method B)

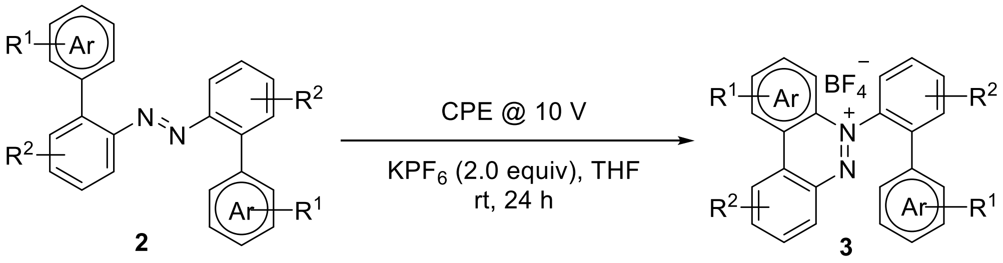

The electrooxidation was performed in an undivided cell with a platinum anode (2.0 cm × 1.0 cm × 0.1 mm) and a carbon cathode. Substrate **2** (0.025 mmol) and KPF$_6$ (2.0 equiv) were added in THF (3 mL). The electrochemical oxidation was carried out at room temperature with a constant voltage of 10.0 V without a reference electrode and maintained for 24 h. Upon completed, the solution was transferred to a round-bottomed flask with DCM and concentrated in vacuo followed by anion exchange. Then appropriate amounts of internal standard were added for checking yields by ^{1}H NMR spectroscopy.

General procedure for the synthesis of benzo[c]cinnolinium salt 3a with 1.0 mmol scale by method A

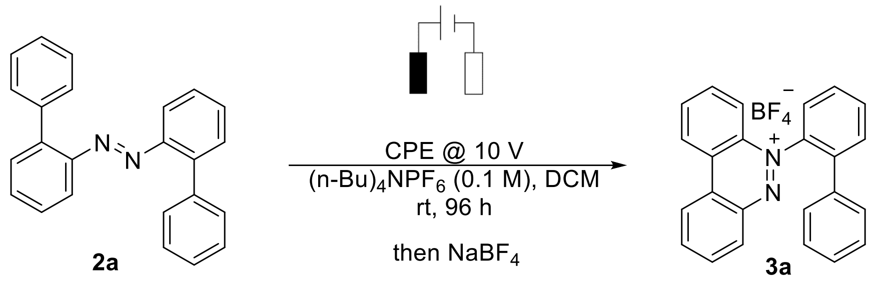

A suitable container was charged with substrate **2a** (334.4 mg, 1.0 mmol), $(n\text{-Bu})_4NPF_6$ (0.1 M), DCM (120.0 mL) and a magnetic stir bar. The container was equipped with platinum electrodes (4.0 cm × 1.0 cm × 0.1 mm) as an anode and a glassy carbon as a cathode. The whole cell was an undivided cell. The reaction mixture was stirred and electrolyzed at a constant voltage of 10.0 V without reference electrode under room temperature for 96 h. After completion of the reaction, the mixture was concentrated in vacuo followed by anion exchange. Then appropriate amounts of internal standard were added for checking yields, giving 56% (0.56 mmol) by ^{1}H NMR spectroscopy.

Table S1. Optimization of the electrochemical intramolecular cyclization of 2-azobiaryls[a]

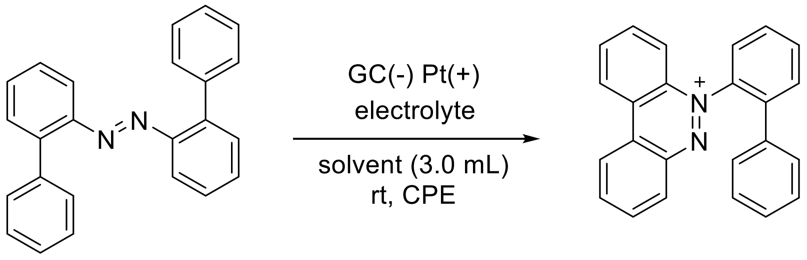

Entry	Potential(V)	Electrolyte	Time(h)	Solvent(mL)	Yield(%)[b]
1	1	$(n\text{-Bu})_4NPF_6$ (0.1 M)	24	DCM	0
2	5	$(n\text{-Bu})_4NPF_6$ (0.1 M)	24	DCM	79
3	8	$(n\text{-Bu})_4NPF_6$ (0.1 M)	24	DCM	90
4	10	$(n\text{-Bu})_4NPF_6$ (0.1 M)	24	DCM	99
5	10	$(n\text{-Bu})_4NBF_4$ (0.1 M)	24	DCM	37
6	10	$(n\text{-Bu})_4NPF_6$ (0.1 M)	6	DCM	68
7	10	$(n\text{-Bu})_4NPF_6$ (0.1 M)	3	DCM	59
8	10	KPF_6 (2.0 equiv)	24	DCM	0
9	10	KPF_6 (2.0 equiv)	24	TFE	37
10	10	KPF_6 (2.0 equiv)	24	ACN	0
11	10	KPF_6 (2.0 equiv)	24	THF	90

[a]Substrate **2** (0.025 mmol), electrolyte, solvent, undivided cell with Pt electrode (1.0×2.0 cm^2) and glassy carbon electrode, constant voltage at room temperature. [b]Yields were determined by ^{1}H NMR spectroscopic using 1,3,5-trimethoxybenzene as an internal standard unless otherwise noted.

Spectral data of 2-azobiaryls

(*E*)-1,2-bis(4'-methyl-[1,1'-biphenyl]-2-yl)diazene (2b)

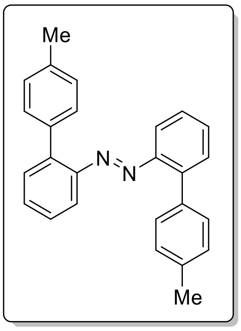

Orange solid (144.9 mg, 40 % yield); R_f = 0.23 (ethyl acetate/hexanes = 0.2/99.8); m.p. 209-211 ºC; **^{1}H NMR** (400 MHz, $CDCl_3$): δ 7.55 (d, *J* = 7.6 Hz, 2H), 7.51-7.46 (m, 4H), 7.40-7.33 (m, 6H), 7.28 (s, 4H), 2.45 (s, 6H); **^{13}C NMR** (100 MHz, $CDCl_3$): δ 149.9, 141.3, 137.0, 135.9, 130.8, 130.7, 130.5, 128.4, 127.7, 116.4, 21.2; **HRMS** (ESI$^+$) calcd for $C_{26}H_{22}N_2Na$ 385.1681, found 385.1681; **IR** (KBr): 3059, 1611, 1514 and 838 cm^{-1}.

(*E*)-1,2-bis(2'-methyl-[1,1'-biphenyl]-2-yl)diazene (2d)

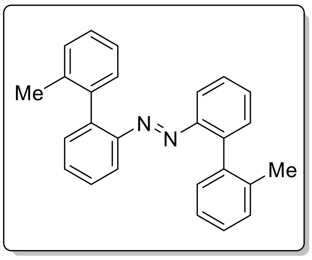

Red solid (190.8 mg, 50% yield); R_f = 0.25 (ethyl acetate/hexanes = 0.2/99.8); m.p. 136-138 °C; **^{1}H NMR** (400 MHz, $CDCl_3$): δ 7.45 (t, *J* = 7.2 Hz, 2H), 7.39-7.21 (m, 14H), 2.11 (s, 6H); **^{13}C NMR** (100 MHz, $CDCl_3$): δ 150.3, 141.8, 139.3, 136.5, 130.9, 130.4(2C), 129.5, 128.0, 127.4, 125.1, 115.6, 20.6; **HRMS** (ESI$^+$) (M+H) calcd for $C_{26}H_{23}N_2^+$ 363.1856, found 363.1858; **IR** (KBr): 2917, 2850, 1731, 1469, 776, 754 and 725 cm^{-1}.

(*E*)-1,2-bis(4'-methoxy-[1,1'-biphenyl]-2-yl)diazene (2e)

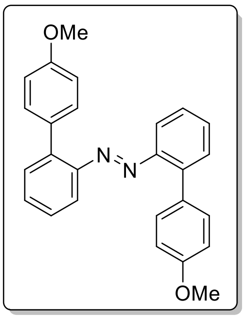

Red solid (159.1 mg, 36% yield); R_f = 0.33 (dichloromethane/hexanes = 2/3); m.p. 236-238 °C; **^{1}H NMR** (400 MHz, $CDCl_3$): δ 7.55 (d, *J* = 7.2 Hz, 2H), 7.51-7.41 (m, 8H), 7.34 (t, *J* = 8.4 Hz, 2H), 7.01 (d, *J* = 8.4 Hz, 4H), 3.89 (s, 6H); **^{13}C NMR** (100 MHz, $CDCl_3$): δ 159.1, 149.9, 141.0, 132.1, 131.4, 130.7, 130.6, 127.6, 116.4, 113.2, 55.3; **HRMS** (ESI^+) (M+H) calcd for $C_{26}H_{23}O_2N_2^+$ 395.1754, found 395.1751; **IR** (KBr): 2917, 2849, 1733, 1462, 770, 752 and 719 cm^{-1}.

(*E*)-1,2-bis(3'-methoxy-[1,1'-biphenyl]-2-yl)diazene (2f)

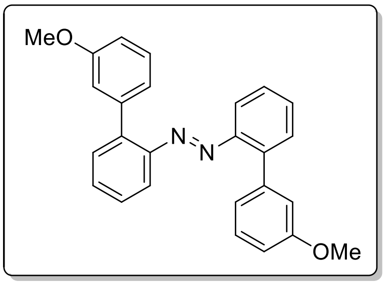

Red solid (216.7 mg, 55% yield); R_f = 0.20 (ethyl acetate/hexanes = 0.4/99.6); m.p. 116-118 °C; **^{1}H NMR** (400 MHz, $CDCl_3$): δ 7.57 (d, *J* = 8.0 Hz, 2H), 7.49 (t, *J* = 8.2 Hz, 4H), 7.37 (t, *J* = 7.6 Hz, 4H), 7.07 (d, *J* = 8.0 Hz, 4H), 6.97 (t, *J* = 8.0 Hz, 2H), 3.84 (s, 6H); **^{13}C NMR** (100 MHz, $CDCl_3$): δ 159.0, 150.0, 141.3, 140.4, 130.8, 130.6, 128.6, 128.1, 123.6, 116.4, 116.3, 113.2, 55.3; **HRMS** (ESI^+) (M+H) calcd for $C_{26}H_{23}O_2N_2^+$ 395.1754, found 395.1761; **IR** (KBr): 2918, 2850, 1734, 1465, 769, 747 and 724 cm^{-1}.

(*E*)-1,2-bis(4'-fluoro-[1,1'-biphenyl]-2-yl)diazene (2g)

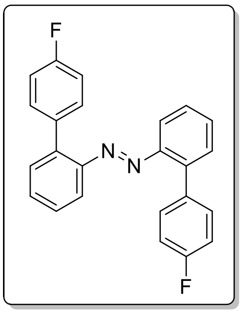

Red solid (240.0 mg, 50% yield); R_f = 0.40 (dichloromethane/hexanes = 1/19); m.p. 176-178 °C; **^{1}H NMR** (400 MHz, $CDCl_3$): δ 7.56-7.43 (m, 10H), 7.39 (t, *J* = 7.4 Hz, 2H), 7.17 (t, *J* = 8.6 Hz, 4H); **^{13}C NMR** (100 MHz, $CDCl_3$): δ 163.7, 161.2, 149.6, 140.6, 134.89, 134.85, 132.4, 132.3, 130.9, 130.8, 128.2, 116.2, 114.7, 114.5; **HRMS** (ESI^+) (M+H) calcd for $C_{24}H_{17}N_2F_2^+$ 371.1354, found 371.1357; **IR** (KBr): 2918, 2850, 1733, 1469 and 746, cm^{-1}.

(*E*)-1,2-bis(4-fluoro-[1,1'-biphenyl]-2-yl)diazene (2j)

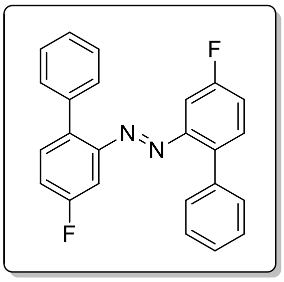

Red solid (227.7 mg, 46% yield); R_f = 0.31 (dichloromethane/hexanes = 3/22); m.p. 200-202 °C; **^{1}H NMR** (400 MHz, $CDCl_3$): δ 7.55 (t, *J* = 7.0 Hz, 2H), 7.52-7.40 (m, 10H), 7.29-7.16 (m, 4H); **^{13}C NMR** (100 MHz, $CDCl_3$): δ 163.6, 161.1, 150.4, 150.3, 138.38, 138.35, 137.9, 132.4, 132.3, 130.8, 127.8, 127.6, 118.2, 118.0, 103.1, 102.9; **HRMS** (ESI$^+$) (M+H) calcd for $C_{24}H_{17}N_2F_2^+$ 371.1354, found 371.1352; **IR** (KBr): 2917, 2849, 1730, 1447, 771, 744 and 734 cm^{-1}.

(*E*)-1,2-bis(4,4'-dimethyl-[1,1'-biphenyl]-2-yl)diazene (2k)

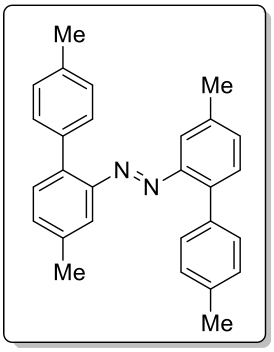

Red solid (218.7 mg, 56% yield); R_f = 0.25 (dichloromethane/hexanes = 1/19); m.p. 217-219 °C; **^{1}H NMR** (400 MHz, $CDCl_3$): δ 7.47 (d, *J* = 8.4 Hz, 2H), 7.41-7.37 (m, 4H), 7.33-7.29 (m, 4H), 7.28-7.24 (m, 4H), 2.45 (s, 6H), 2.39 (s, 6H); **^{13}C NMR** (100 MHz, $CDCl_3$): δ 149.9, 138.3, 137.6, 136.9, 135.9, 131.3, 130.8, 130.5, 128.4, 116.9, 21.24, 21.22; **HRMS** (ESI$^+$) (M+H) calcd for $C_{28}H_{27}N_2^+$ 391.2169, found 391.2168; **IR** (KBr): 2915, 1729, 1482 and 807 cm^{-1}.

(*E*)-1,2-bis(4'-fluoro-4-methyl-[1,1'-biphenyl]-2-yl)diazene (2l)

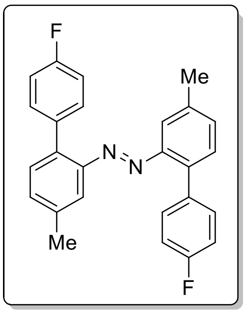

Red solid (248.9 mg, 52% yield); R_f = 0.22 (dichloromethane/hexanes = 1/19); m.p. 218-220°C; **^{1}H NMR**

(400 MHz, $CDCl_3$): δ 7.47-7.40 (m, 6H), 7.32 (d, *J* = 7.6 Hz, 2H), 7.28 (s, 2H), 7.14 (t, *J* = 8.6 Hz, 4H), 2.39 (s, 6H) ; **^{13}C NMR** (100 MHz, $CDCl_3$): δ 163.6, 161.2, 149.6, 138.1, 137.7, 134.9, 134.8, 132.44, 132.36, 131.7, 130.6, 116.6, 114.6, 114.4, 21.3; **HRMS** (ESI^+) (M+H) calcd for $C_{26}H_{21}N_2F_2^+$ 399.1667, found 399.1662; **IR** (KBr): 2916, 2849, 1732, 1486 and 806 cm^{-1}.

(*E*)-1,2-bis(2-(naphthalen-1-yl)phenyl)diazene (2n)

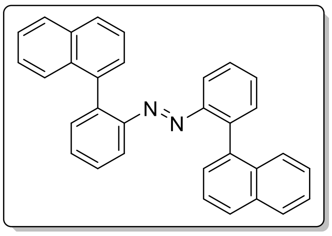

Orange solid (117.2 mg, 27 % yield); R_f = 0.21 (ethyl acetate/hexanes = 0.2/99.8); m.p. 203-204 °C; **^{1}H NMR** (400 MHz, $CDCl_3$): δ 7.94 (t, *J* = 6.9 Hz, 4H), 7.61-7.53 (m, 3H), 7.50-7.34 (m, 10H), 7.29 (t, *J* = 7.6 Hz, 1H), 7.16 (q, *J* = 7.7 Hz, 2H), 6.77 (d, *J* = 8.1 Hz, 1H), 6.63 (d, *J* = 8.1 Hz, 1H); **^{13}C NMR** (100 MHz, $CDCl_3$): δ 151.1, 151.0, 139.9, 139.8, 137.3, 137.2, 133.3, 133.2, 132.9, 132.6, 131.6, 131.4, 130.8, 130.2, 130.1, 128.4, 128.2, 128.1, 128.0, 127.9, 127.87, 127.8, 126.8, 126.7, 125.93, 125.91, 125.5, 125.4, 125.0, 124.9, 115.9, 115.8; **HRMS** (ESI^+) calcd for $C_{32}H_{22}N_2Na$ 457.1681, found 457.1682; **IR** (KBr): 3055, 1590 and 1506 cm^{-1}. [Note: The ^{13}C NMR shows two diastereomers]

(*E*)-1,2-bis(2-(naphthalen-2-yl)phenyl)diazene (2o)

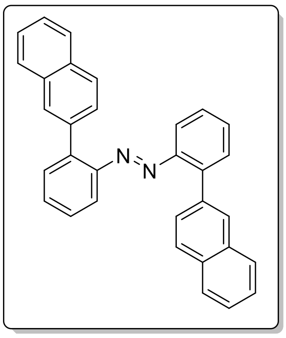

Red solid (40 mg, 8 %); R_f = 0.22 (ethyl acetate/hexanes = 0.2/99.8); m.p. 218-221 °C; **^{1}H NMR** (400 MHz, $CDCl_3$): δ 7.97-7.90 (m, 8H), 7.67 (d, *J* = 8.2 Hz, 4H), 7.53 (t, *J* = 8.8 Hz, 8H), 7.34 (t, *J* = 7.7 Hz, 2H); **^{13}C NMR** (100 MHz, $CDCl_3$): δ 149.8, 141.4, 136.6, 133.1, 132.5, 131.0, 130.7, 129.6, 129.3, 128.2 (2C), 127.6, 126.8, 126.2, 126.1, 116.3; **HRMS** (ESI^+) calcd for $[C_{32}H_{23}N_2]^+$ 435.1856, found 435.1864; **IR** (KBr): 3053, 2918, 1593, and 1460 cm^{-1}.

(*E*)-1-(4'-fluoro-[1,1'-biphenyl]-2-yl)-2-(4'-methoxy-[1,1'-biphenyl]-2-yl)diazene (2p)

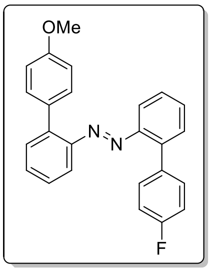

Red solid (60.0 mg , 7% yield); R_f = 0.30 (dichloromethane/hexanes = 1/19); m.p. 224-226 °C; **^{1}H NMR** (400 MHz, $CDCl_3$): δ 7.60-7.42 (m, 10H), 7.42-7.33 (m, 2H), 7.17 (t, *J* = 8.6 Hz, 2H), 7.03 (d, *J* = 8.4 Hz, 2H), 3.91 (s, 3H) ; **^{13}C NMR** (100 MHz, $CDCl_3$): δ 163.7, 161.2, 159.1, 149.8, 149.7, 141.2, 140.4, 135.0, 134.9, 132.4, 132.3, 132.0, 131.3, 130.81, 130.76, 130.70, 130.67, 128.2, 127.6, 116.4, 116.2, 114.7, 114.5, 113.2, 55.3; **HRMS** (ESI^+) (M+H) calcd for $C_{25}H_{20}OFN_2^+$ 383.1554, found 383.1550; **IR** (KBr): 2917, 2849, 1730, 1469, 771, 752 and 719 cm^{-1}.

(*E*)-1,2-bis(4'-(trifluoromethyl)-[1,1'-biphenyl]-2-yl)diazene (2q)

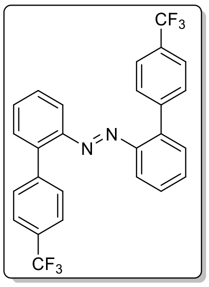

Red solid (42.3 mg, 36% yield); R_f = 0.65 (hexanes/EA = 9.5:0.5); mp: 233-236 °C; **^{1}H NMR** ($CDCl_3$, 400 MHz) δ 7.71 (d, *J* = 8.0 Hz, 4H), 7.60 (d, *J* = 8.0 Hz, 4H), 7.56–7.54 (m, 4H), 7.50–7.42 (m, 4H) ppm; **^{13}C NMR** ($CDCl_3$, 100 MHz) δ 149.3, 142.5, 140.2, 131.1, 130.9, 130.7, 129.2 (d, $^2J_{FC}$ = 32.3 Hz), 128.8, 124.2 (d, $^1J_{FC}$ = 270.0 Hz), 124.4 (q, $^3J_{FC}$ = 3.7 Hz), 116.1 ppm; HRMS (ESI^+) calcd for $C_{26}H_{16}N_2F_6$ 470.1223, found 470.1221; **IR** (KBr) 2955, 2919, 1734, 1463, 1402, 1329, 1160, 1109, 1071, 881, 774, 760, 734, 609 cm^{-1}.

(*E*)-1,2-bis(2-cyclopropylphenyl)diazene (2v)

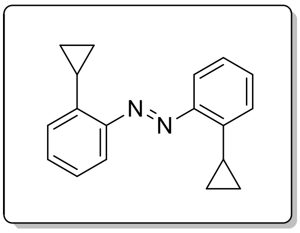

Red solid (204.6 mg, 76% yield); R_f = 0.29 (ethyl acetate/hexanes = 1/99); m.p. 110-112 °C; **^{1}H NMR** (400

MHz, $CDCl_3$): δ 7.66 (dd, *J* = 8.0 Hz, *J* = 1.2 Hz, 2H), 7.37 (td, *J* = 7.4 Hz, *J* = 1.2 Hz, 2H), 7.23 (td, *J* = 7.6 Hz, *J* = 1.2 Hz, 2H), 6.99 (d, *J* = 7.6 Hz, 2H), 3.07 (tt, *J* = 8.6 Hz, *J* = 1.2 Hz, 2H), 1.16-1.10 (m, 4H), 0.92-0.88 (m, 4H); **^{13}C NMR** (100 MHz, $CDCl_3$): δ 151.4, 143.7, 130.9, 125.5, 124.1, 115.6, 10.3, 10.1; **HRMS** (ESI$^+$) (M+H) calcd for $C_{18}H_{19}N_2^+$ 263.1543, found 263.1542; **IR** (KBr): 3083, 3065, 2995, 1595, 1482, 1428, 1215, 1150, 1047, 1028, 796 and 766 cm^{-1}.

Spectral data of salt products 3

5-([1,1'-biphenyl]-2-yl)benzo[c]cinnolin-5-ium tetrafluoroborate (3a)

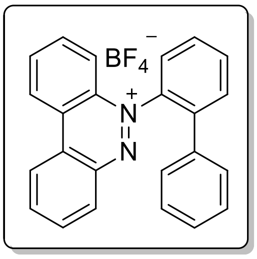

Yellow solid (41.2 mg, 98% yield); R_f = 0.30 (methanol/ethyl acetate = 1/4); m.p. 266-268 °C; **^{1}H NMR** (400 MHz, DMSO-d_6): δ 9.32 (dd, *J* = 8.6 Hz, *J* = 3.0 Hz, 2H), 8.94 (d, *J* = 8.8 Hz, 1H), 8.68 (t, *J* = 7.8 Hz, 1H), 8.46 (t, *J* = 7.8 Hz, 1H), 8.36 (t, *J* = 7.6 Hz, 1H), 8.17 (t, *J* = 7.8 Hz, 1H), 8.10 (d, *J* = 8.8 Hz, 2H), 8.03 (t, *J* = 7.6 Hz, 1H), 7.93 (t, *J* = 7.6 Hz, 1H), 7.85 (d, *J* = 7.6 Hz, 1H), 7.07-6.96 (m, 5H); **^{13}C NMR** (100 MHz, DMSO-d_6): δ 143.1, 141.6, 140.5, 138.6, 138.0, 135.72, 135.70, 134.8, 134.3, 133.0 (2C), 131.7, 129.9, 128.8, 128.57, 128.55, 128.1, 127.5, 126.8, 124.5, 123.4, 121.8; **HRMS** (ESI$^+$) calcd for $C_{24}H_{17}N_2^+$ 333.1392, found 333.1388; **IR** (KBr): 2951, 1732, 1608, 1572, 1396, 1054 and 765 cm^{-1}.

3-methyl-5-(4'-methyl-[1,1'-biphenyl]-2-yl)benzo[c]cinnolin-5-ium tetrafluoroborate (3b)

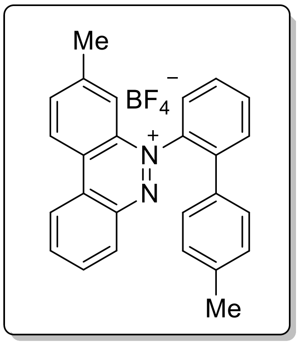

Yellow solid (44.4 mg, 99% yield); R_f = 0.30 (methanol/ethyl acetate = 1/4); m.p. 218-220 °C; **^{1}H NMR** (400 MHz, CD_3OD): δ 9.17 (d, *J* = 8.4 Hz, 1H), 9.11 (d, *J* = 8.8 Hz, 1H), 8.82 (d, *J* = 8.8 Hz, 1H), 8.58 (t, *J* = 7.6 Hz, 1H), 8.38 (t, *J* = 7.8 Hz, 1H), 8.17 (d, *J* = 8.4 Hz, 1H), 8.03-7.93 (m, 2H), 7.90-7.84 (m, 1H), 7.84-7.77 (m, 2H), 6.91-6.77 (m, 4H), 2.63 (s, 3H), 2.05 (s, 3H); **^{13}C NMR** (100 MHz, CD_3OD): δ 148.2, 144.8, 142.13, 142.11, 140.3, 140.0, 139.9, 138.7, 134.7, 134.5, 133.9, 133.8, 132.9, 130.6, 130.3, 129.0, 128.6, 128.5, 128.2, 125.1, 124.0, 121.3, 22.3, 20.8; **HRMS** (ESI$^+$) calcd for $C_{26}H_{21}N_2^+$ 361.1705, found 361.1714; **IR** (KBr): 2955, 1731, 1609, 1566, 1403, 1057 and 773 cm^{-1}.

2-methyl-5-(3'-methyl-[1,1'-biphenyl]-2-yl)benzo[c]cinnolin-5-ium tetrafluoroborate (3c)

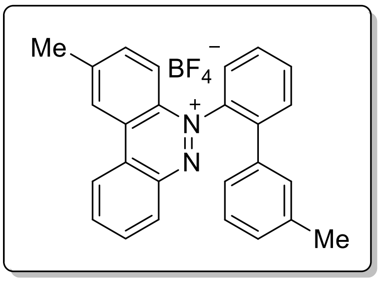

Yellow solid (40.8 mg, 91% yield); R_f = 0.30 (methanol/ethyl acetate = 3/7); m.p. 154-156 °C; **^{1}H NMR** (400 MHz, $CDCl_3$): δ 9.12 (d, *J* = 8.4 Hz, 1H), 8.90 (s, 1H), 8.65 (d, *J* = 7.6 Hz, 1H), 8.47 (t, *J* = 6.4 Hz, 1H), 8.24 (t, *J* = 7.6 Hz, 1H), 7.94-7.74 (m, 5H), 7.70 (d, *J* = 7.6 Hz, 1H), 6.87-6.74 (m, 3H), 6.62 (d, *J* = 6.8 Hz, 1H), 2.72 (s, 3H), 1.99 (s, 3H); **^{13}C NMR** (100 MHz, $CDCl_3$): δ 148.3, 143.2, 140.8, 140.4, 138.6, 138.5, 137.3, 136.4, 135.4, 133.6, 132.5, 132.2, 131.5, 129.6, 129.3, 128.9, 128.7, 128.4, 127.1, 126.3, 124.6, 123.61, 123.58, 120.8, 22.6, 21.0; **HRMS** (ESI$^+$) calcd for $C_{26}H_{21}N_2^+$ 361.1705, found 361.1702; **IR** (KBr): 2954, 1729, 1609, 1570, 1397, 1040 and 772 cm^{-1}.

1-methyl-5-(2'-methyl-[1,1'-biphenyl]-2-yl)benzo[c]cinnolin-5-ium tetrafluoroborate (3d)

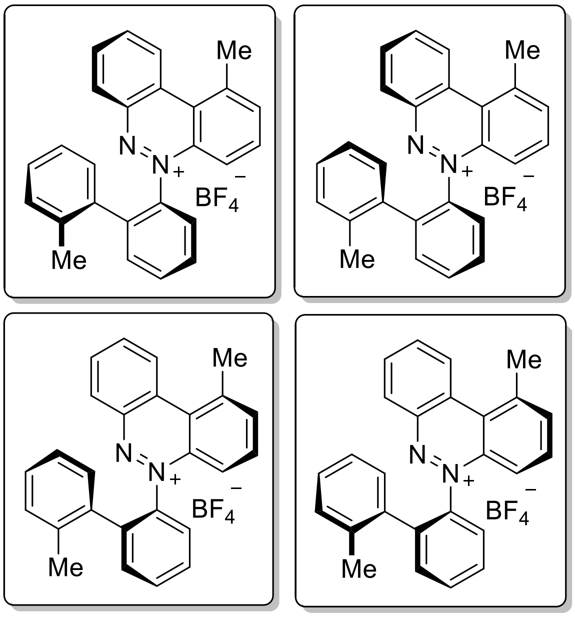

Yellow solid (14.8 mg, 33% yield); R_f = 0.20 (methanol/chloroform = 3/47); m.p. 246-248 °C; **^{1}H NMR** (400 MHz, CD_3OD): δ 9.36-9.30 (m, 2H), 8.98 (d, *J* = 8.4 Hz, 1H), 8.74 (d, *J* = 8.4 Hz, 1H), 8.63 (t, *J* = 8.0 Hz, 1H), 8.57 (t, *J* = 8.2 Hz, 1H), 8.48-8.39 (m, 1H), 8.38-8.24 (m, 5H), 8.21-8.16 (m, 2H),8.10-8.03 (m, 2H), 7.98 (d, *J* = 8.0 Hz, 2H), 7.91 (d, *J* = 6.4 Hz, 2H), 7.75 (d, *J* = 7.2 Hz, 1H), 7.71 (d, *J* = 7.6 Hz, 1H), 6.98 (d, *J* = 7.6 Hz, 1H), 6.94 (d, *J* = 8.0 Hz, 1H), 6.90-6.85 (m, 2H),6.83 (d, *J* = 8.0 Hz, 1H), 6.76 (d, *J* = 7.6 Hz, 1H), 6.65-6.59 (m, 2H), 3.31 (s, 3H), 3.26 (s, 3H), 3.24 (s, 3H), 2.19 (s, 3H) ; **^{13}C NMR** (100 MHz, CD_3OD): δ144.9, 143.4, 143.1, 142.0, 141.9, 141.6, 141.1, 140.6, 139.9, 139.84, 139.78, 139.3, 137.9, 136.9, 136.2, 135.2, 134.7, 134.5, 134.14, 134.11, 134.08, 133.7, 133.6, 133.5, 133.4, 133.2, 131.8, 131.3, 131.1, 130.70, 130.65, 130.6, 129.9, 129.8, 129.7, 129.4, 128.9, 128.7, 128.0, 127.83, 127.81, 127.6, 126.7, 126.23, 126.20, 126.1, 122.0, 121.5, 26.03, 25.98, 20.4, 20.2; **HRMS** (ESI$^+$) calcd for $C_{26}H_{21}N_2^+$ 361.1710, found 361.1699; **IR** (KBr): 2956, 1730, 1596, 1572, 1387, 1057 and 762 cm^{-1}.

3-methoxy-5-(4'-methoxy-[1,1'-biphenyl]-2-yl)benzo[c]cinnolin-5-ium tetrafluoroborate (3e)

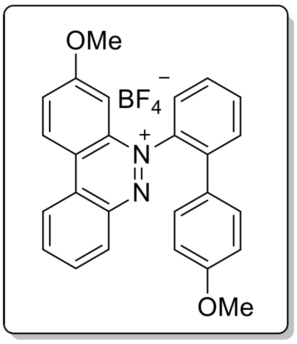

Orange solid (33.1 mg, 69% yield); R_f = 0.28 (methanol/ dichloromethane = 1/9); m.p. 214-216 °C; **^{1}H NMR** (400 MHz, $CD_3OD/CDCl_3$ = 6/1): δ 9.12 (d, *J* = 9.6 Hz, 1H), 9.06 (d, *J* = 8.4 Hz, 1H), 8.74 (d, *J* = 8.4 Hz, 1H), 8.51 (t, *J* = 7.6 Hz, 1H), 8.30 (t, *J* = 7.8 Hz, 1H), 7.97 (t, *J* = 7.4 Hz, 1H), 7.94-7.88 (m, 2H), 7.87-7.79 (m, 2H) 7.08 (d, *J* = 2.0 Hz, 1H), 6.92 (d, *J* = 8.4 Hz, 2H), 6.57 (d, *J* = 8.4 Hz, 2H), 3.91 (s, 3H), 3.57 (s, 3H); **^{13}C NMR** (100 MHz, $CD_3OD/CDCl_3$ = 6/1): δ 165.3, 161.1, 144.6, 142.2, 141.8, 141.7, 139.5, 134.2, 133.8, 133.1, 132.8, 130.43, 130.36, 129.24, 129.19, 128.7, 128.2, 127.1, 125.9, 123.6, 115.2, 100.4, 57.3, 55.6; **HRMS** (ESI$^+$) calcd for $C_{26}H_{21}O_2N_2^+$ 393.1598, found 393.1609; **IR** (KBr): 2955, 1733, 1612, 1567, 1469, 1376, 1209, 1034 and 772 cm^{-1}.

2-methoxy-5-(3'-methoxy-[1,1'-biphenyl]-2-yl)benzo[c]cinnolin-5-ium tetrafluoroborate (3f)

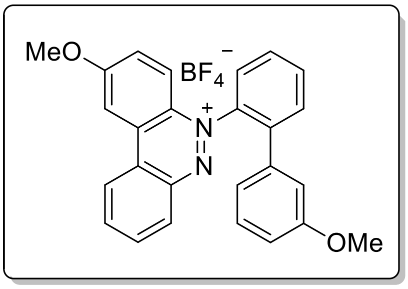

Yellow solid (34.1 mg, 71% yield); R_f = 0.33 (methanol/dichloromethane = 1/9); m.p. 96-98 °C; **^{1}H NMR** (400 MHz, CD_3OD): δ 9.21 (d, *J* = 8.4 Hz, 1H), 8.78 (d, *J* = 8.4 Hz, 1H), 8.52 (t, *J* = 7.6 Hz, 1H), 8.44 (d, *J* = 2.4 Hz, 1H), 8.37 (t, *J* = 7.8 Hz, 1H), 8.03-7.93 (m, 3H), 7.89-7.83 (m, 1H), 7.81 (d, *J* = 7.6 Hz, 1H), 7.70 (dd, *J* = 9.6 Hz, *J* = 2.8 Hz, 1H), 6.90 (t, *J* = 8.0 Hz, 1H), 6.64-6.49 (m, 3H), 4.20 (s, 3H), 3.40 (s, 3H); **^{13}C NMR** (100 MHz, CD_3OD): δ 166.4, 160.9, 144.6, 142.2, 140.5, 139.8, 138.5, 136.4, 135.1, 133.8, 133.5, 133.2, 132.6, 130.8, 130.7, 128.1, 127.9, 127.1, 125.3, 124.6, 121.4, 115.3, 114.6, 103.5, 58.0, 55.5; **HRMS** (ESI$^+$) calcd for $C_{26}H_{21}O_2N_2^+$ 393.1603, found 393.1605; **IR** (KBr): 2921, 1731, 1606, 1571, 1382, 1267, 1056 and 772 cm^{-1}.

3-fluoro-5-(4'-fluoro-[1,1'-biphenyl]-2-yl)benzo[c]cinnolin-5-ium tetrafluoroborate (3g)

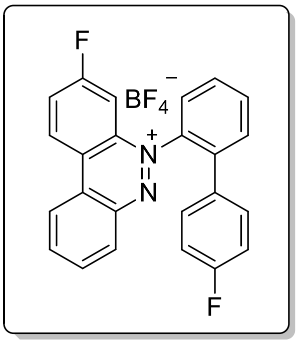

Yellow solid (14.6 mg, 32% yield); R_f = 0.25 (methanol/dichloromethane/ethyl acetate = 2/1/22); m.p. 100-102 °C; **^{1}H NMR** (400 MHz, CD_3OD): δ 9.34 (dd, J = 9.6 Hz, J = 5.2 Hz, 1H), 9.20 (d, J = 8.8 Hz, 1H), 8.85 (d, J = 9.2 Hz, 1H), 8.65 (t, J = 7.8 Hz, 1H), 8.42 (ddd, J = 8.5 Hz, J = 7.2 Hz, J = 1.2 Hz, 1H), 8.19 (ddd, J = 9.5 Hz, J = 7.7 Hz, J = 2.4 Hz, 1H), 8.03-7.98 (m, 1H), 7.98-7.95 (m, 1H),7.92-7.86 (m, 1H), 7.84-7.78 (m, 2H), 7.08-7.02 (m, 2H), 6.80-6.72 (m, 2H); **^{13}C**

NMR (100MHz, CD_3OD): δ 167.0, 165.0, 164.4, 162.5, 144.8, 143.2, 141.6, 141.3, 141.2, 138.7, 135.1, 134.1, 133.9, 133.1, 133.03, 132.99, 131.23, 131.15 (2C), 131.0, 129.2, 129.1, 128.3, 128.1, 127.4, 127.0, 126.8, 124.1, 116.8, 116.6, 107.6, 107.3; **HRMS** (ESI+) calcd for $C_{24}H_{15}F_2N_2^+$ 369.1198, found 369.1209; **IR** (KBr): 2921, 1730, 1607, 1577, 1465, 1405, 1191, 1060 and 771 cm^{-1}.

2-fluoro-5-(3'-fluoro-[1,1'-biphenyl]-2-yl)benzo[c]cinnolin-5-ium tetrafluoroborate (3h)

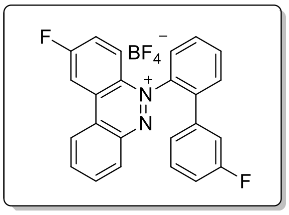

Yellow solid (12.3 mg, 27% yield); R_f = 0.40 (methanol/ethyl acetate = 2/3); m.p. 110-112 °C; **^{1}H NMR** (400 MHz, CD_3OD): δ 9.19 (d, *J* = 8.8 Hz, 1H), 9.02 (dd, *J* = 9.0 Hz, *J* = 2.6 Hz, 1H), 8.91 (d, *J* = 8.4 Hz, 1H), 8.65 (t, *J* = 7.8 Hz, 1H), 8.47 (t, *J* = 7.6 Hz, 1H), 8.29 (dd, *J* = 9.8 Hz, *J* = 4.6 Hz, 1H), 8.07-7.99 (m, 3H), 7.96-7.90 (m, 1H), 7.86 (dd, *J* = 7.6 Hz, *J* = 1.2 Hz, 1H), 7.03-6.96 (m, 1H), 6.92 (dt, *J* = 9.6 Hz, *J* = 2.0 Hz, 1H), 6.82 (td, *J* = 8.2 Hz, *J* = 2.4 Hz, 1H), 6.77 (d, *J* = 7.6 Hz, 1H); **^{13}C NMR** (100MHz, CD_3OD): δ 168.1, 165.5, 165.0, 162.6, 144.6, 142.5, 142.0, 139.34, 139.26, 138.69, 138.68, 137.6, 135.8, 134.2 (2C), 133.2, 133.0, 132.9, 131.8, 131.7, 131.3, 128.3, 128.2, 128.05, 127.99, 127.1, 127.0, 125.9, 125.6, 125.21, 125.18, 124.7, 116.6, 116.4, 110.6, 110.3; **HRMS** (ESI^+) calcd for $C_{24}H_{15}F_2N_2^+$ 369.1198, found 369.1207; **IR** (KBr): 2920, 1731, 1609, 1573, 1472, 1398, 1185, 1055 and 771 cm^{-1}.

8-methyl-5-(4-methyl-[1,1'-biphenyl]-2-yl)benzo[c]cinnolin-5-ium tetrafluoroborate (3i)

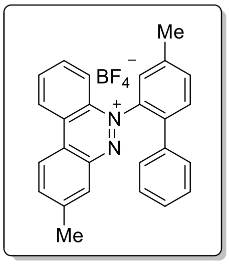

Yellow solid (31.4 mg, 70% yield); R_f = 0.30 (methanol/ethyl acetate = 1/4); m.p. 284-286 °C; **^{1}H NMR** (400 MHz, DMSO-d_6): δ 9.29 (t, *J* = 10.0 Hz, 2H), 8.75 (s, 1H), 8.56 (d, *J* = 8.4 Hz, 1H), 8.33 (t, *J* = 7.4 Hz, 1H), 8.13 (t, *J* = 7.8 Hz, 1H), 8.09-8.02 (m, 1H), 7.91 (s, 1H), 7.84 (d, *J* = 8.0 Hz, 1H), 7.74 (d, *J* = 7.6 Hz, 1H), 7.08-6.91 (m, 5H), 2.78 (s, 3H), 2.56 (s, 3H); **^{13}C NMR** (100 MHz, DMSO-d_6): δ 145.2, 143.5, 143.2, 140.1, 139.7, 138.1, 135.5, 135.2, 134.9, 134.3, 133.2, 131.2, 130.7, 128.5, 128.3, 128.1, 127.8, 127.5, 124.8, 124.1, 123.0, 121.5, 21.2, 20.6; **HRMS** (ESI^+) calcd for $C_{26}H_{21}N_2^+$ 361.1705, found 361.1705; **IR** (KBr): 2923, 1729, 1617, 1571, 1397, 1052 and 771 cm^{-1}.

8-fluoro-5-(4-fluoro-[1,1'-biphenyl]-2-yl)benzo[c]cinnolin-5-ium tetrafluoroborate (3j)

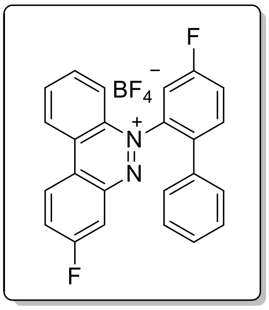

Yellow solid (22.4 mg, 49% yield); R_f = 0.30 (methanol/dichloromethane = 1/9); m.p. 266-268 °C; **^{1}H NMR** (400 MHz, $CDCl_3$:CD_3OD=5:1): δ 9.11 (dd, *J* = 10.0 Hz, *J* = 4.8 Hz, 1H), 8.99 (d, *J* = 8.8 Hz, 1H), 8.29-8.22 (m, 2H), 8.20 (t, *J* = 8.0 Hz, 1H), 8.00 (t, *J* = 7.6 Hz, 1H), 7.90 (d, *J* = 9.2 Hz, 1H), 7.71-7.62 (m, 2H), 7.58 (td, *J* = 8.2 Hz, *J* = 2.4 Hz, 1H), 6.96-6.90 (m, 1H), 6.88 (t, *J* = 7.4 Hz, 2H), 6.79 (d, *J* = 6.8 Hz, 2H); **^{13}C NMR** (100 MHz, $CDCl_3$:CD_3OD = 5:1): δ 165.6, 163.2, 163.0, 160.7, 144.5, 144.3, 140.5, 140.4, 138.3 (2C), 136.0, 134.79, 134.75, 134.6, 134.3, 133.2, 133.1, 131.9, 131.6, 129.0, 128.7, 128.6, 127.6, 126.3, 126.2, 124.43, 124.36, 120.8, 120.2, 120.0, 115.4, 115.1, 114.9, 114.7; **HRMS** (ESI$^+$) calcd for $C_{24}H_{15}F_2N_2^+$ 369.1203, found 369.1206; **IR** (KBr): 2918, 1733, 1621, 1573, 1400, 1175, 1053 and 771 cm^{-1}.

5-(4,4'-dimethyl-[1,1'-biphenyl]-2-yl)-3,8-dimethylbenzo[c]cinnolin-5-ium tetrafluoroborate (3k)

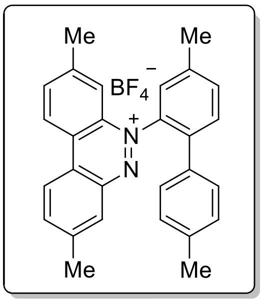

Yellow solid (21.0 mg, 44% yield); R_f = 0.25 (methanol/dichloromethane = 1/19); m.p. 132-134 °C; **^{1}H NMR** (400 MHz, $CDCl_3$): δ 9.04 (dd, *J* = 9.0 Hz, *J* = 5.0 Hz, 2H), 8.40 (s, 1H), 8.33 (d, *J* = 8.4 Hz, 1H), 8.03 (d, *J* = 8.8 Hz, 1H),7.69 (d, *J* = 8.0 Hz, 1H),7.65 (s, 1H), 7.58 (d, *J* = 10.4 Hz, 2H), 6.80-6.69 (q, *J* = 8.2 Hz, 4H), 2.76 (s, 3H), 2.60 (s, 3H), 2.57 (s, 3H), 2.07 (s, 3H) ; **^{13}C NMR** (100 MHz, $CDCl_3$): δ146.1, 144.8, 143.55, 143.52, 140.2, 140.1, 138.40, 138.36, 137.6, 135.6, 133.3, 132.7, 131.3, 130.2, 129.4, 127.5, 127.4, 127.1, 125.3, 124.5, 123.3, 119.4, 22.5, 21.9, 21.0, 20.8; **HRMS** (ESI$^+$) calcd for $C_{28}H_{25}N_2^+$ 389.2012, found 389.2026; **IR** (KBr): 2923, 1731, 1618, 1565, 1404, 1057 and 817 cm^{-1}.

3-fluoro-5-(4'-fluoro-4-methyl-[1,1'-biphenyl]-2-yl)-8-methylbenzo[c]cinnolin-5-ium tetrafluoroborate (3l)

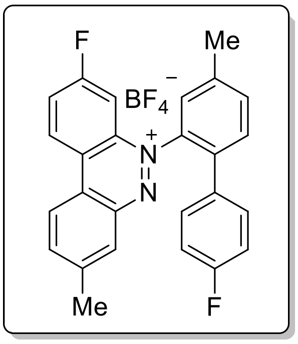

Yellow solid (14.5 mg, 30% yield); R_f = 0.30 (methanol/dichloromethane = 1/9); m.p. 114-116 °C; **^{1}H NMR** (400 MHz, $CDCl_3$): δ 9.11 (dd, *J* = 9.6 Hz, *J* = 5.2 Hz, 1H), 8.92 (d, *J* = 8.8 Hz, 1H), 8.42 (s, 1H), 8.27 (d, *J* = 9.2 Hz, 1H), 7.88-7.81 (m, 2H), 7.69 (d, *J* = 8.0 Hz, 1H), 7.56 (d, *J* = 8.0 Hz, 1H), 7.44 (dd, *J* = 8.6 Hz, *J* = 2.2 Hz, 1H), 6.92-6.85 (dd, *J* = 8.4 Hz, *J* = 5.2 Hz, 2H), 6.68 (t, *J* = 8.4 Hz, 2H), 2.75 (s, 3H), 2.60 (s, 3H); **^{13}C NMR** (100 MHz, $CDCl_3$): δ 165.4, 163.6,

162.8, 161.1, 145.2, 144.2, 143.9, 141.0, 140.0, 139.7, 139.6, 134.3, 133.4, 131.65, 131.61, 131.2, 130.6, 129.7, 129.6, 128.6, 128.5, 128.0, 126.2, 125.8, 125.6, 125.5, 123.2, 116.1, 115.94, 115.87, 105.8, 105.5, 21.9, 21.0; **HRMS** (ESI$^+$) calcd for $C_{26}H_{19}F_2N_2^+$ 397.1566, found 397.1568; **IR** (KBr): 2923, 1731, 1621, 1577,1478, 1408, 1053 and 771 cm^{-1}.

4-(2-(thiophen-2-yl)phenyl)-4l4-thieno[3,2-c]cinnoline tetrafluoroborate (3m)

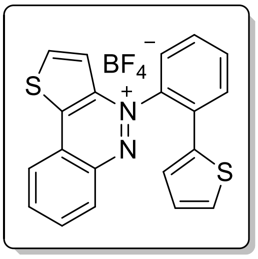

Yellow solid (15.1 mg, 35% yield); R_f = 0.25 (methanol/ethyl acetate = 1/9); m.p. 220-222 °C; **^{1}H NMR** (400 MHz, DMSO-d$_6$): δ 8.91 (d, *J* = 8.4 Hz, 1H), 8.85 (d, *J* = 8.8 Hz, 1H), 8.74 (d, *J* = 5.6 Hz, 1H), 8.56 (t, *J* = 7.8 Hz, 1H), 8.40 (t, *J* = 7.8 Hz, 1H), 8.03 (t, *J* = 7.8 Hz, 2H), 7.97 (t, *J* = 7.6 Hz, 1H), 7.85 (t, *J* = 7.6 Hz, 1H), 7.64 (d, *J* = 6.0 Hz, 1H), 7.36 (d, *J* = 5.2 Hz, 1H), 6.88 (d, *J* = 2.8 Hz, 1H), 6.84 (t, *J* = 4.4 Hz, 1H); **^{13}C NMR** (100 MHz, DMSO-d$_6$): δ 147.4, 144.2, 142.8, 141.1, 140.2, 139.9, 135.7, 134.8, 133.0, 131.2 (2C), 130.0, 129.8, 128.9, 128.2 (2C), 127.6, 126.9, 124.0, 118.6; **HRMS** (ESI$^+$) calcd for $C_{20}H_{13}N_2S_2^+$ 345.0520, found 345.0517; **IR** (KBr): 2922, 1731, 1611, 1550, 1420, 1263, 1055 and 770 cm^{-1}.

6-(2-(naphthalen-1-yl)phenyl)dibenzo[c,f]cinnolin-6-ium tetrafluoroborate (3n)

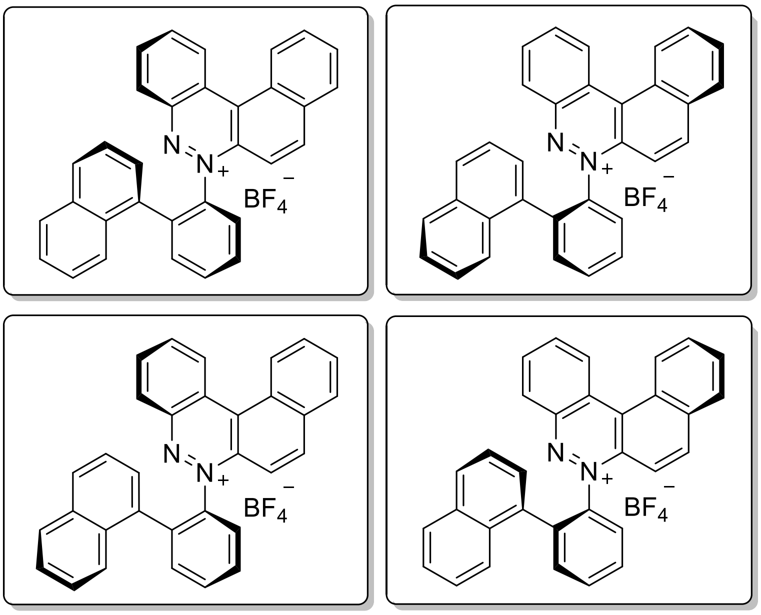

Orange solid (10.4 mg, 20% yield); R_f = 0.40 (methanol/DCM = 1/9); m.p. 150-152 °C; **^{1}H NMR** (400 MHz, CDCl$_3$): δ 9.19 (d, *J* = 9.2 Hz, 1H), 9.12 (d, *J* = 8.8 Hz, 1H), 9.06-8.98 (m, 2H), 8.71 (d, *J* = 8.8 Hz, 1H), 8.45 (t, *J* = 8.0 Hz, 1H), 8.35 (d, *J* = 9.6 Hz, 1H), 8.31 (t, *J* = 8.0 Hz, 1H), 8.24 (t, *J* = 7.8 Hz, 1H), 8.20-8.10 (m, 4H), 8.03-7.85 (m, 11H), 7.84-7.73 (m, 5H), 7.65 (d, *J* = 8.4 Hz, 1H), 7.54-7.48 (m, 2H), 7.43-7.34 (m, 4H), 7.34-7.27 (m, 2H), 7.14-7.04 (m, 2H), 7.00-6.89 (m, 2H); **^{13}C NMR** (100 MHz, CDCl$_3$): δ 144.7, 144.6, 141.9, 141.8, 140.4, 140.1, 139.34, 139.31, 137.6, 136.91, 136.86, 136.3, 134.0, 133.8, 133.6, 133.3, 133.2, 132.94,

132.88, 132.7, 132.6, 132.3, 132.2, 132.04, 132.00, 131.8, 131.7, 131.2, 131.1, 130.8, 130.7, 130.6, 130.2, 130.1 (2C), 129.94 (2C), 129.86, 129.5, 129.3, 129.0 (2C), 128.4, 127.94, 127.86, 127.7, 127.2, 126.8, 126.7, 126.50, 126.47, 126.4, 126.3, 126.24, 126.20, 126.1 (2C), 125.9 (2C), 124.6, 124.5, 124.3, 116.3, 116.2; **HRMS** (ESI^+) calcd for $C_{32}H_{21}N_2^+$ 433.1700, found 433.1705; **IR** (KBr): 2922, 1729, 1606, 1563, 1396, 1052 and 779 cm^{-1}.

6-(2-(naphthalen-2-yl)phenyl)dibenzo[c,g]cinnolin-6-ium tetrafluoroborate (3o)

Orange solid (11.4 mg, 22% yield); R_f = 0.20 (methanol/dichloromethane = 1/19); m.p. 238-240 °C; **^{1}H NMR** (400 MHz, $CDCl_3$): δ 8.89 (d, *J* = 8.8 Hz, 1H), 8.73 (d, *J* = 8.4 Hz, 1H), 8.53 (d, *J* = 7.6 Hz, 1H), 8.42-8.32 (m, 2H), 8.26 (t, *J* = 7.8 Hz, 1H), 8.00 (t, *J* = 7.6 Hz, 1H), 7.93 (t, *J* = 7.6 Hz, 1H), 7.87 (d, *J* = 9.2 Hz, 1H), 7.81-7.74 (m, 2H), 7.68 (d, *J* = 8.4 Hz, 1H), 7.59 (d, *J* = 6.8 Hz, 1H), 7.56-7.49 (m, 1H), 7.48 (d, *J* = 8.0 Hz, 1H), 7.33 (t, *J* = 7.8 Hz, 1H), 7.28 (d, *J* = 7.2 Hz, 1H), 7.06 (t, *J* = 8.4 Hz, 2H), 6.51 (s, 1H), 6.04 (d, *J* = 8.4 Hz, 1H); **^{13}C NMR** (100 MHz, $CDCl_3$): δ 145.9, 144.5, 139.8, 138.6, 137.7, 136.8, 134.7, 134.4, 132.6, 132.5, 132.3, 132.2, 132.0, 131.6, 131.2, 131.1, 130.9, 129.8, 129.0, 128.0, 127.41, 127.37, 127.3, 126.92, 126.89, 126.8, 126.5, 125.8, 123.63, 123.61, 122.6, 118.7; **HRMS** (ESI^+) calcd for $C_{32}H_{21}N_2^+$ 433.1705, found 433.1702; **IR** (KBr): 2923, 1730, 1604, 1562, 1500, 1416, 1349, 1052 and 771 cm^{-1}.

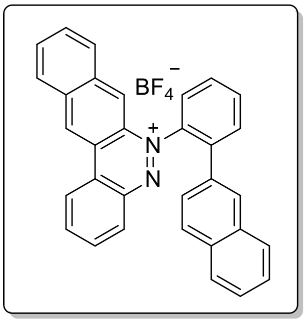

5-(4'-fluoro-[1,1'-biphenyl]-2-yl)-3-methoxybenzo[c]cinnolin-5-ium tetrafluoroborate (3p)

Red solid (32.3 mg, 69% yield); R_f = 0.25 (methanol/dichloromethane = 1/19); m.p. 98-100 °C; **^{1}H NMR** (400 MHz, CD_3OD): δ 9.16 (d, *J* = 9.2 Hz, 1H), 9.09 (d, *J* = 8.8 Hz, 1H), 8.72 (d, *J* = 8.8 Hz, 1H), 8.52 (ddd, *J* = 8.5 Hz, *J* = 7.2 Hz, *J* = 1.2 Hz, 1H), 8.30 (ddd, *J* = 8.5 Hz, *J* = 7.2 Hz, *J* = 1.2 Hz, 1H), 8.03-7.93 (m, 3H), 7.93-7.87 (m, 1H), 7.86 (dd, *J* = 7.8 Hz, *J* = 1.4 Hz, 1H), 7.13 (d, *J* = 2.4 Hz, 1H), 7.11-7.03 (m, 2H), 6.83-6.74 (m, 2H), 3.94 (s, 3H); **^{13}C NMR** (100 MHz, CD_3OD): δ 165.6, 165.2, 162.8, 144.7, 142.5, 141.95, 141.91, 138.9, 134.3, 134.0, 133.6, 133.5, 133.2, 133.0, 131.5, 131.4, 131.1, 129.3, 128.9, 128.3, 127.3, 126.1, 123.7, 116.9, 116.7, 100.4, 57.4; **HRMS** (ESI^+) calcd for $C_{25}H_{18}OFN_2^+$ 381.1398, found 381.1412; **IR** (KBr): 2920, 1729, 1647, 1469, 1413, 1276,

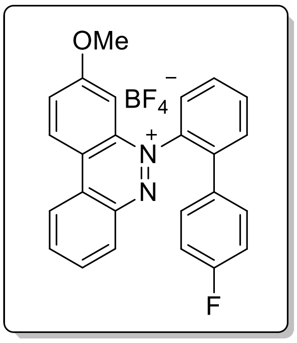

1209, 1060 and 771 cm^{-1}.

5-phenylbenzo[c]cinnolin-5-ium tetrafluoroborate (3r)

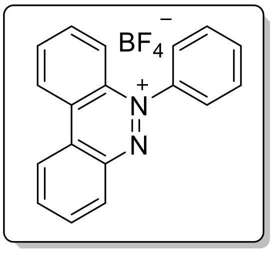

Yellow solid (7.9 mg, 23% yield); R_f = 0.35 (methanol/dichloromethane = 1/9); m.p. 218-220 °C; **^{1}H NMR** (400 MHz, CD_3OD): δ 9.39 (d, *J* = 8.4 Hz, 1H), 9.32 (d, *J* = 8.8 Hz, 1H), 8.90 (d, *J* = 8.4 Hz, 1H), 8.64 (t, *J* = 7.6 Hz, 1H), 8.46–8.41 (m, 2H), 8.28–8.23 (m, 2H), 7.95–7.85 (m, 5H). **^{13}C NMR** (100 MHz, CD_3OD): δ 143.2, 140.4, 138.8, 135.0, 133.8, 133.4, 132.8, 132.1, 130.0, 129.6, 127.2, 126.1, 124.1, 122.7, 121.7; **HRMS** (ESI$^+$) calcd for $C_{18}H_{13}N_2^+$ 257.1079, found 257.1081; **IR** (KBr): 3341, 2918, 1736, 1466, 1261, 1180, 1082 and 799 cm^{-1}.

3-methyl-5-phenylbenzo[c]cinnolin-5-ium tetrafluoroborate (3s)

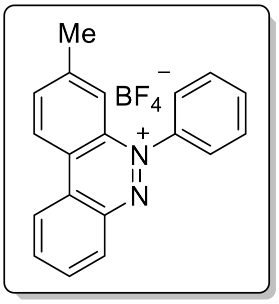

Green solid; (9.7 mg, 27% yield); R_f = 0.3 (methanol/EA = 1/9); m.p. 181-184 °C; **^{1}H NMR** (400 MHz, CD_3OD): δ 9.30–9.26 (m, 2H), 8.84 (d, *J* = 8.8 Hz, 1H), 8.60 (t, *J* = 7.8 Hz, 1H), 8.40–8.35 (m, 1H), 8.33–8.30 (m, 1H), 7.99 (s, 1H), 7.94–7.85 (m, 5H), 2.70 (s, 3H); **^{13}C NMR** (100 MHz, CD_3OD): δ 147.9, 145.1, 144.6, 141.6, 140.5, 138.5, 134.4, 133.8, 133.4, 131.4, 129.3, 128.7, 127.5, 125.2, 124.0, 121.4, 22.4; **HRMS** (ESI$^+$) calcd for $C_{19}H_{15}N_2^+$ 271.1235, found 271.1237; **IR** (KBr): 3306, 2955, 2922, 2851, 1732, 1609, 1568, 1489, 1401, 1377, 1361, 1057 cm^{-1}.

Photophysical properties

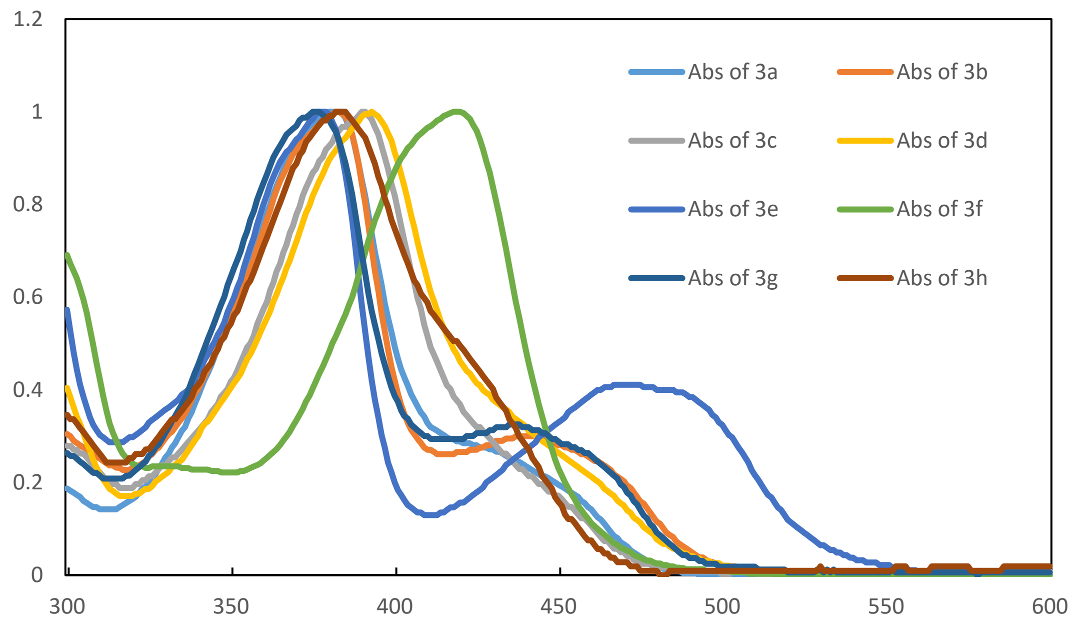

Figure S1. UV–vis spectra Benzo[c]cinnolinium salts of **3a-3h.**

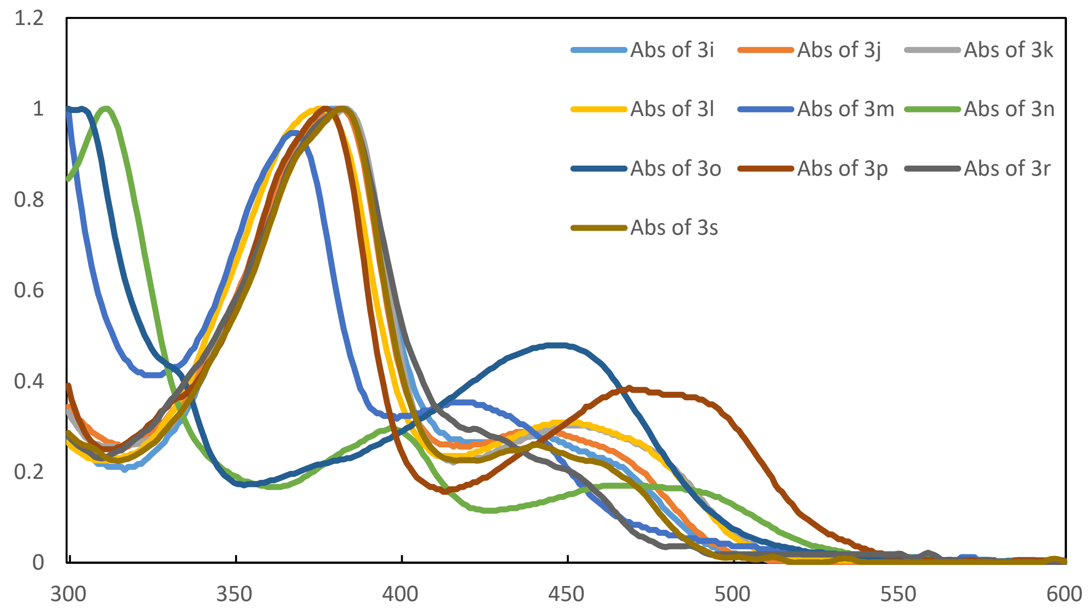

Figure S2. UV–vis absorption spectra of Benzo[c]cinnolinium salts **3i-3p**, **3r** and **3s.**

Table S2. Photophysical properties of benzo[c]cinnolinium salts **3a** and **3p**

compound	λ_{abs} (nm)[a]	ε_{max} ($M^{-1}cm^{-1}$)	λ_{fl} (nm)[b]	Stokes shift (nm)	Φ_{fl}[c]
**3a**	381	22700	-	-	-
**3p**	378, 469	18600, 6700	571, 571	193, 102	0.03, 0.10

[a] Absorption spectra of salts were measured in dichloromethane solutions (1.0×10^{-5} M) at room temperature.

[b] Fluorescence spectra of salts were measured in dichloromethane solutions (1.0×10^{-5} M) at room temperature.

[c] Quantum yields were measured in degassed ethanol solution and the reference standard is anthracene (λ_{ex} = 378 & 469 nm).

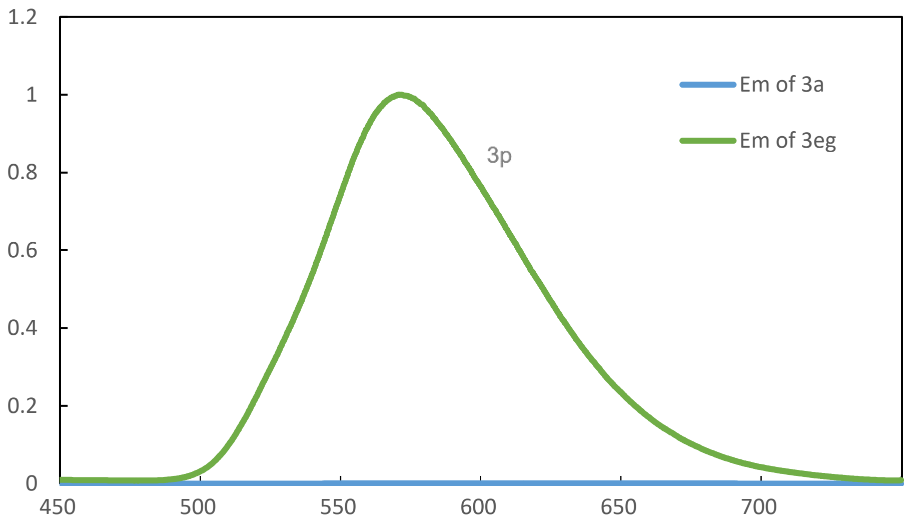

Figure S3. Benzo[c]cinnolinium salts **3a** and **3p** fluorescence emission spectra.

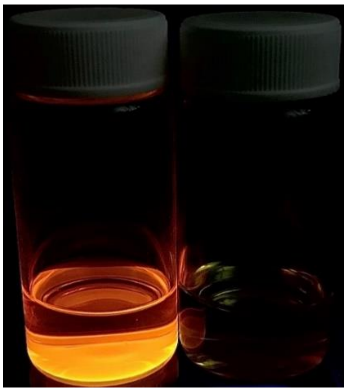

Figure S4. Fluorescence emission of benzo[c]cinnolinium salts **3a** (right) and **3p** (left).

^{1}H and ^{13}C NMR spectra of compounds

^{1}H NMR spectrum of **2b** (400 MHz, $CDCl_3$)

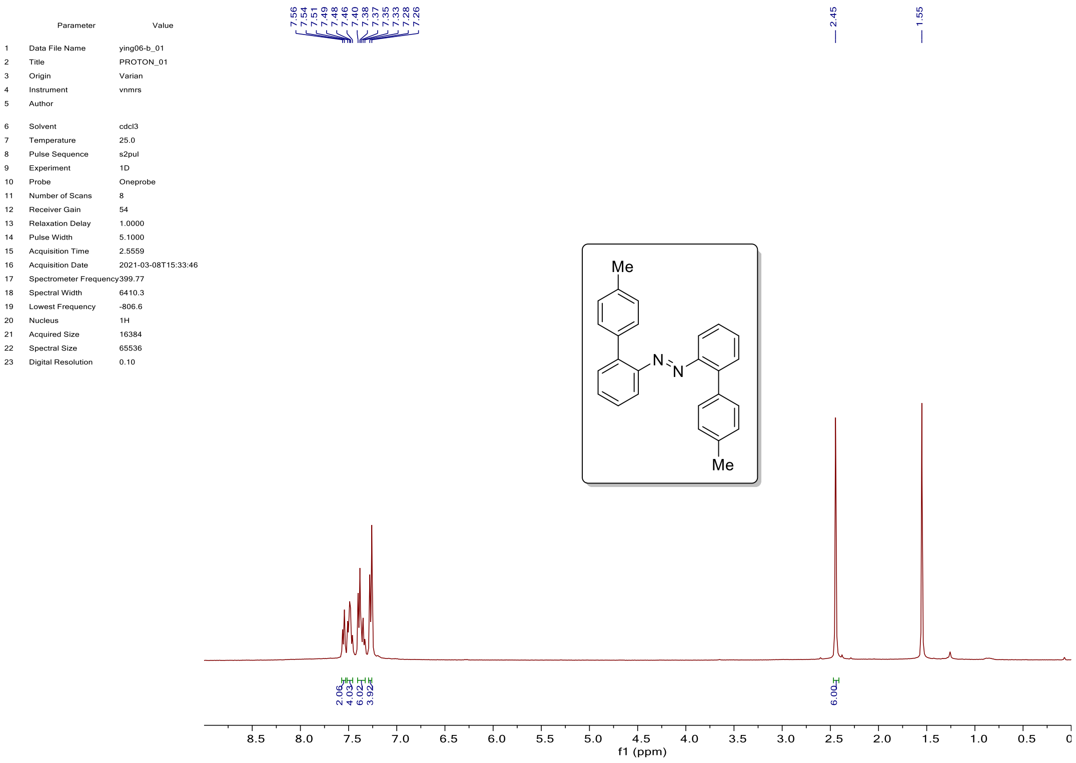

^{13}C NMR spectrum of **2b** (100 MHz, $CDCl_3$)

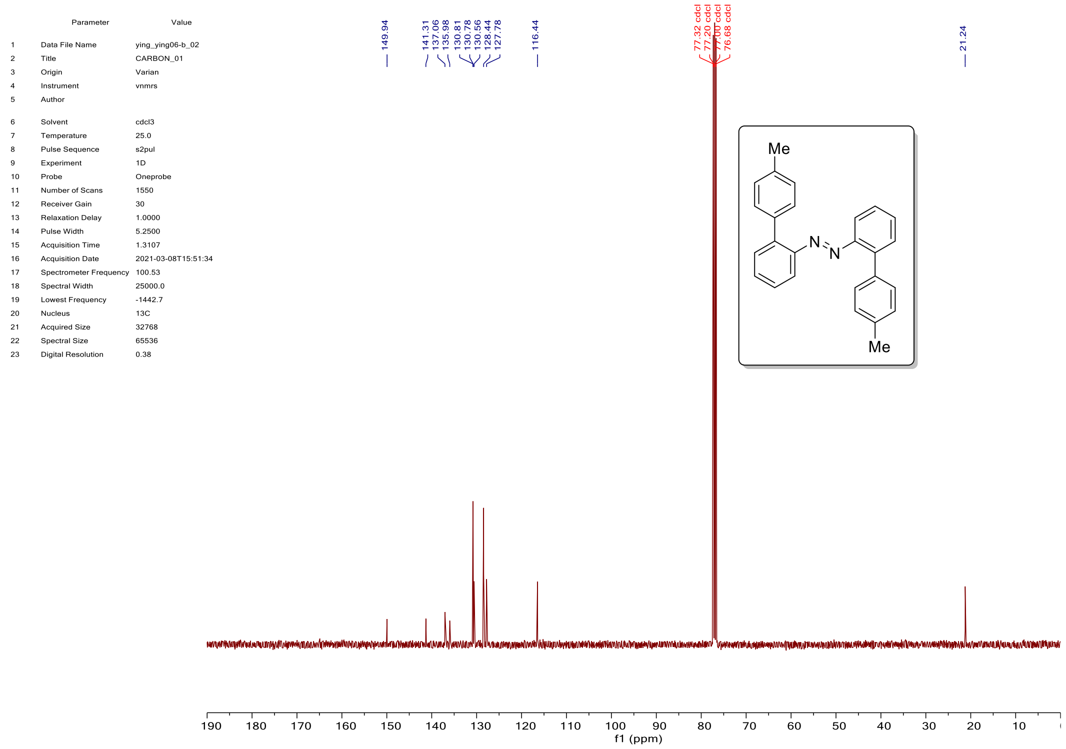

^{1}H NMR spectrum of compound **2d** (400 MHz, $CDCl_3$)

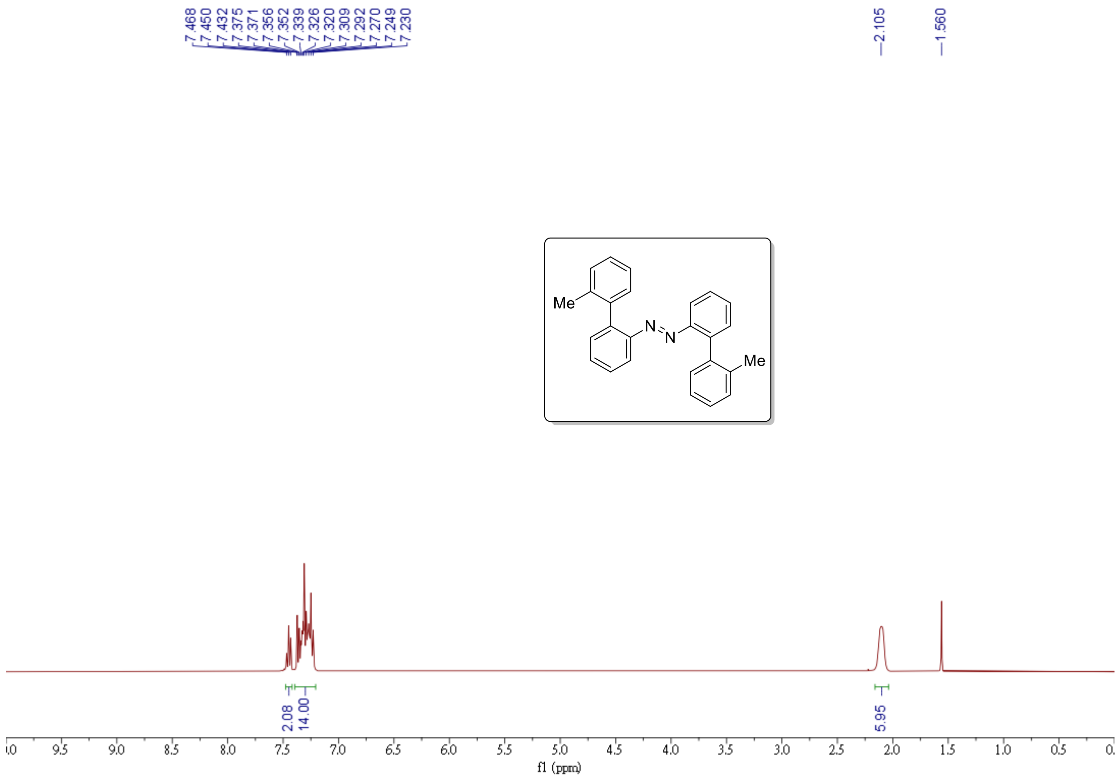

^{13}C NMR spectrum of compound **2d** (100 MHz, $CDCl_3$)

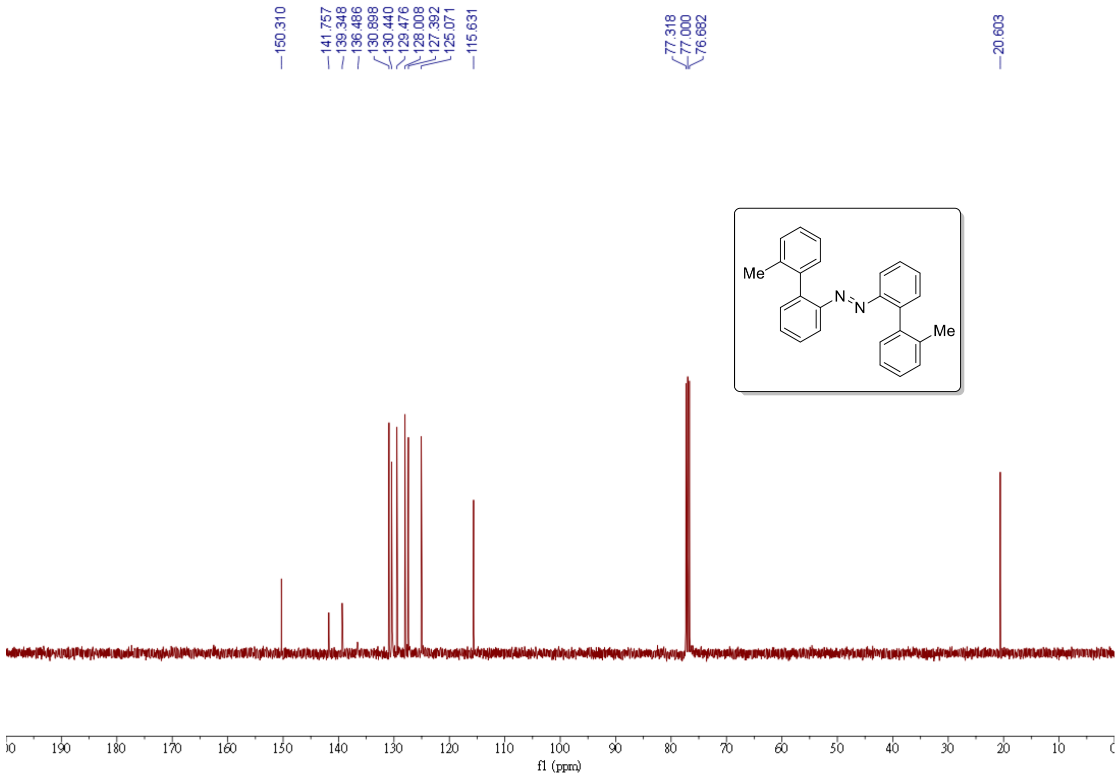

^{1}H NMR spectrum of compound **2e** (400 MHz, $CDCl_3$)

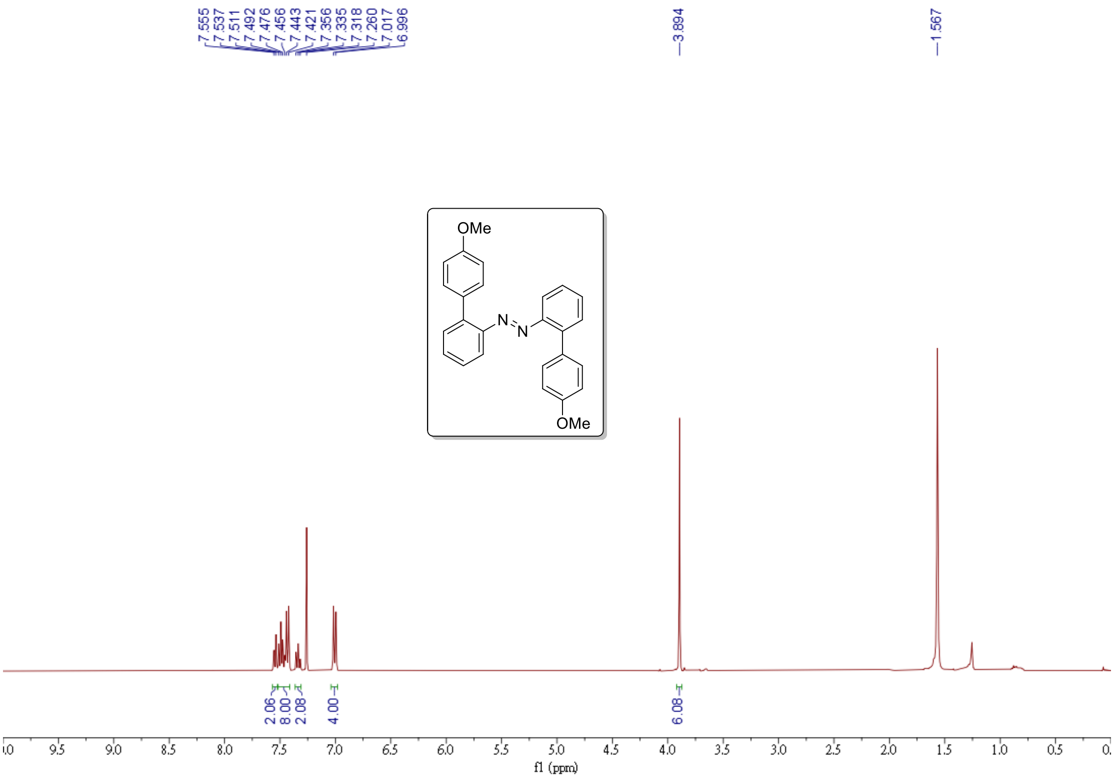

^{13}C NMR spectrum of compound **2e** (100 MHz, $CDCl_3$)

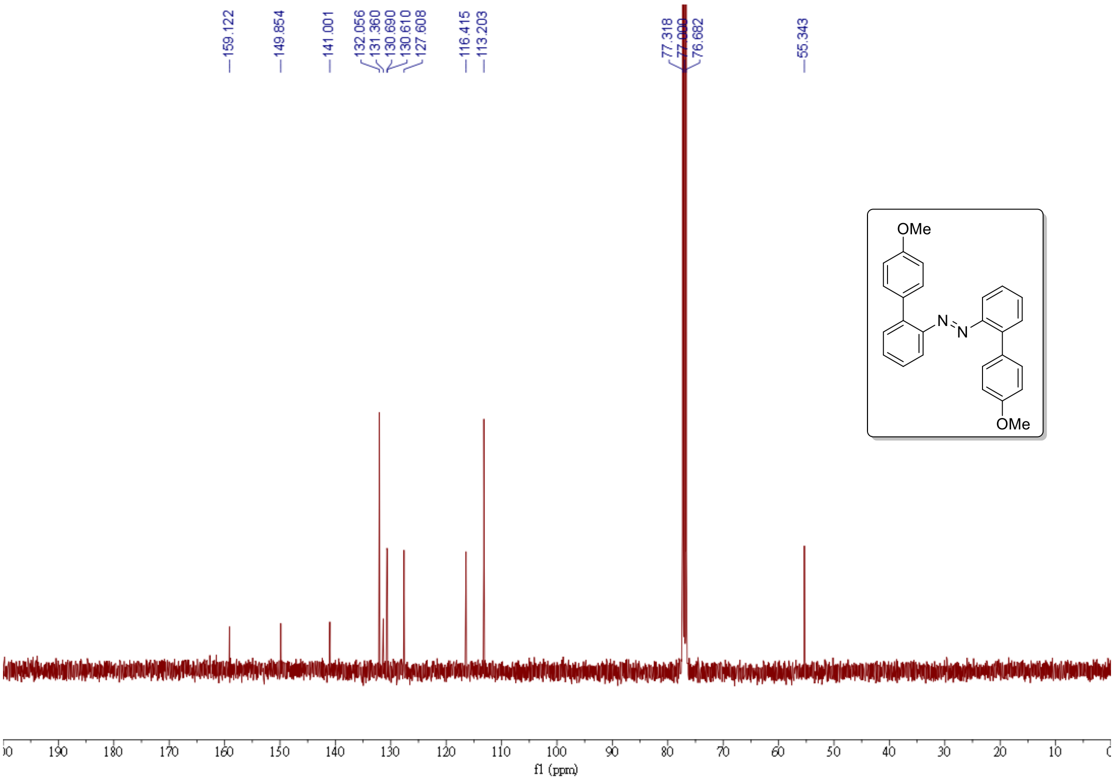

^{1}H NMR spectrum of compound **2f** (400 MHz, $CDCl_3$)

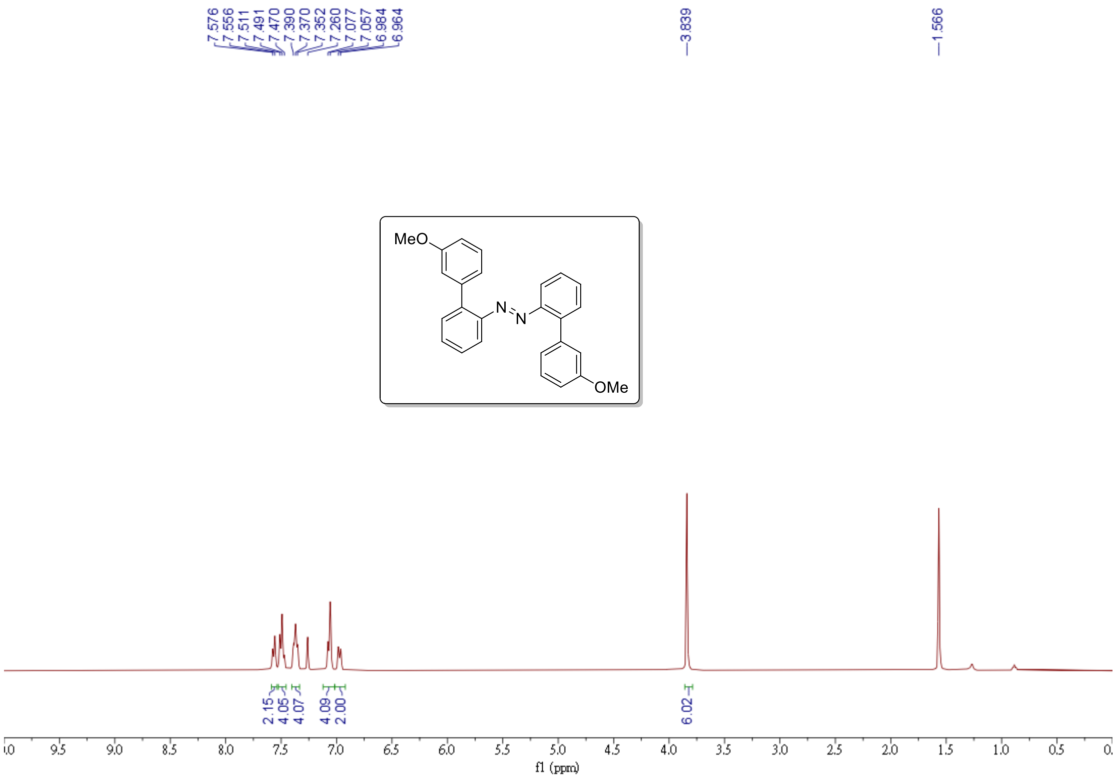

^{13}C NMR spectrum of compound **2f** (100 MHz, $CDCl_3$)

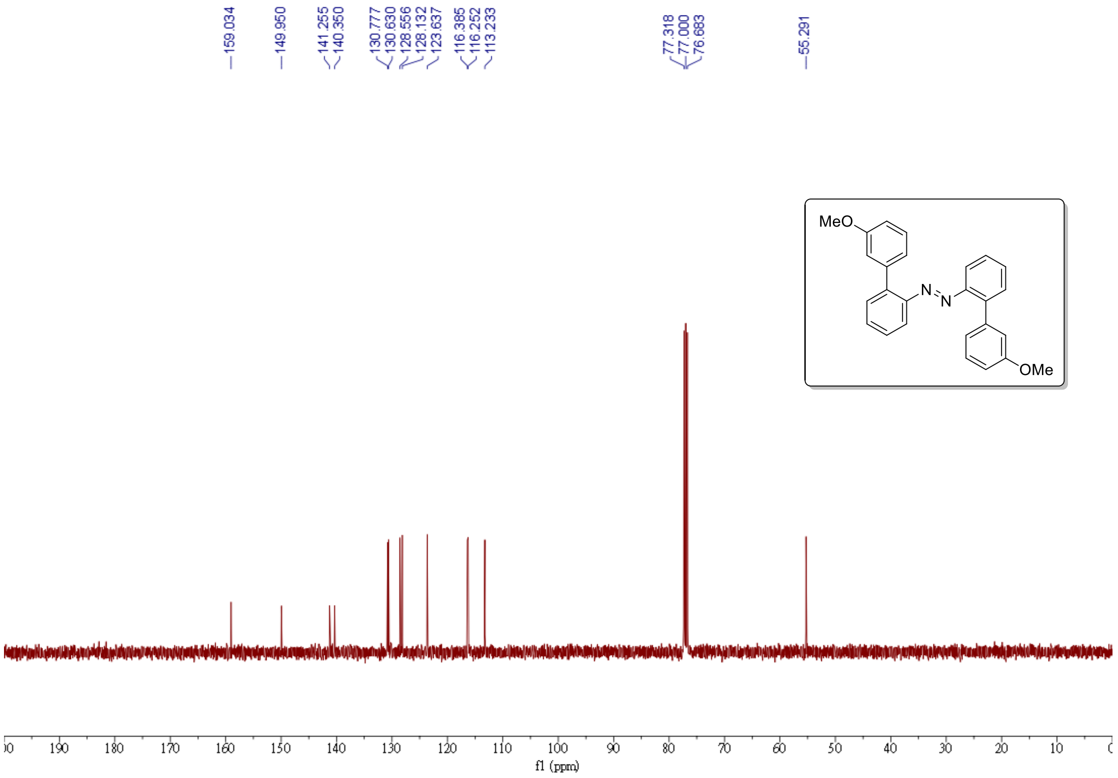

^{1}H NMR spectrum of compound **2g** (400 MHz, $CDCl_3$)

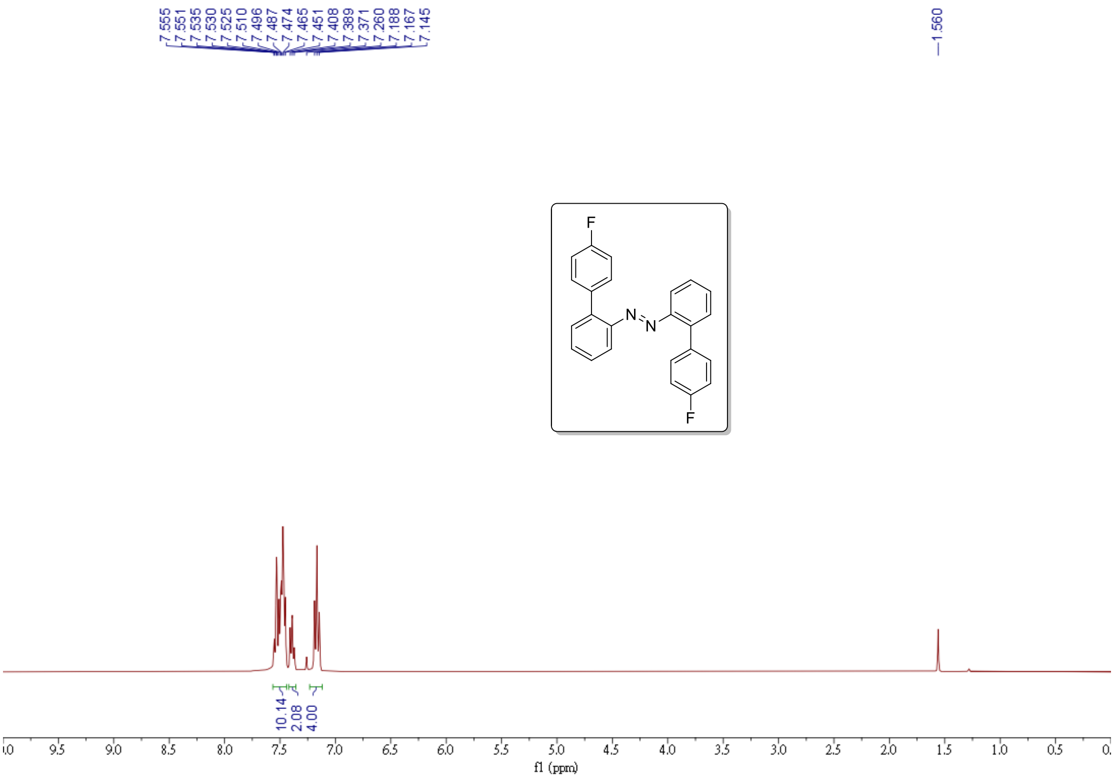

^{13}C NMR spectrum of compound **2g** (100 MHz, $CDCl_3$)

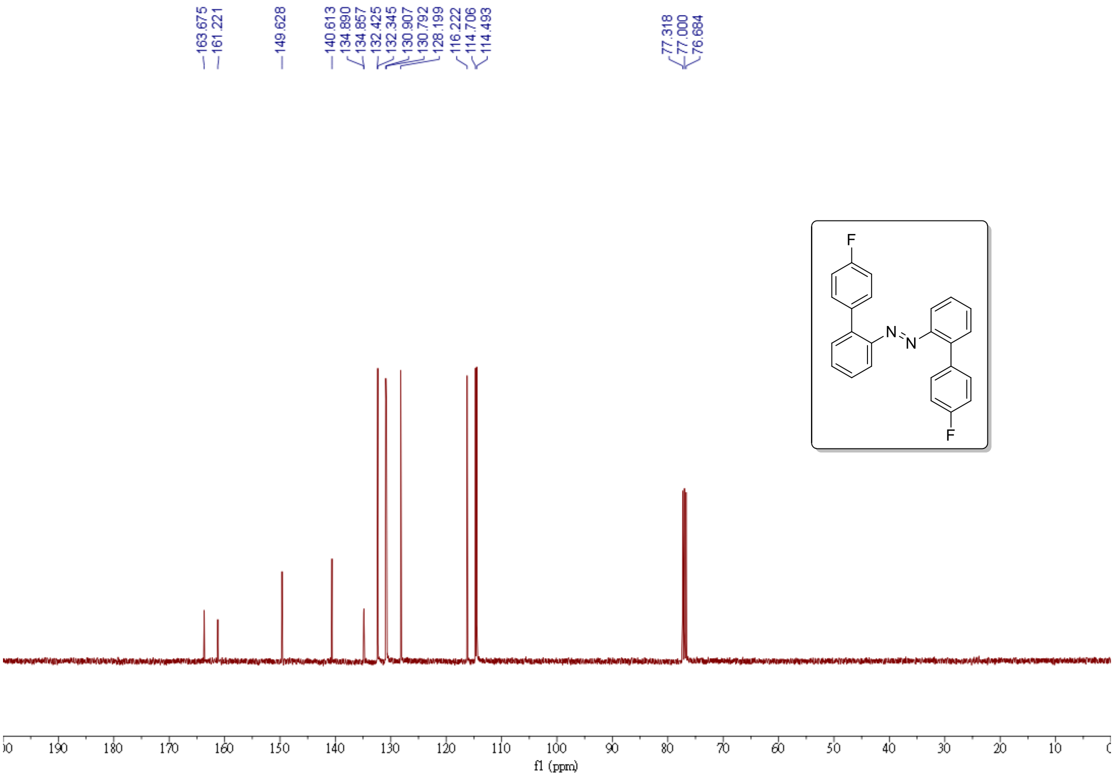

^{1}H NMR spectrum of compound **2j** (400 MHz, $CDCl_3$)

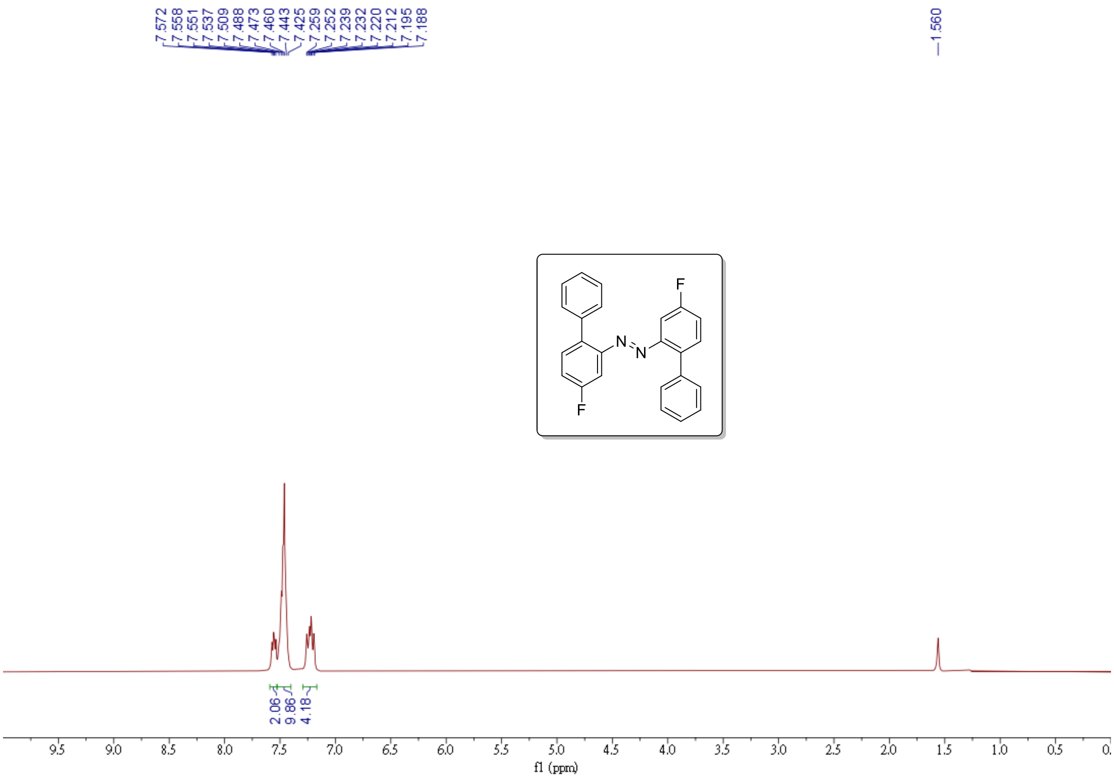

^{13}C NMR spectrum of compound **2j** (100 MHz, $CDCl_3$)

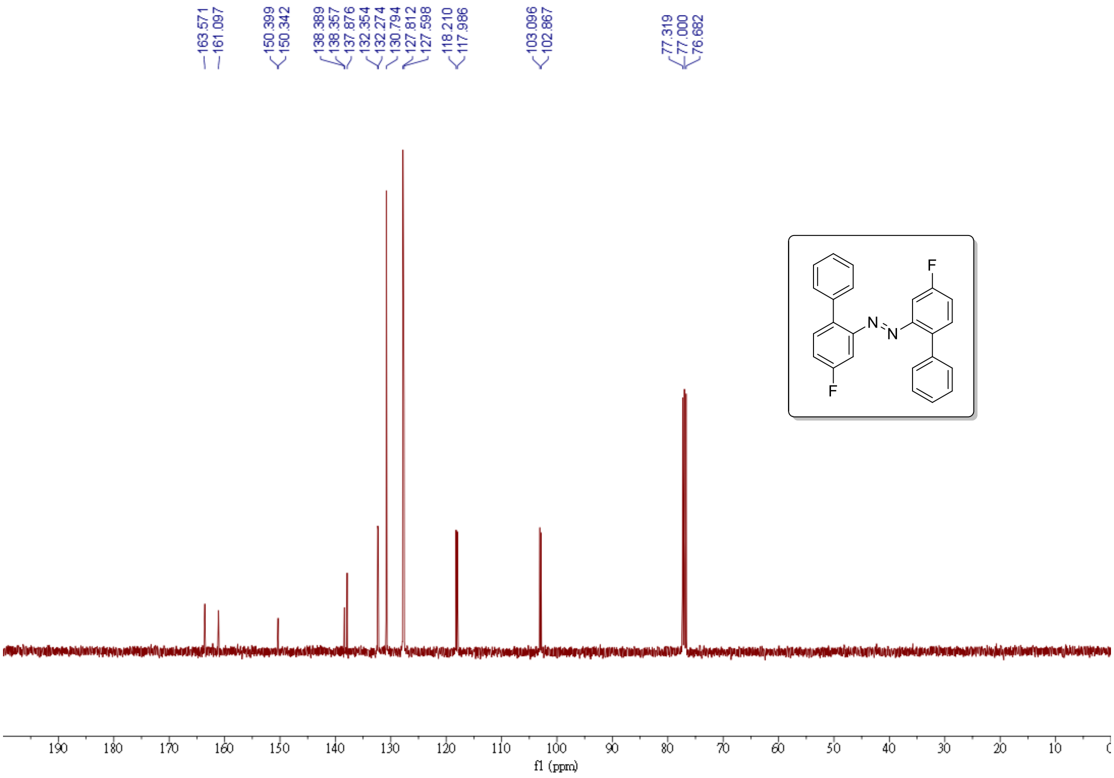

^{1}H NMR spectrum of compound **2k** (400 MHz, $CDCl_3$)

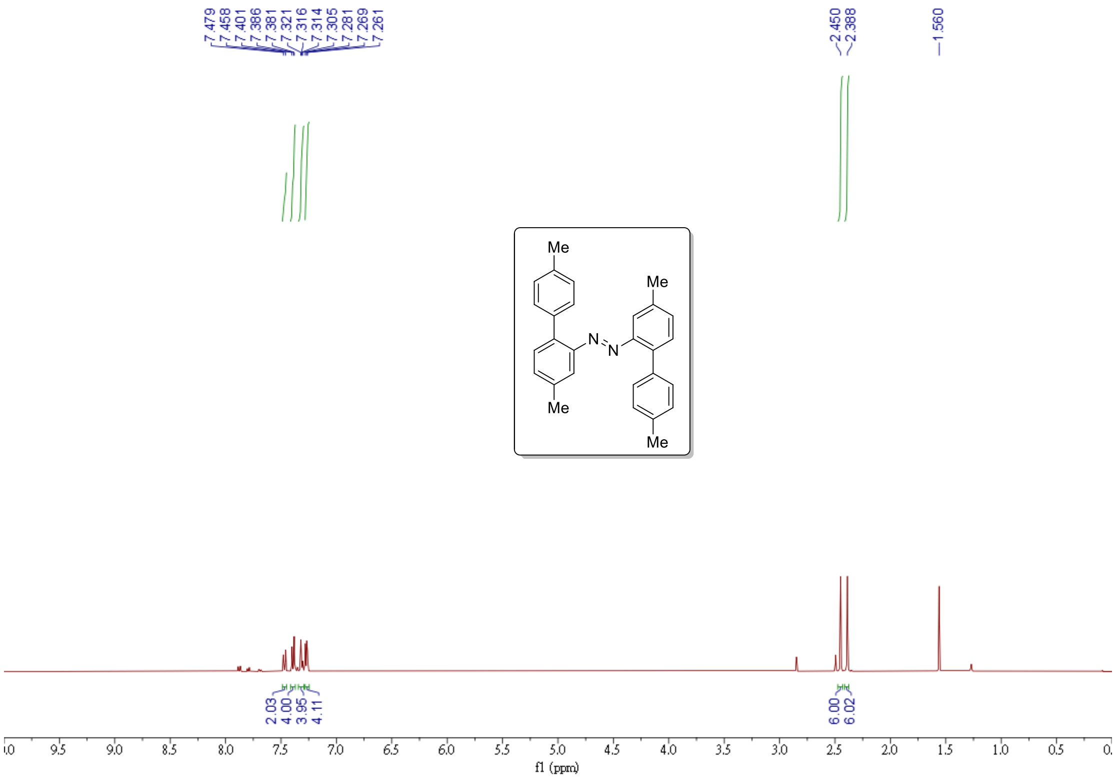

^{13}C NMR spectrum of compound **2k** (100 MHz, $CDCl_3$)

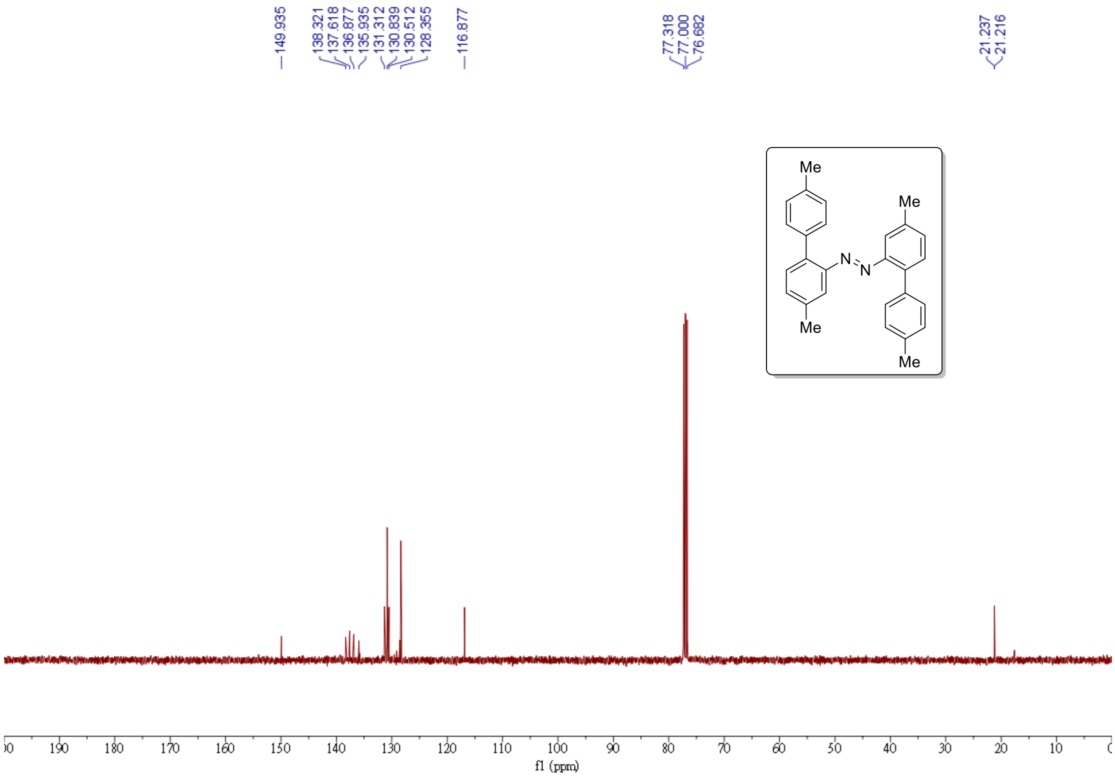

^{1}H NMR spectrum of compound **2l** (400 MHz, $CDCl_3$)

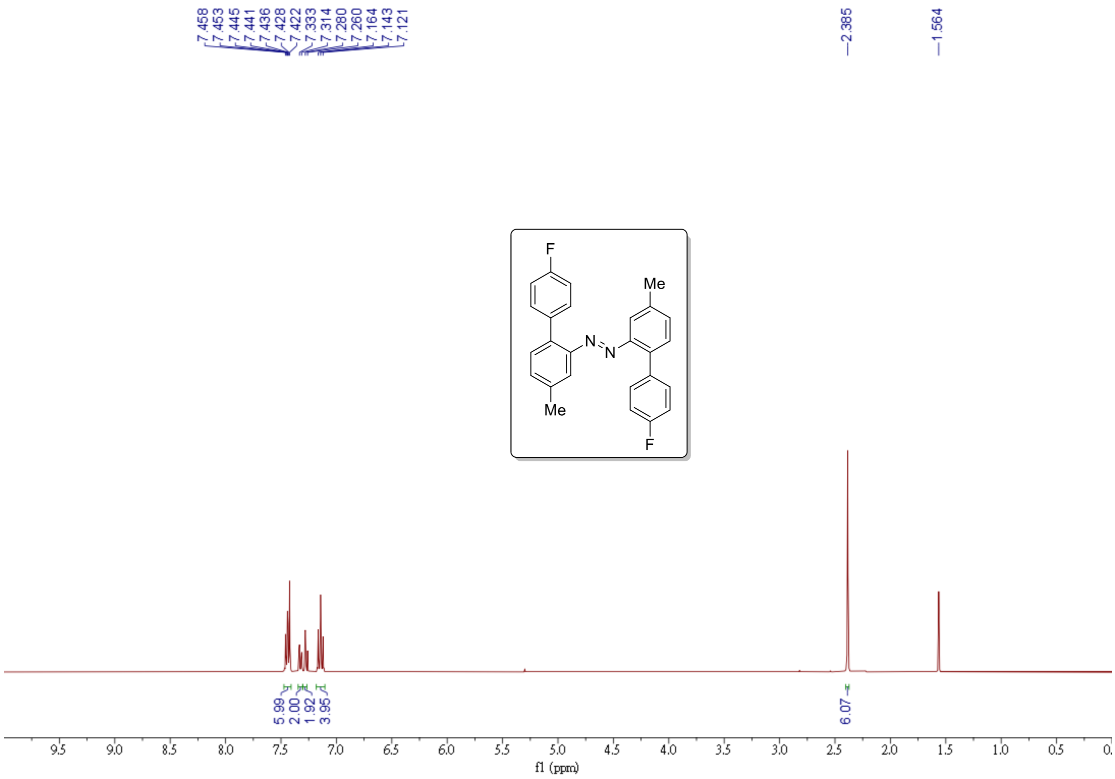

^{13}C NMR spectrum of compound **2l** (100 MHz, $CDCl_3$)

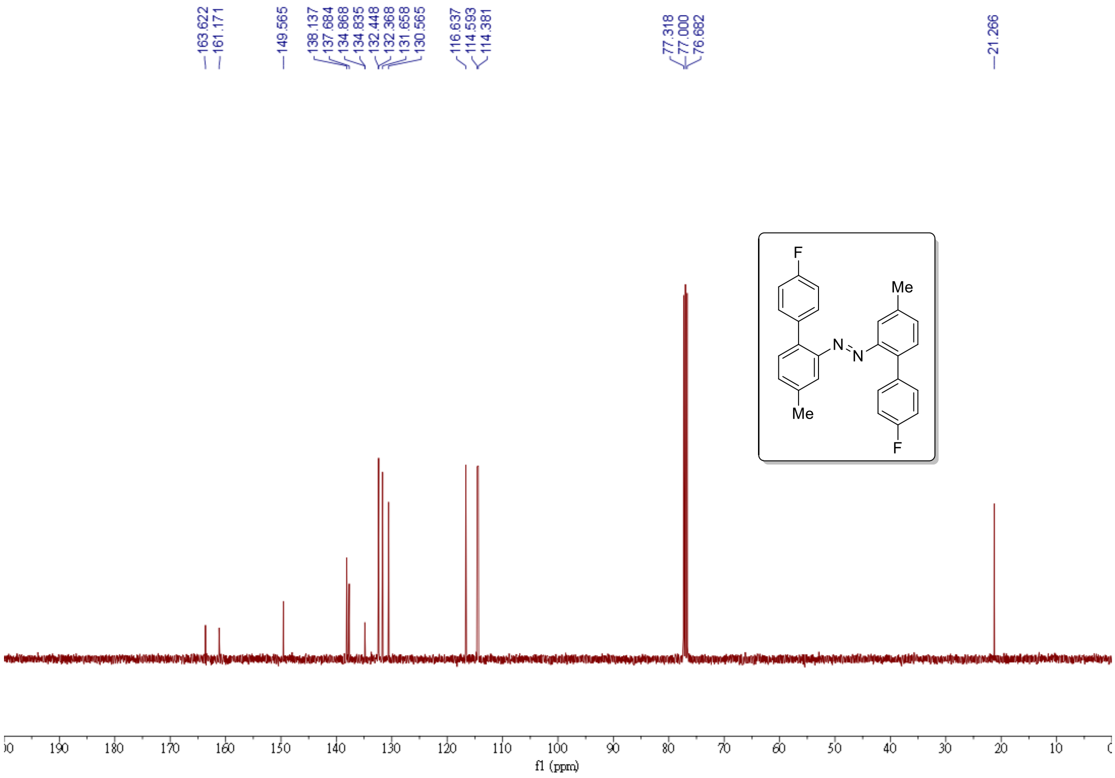

^{1}H NMR spectrum of **2n** (400 MHz, $CDCl_3$)

	Parameter	Value
1	Data File Name	ying07-b_02
2	Title	PROTON_01
3	Origin	Varian
4	Instrument	vnmrs
5	Solvent	cdcl3
6	Temperature	25.0
7	Pulse Sequence	s2pul
8	Experiment	1D
9	Probe	Oneprobe
10	Number of Scans	8
11	Receiver Gain	48
12	Relaxation Delay	1.0000
13	Pulse Width	5.1000
14	Acquisition Time	2.5559
15	Acquisition Date	2021-03-15T20:46:33
16	Spectrometer Frequency	399.77
17	Spectral Width	6410.3
18	Lowest Frequency	-806.6
19	Nucleus	1H
20	Acquired Size	16384
21	Spectral Size	65536
22	Digital Resolution	0.10

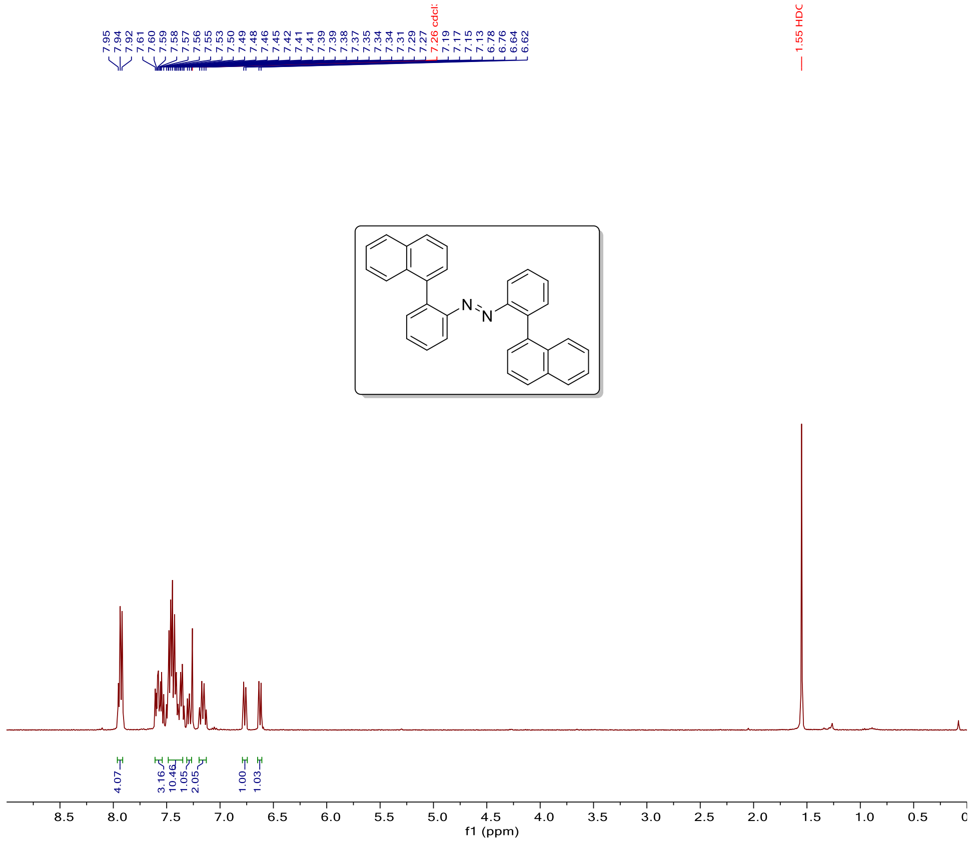

^{13}C NMR spectrum of **2n** (100 MHz, $CDCl_3$)

	Parameter	Value
1	Data File Name	ying07-b_naphyl_02
2	Title	CARBON_02
3	Origin	Varian
4	Instrument	vnmrs
5	Solvent	cdcl3
6	Temperature	25.0
7	Pulse Sequence	s2pul
8	Experiment	1D
9	Probe	Oneprobe
10	Number of Scans	1600
11	Receiver Gain	30
12	Relaxation Delay	1.0000
13	Pulse Width	5.2500
14	Acquisition Time	1.3107
15	Acquisition Date	2021-03-15T21:57:59
16	Spectrometer Frequency	100.53
17	Spectral Width	25000.0
18	Lowest Frequency	-1442.7
19	Nucleus	13C
20	Acquired Size	32768
21	Spectral Size	65536
22	Digital Resolution	0.38

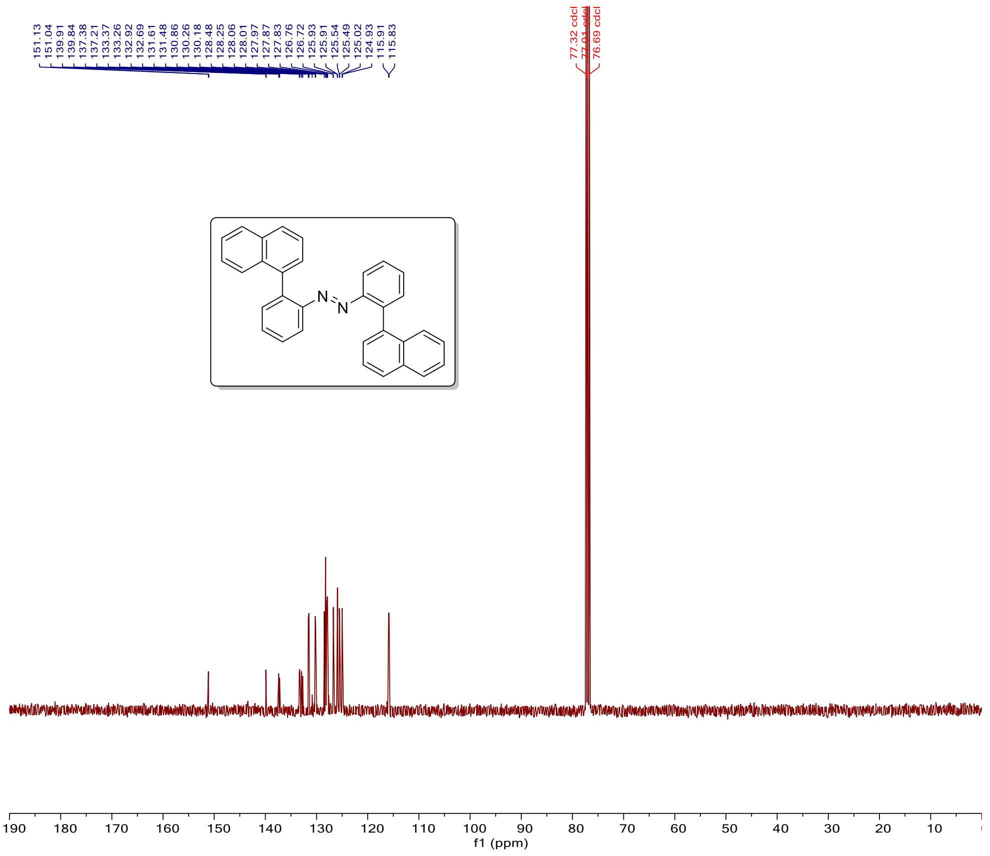

^{1}H NMR spectrum of **2o** (400 MHz, $CDCl_3$)

	Parameter	Value
1	Data File Name	ying11-b_2-naphyl_01
2	Title	PROTON_01
3	Origin	Varian
4	Instrument	vnmrs
5	Solvent	cdcl3
6	Temperature	25.0
7	Pulse Sequence	s2pul
8	Experiment	1D
9	Probe	Oneprobe
10	Number of Scans	8
11	Receiver Gain	54
12	Relaxation Delay	1.0000
13	Pulse Width	5.1000
14	Acquisition Time	2.5559
15	Acquisition Date	2021-05-23T10:27:07
16	Spectrometer Frequency	399.77
17	Spectral Width	6410.3
18	Lowest Frequency	-806.6
19	Nucleus	1H
20	Acquired Size	16384
21	Spectral Size	65536
22	Digital Resolution	0.10

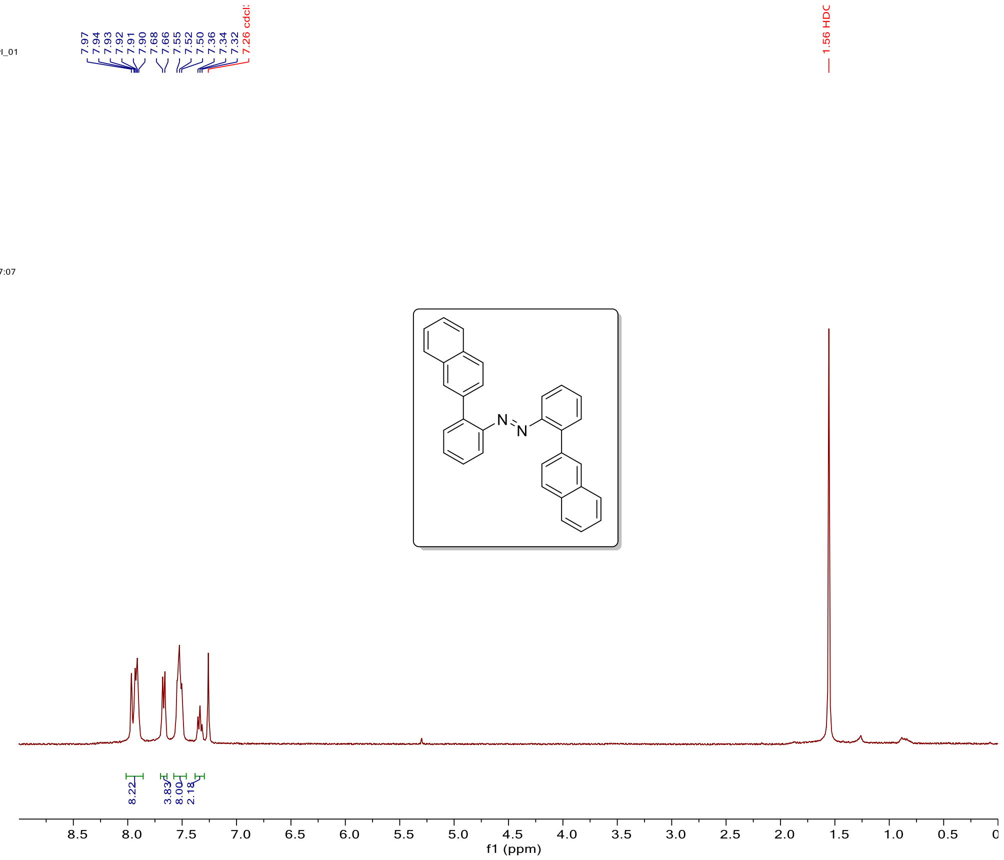

^{13}C NMR spectrum of **2o** (100 MHz, $CDCl_3$)

	Parameter	Value
1	Data File Name	ying11-b_2-nathyl_01
2	Title	CARBON_01
3	Origin	Varian
4	Instrument	vnmrs
5	Solvent	cdcl3
6	Temperature	25.0
7	Pulse Sequence	s2pul
8	Experiment	1D
9	Probe	Oneprobe
10	Number of Scans	4800
11	Receiver Gain	30
12	Relaxation Delay	1.0000
13	Pulse Width	5.2500
14	Acquisition Time	1.3107
15	Acquisition Date	2021-05-27T21:42:44
16	Spectrometer Frequency	100.53
17	Spectral Width	25000.0
18	Lowest Frequency	-1442.7
19	Nucleus	13C
20	Acquired Size	32768
21	Spectral Size	65536
22	Digital Resolution	0.38

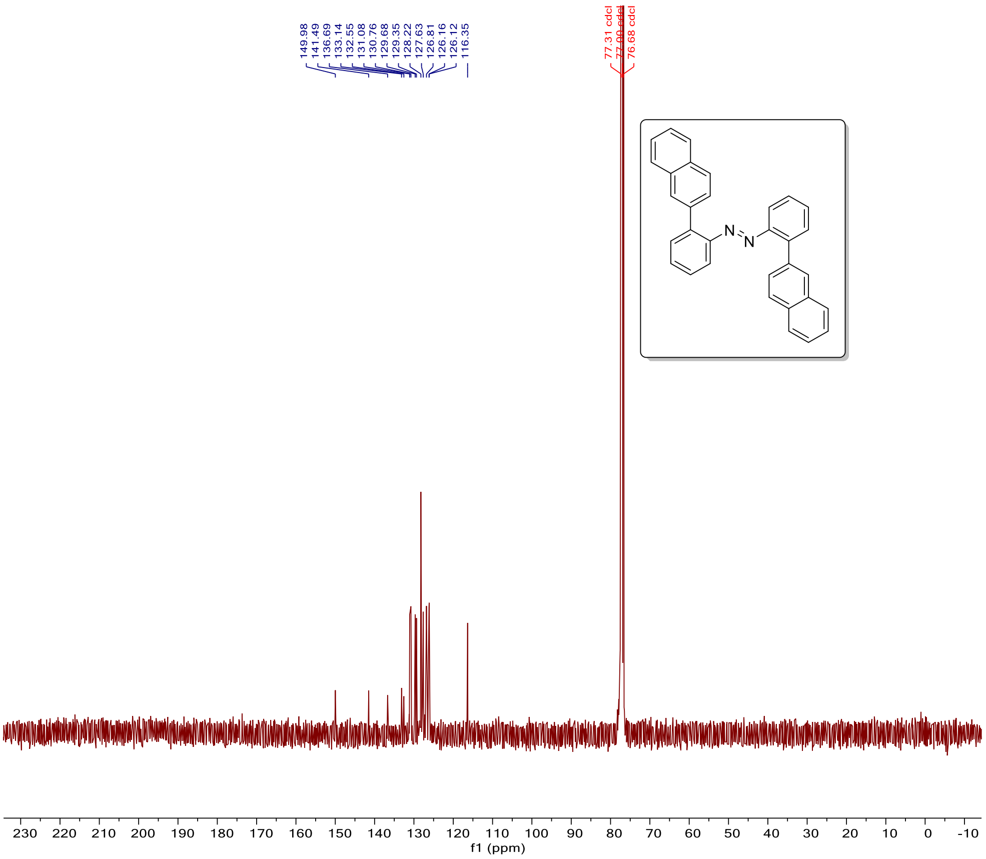

^{1}H NMR spectrum of compound **2p** (400 MHz, $CDCl_3$)

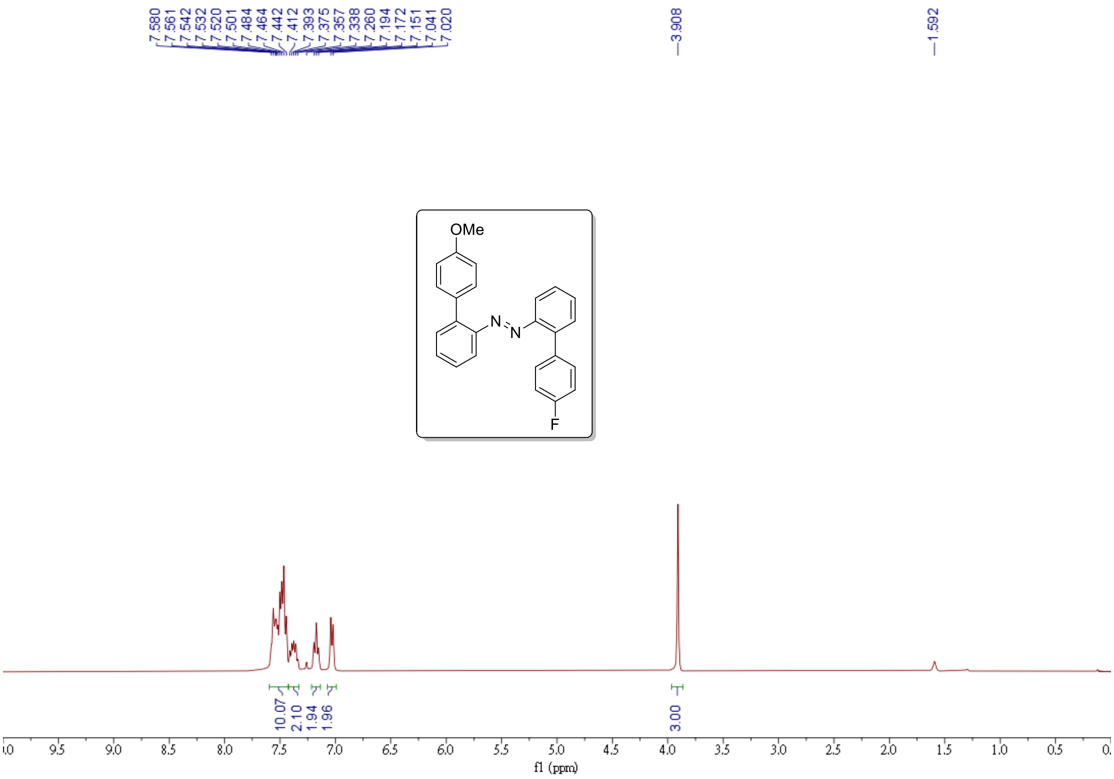

^{13}C NMR spectrum of compound **2p** (100 MHz, $CDCl_3$)

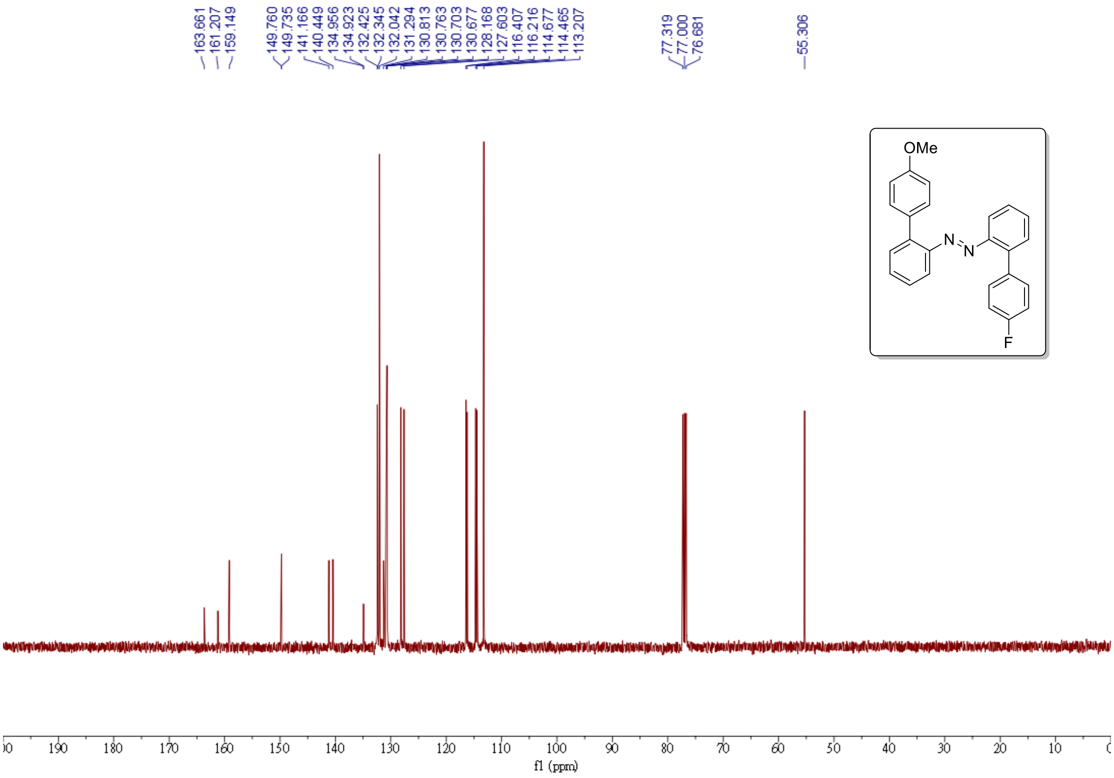

^{1}H NMR spectrum of compound **2q** ($CDCl_3$, 400 MHz)

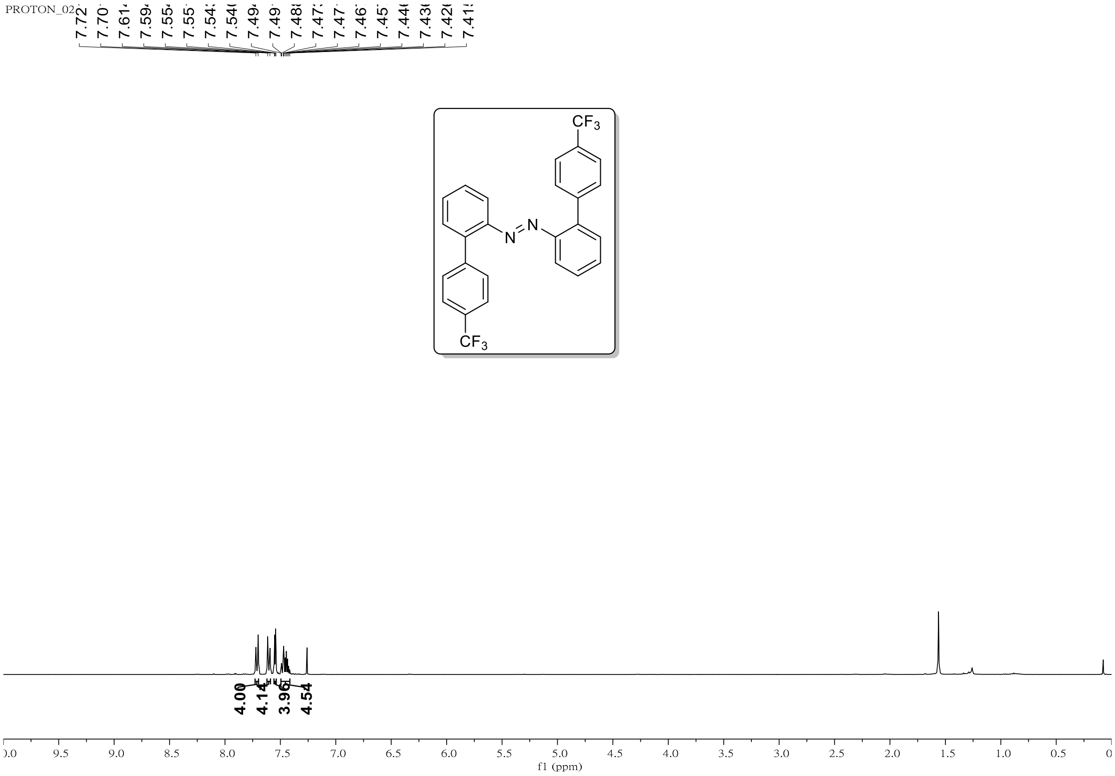

^{13}C NMR spectrum of compound **2q** ($CDCl_3$, 100 MHz)

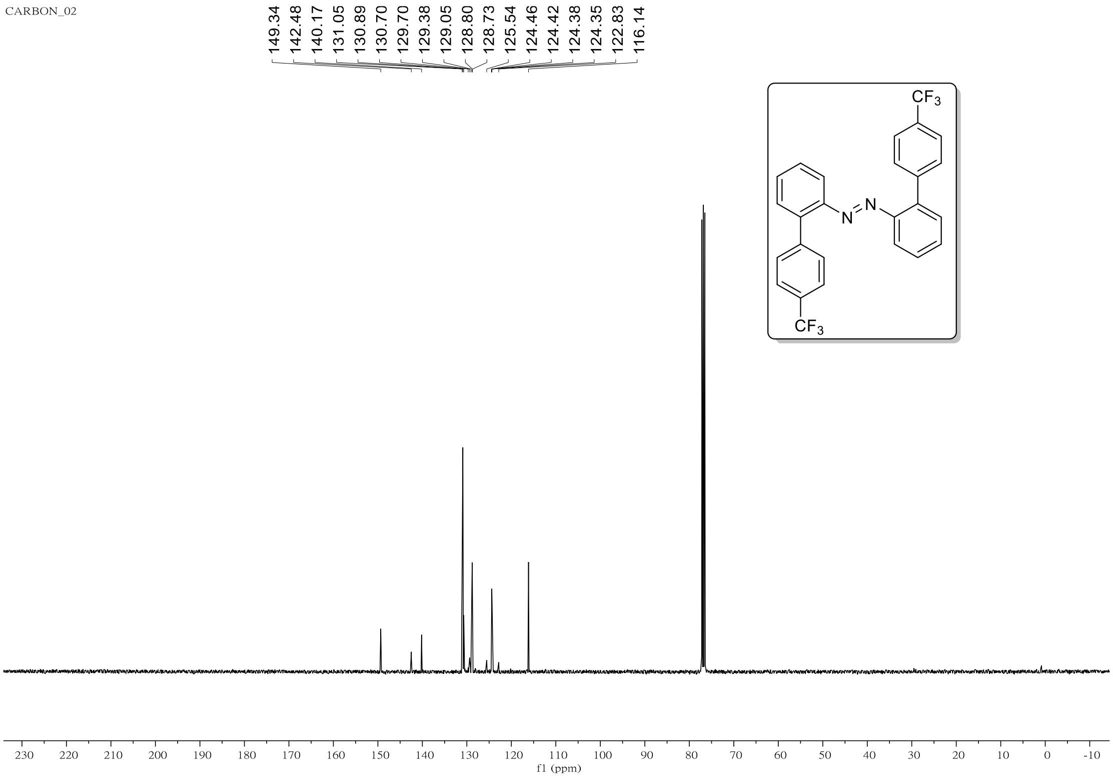

^{1}H NMR spectrum of compound **2v** (400 MHz, $CDCl_3$)

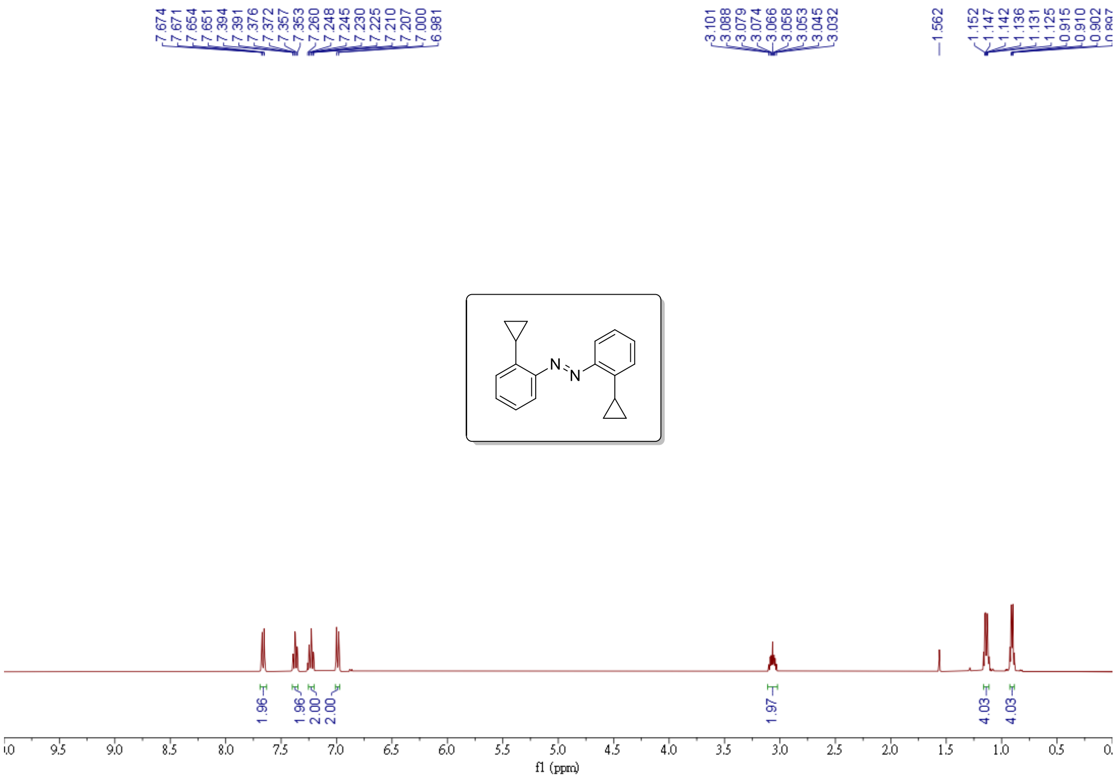

^{13}C NMR spectrum of compound **2v** (100 MHz, $CDCl_3$)

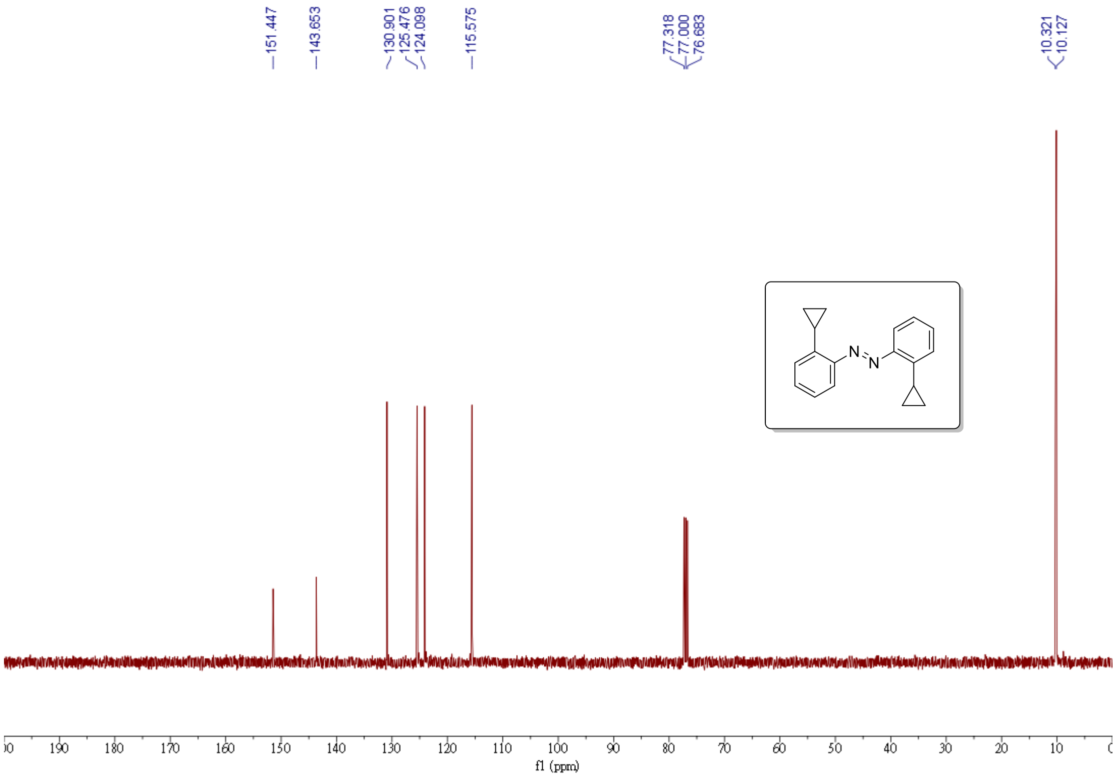

^{1}H NMR spectrum of compound **3a** (400 MHz, DMSO-d_6)

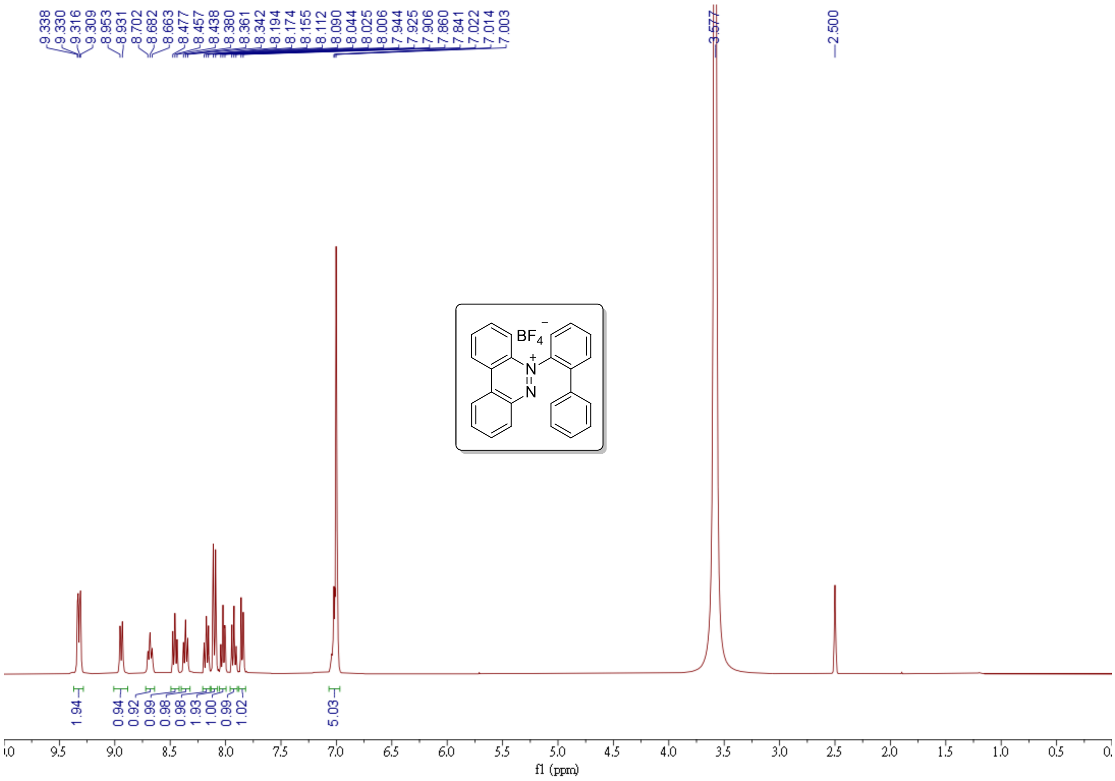

^{13}C NMR spectrum of compound **3a** (100 MHz, DMSO-d_6)

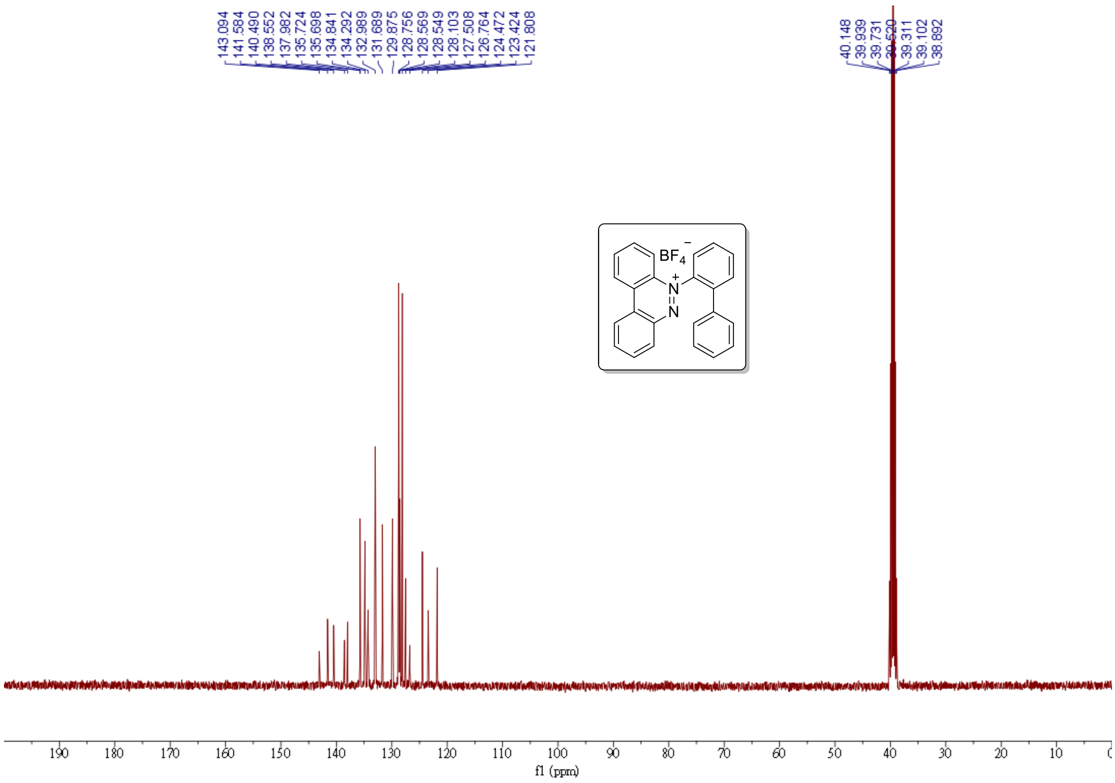

^{1}H NMR spectrum of compound **3b** (400 MHz, CD_3OD)

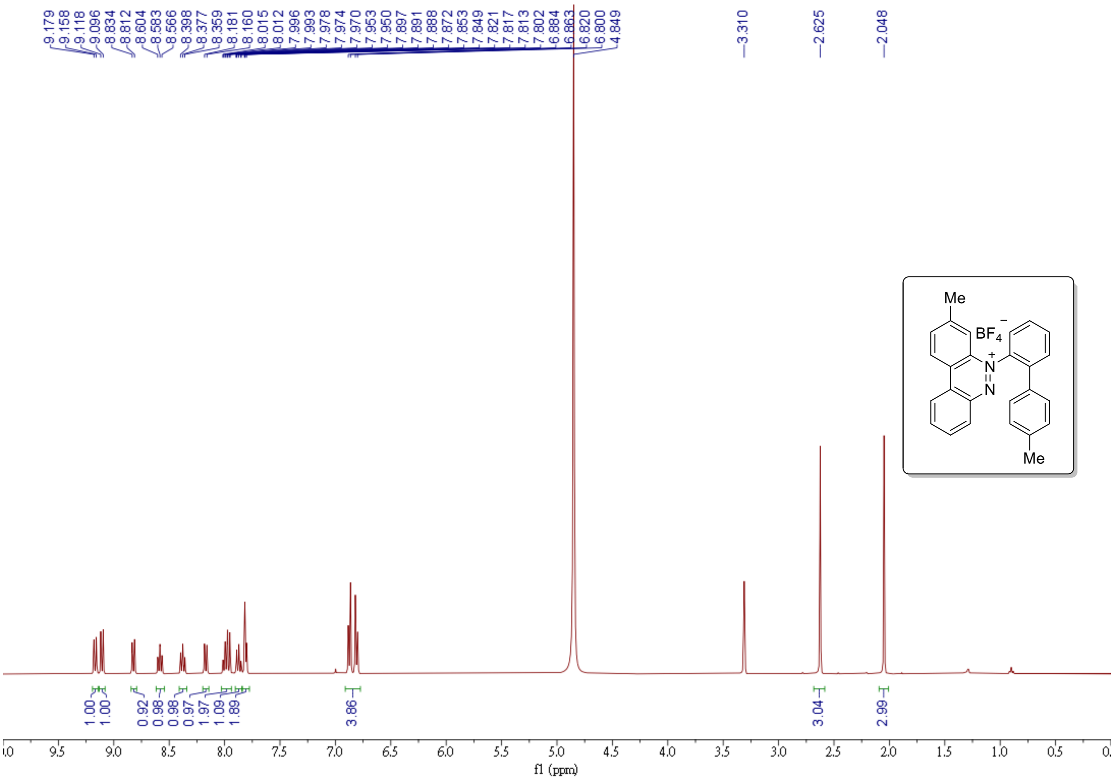

^{13}C NMR spectrum of compound **3b** (100 MHz, CD_3OD)

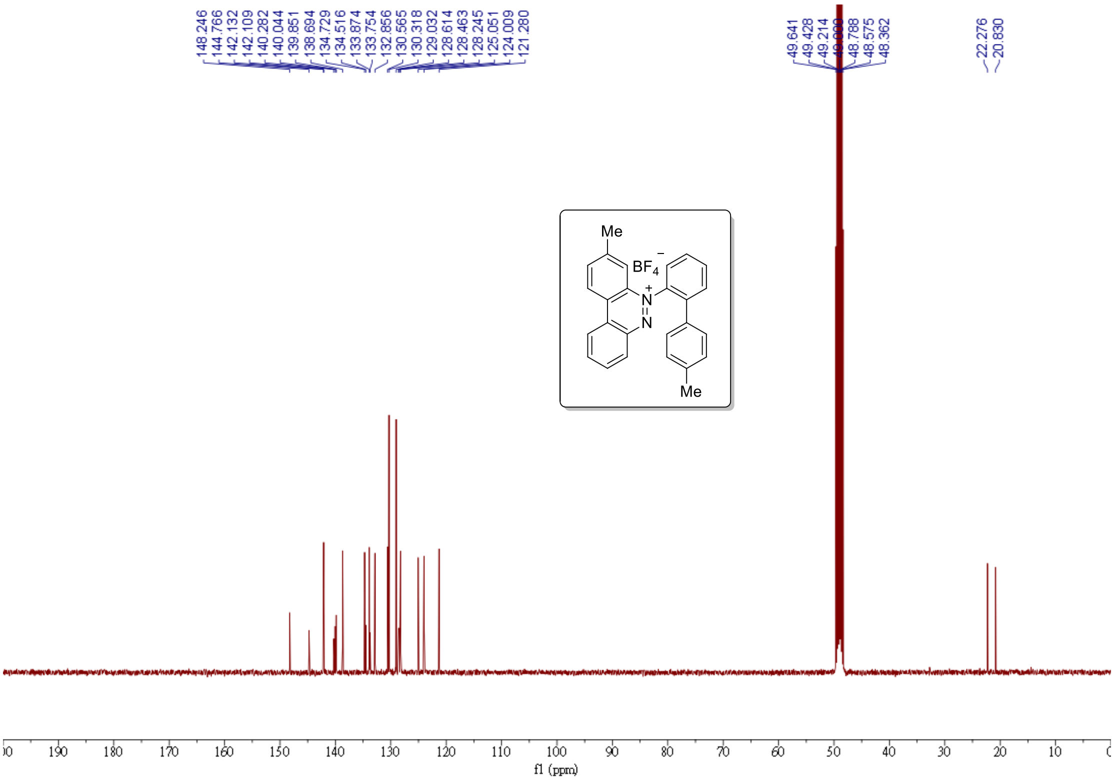

^{1}H NMR spectrum of compound **3c** (400 MHz, $CDCl_3$)

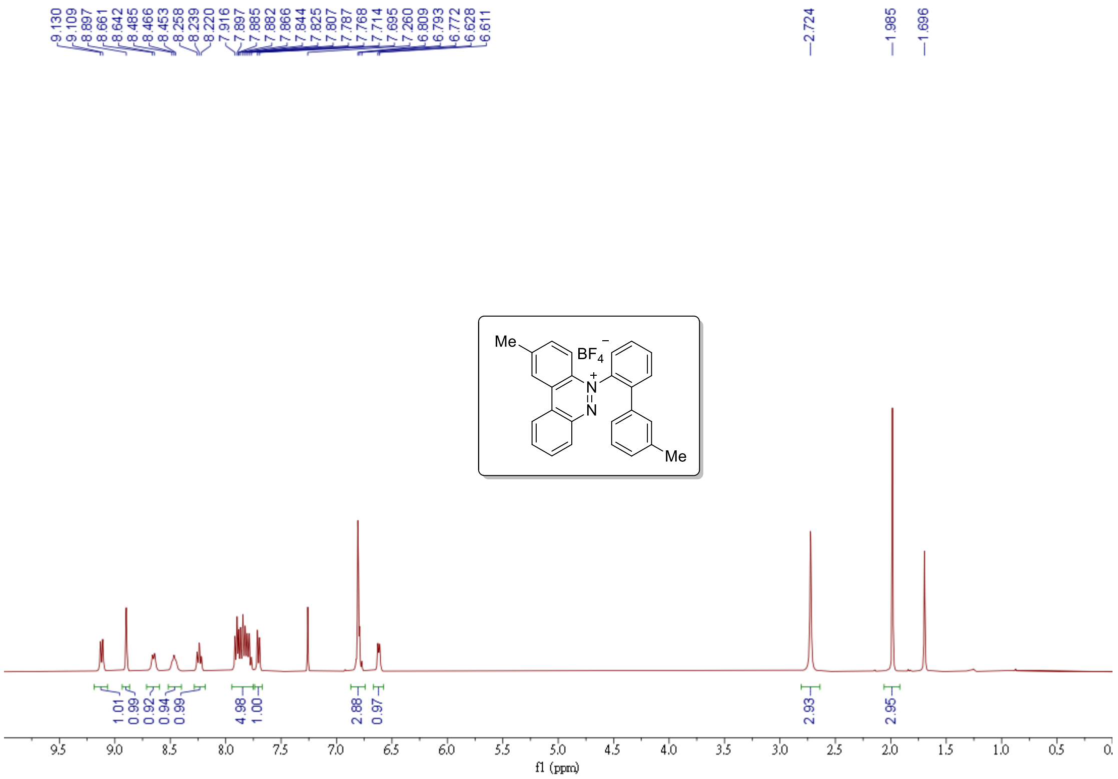

^{13}C NMR spectrum of compound **3c** (100 MHz, $CDCl_3$)

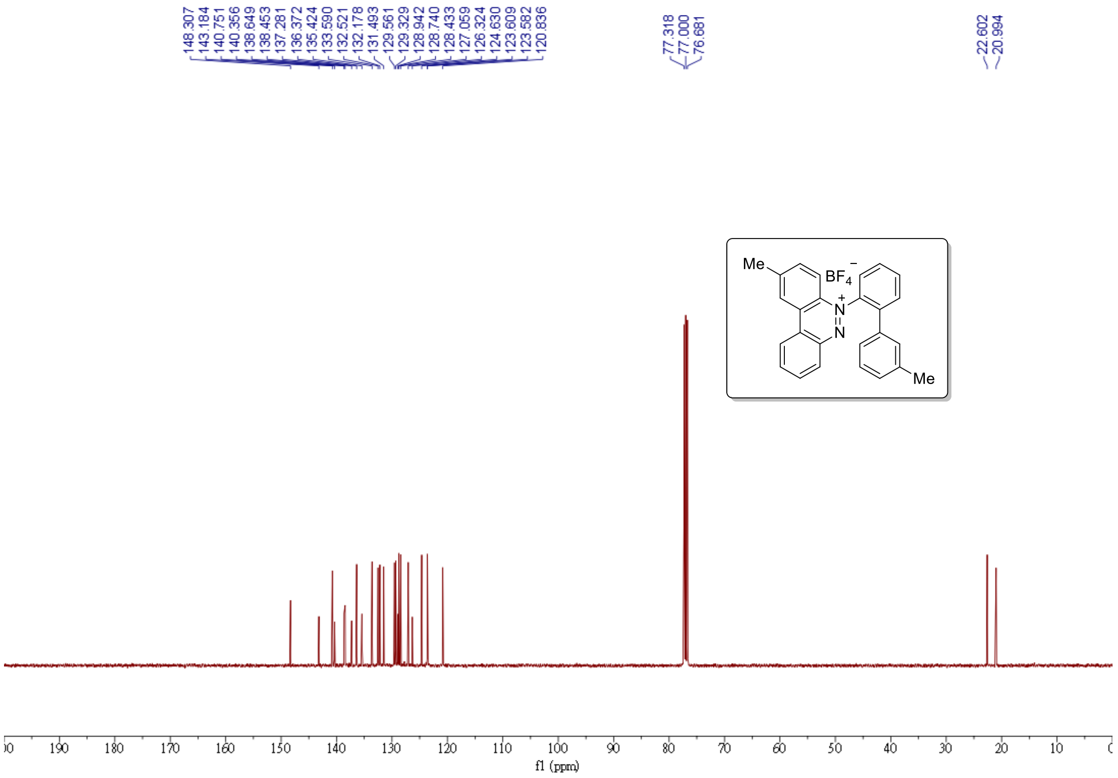

^{1}H NMR spectrum of compound **3d** (400 MHz, CD_3OD)

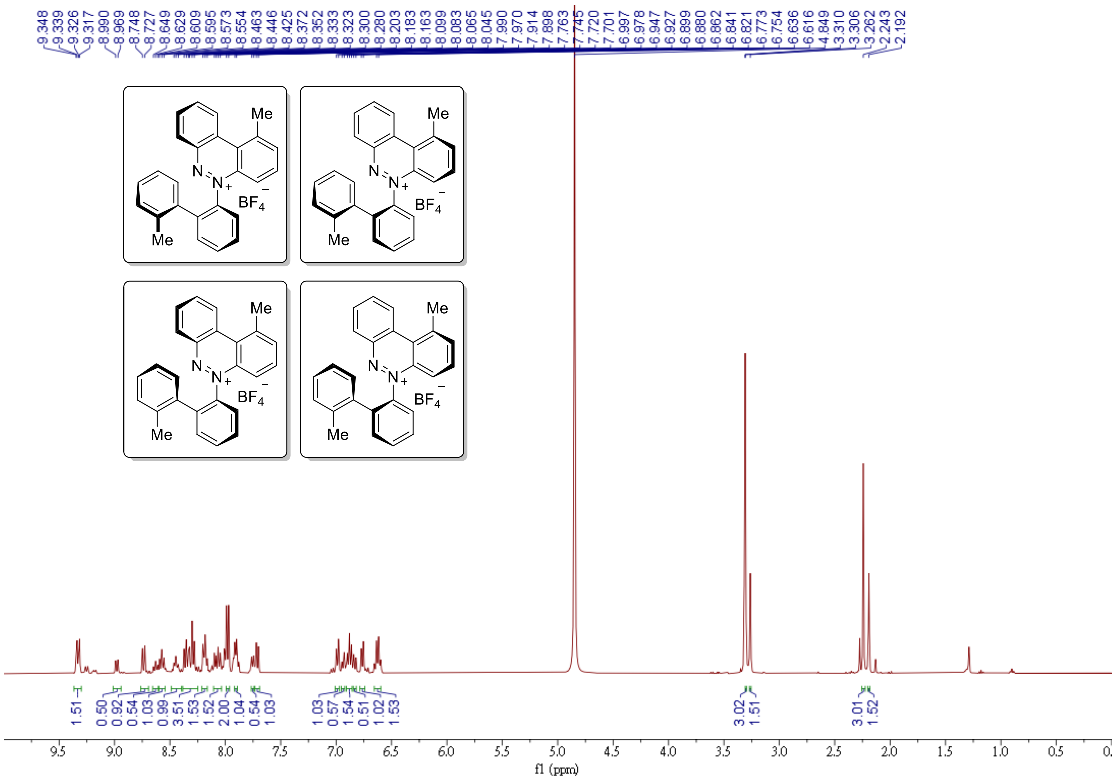

^{13}C NMR spectrum of compound **3d** (100 MHz, CD_3OD)

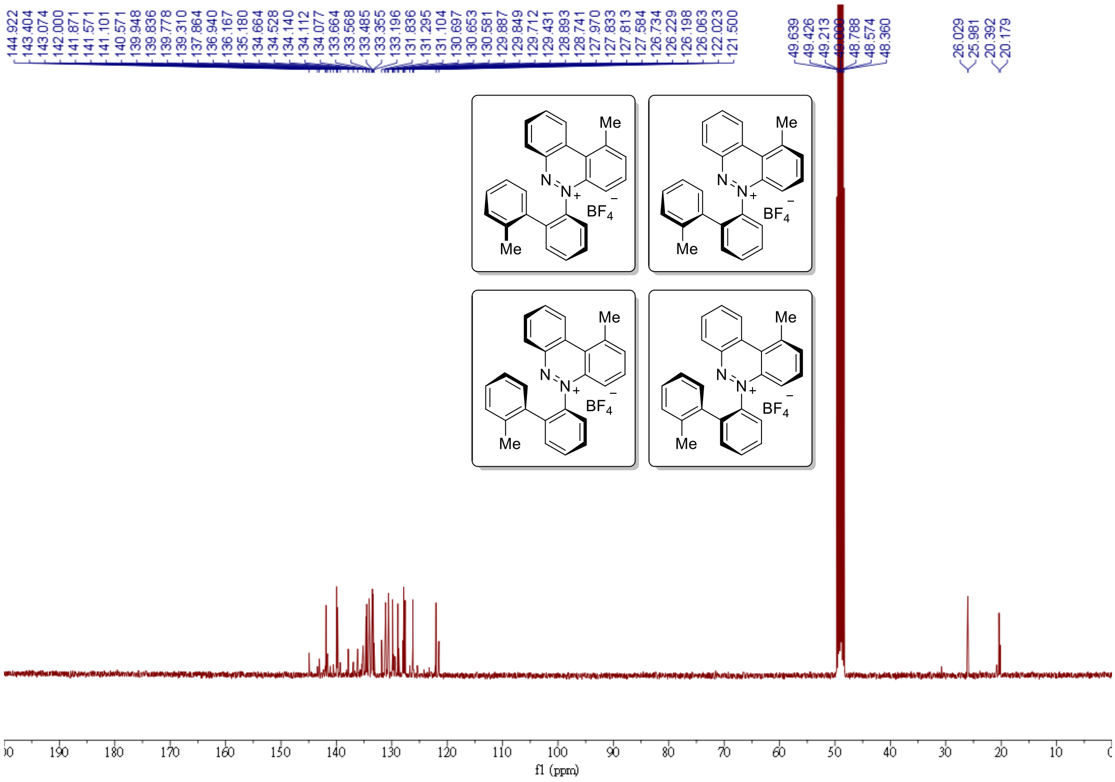

^{1}H NMR spectrum of compound **3e** (400 MHz, $CD_3OD/CDCl_3$ = 6/1)

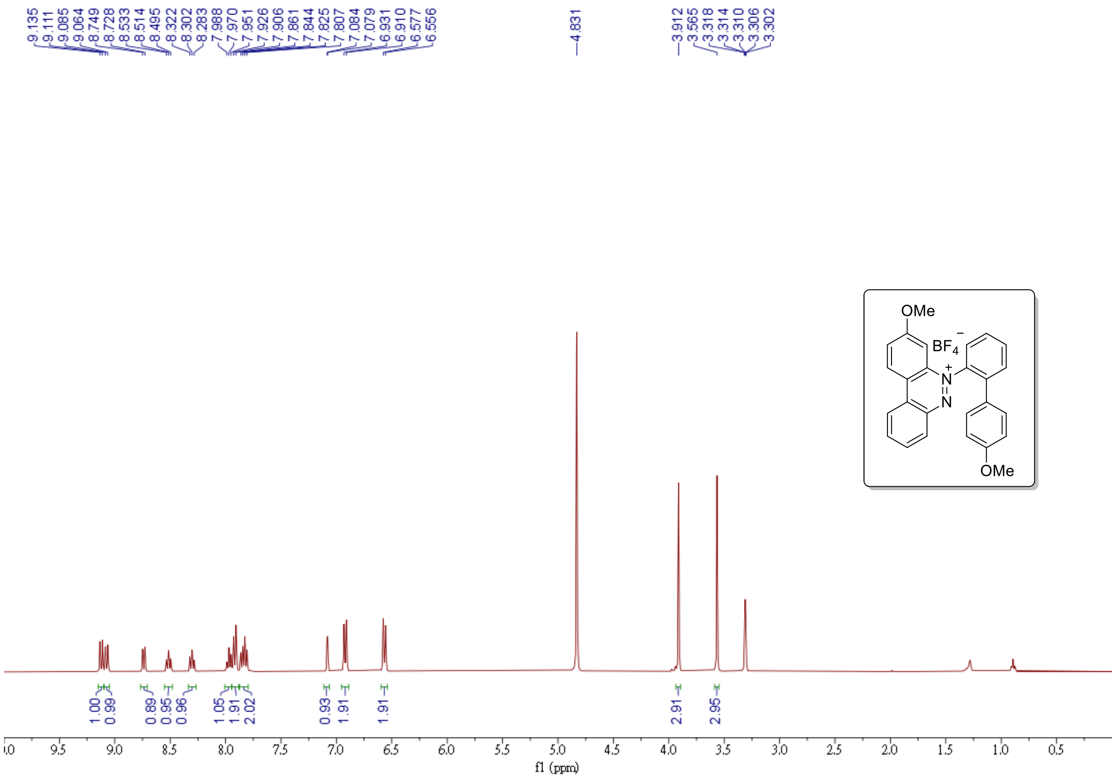

^{13}C NMR spectrum of compound **3e** (100 MHz, $CD_3OD/CDCl_3$ = 6/1)

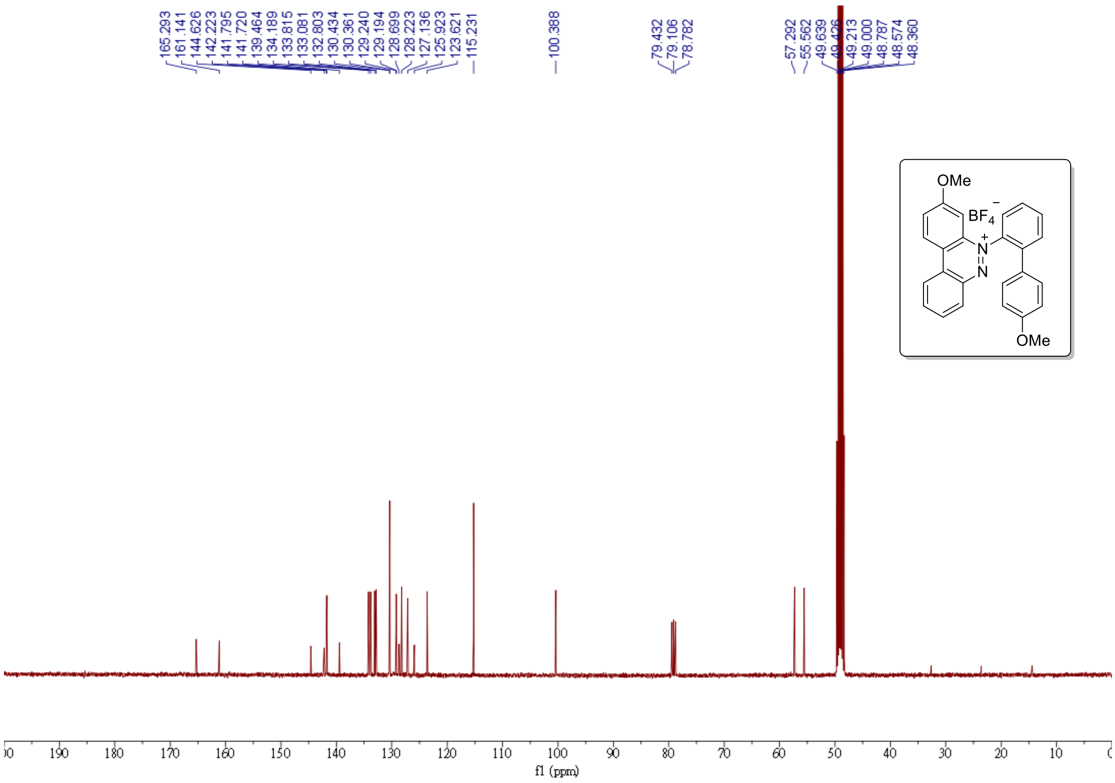

^{1}H NMR spectrum of compound **3f** (400 MHz, CD_3OD)

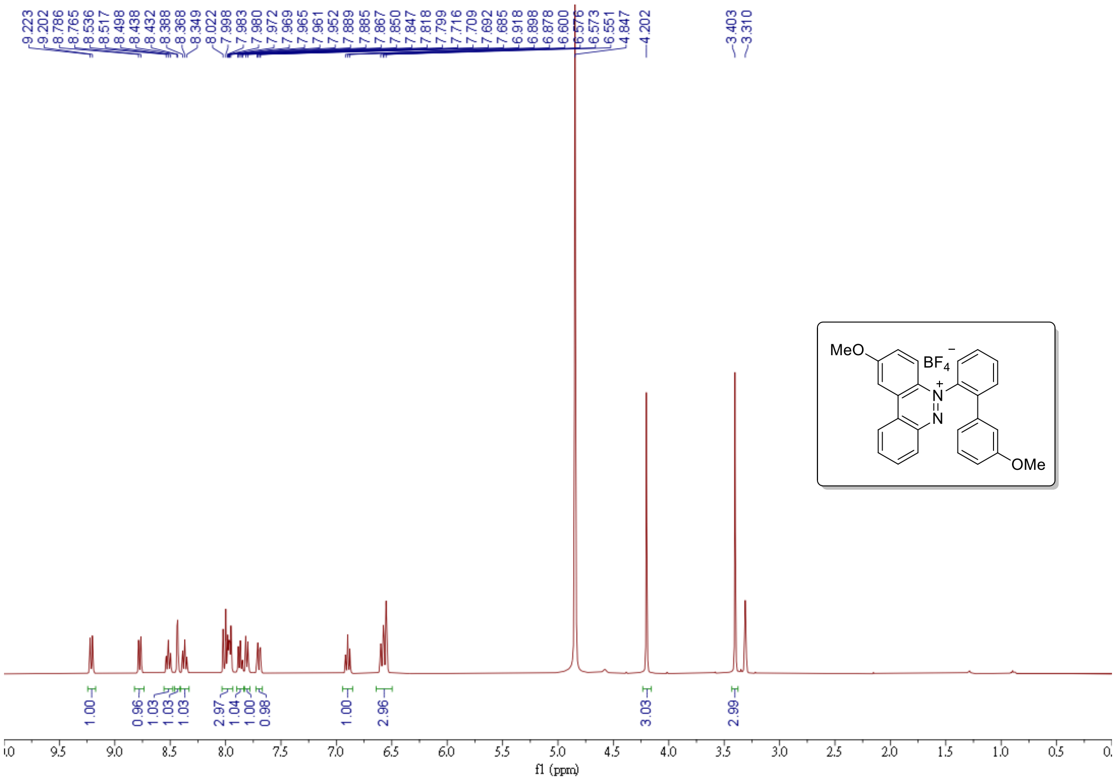

^{13}C NMR spectrum of compound **3f** (100 MHz, CD_3OD)

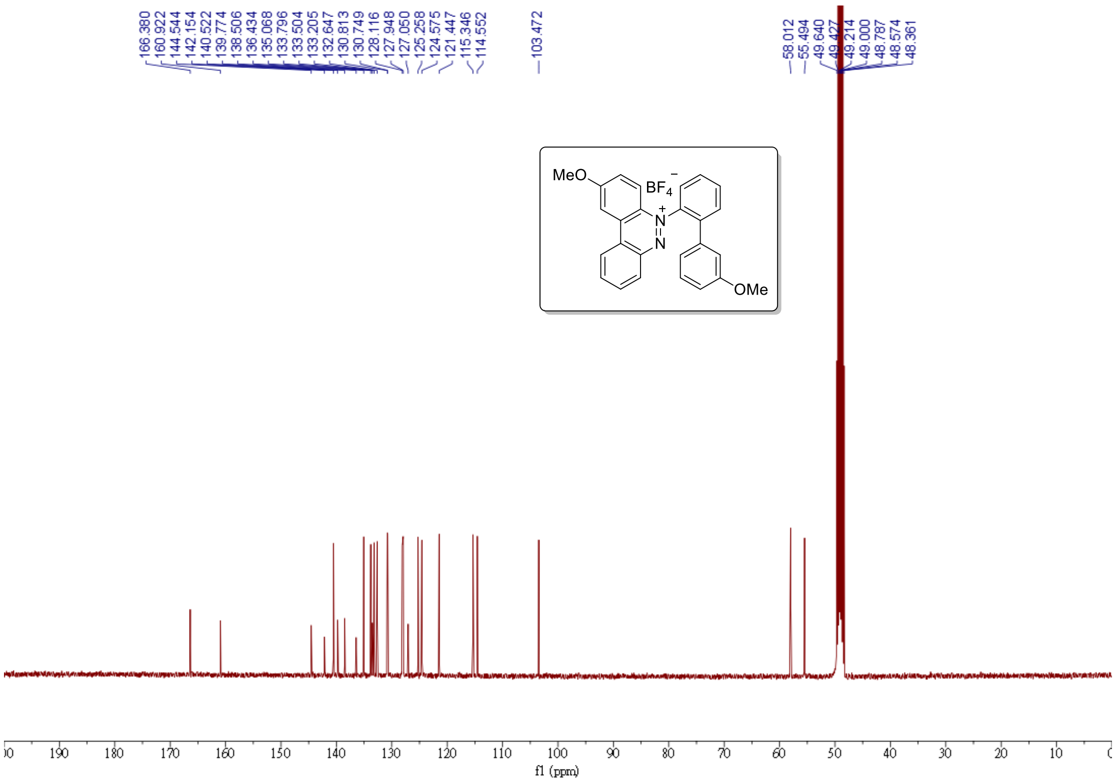

^{1}H NMR spectrum of compound **3g** (400 MHz, CD_3OD)

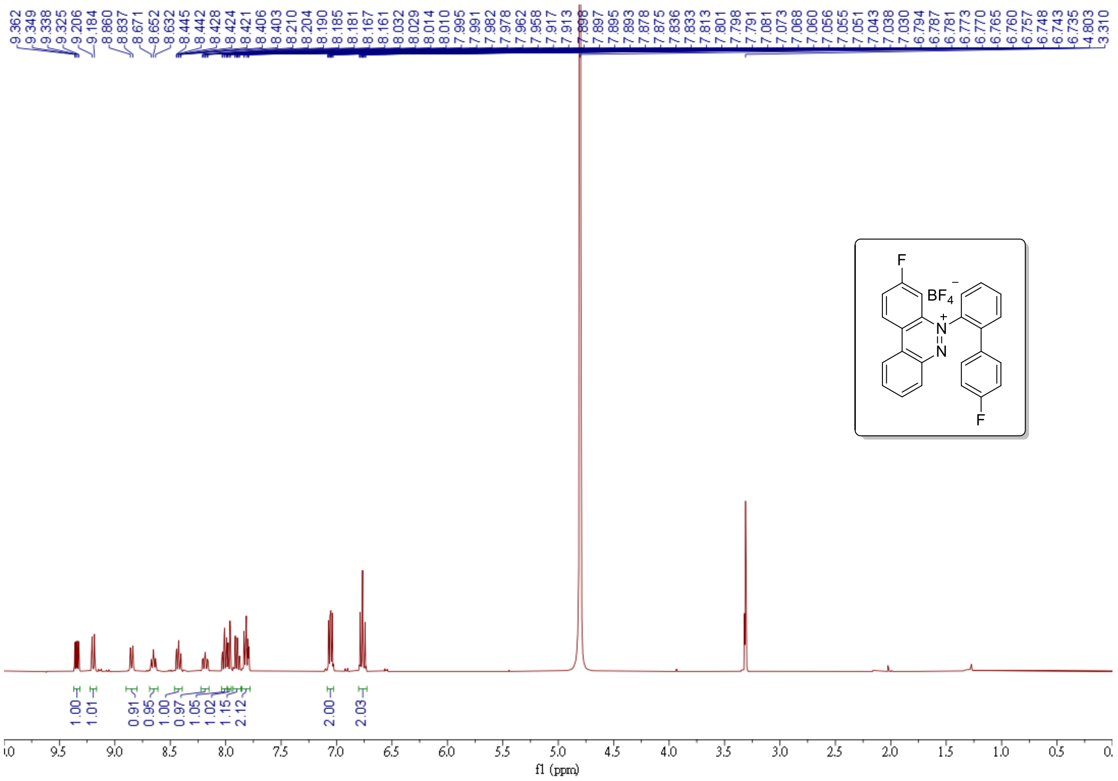

^{13}C NMR spectrum of compound **3g** (100MHz, CD_3OD)

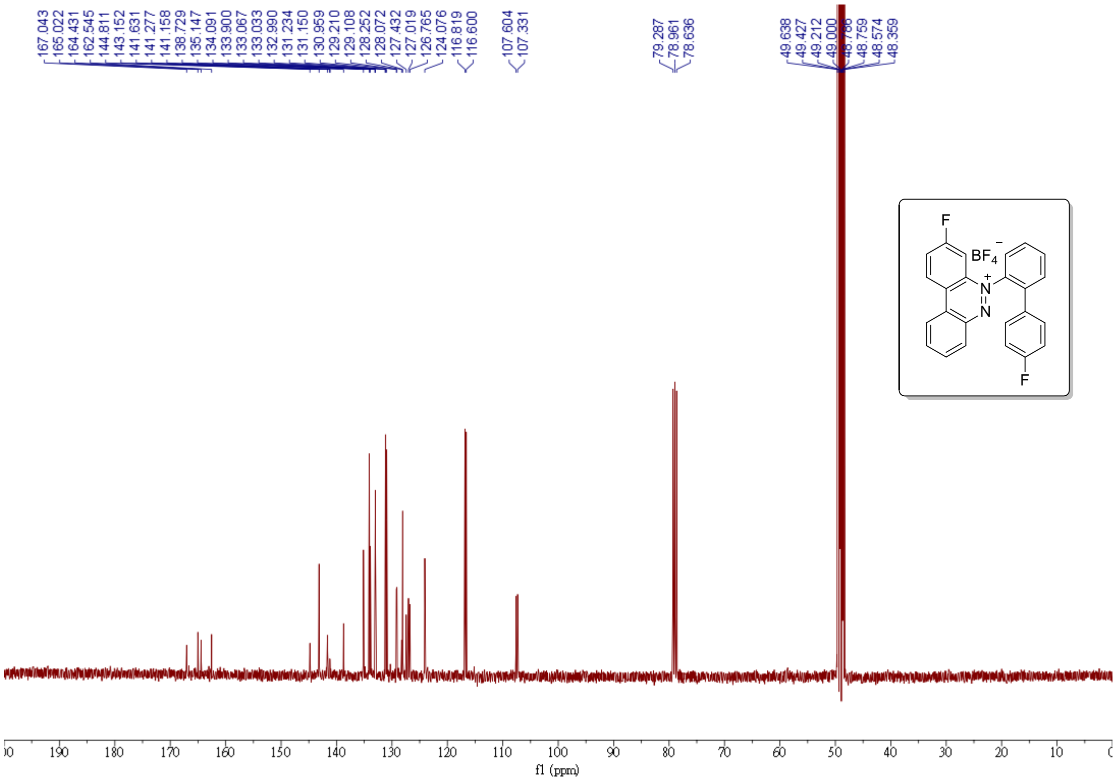

^{1}H NMR spectrum of compound **3h** (400 MHz, CD_3OD)

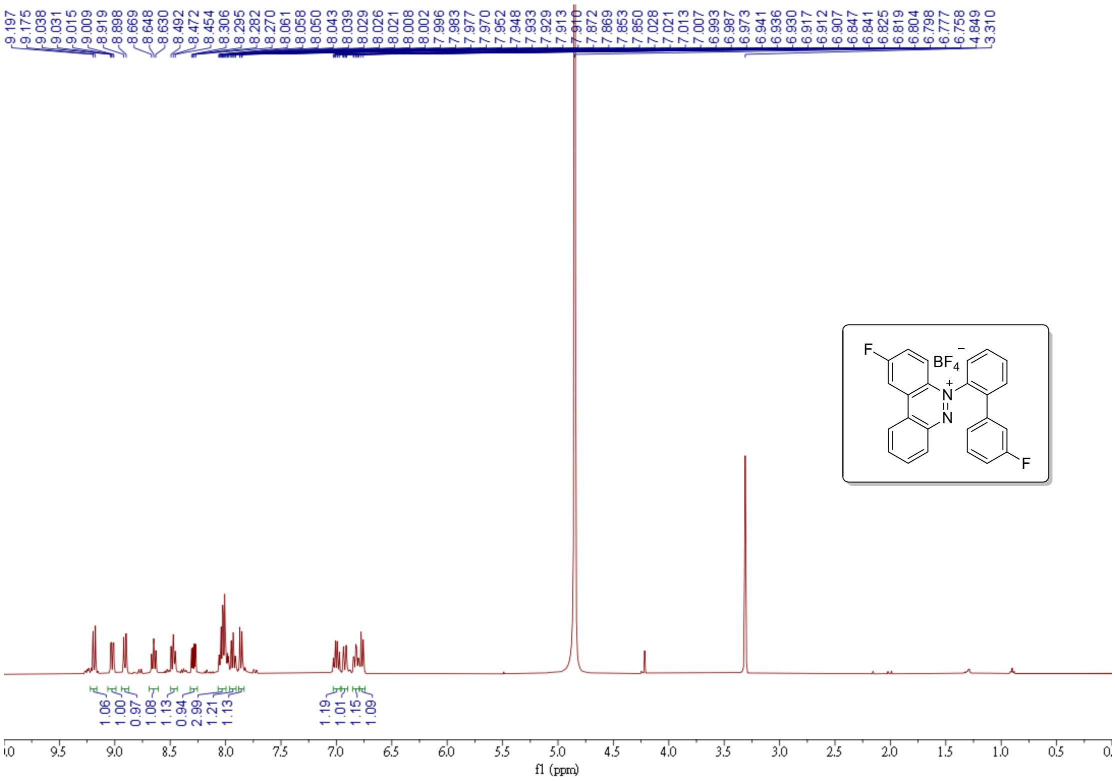

^{13}C NMR spectrum of compound **3h** (100MHz, CD_3OD)

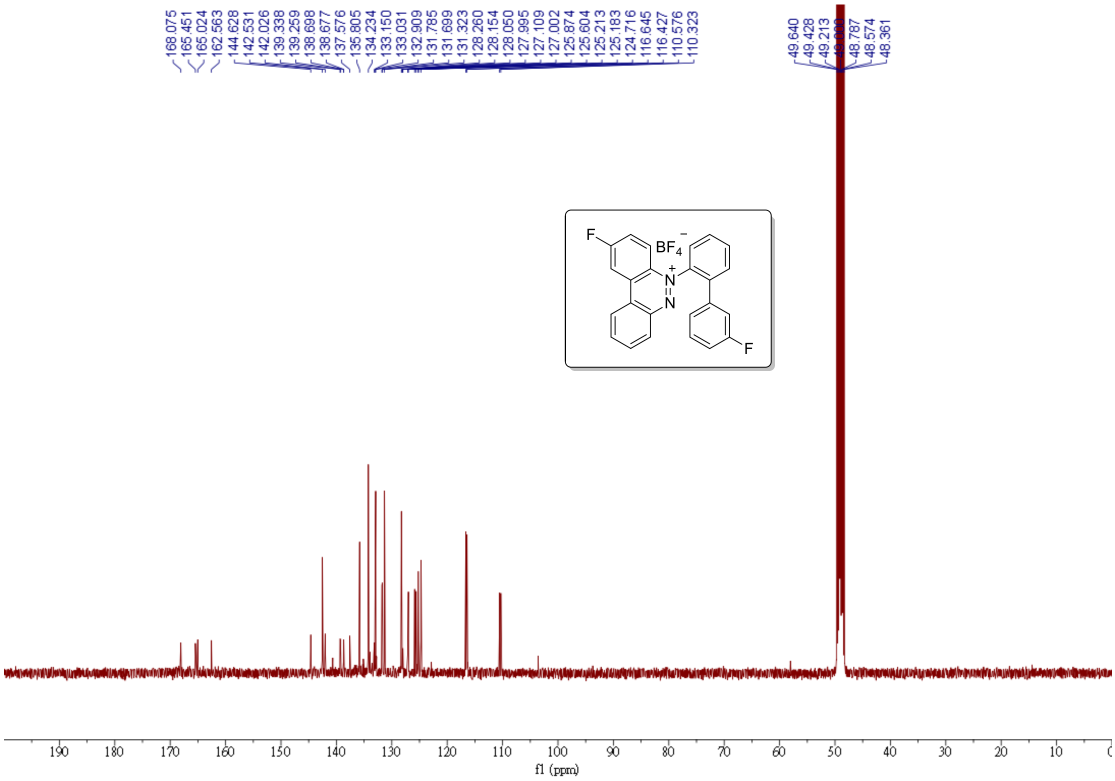

^{1}H NMR spectrum of compound **3i** (400 MHz, DMSO-d$_6$)

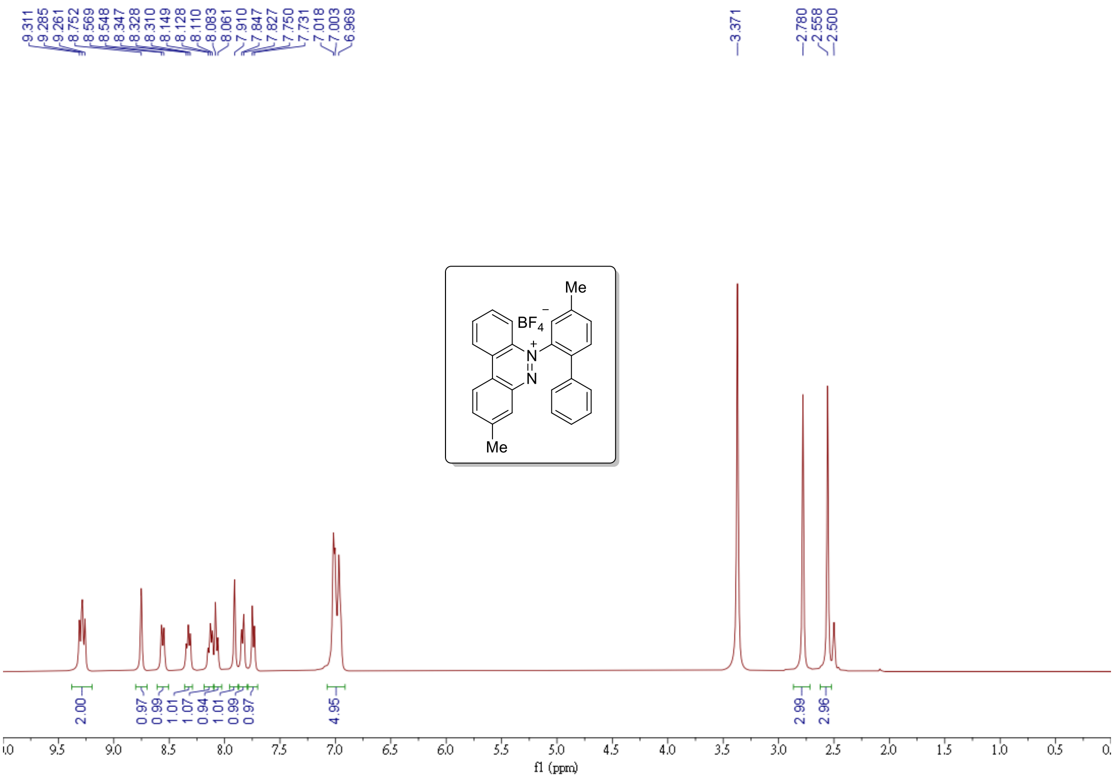

^{13}C NMR spectrum of compound **3i** (100 MHz, DMSO-d$_6$)

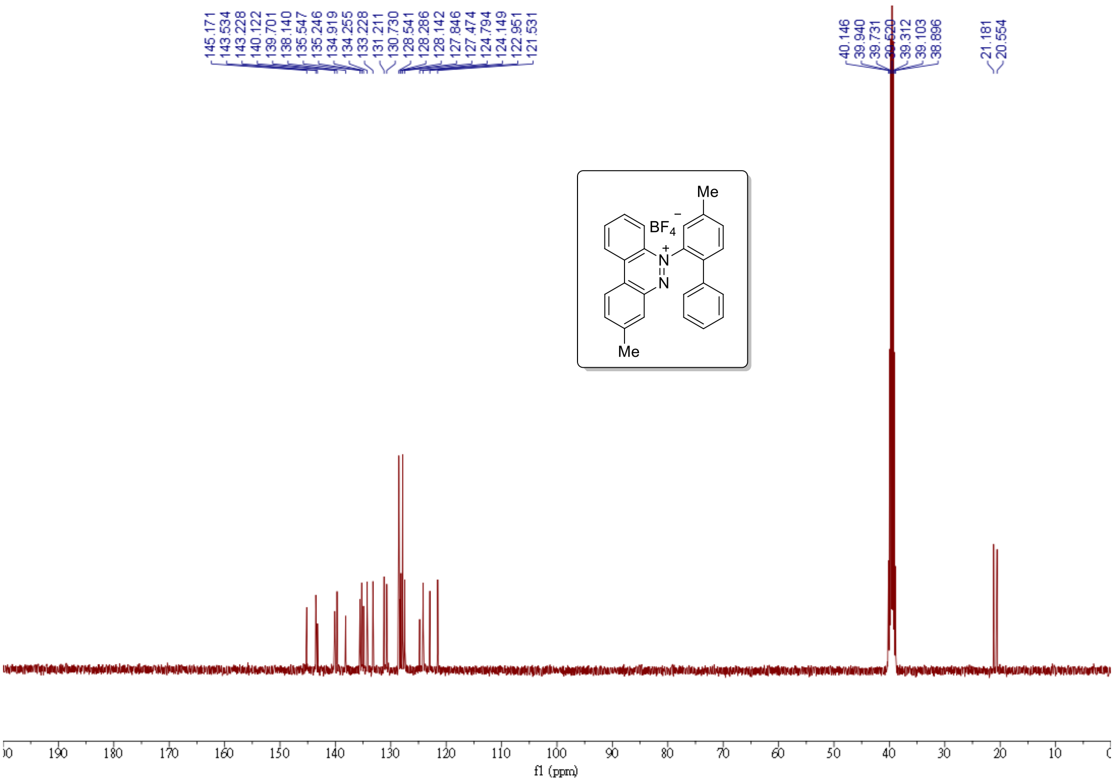

^{1}H NMR spectrum of compound **3j** (400 MHz, $CDCl_3$:CD_3OD = 5:1)

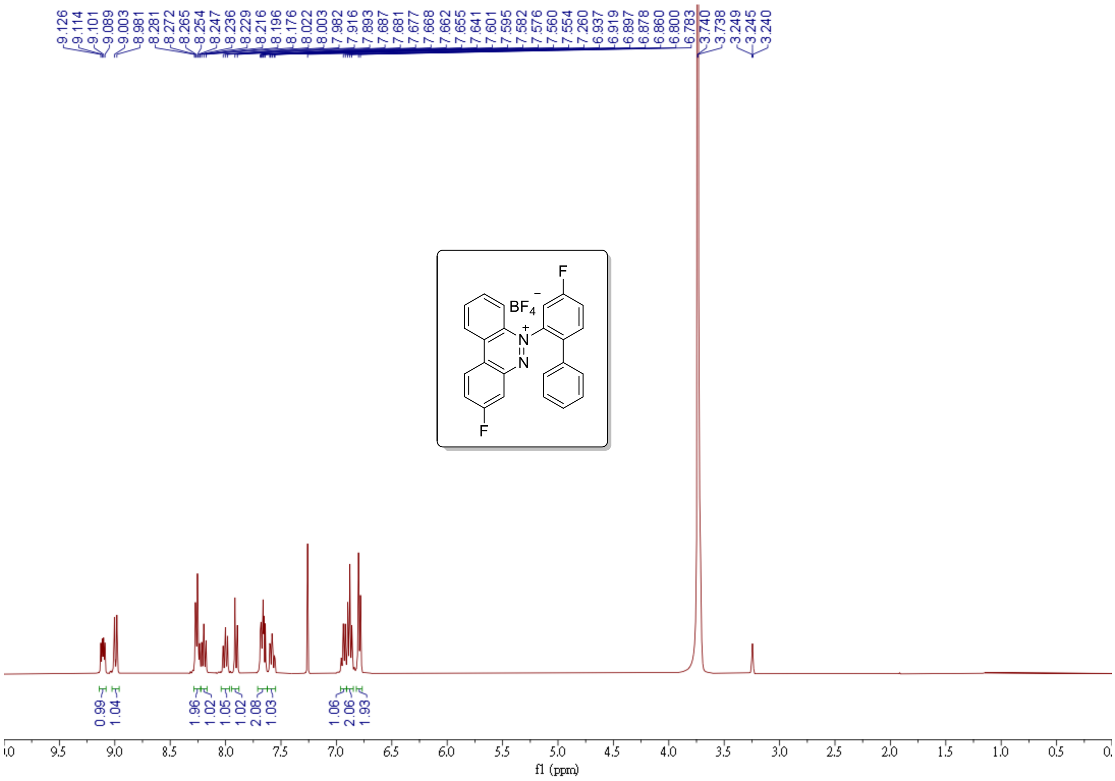

^{13}C NMR spectrum of compound **3j** (100 MHz, $CDCl_3$:CD_3OD = 5:1)

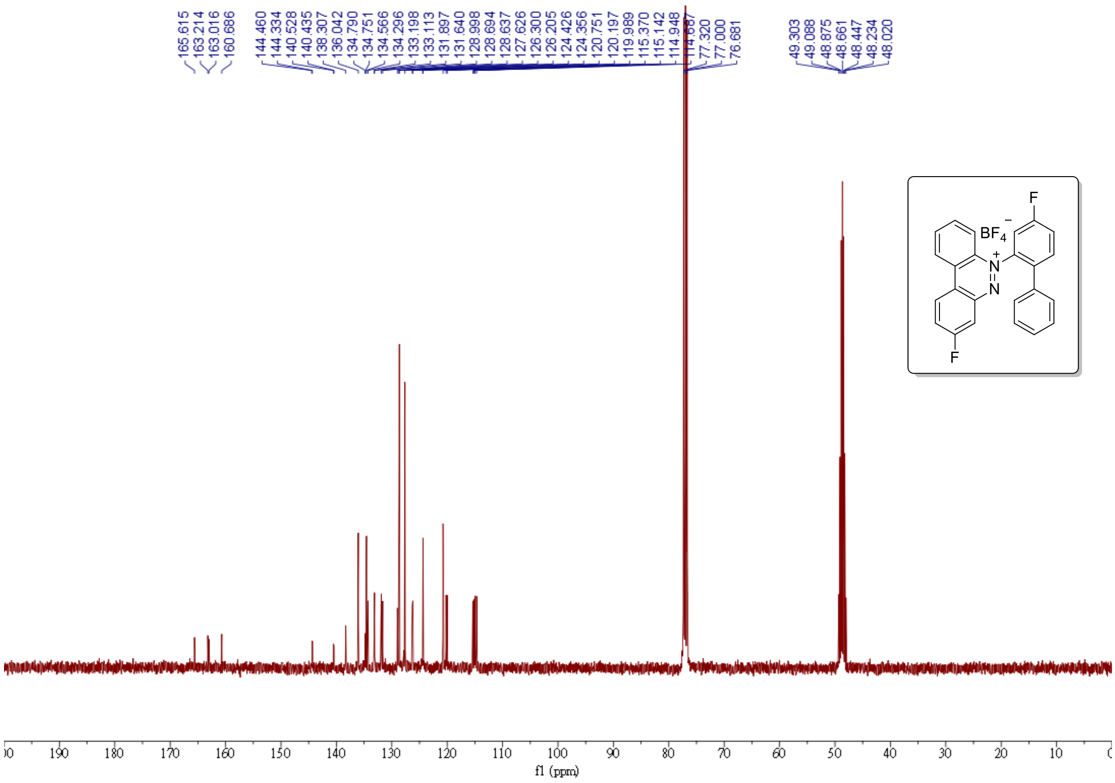

^{1}H NMR spectrum of compound **3k** (400 MHz, $CDCl_3$)

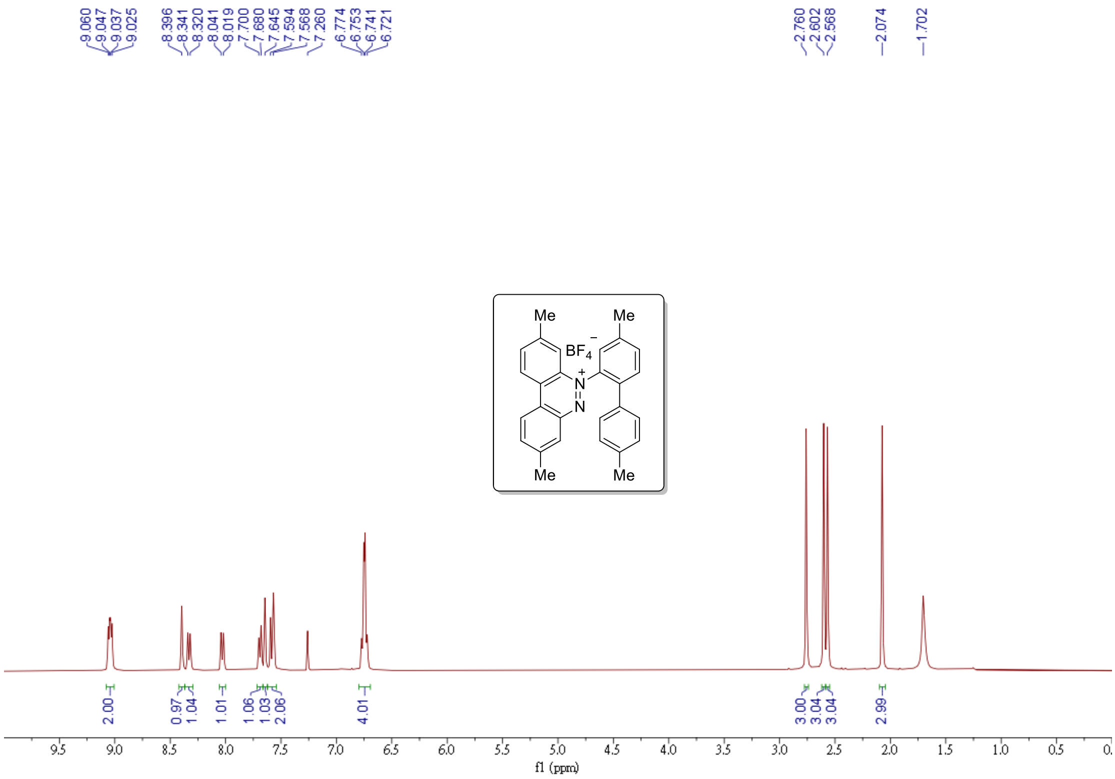

^{13}C NMR spectrum of compound **3k** (100 MHz, $CDCl_3$)

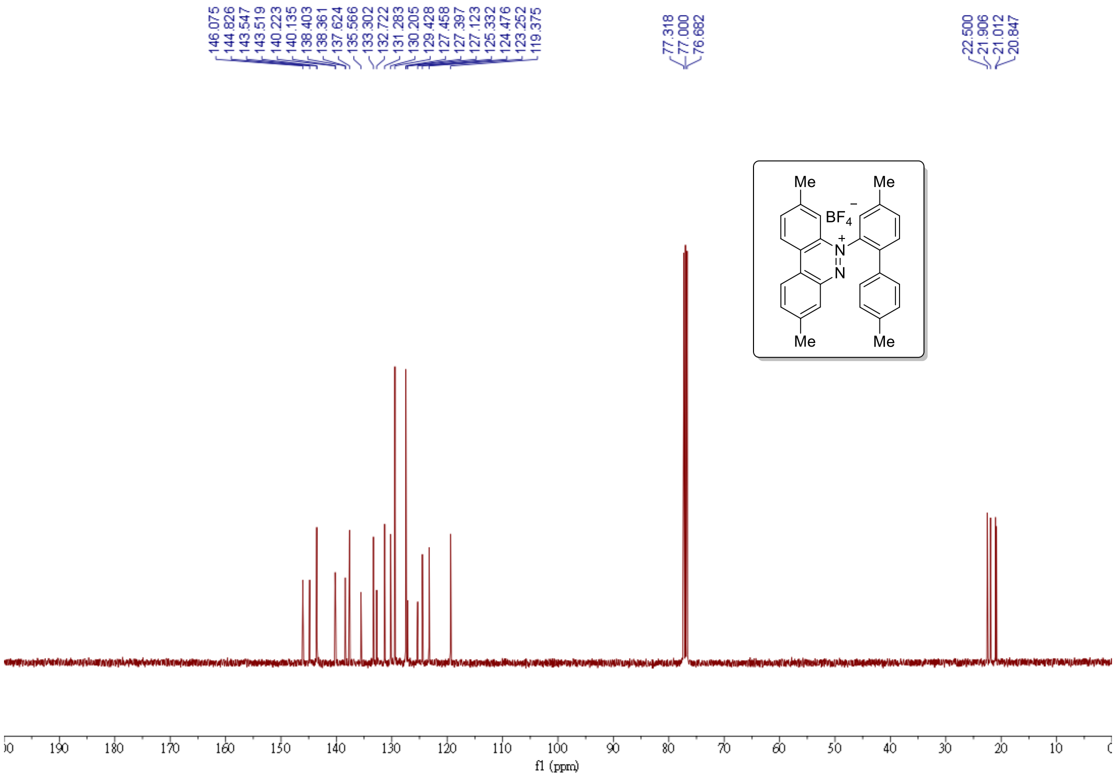

^{1}H NMR spectrum of compound **3l** (400 MHz, $CDCl_3$)

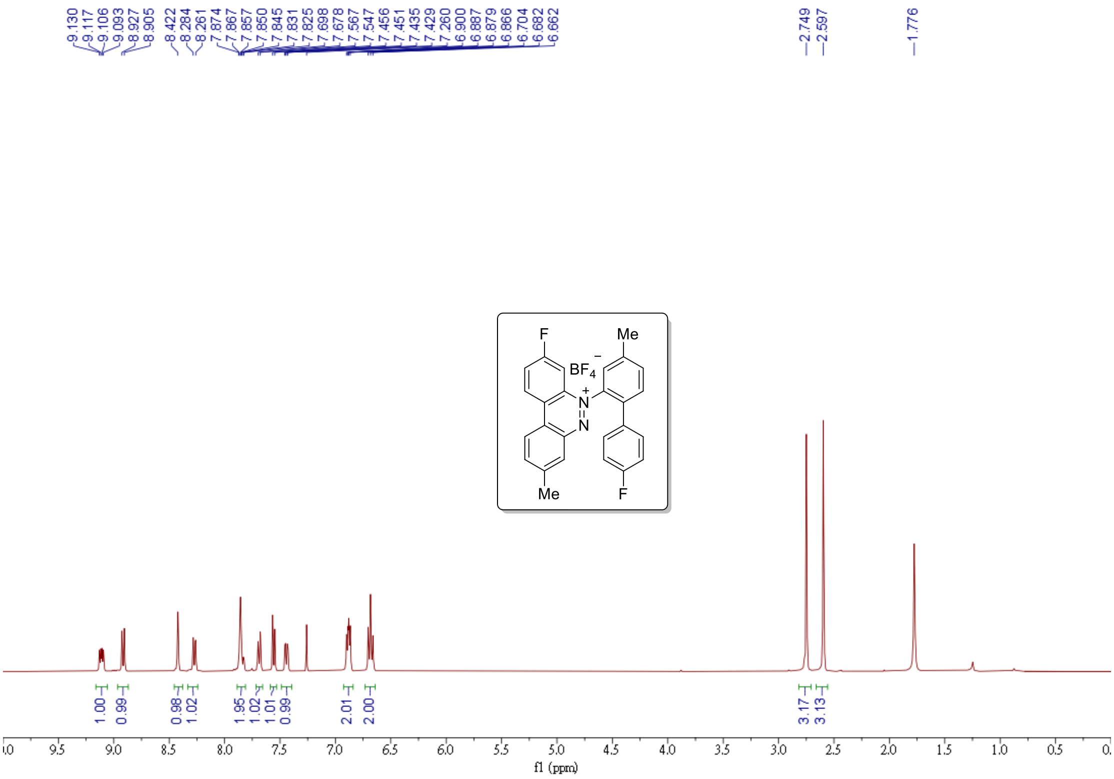

^{13}C NMR spectrum of compound **3l** (100 MHz, $CDCl_3$)

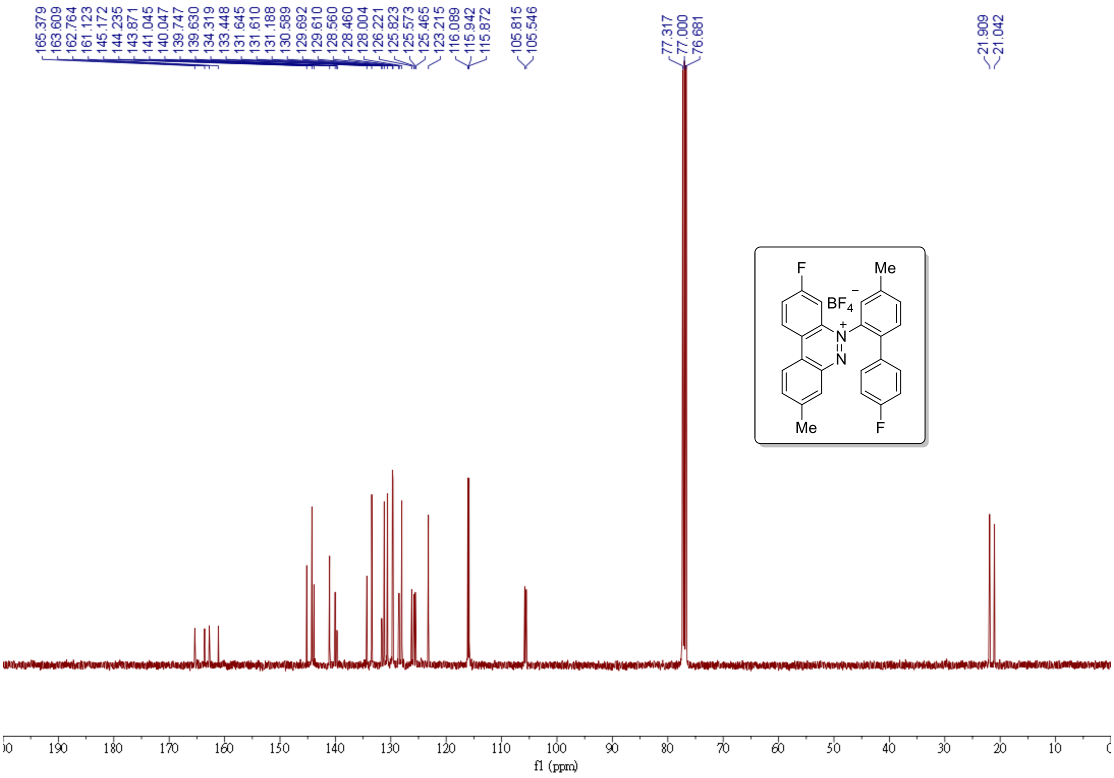

^{1}H NMR spectrum of compound **3m** (400 MHz, DMSO-d_6)

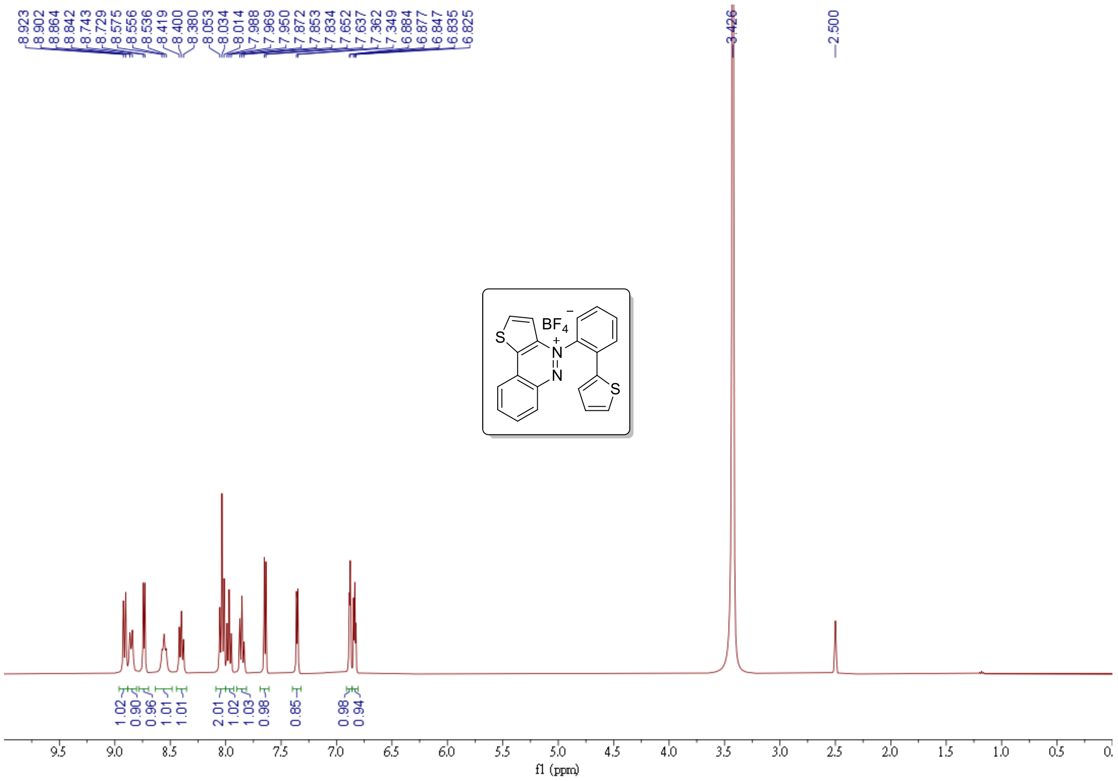

^{13}C NMR spectrum of compound **3m** (100 MHz, DMSO-d_6)

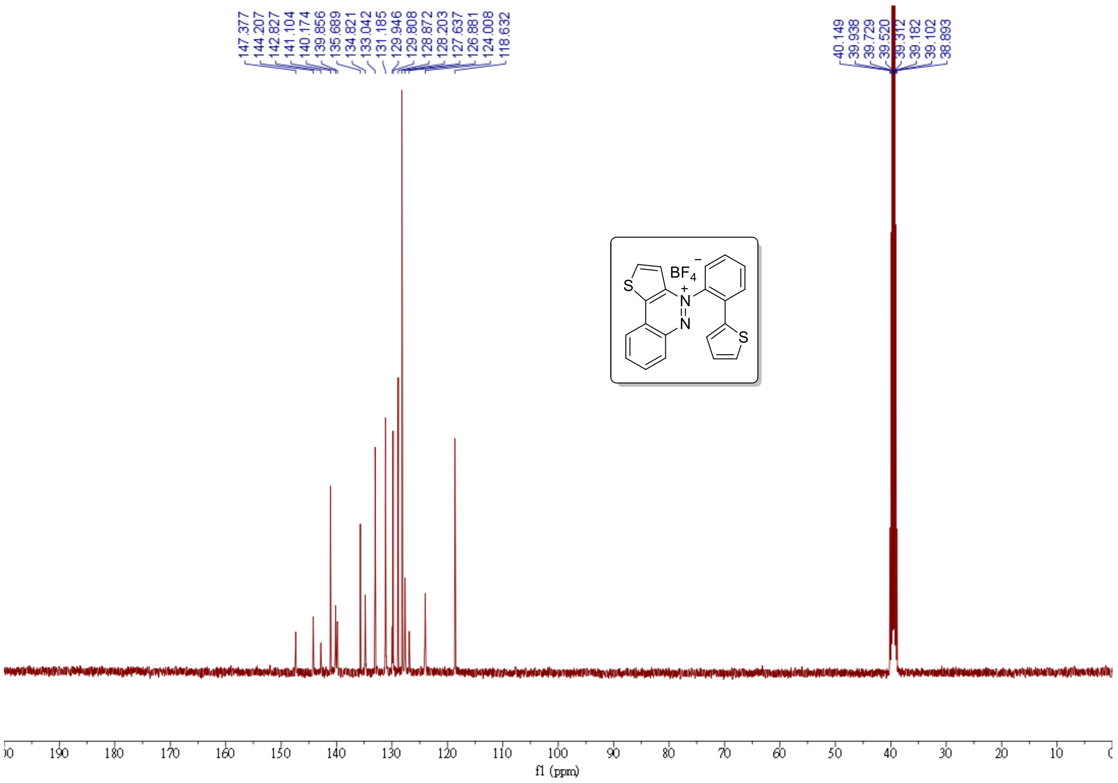

^{1}H NMR spectrum of compound **3n** (400 MHz, $CDCl_3$)

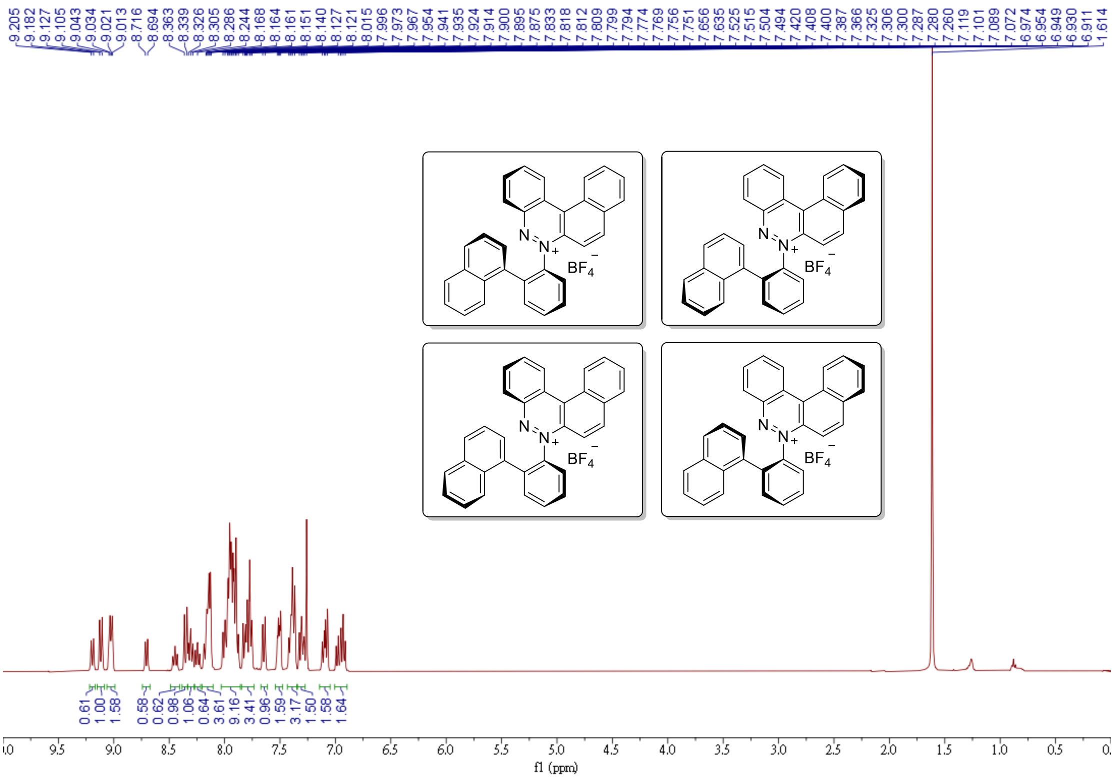

^{13}C NMR spectrum of compound **3n** (100 MHz, $CDCl_3$)

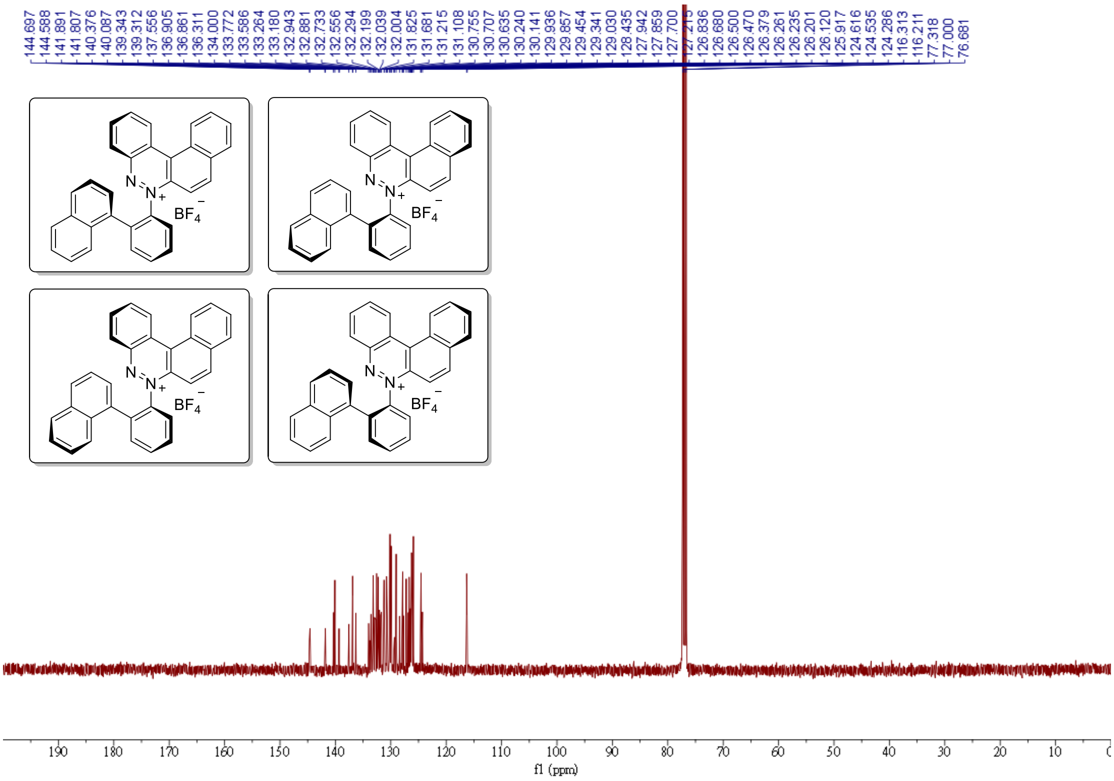

^{1}H NMR spectrum of compound **3o** (400 MHz, $CDCl_3$)

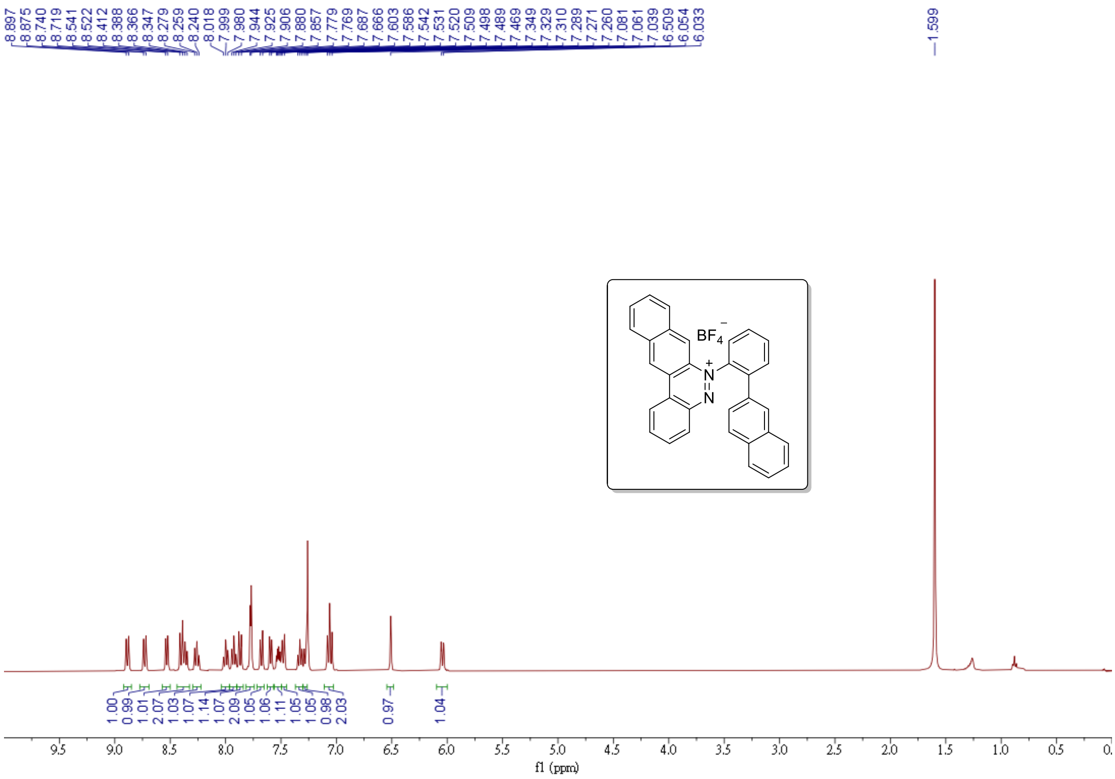

^{13}C NMR spectrum of compound **3o** (100 MHz, $CDCl_3$)

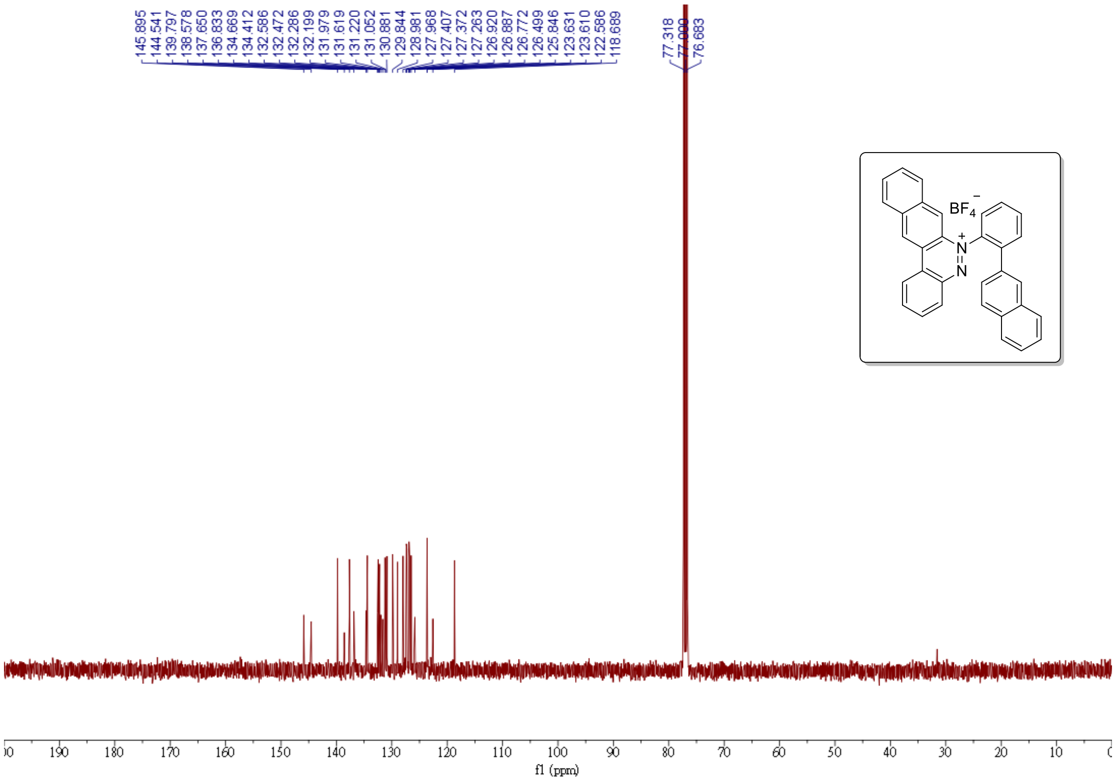

^{1}H NMR spectrum of compound **3p** (400 MHz, CD$_3$OD)

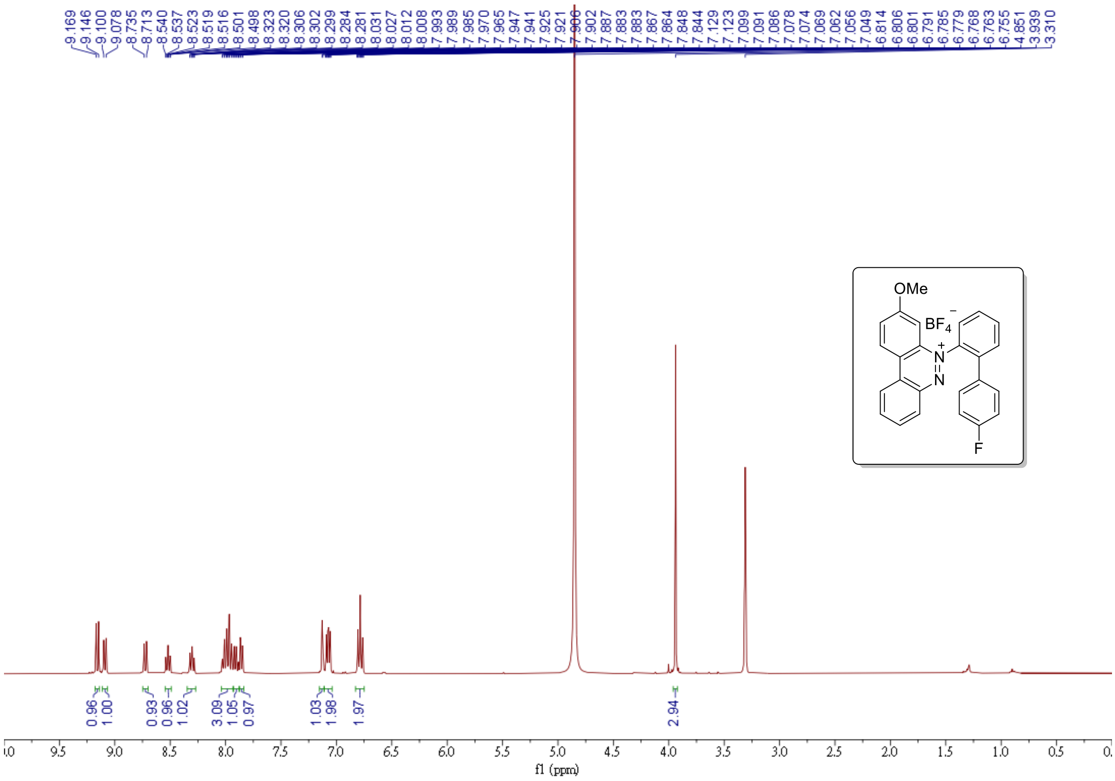

^{13}C NMR spectrum of compound **3p** (100 MHz, CD$_3$OD)

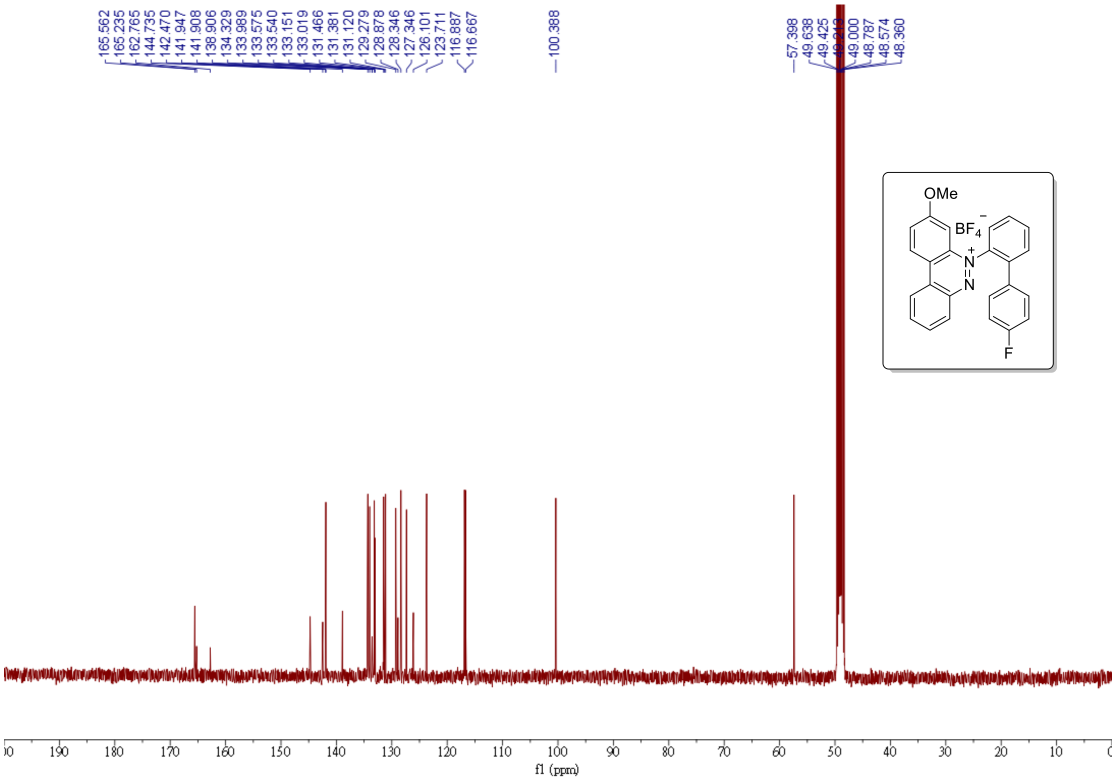

^{1}H NMR spectrum of compound **3r** (400 MHz, CD_3OD)

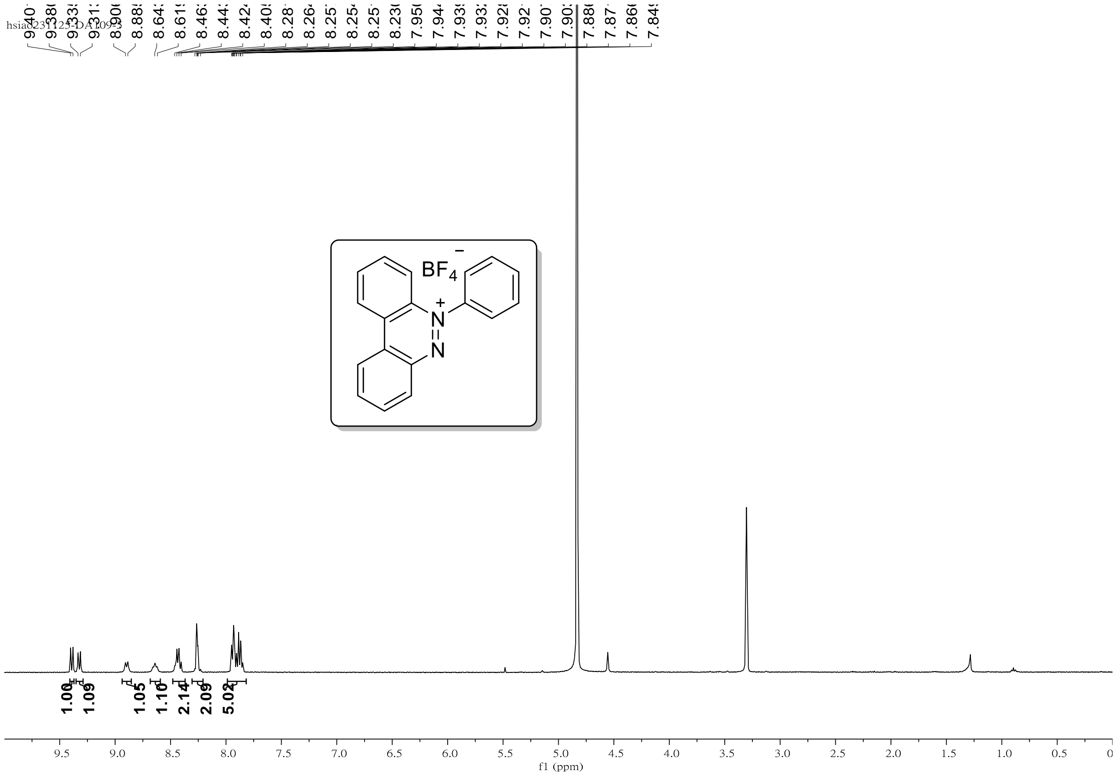

^{13}C NMR spectrum of compound **3r** (100 MHz, CD_3OD)

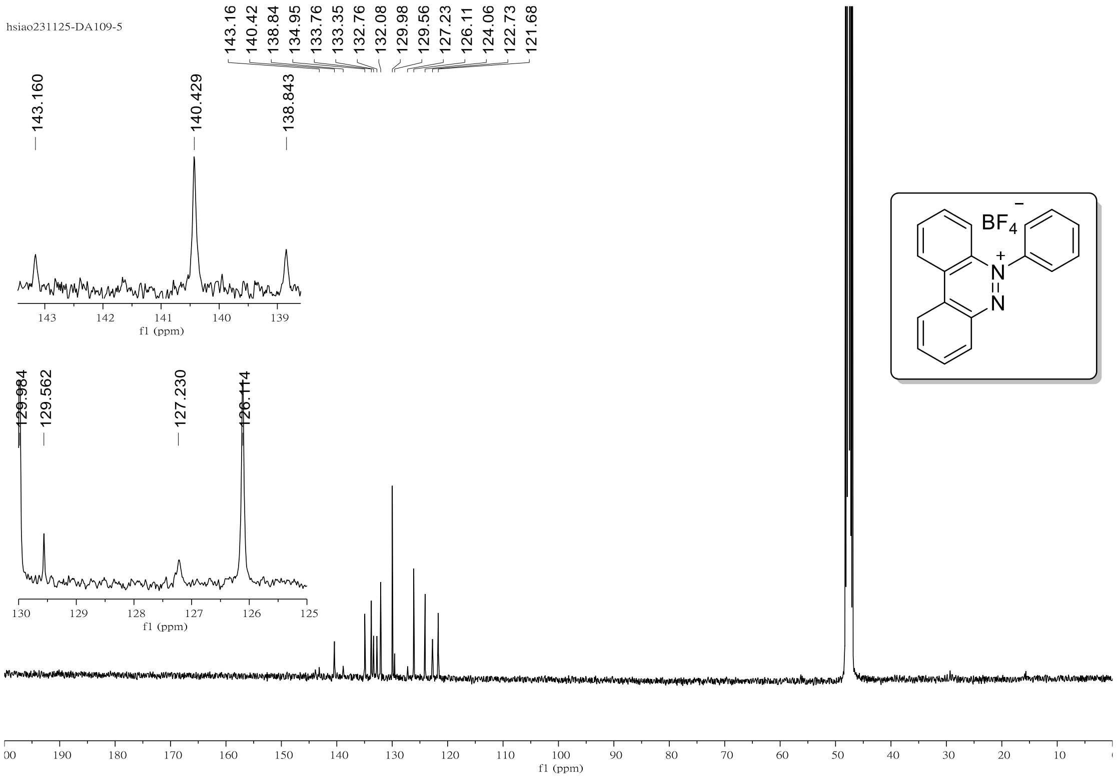

^{1}H NMR spectrum of compound **3s** (400 MHz, CD_3OD)

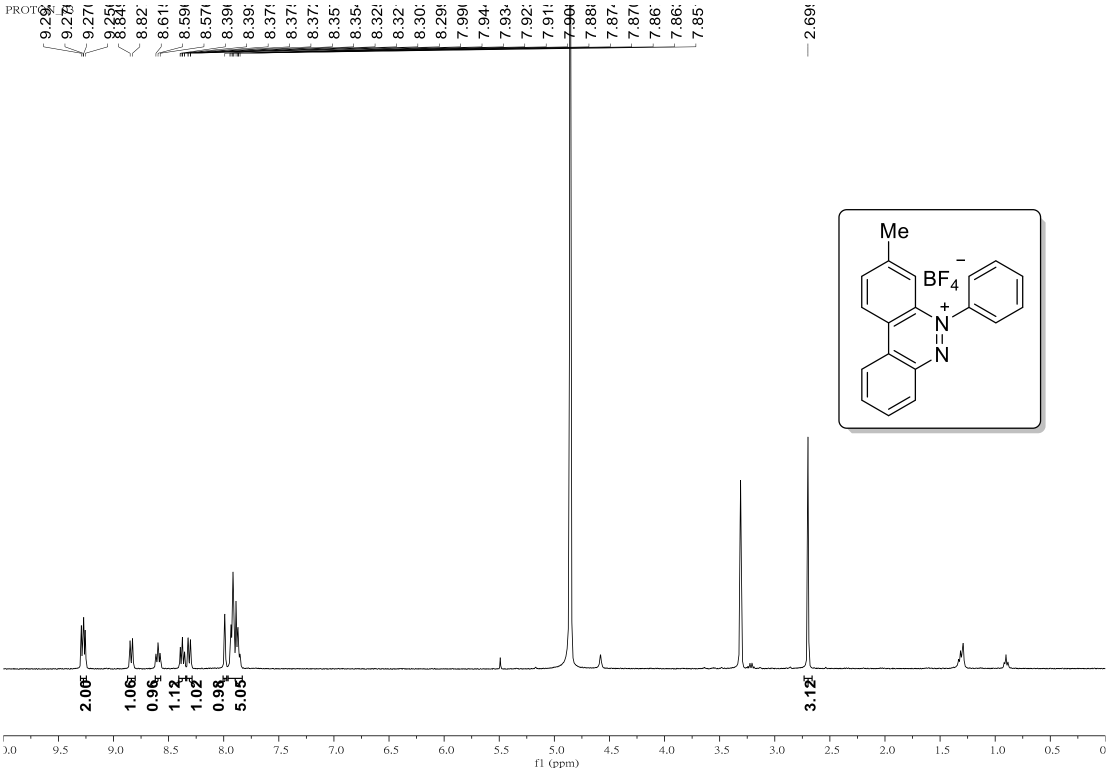

^{13}C NMR spectrum of compound **3s** (100 MHz, CD_3OD)

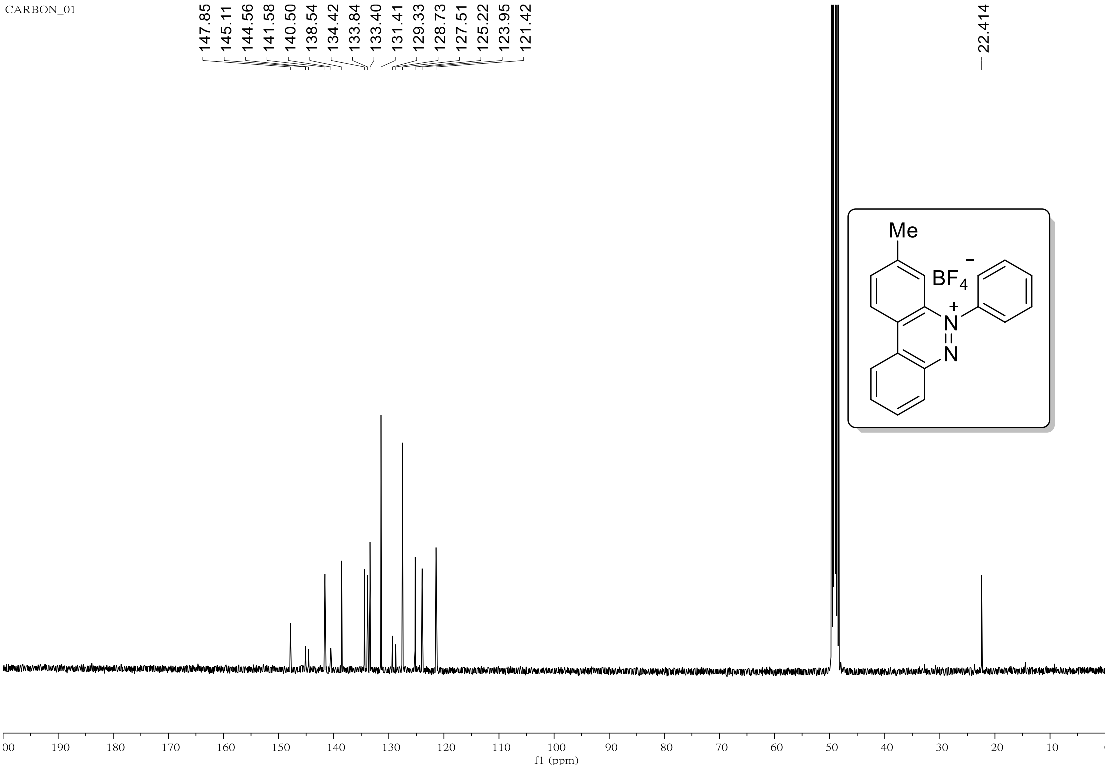

Cyclic voltammetry

CV measurements were conducted with a CHI 621C potentiostat and CHI Version 16.04 software. For all experiments a glassy carbon working electrode (3.0 mm diameter), a platinum wire counter electrode, and an Ag/Ag^{+} electrode was used as a reference electrode. The working electrode was polished with figure-eight motions on a cloth polishing pad in deionized water slurry before conducting experiments. The voltammograms were recorded at 23 ºC in DCM at a substrate concentration of 3.0 mM and with 0.1 M n-Bu_4NPF_6 as supporting electrolyte. An overpressure of nitrogen gas was maintained throughout the experiment. The CV measurement used 0 V as the initial potential and the direction of the reduction potential as the initial scanning direction. The scan rates were 100 mV/s.

Table S3. Cyclic voltammetry study of representative substrates **2a-b**, **2g** and **2q-s** in 0.1 M Bu_4NPF_6/DCM at 23 °C.

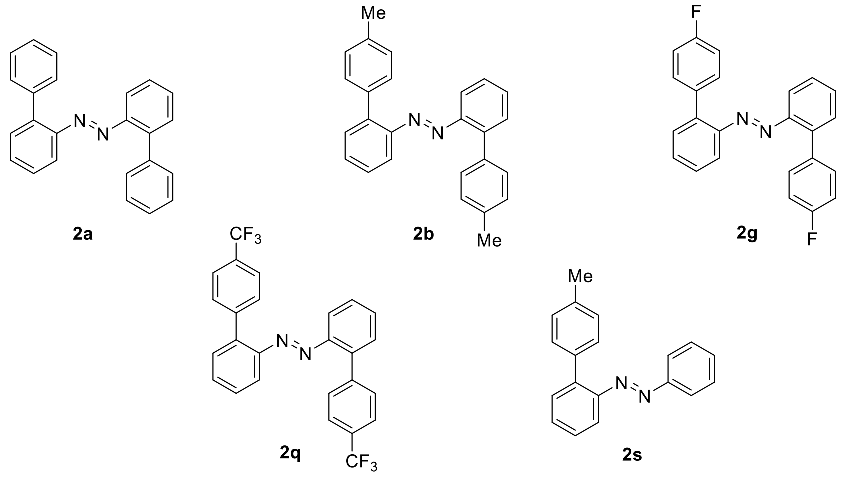

Compound	$E_{1/2}^{red}$	$E_{1/2}^{ox}$	LUMO (eV)[a]	HOMO (eV)[a]	$\Delta E_{HOMO–LUMO}$ (eV)[b]
**2a**	−1.872	1.16	−2.518	−5.55	3.03
**2b**	−1.868	1.08	−2.522	−5.47	2.94
**2g**	−1.48	1.19	−2.91	−5.58	2.67
**2q**	−1.73	1.30	−2.66	−5.69	3.03
**2s**	−1.57	1.22	−2.82	−5.61	2.79

[a]Reduction and oxidation half-wave potentials determined by CV measurement. [b]LUMO and HOMO were estimated by the equations: LUMO = −($E_{1/2}^{red}$ + 4.39), HOMO = −($E_{1/2}^{ox}$ + 4.39), respectively.

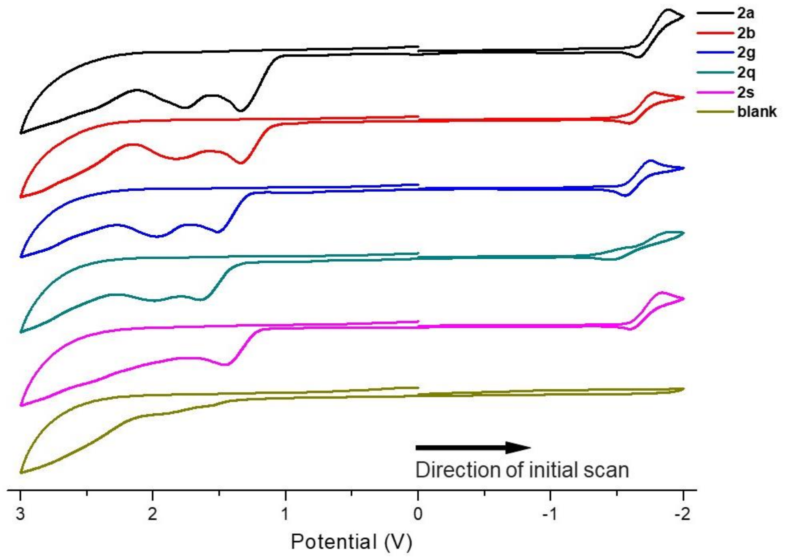

Figure S5. Cyclic voltammograms use the polarographic plotting convention. Cyclic voltammograms of **2a, 2b, 2g, 2q, 2s** and blank (3.0 mM) in 0.1 M Bu_4NPF_6/DCM at 23 °C started at 0 V. The scan rates were 100 mV/s.

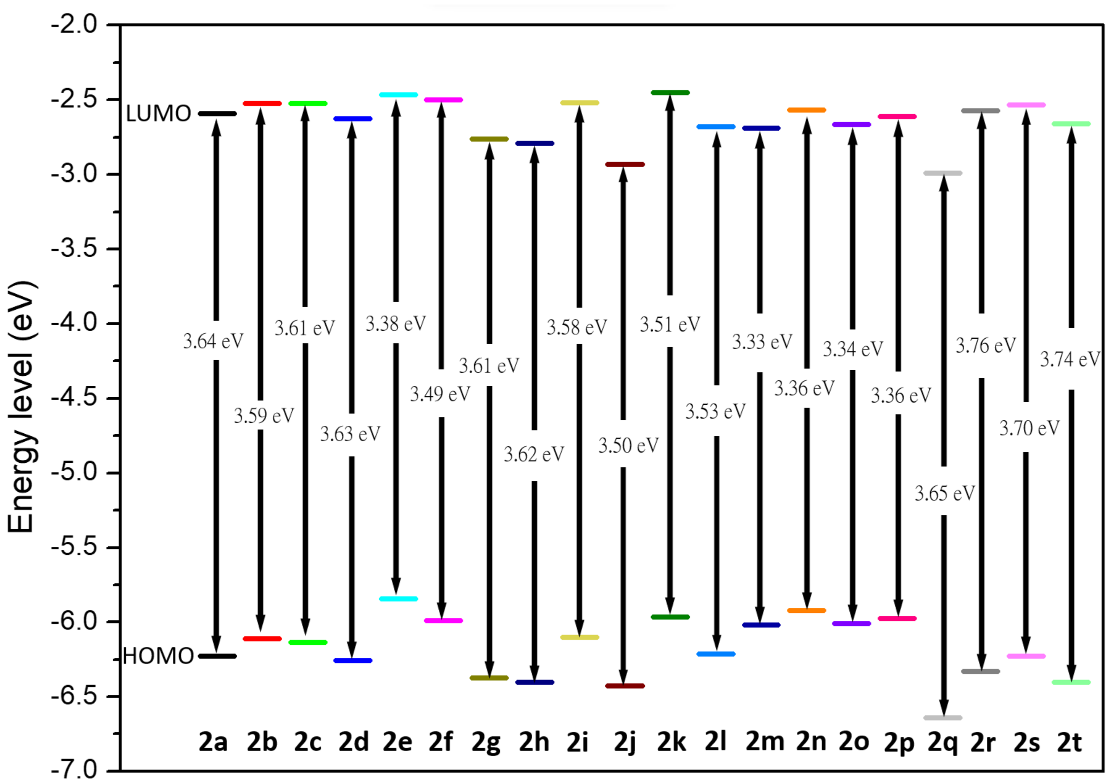

Figure S6a. Computed HOMO and LUMO energy levels of **2a-2t**.

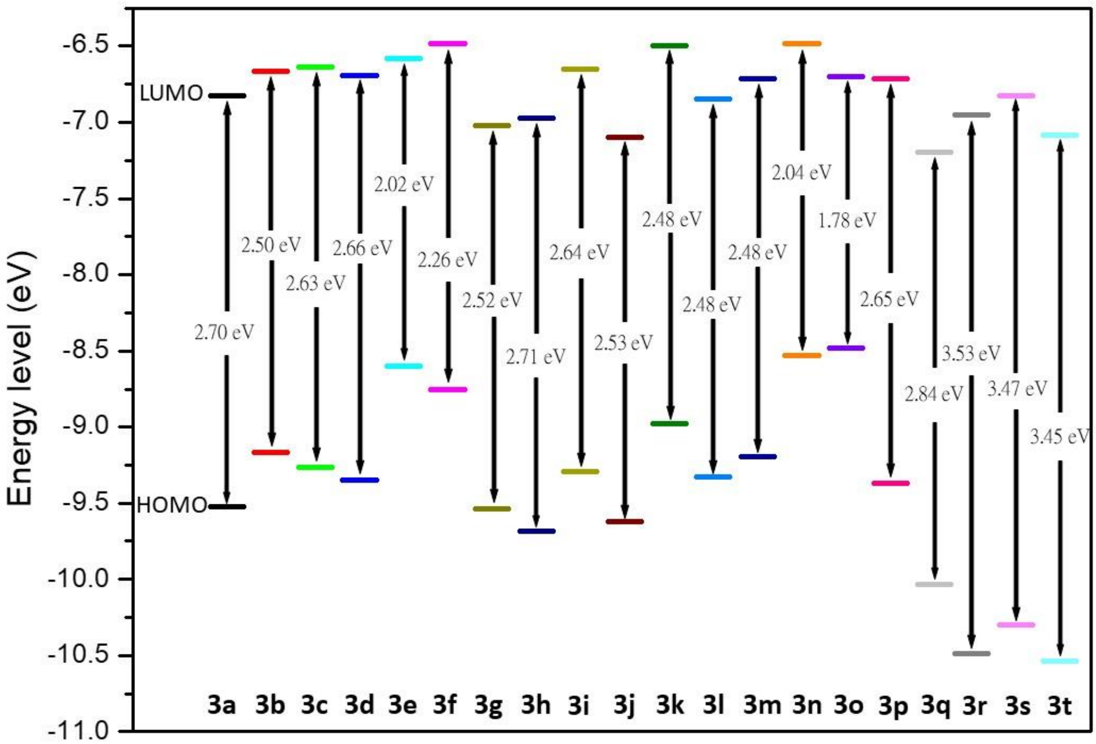

Figure S6b. Computed HOMO and LUMO energy levels of **3a-3t cations**.

Computed UV-vis spectra, MOs, and energy levels of 3a-3t cation[6]

(a)

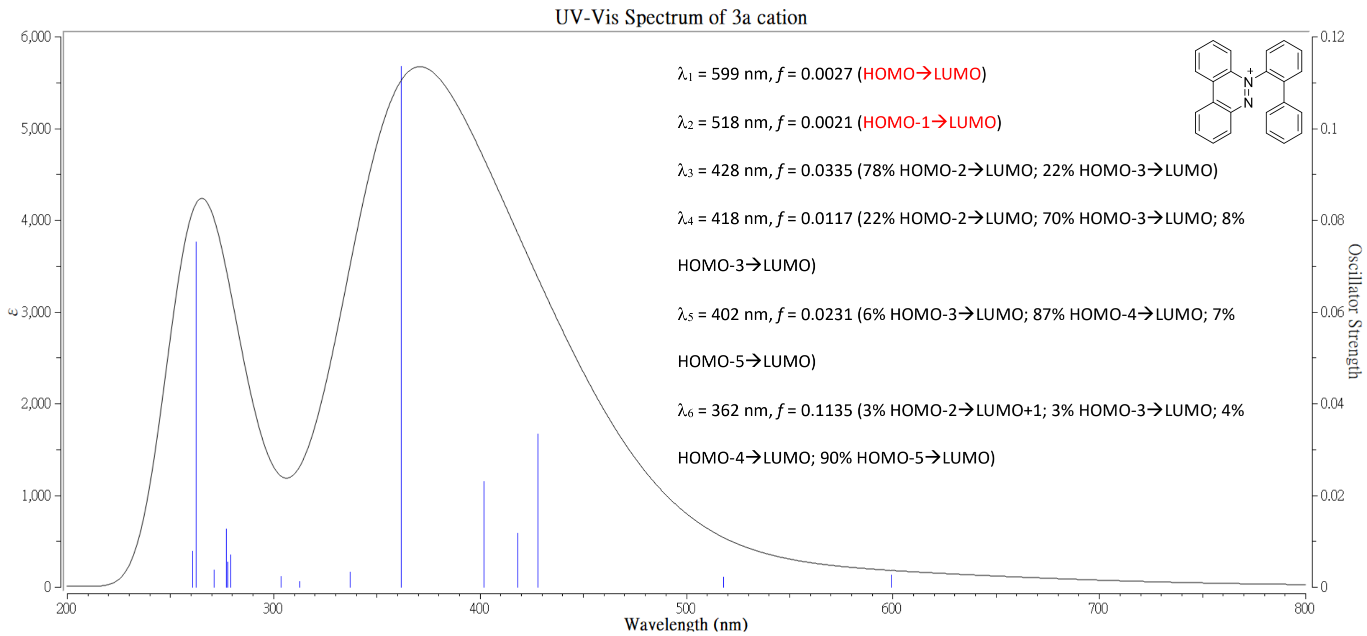

(b)

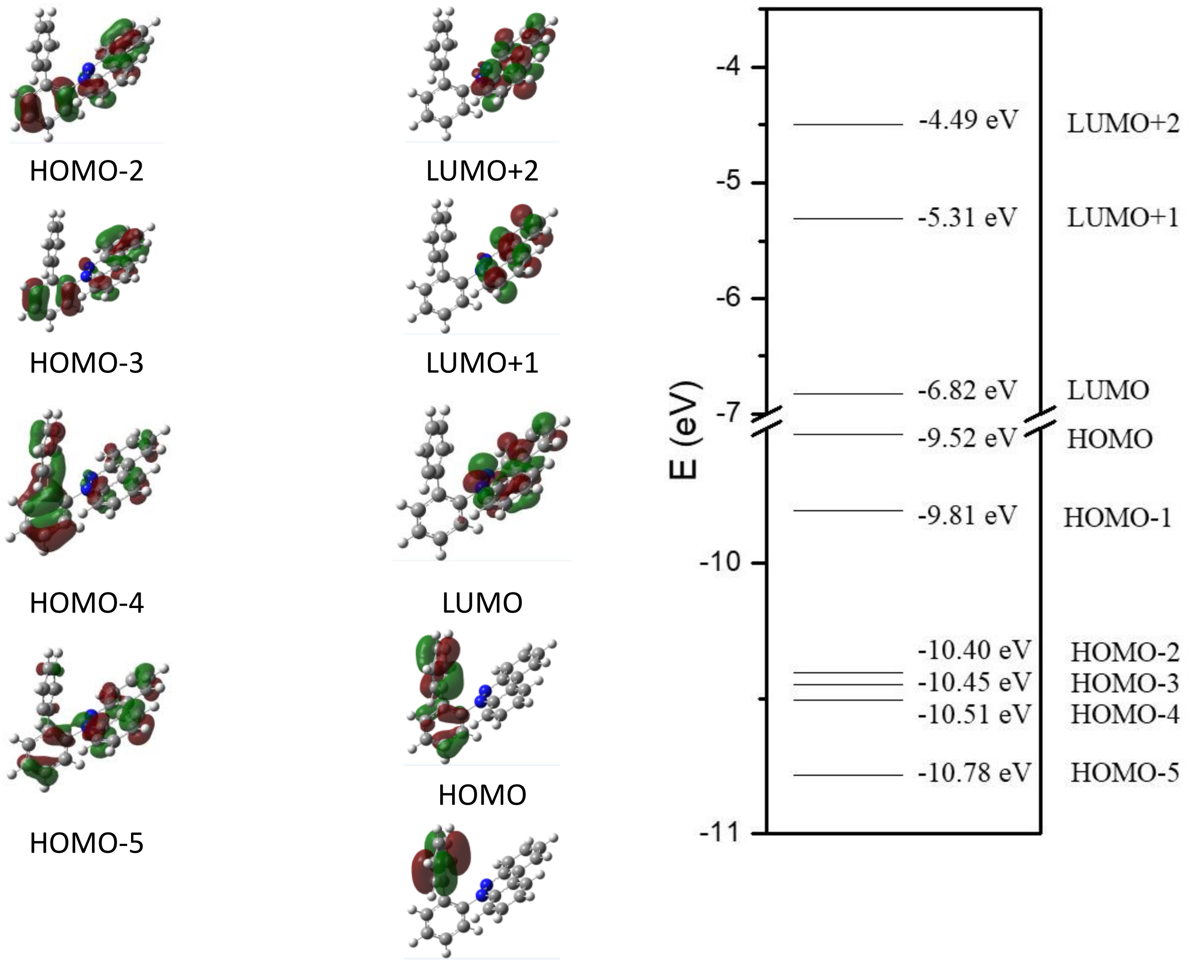

Figure S7. (a) Absorption spectrum of **3a** cation and oscillator strength calculated at B3LYP/6-311G++(2df, 2p) level basis set. (b) MOs of HOMO, HOMO−1, HOMO−2, HOMO−3, HOMO−4, HOMO−5, LUMO, LUMO+1, LUMO+2 (iso value = 0.04) and their corresponding energy levels.

(a)

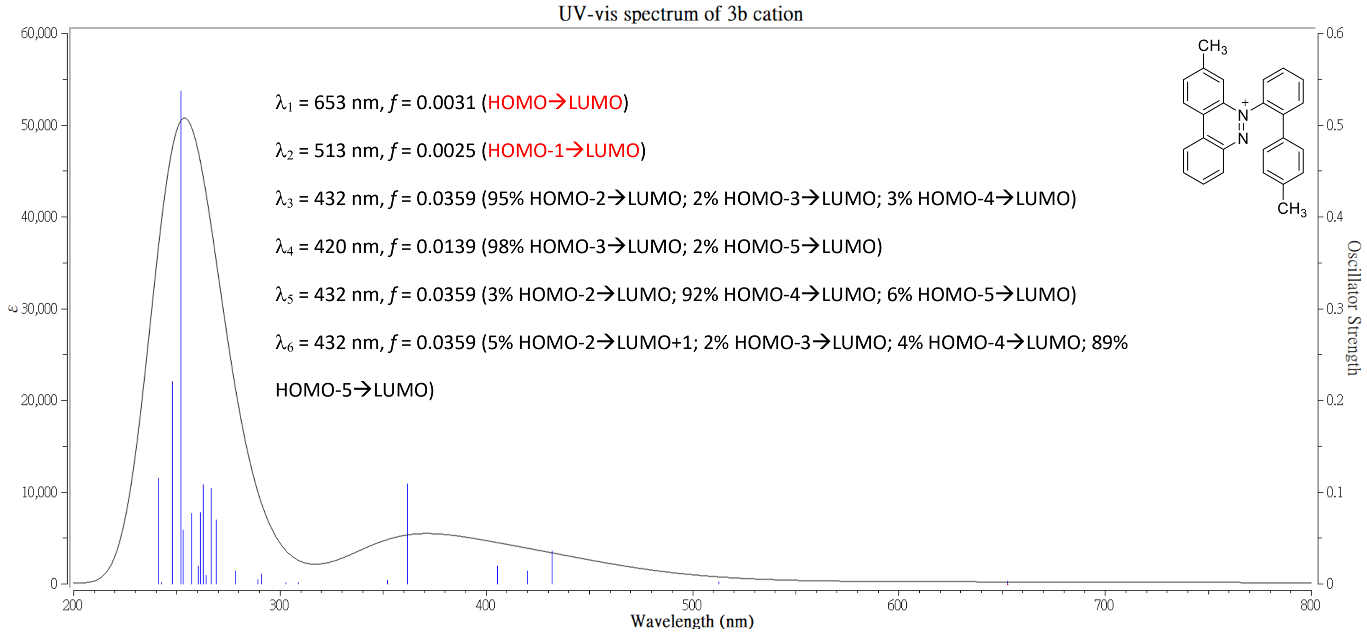

(b)

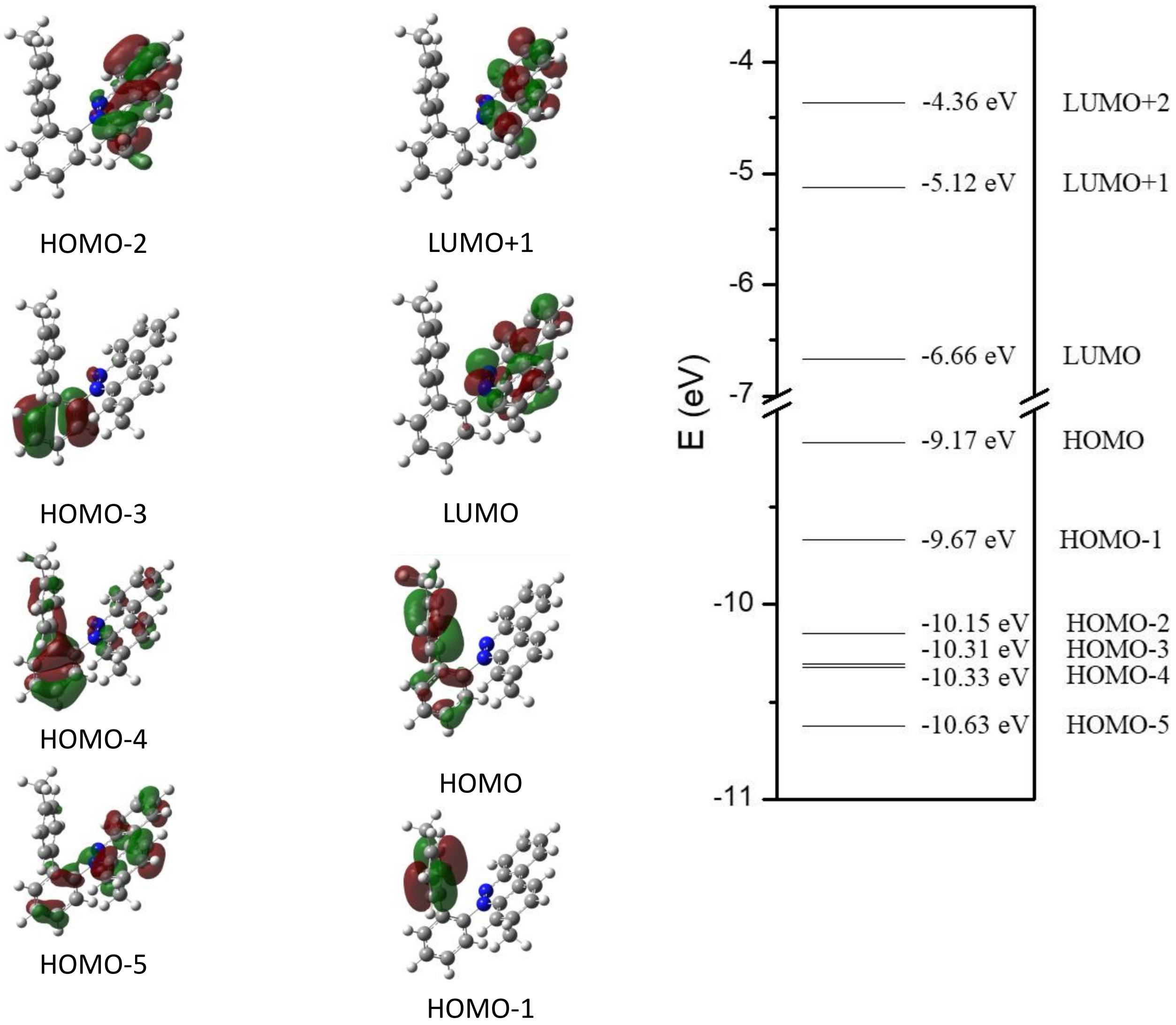

Figure S8. (a) Absorption spectrum of **3b** cation and oscillator strength calculated at B3LYP/6-311G++(2df, 2p) level basis set. (b) MOs of HOMO, HOMO−1, HOMO−2, HOMO−3, HOMO−4, HOMO−5, LUMO, LUMO+1 (iso value = 0.04) and their corresponding energy levels.

(a)

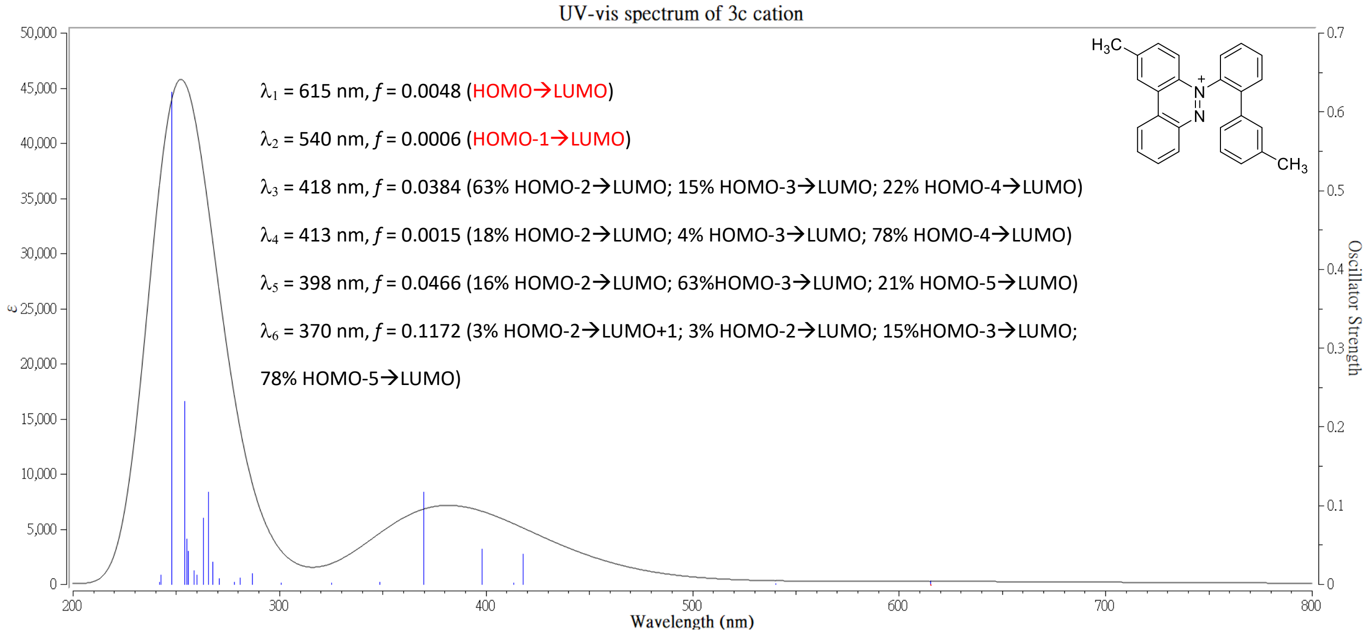

(b)

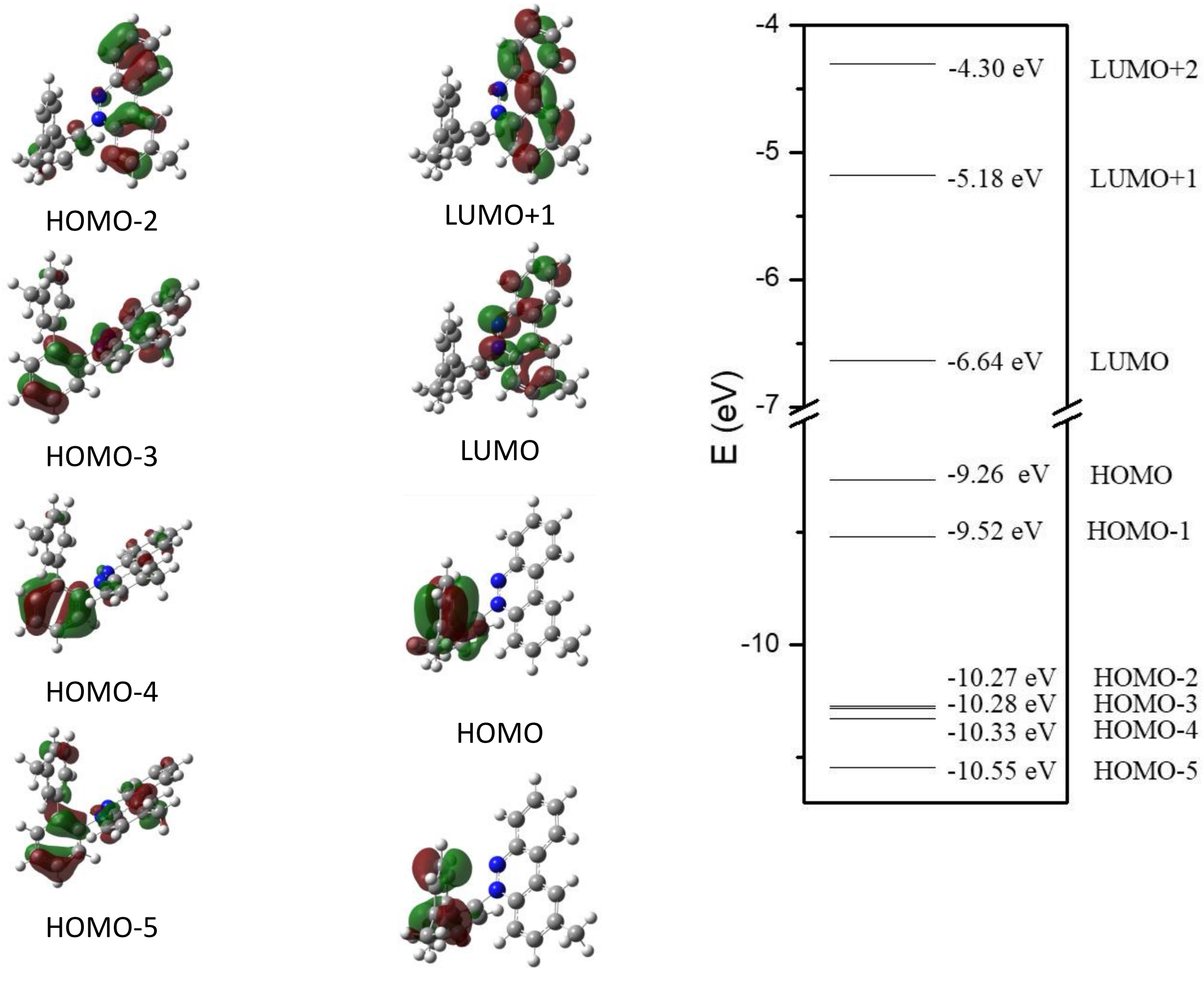

Figure S9. (a) Absorption spectrum of **3c** cation and oscillator strength calculated at B3LYP/6-311G++(2df, 2p) level basis set. (b) MOs of HOMO, HOMO−1, HOMO−2, HOMO−3, HOMO−4, HOMO−5, LUMO, LUMO+1 (iso value = 0.04) and their corresponding energy levels.

(a)

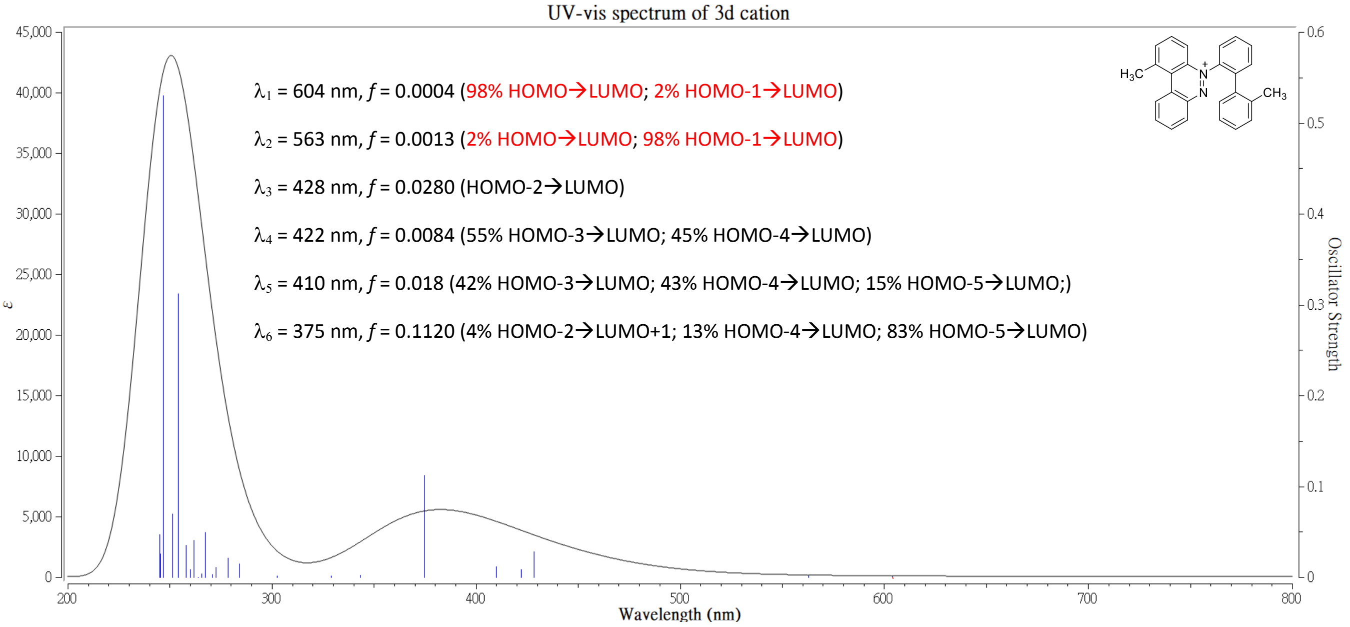

(b)

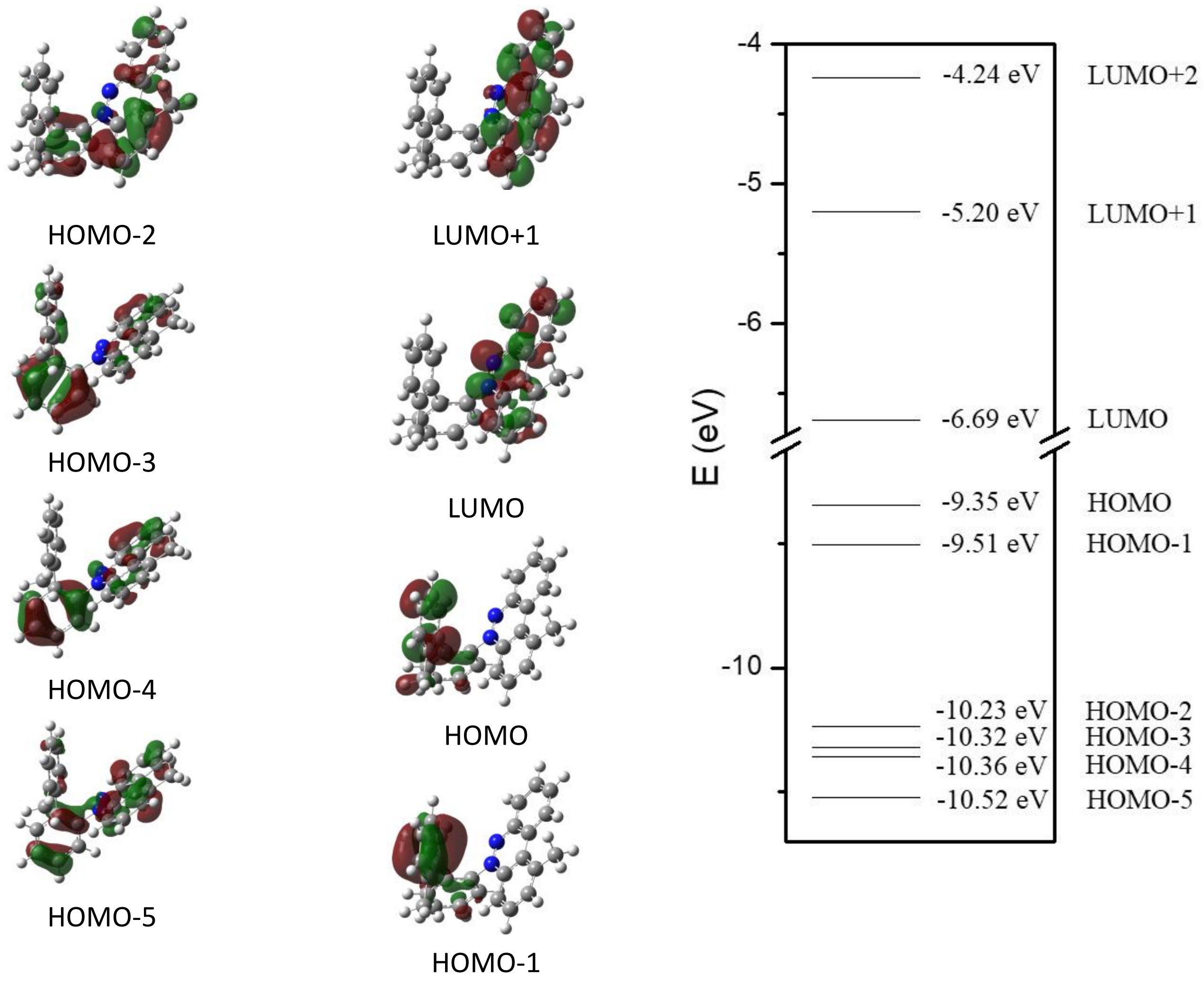

Figure S10. (a) Absorption spectrum of **3d** cation and oscillator strength calculated at B3LYP/6-311G++(2df, 2p) level basis set. (b) MOs of HOMO, HOMO−1, HOMO−2, HOMO−3, HOMO−4, HOMO−5, LUMO, LUMO+1 (iso value = 0.04) and their corresponding energy levels.

(a)

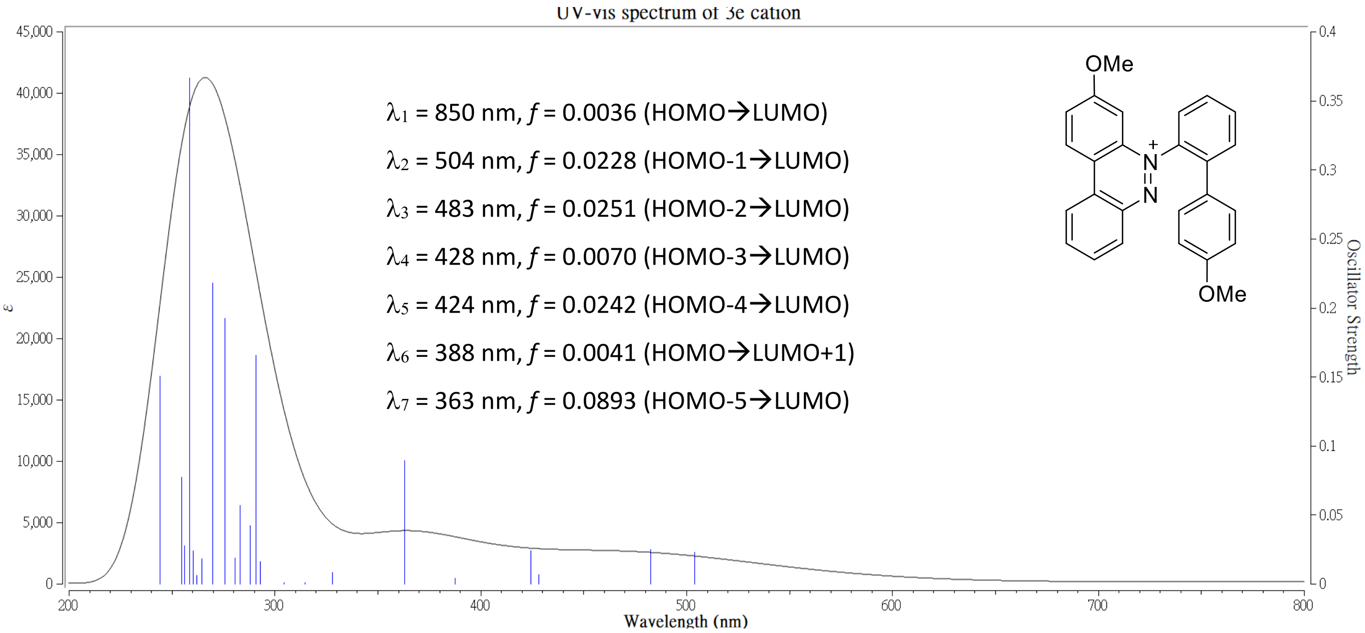

(b)

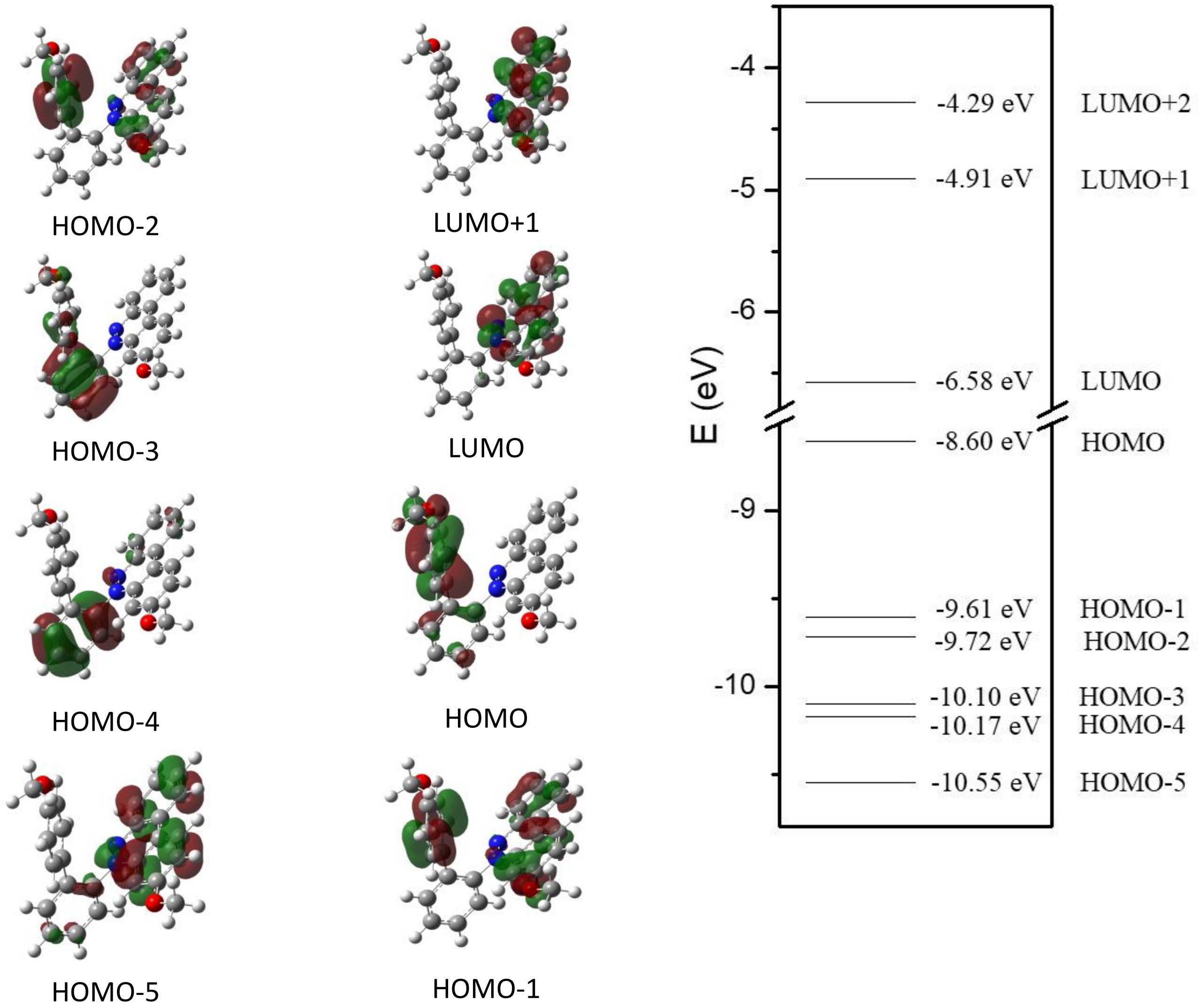

Figure S11. (a) Absorption spectrum of **3e** cation and oscillator strength calculated at B3LYP/6-311G++(2df, 2p) level basis set. (b) MOs of HOMO, HOMO−1, HOMO−2, HOMO−3, HOMO−4, HOMO−5, LUMO, LUMO+1 (iso value = 0.04) and their corresponding energy levels.

(a)

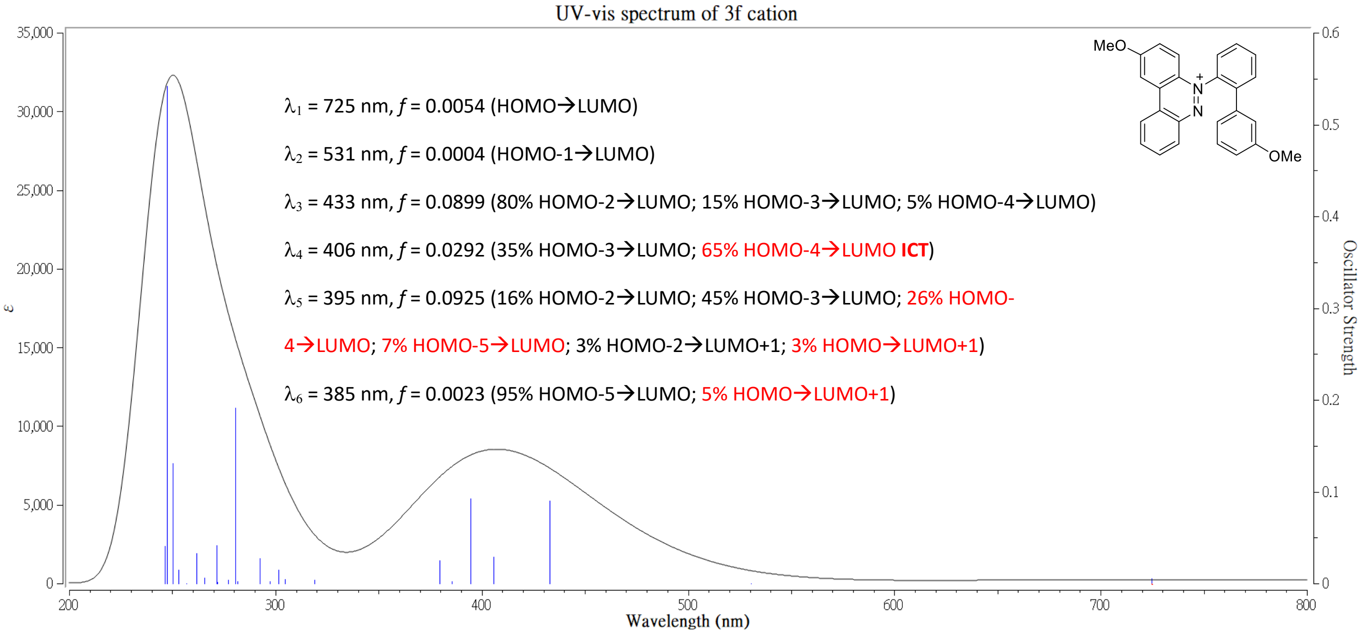

(b)

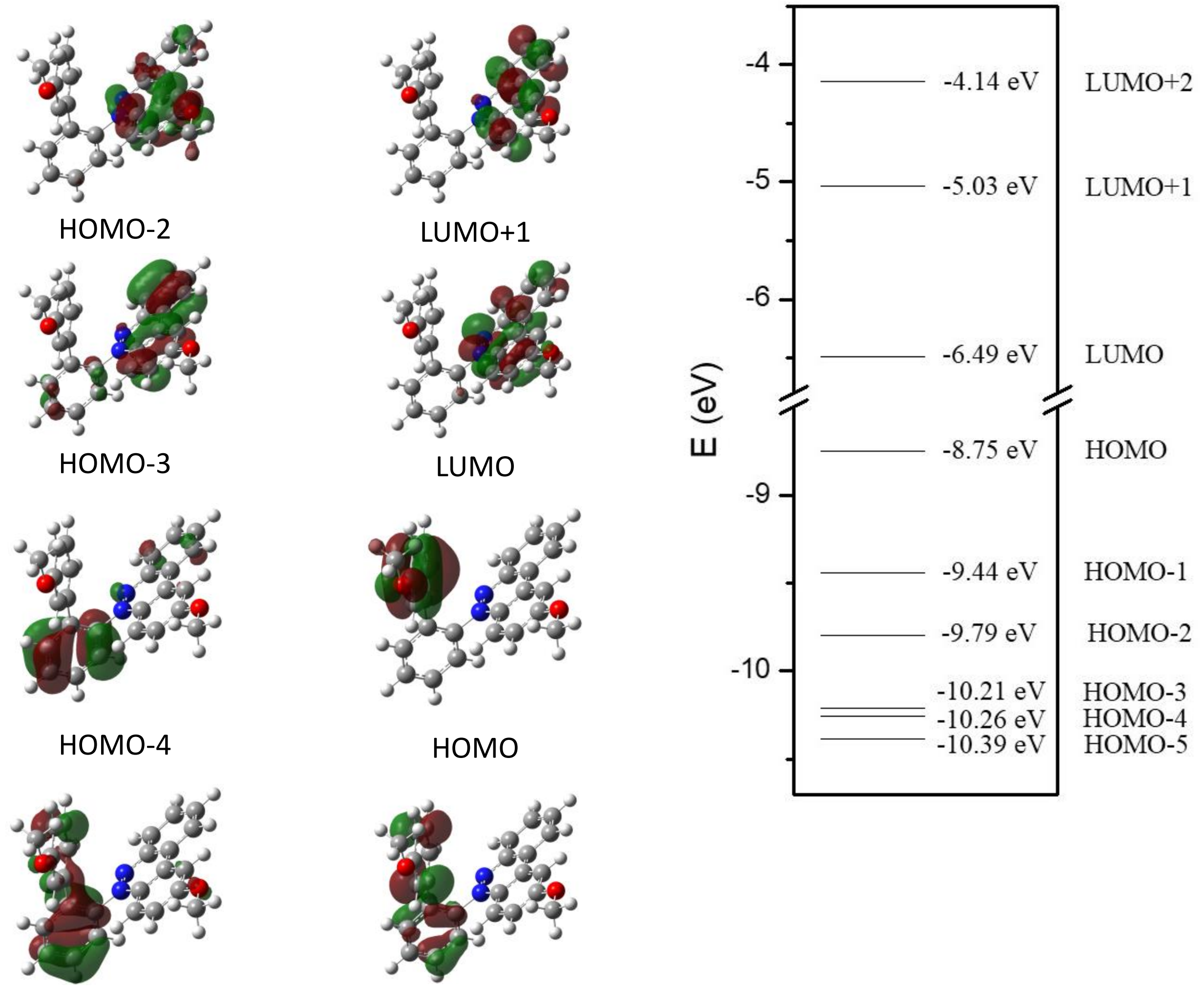

Figure S12. (a) Absorption spectrum of **3f** cation and oscillator strength calculated at B3LYP/6-311G++(2df, 2p) level basis set. (b) MOs of HOMO, HOMO−1, HOMO−2, HOMO−3, HOMO−4, HOMO−5, LUMO, LUMO+1 (iso value = 0.04) and their corresponding energy levels.

(a)

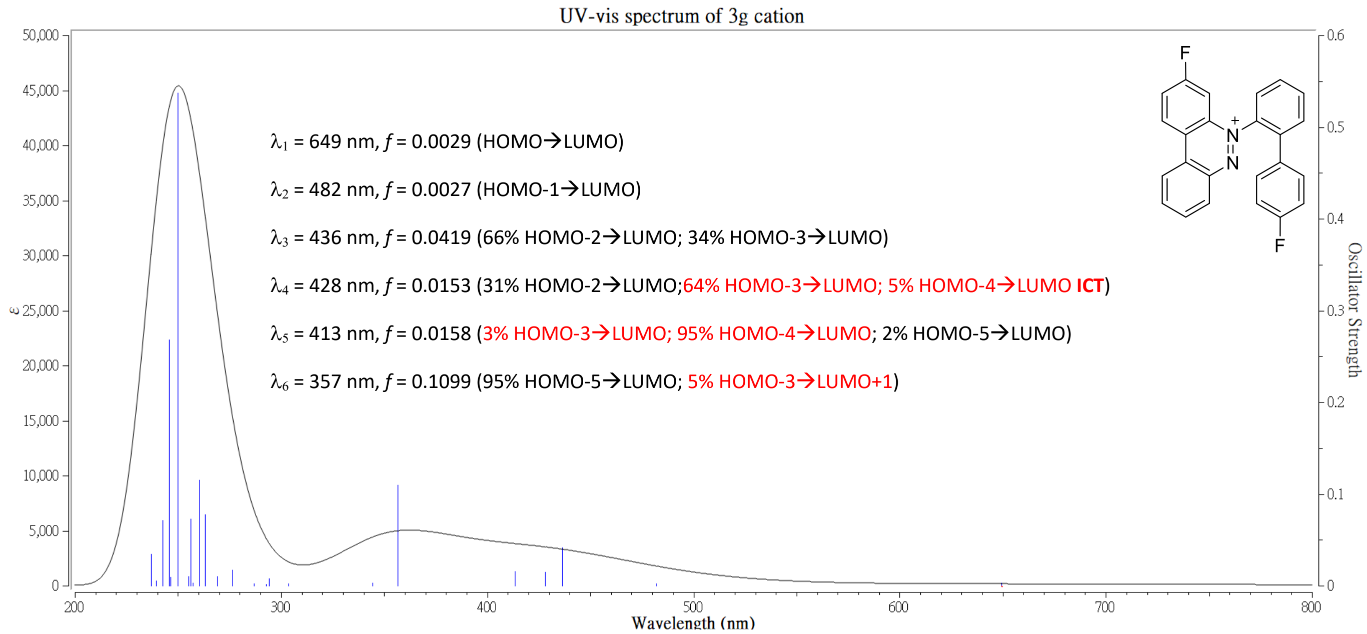

(b)

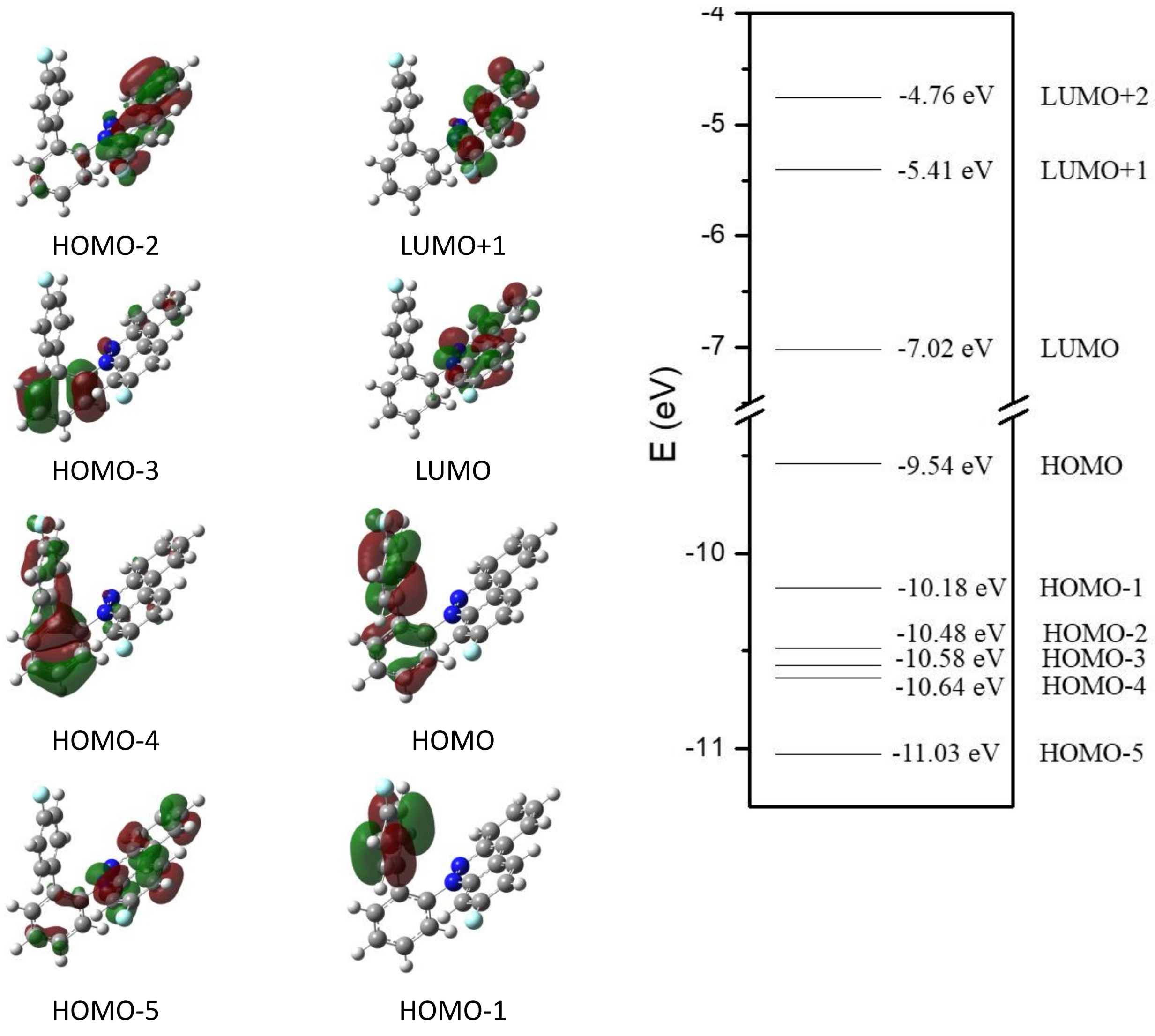

Figure S13. (a) Absorption spectrum of **3g** cation and oscillator strength calculated at B3LYP/6-311G++(2df, 2p) level basis set. (b) MOs of HOMO, HOMO−1, HOMO−2, HOMO−3, HOMO−4, HOMO−5, LUMO, LUMO+1 (iso value = 0.04) and their corresponding energy levels.

(a)

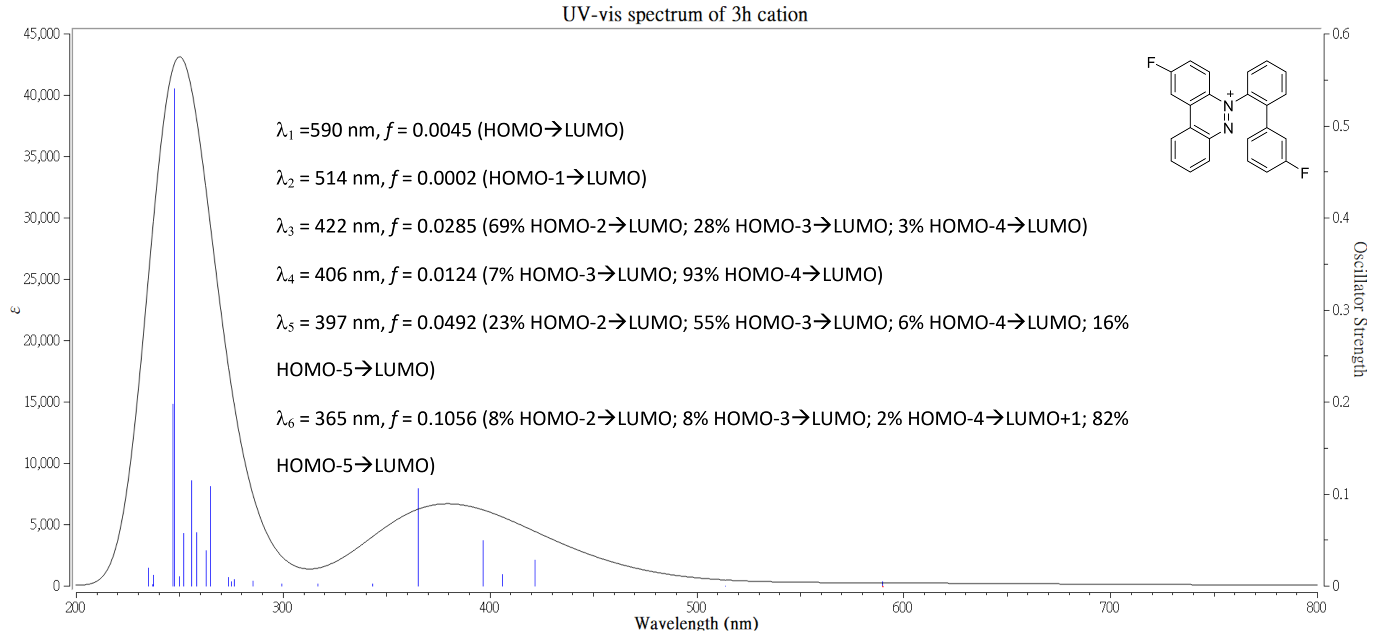

(b)

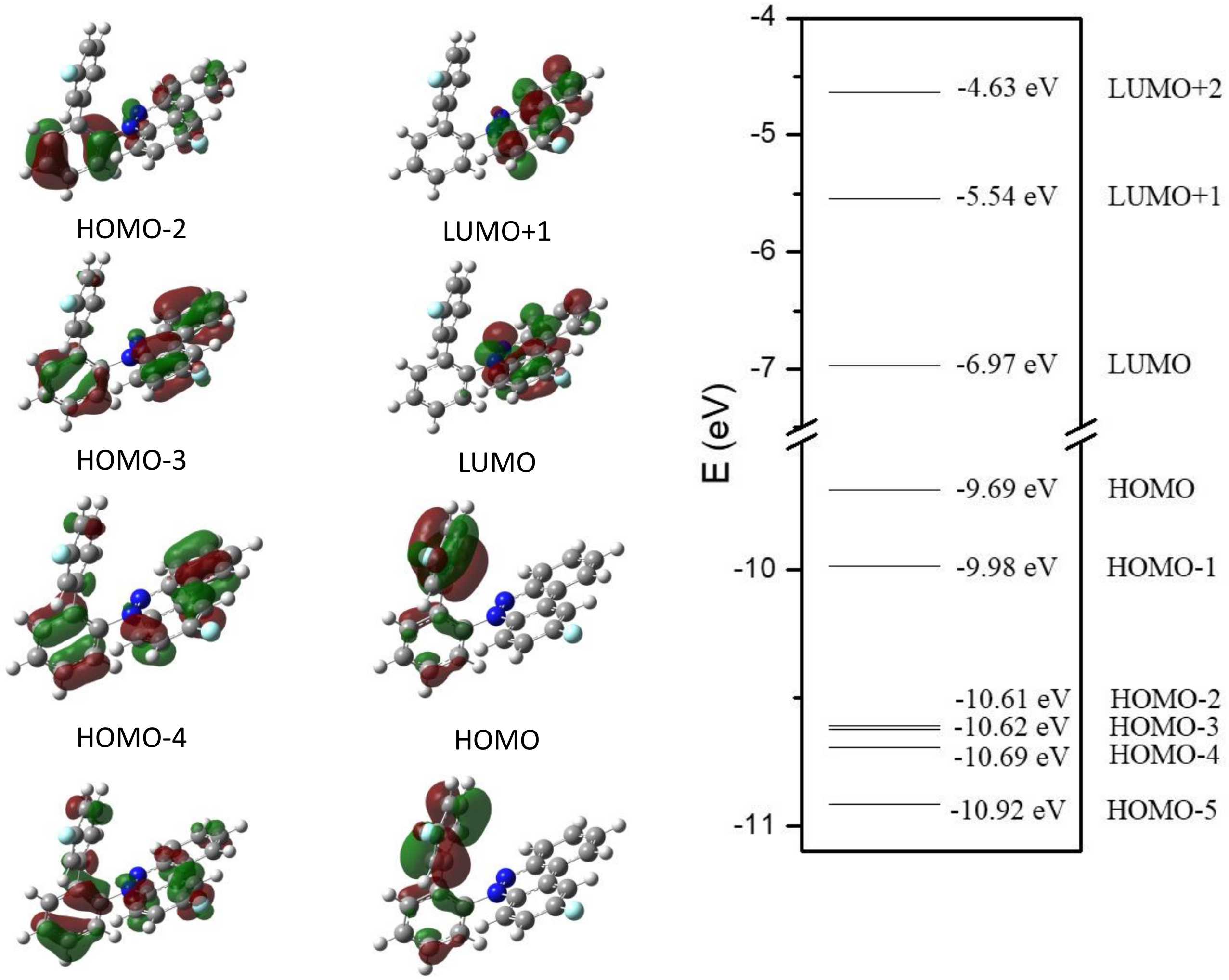

Figure S14. (a) Absorption spectrum of **3h** cation and oscillator strength calculated at B3LYP/6-311G++(2df, 2p) level basis set. (b) MOs of HOMO, HOMO−1, HOMO−2, HOMO−3, HOMO−4, HOMO−5, LUMO, LUMO+1 (iso value = 0.04) and their corresponding energy levels.

(a)

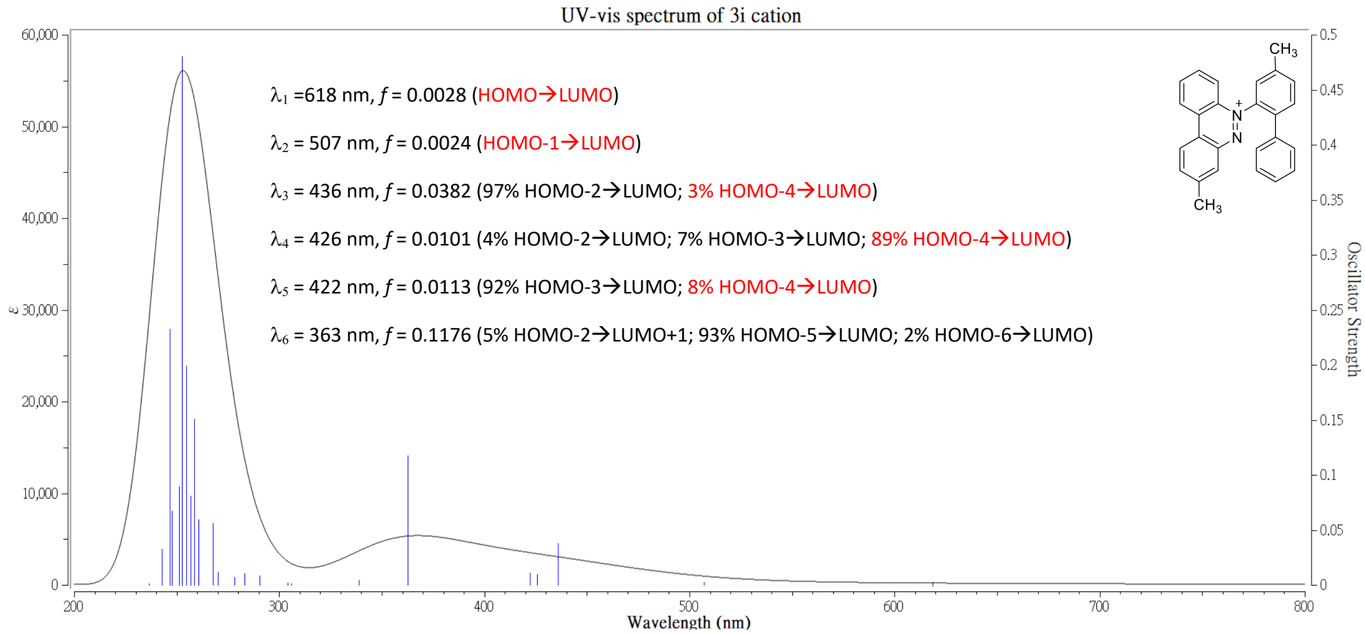

(b)

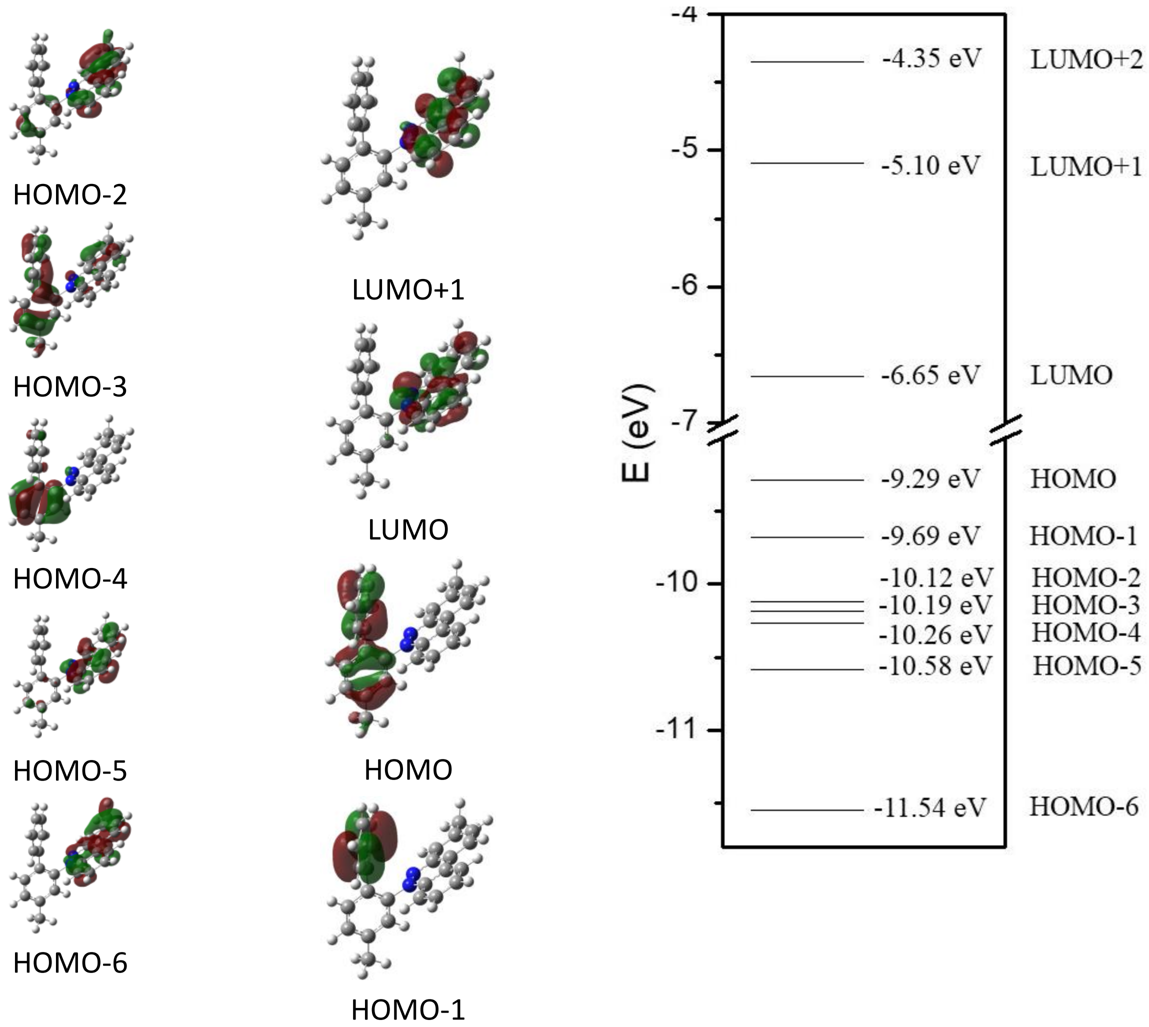

Figure S15. (a) Absorption spectrum of **3i** cation and oscillator strength calculated at B3LYP/6-311G++(2df, 2p) level basis set. (b) MOs of HOMO, HOMO−1, HOMO−2, HOMO−3, HOMO−4, HOMO−5, HOMO−6, LUMO, LUMO+1 (iso value = 0.04) and their corresponding energy levels.

(a)

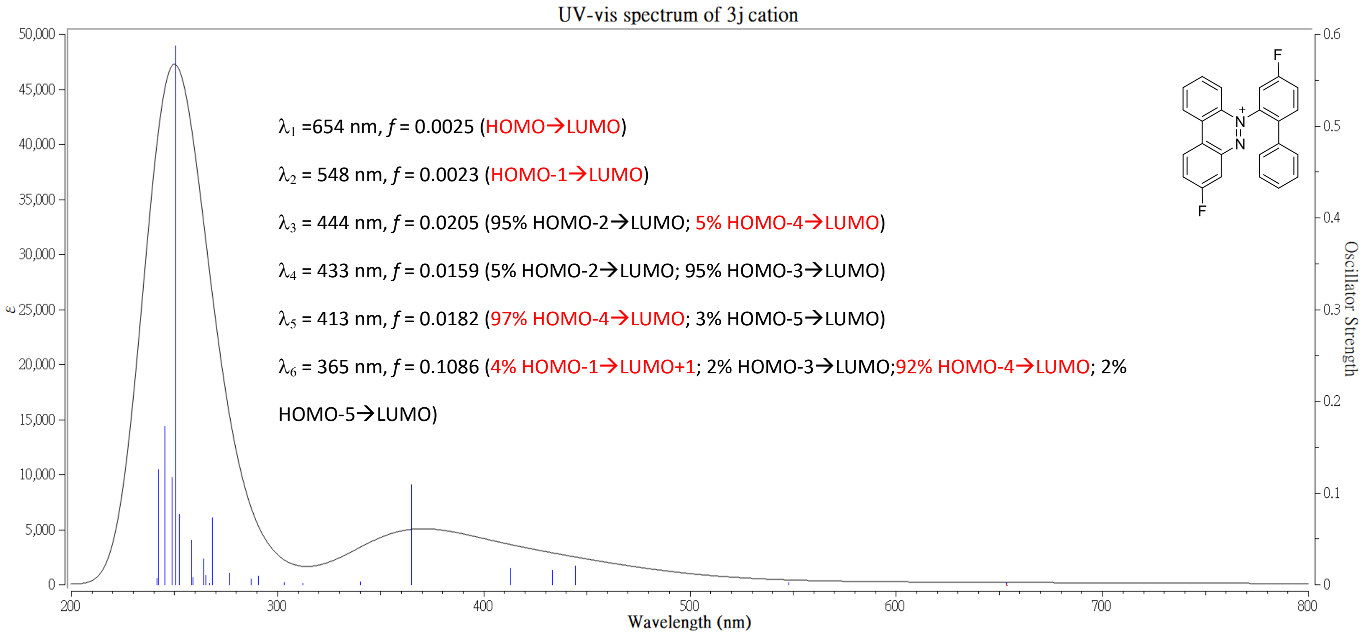

(b)

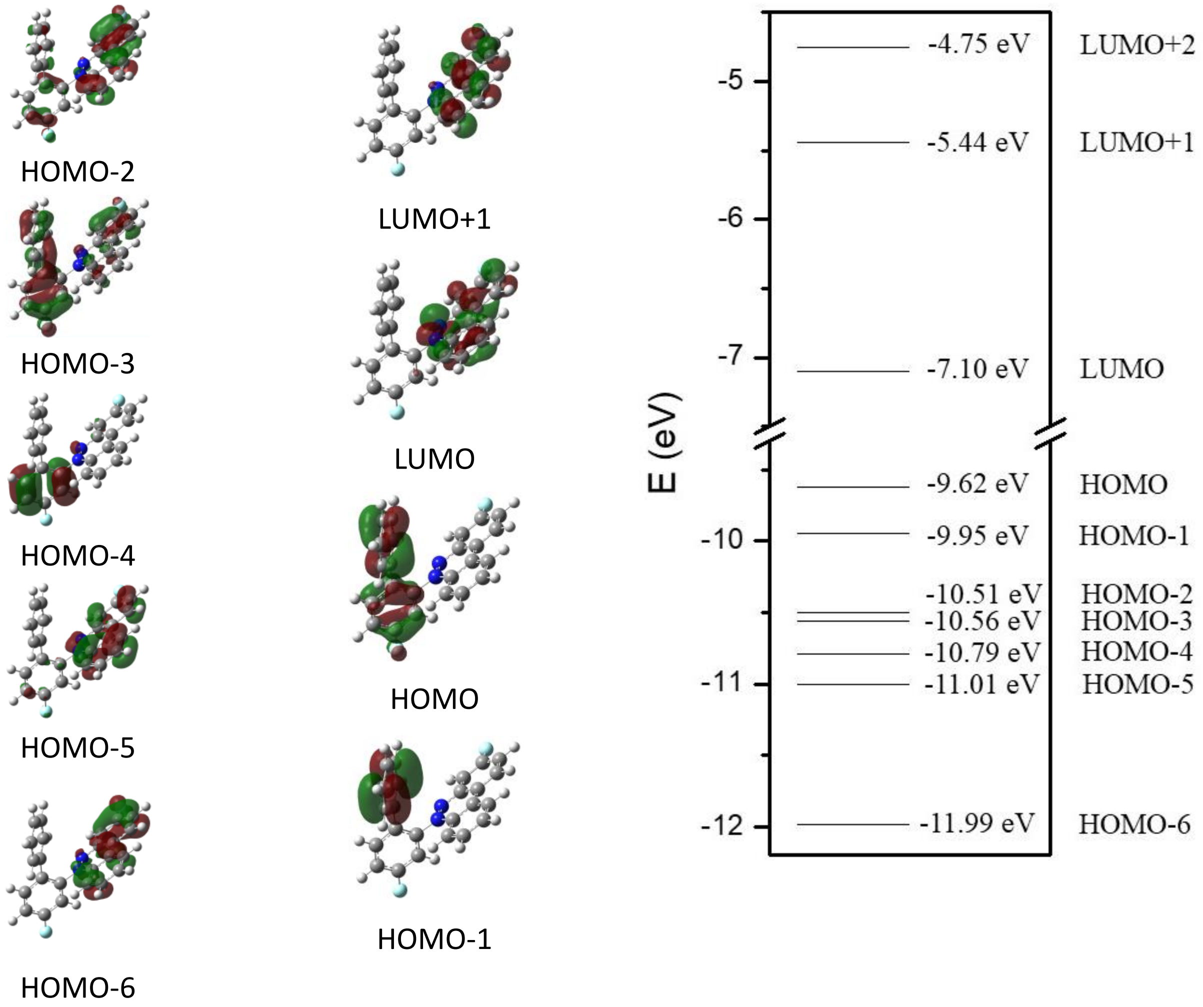

Figure S16. (a) Absorption spectrum of **3j** cation and oscillator strength calculated at B3LYP/6-311G++(2df, 2p) level basis set. (b) MOs of HOMO, HOMO−1, HOMO−2, HOMO−3, HOMO−4, HOMO−5, HOMO−6, LUMO, LUMO+1 (iso value = 0.04) and their corresponding energy levels.

(a)

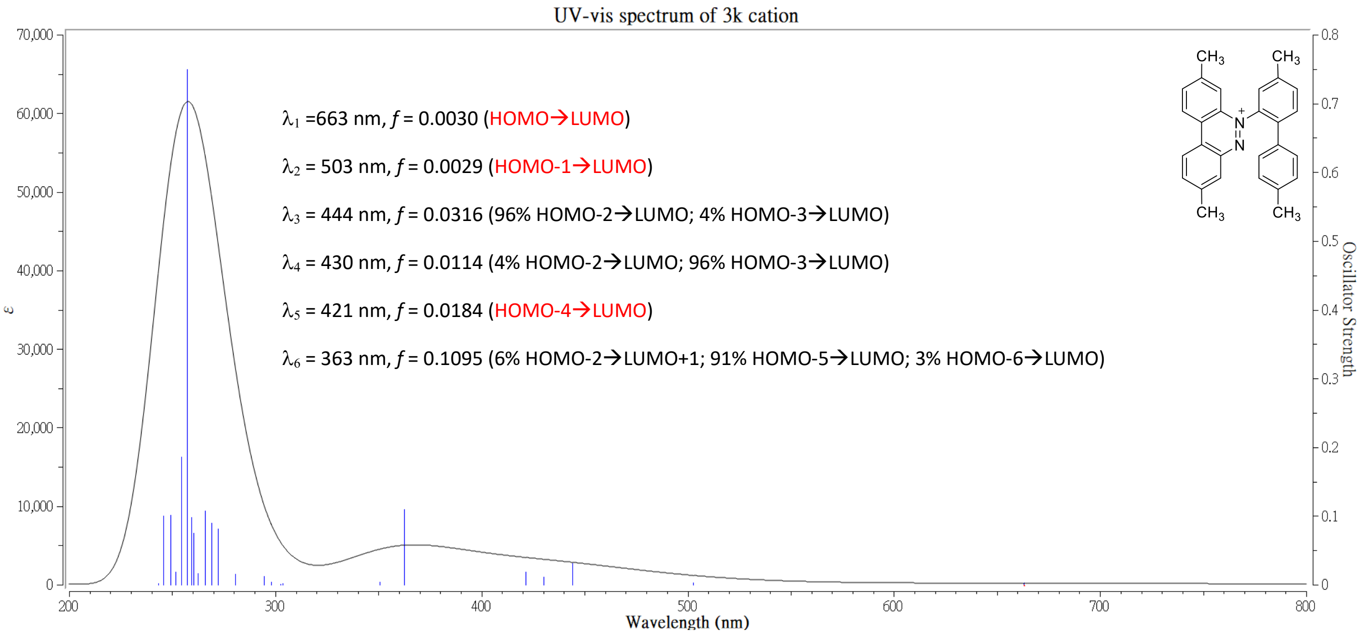

(b)

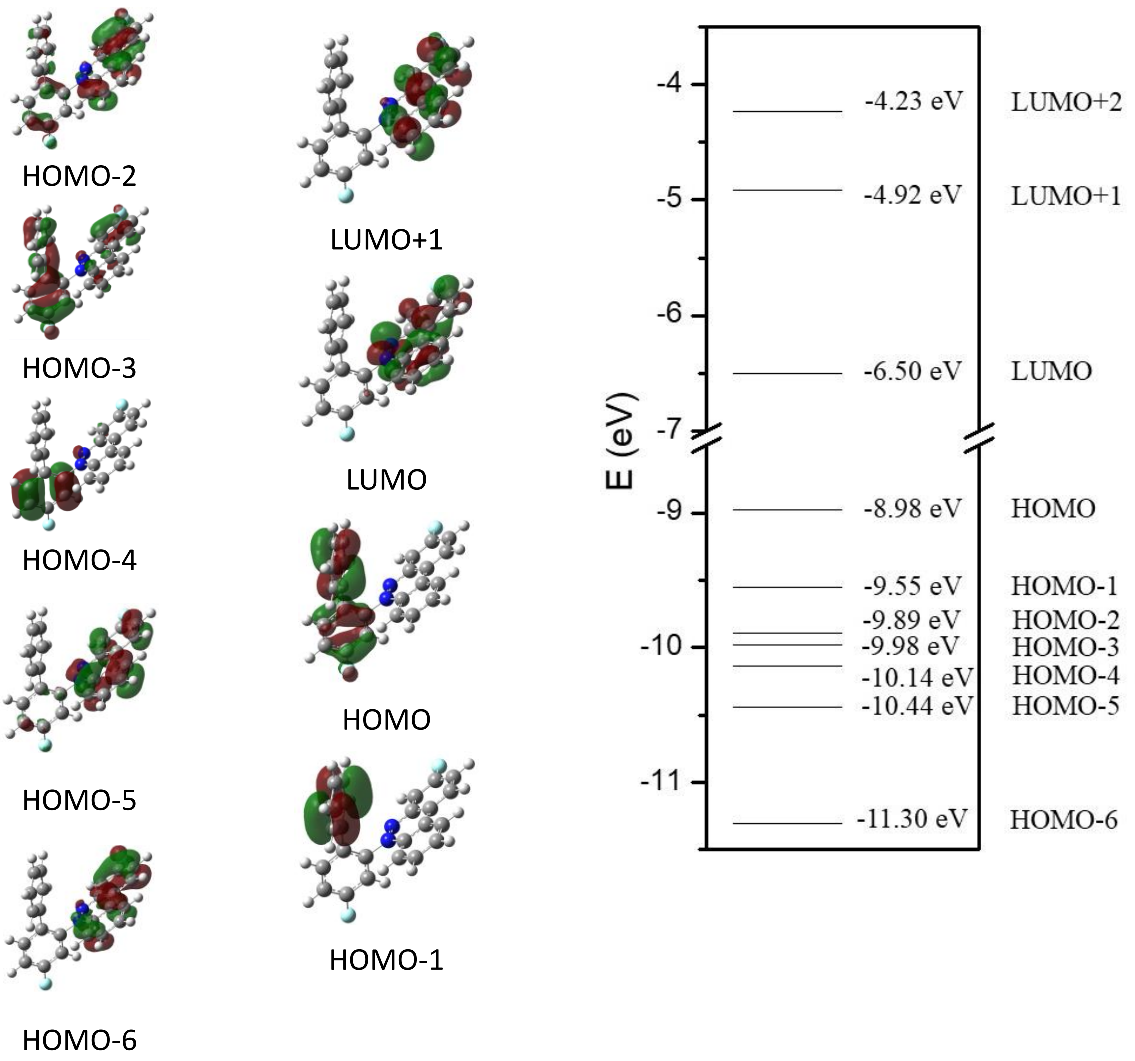

Figure S17. (a) Absorption spectrum of **3k** cation and oscillator strength calculated at B3LYP/6-311G++(2df, 2p) level basis set. (b) MOs of HOMO, HOMO−1, HOMO−2, HOMO−3, HOMO−4, HOMO−5, HOMO−6, LUMO, LUMO+1 (iso value = 0.04) and their corresponding energy levels.

(a)

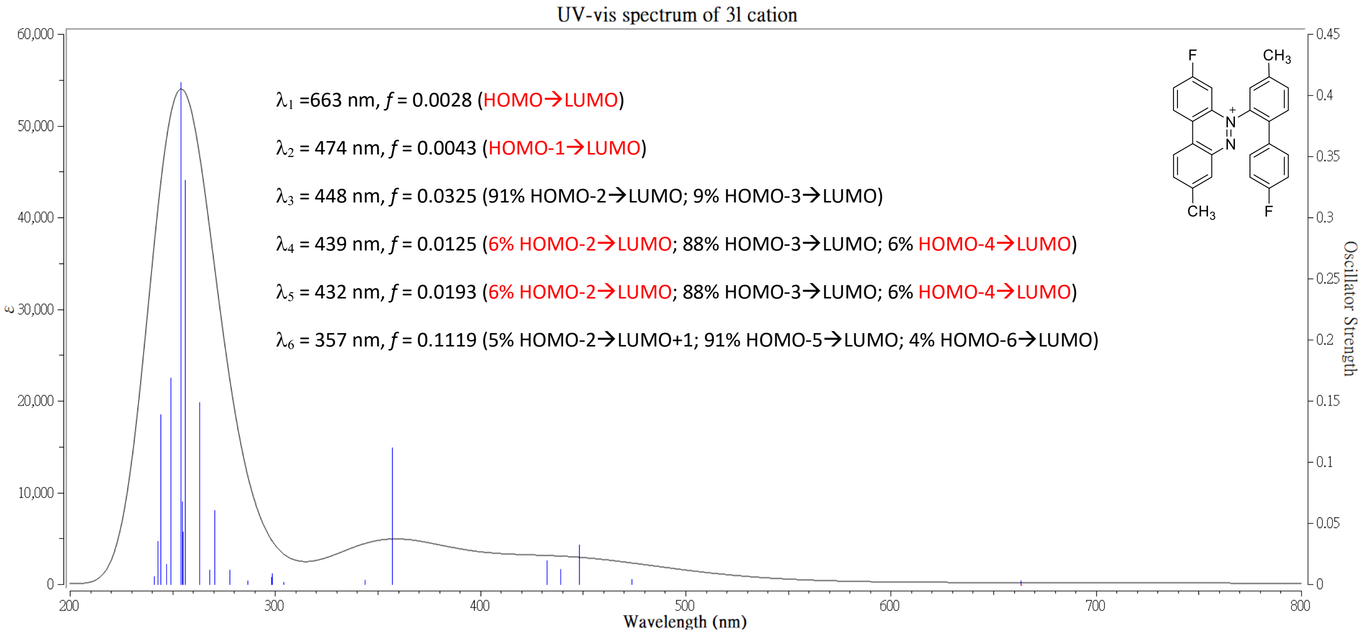

(b)

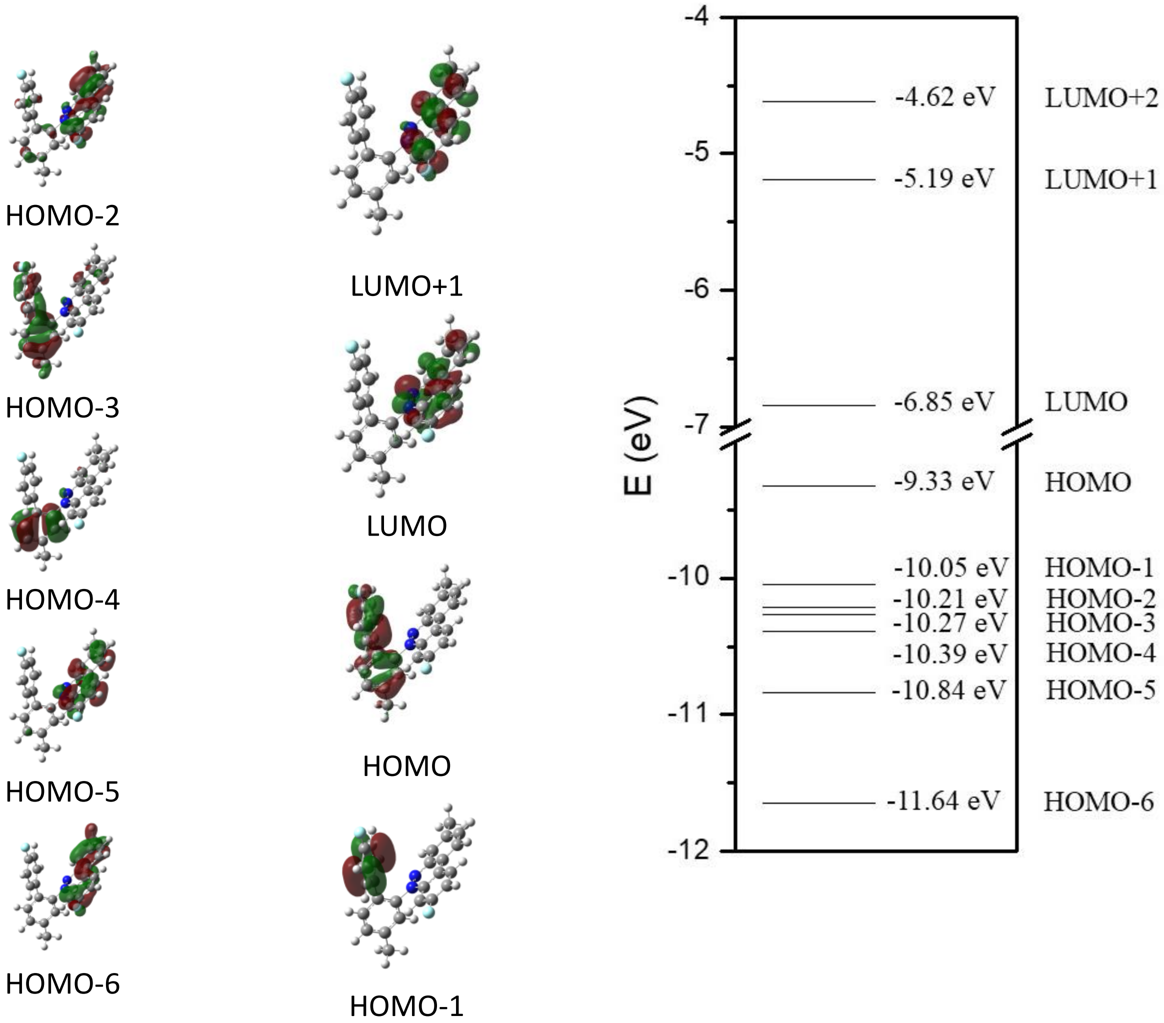

Figure S18. (a) Absorption spectrum of **3l** cation and oscillator strength calculated at B3LYP/6-311G++(2df, 2p) level basis set. (b) MOs of HOMO, HOMO−1, HOMO−2, HOMO−3, HOMO−4, HOMO−5, HOMO−6, LUMO, LUMO+1 (iso value = 0.04) and their corresponding energy levels.

(a)

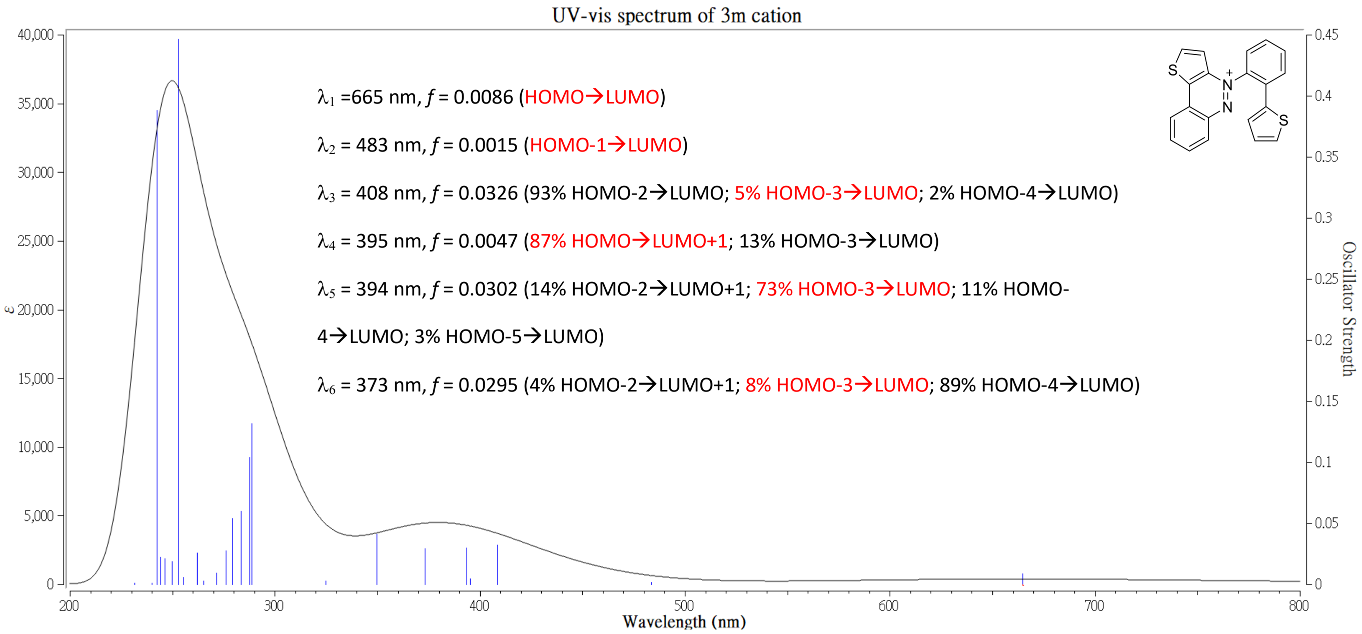

(b)

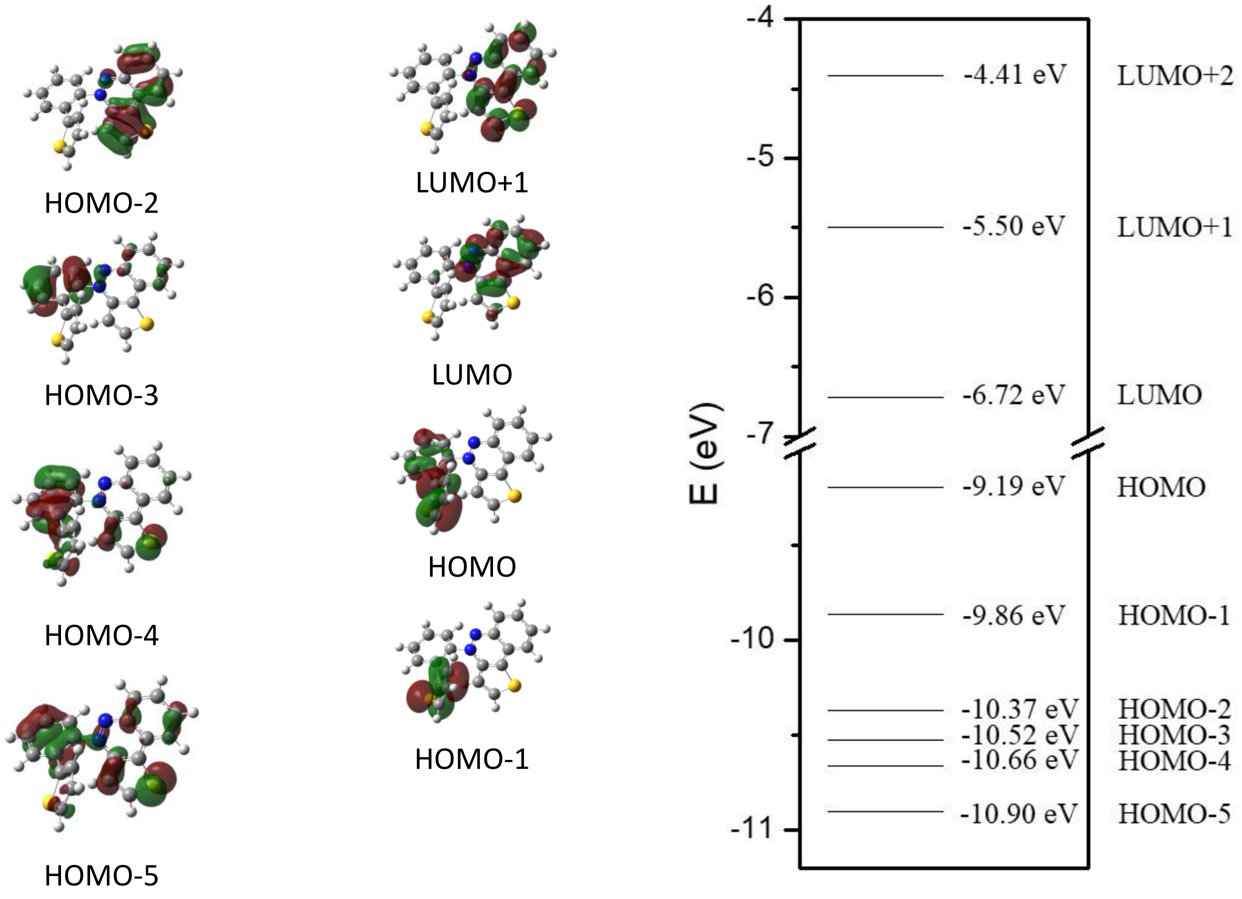

Figure S19. (a) Absorption spectrum of **3m** cation and oscillator strength calculated at B3LYP/6-311G++(2df, 2p) level basis set. (b) MOs of HOMO, HOMO−1, HOMO−2, HOMO−3, HOMO−4, HOMO−5, LUMO, LUMO+1 (iso value = 0.04) and their corresponding energy levels.

(a)

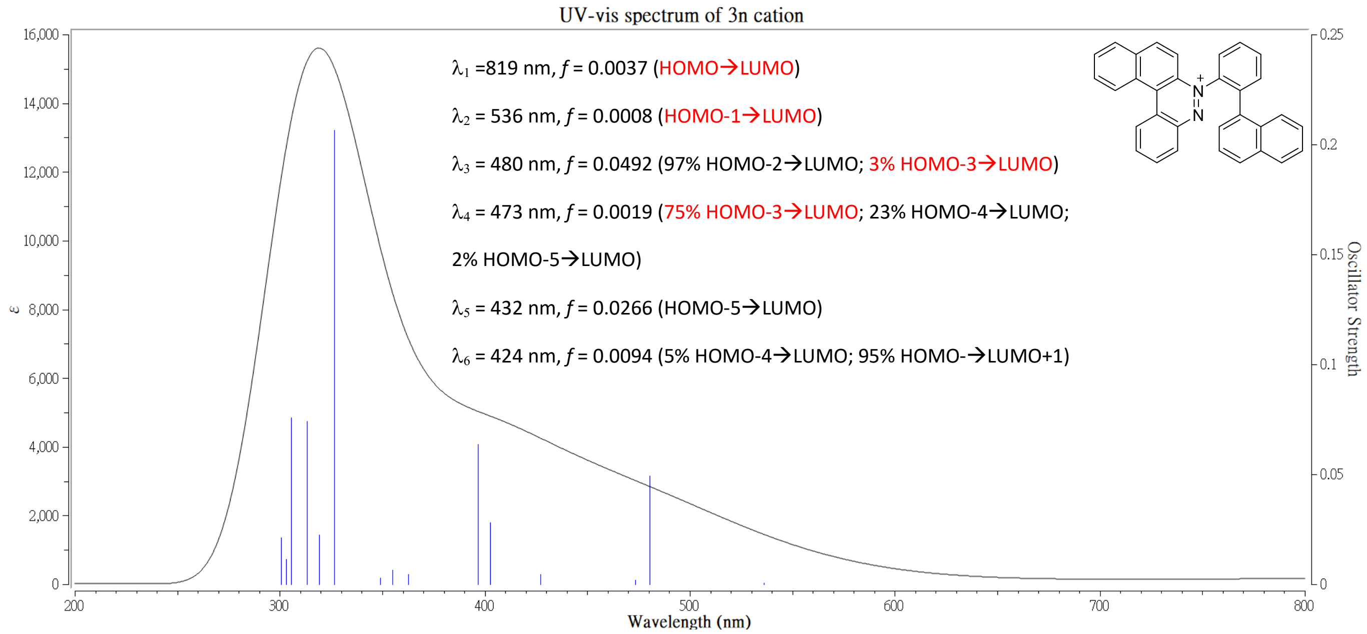

(b)

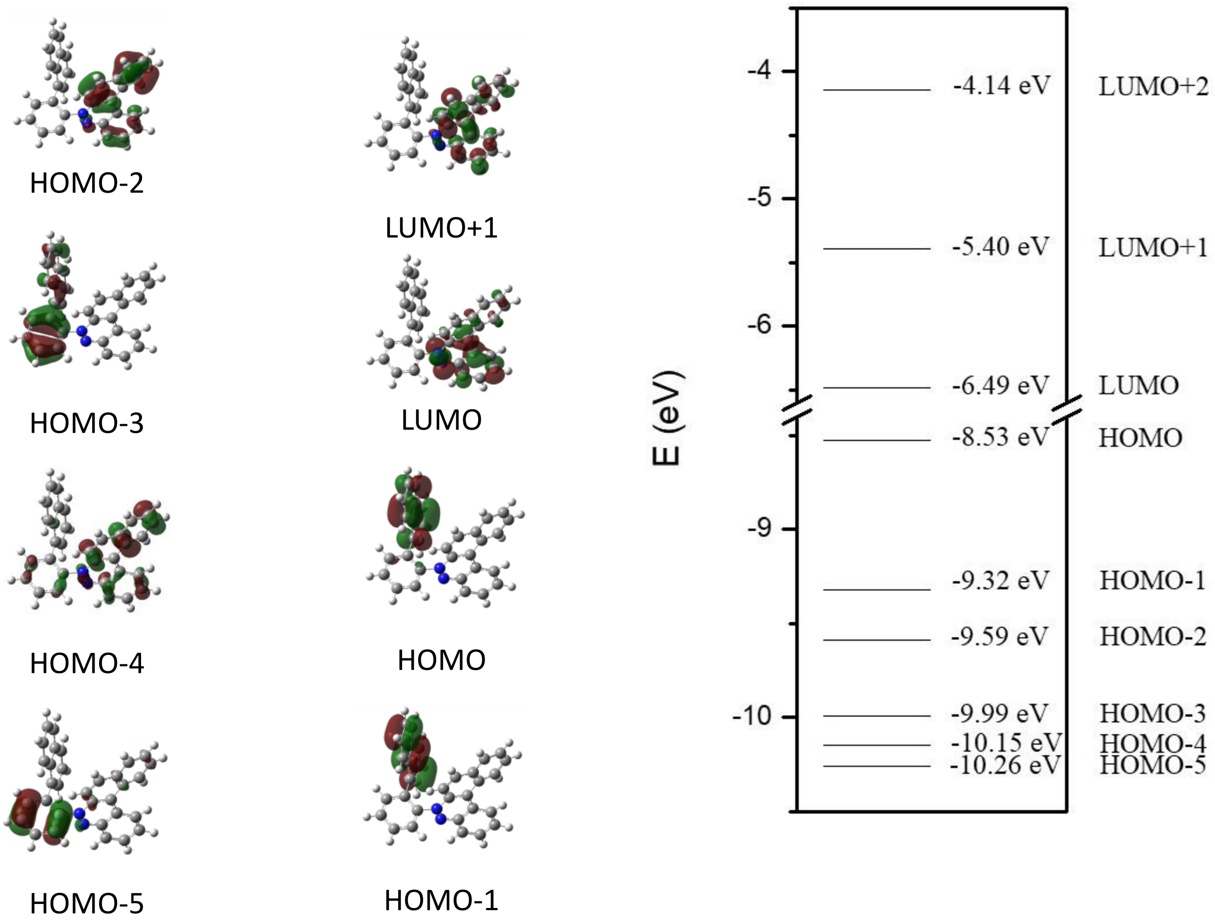

Figure S20. (a) Absorption spectrum of **3n** cation and oscillator strength calculated at B3LYP/6-311G++(2df, 2p) level basis set. (b) MOs of HOMO, HOMO−1, HOMO−2, HOMO−3, HOMO−4, HOMO−5, LUMO, LUMO+1 (iso value = 0.04) and their corresponding energy levels.

(a)

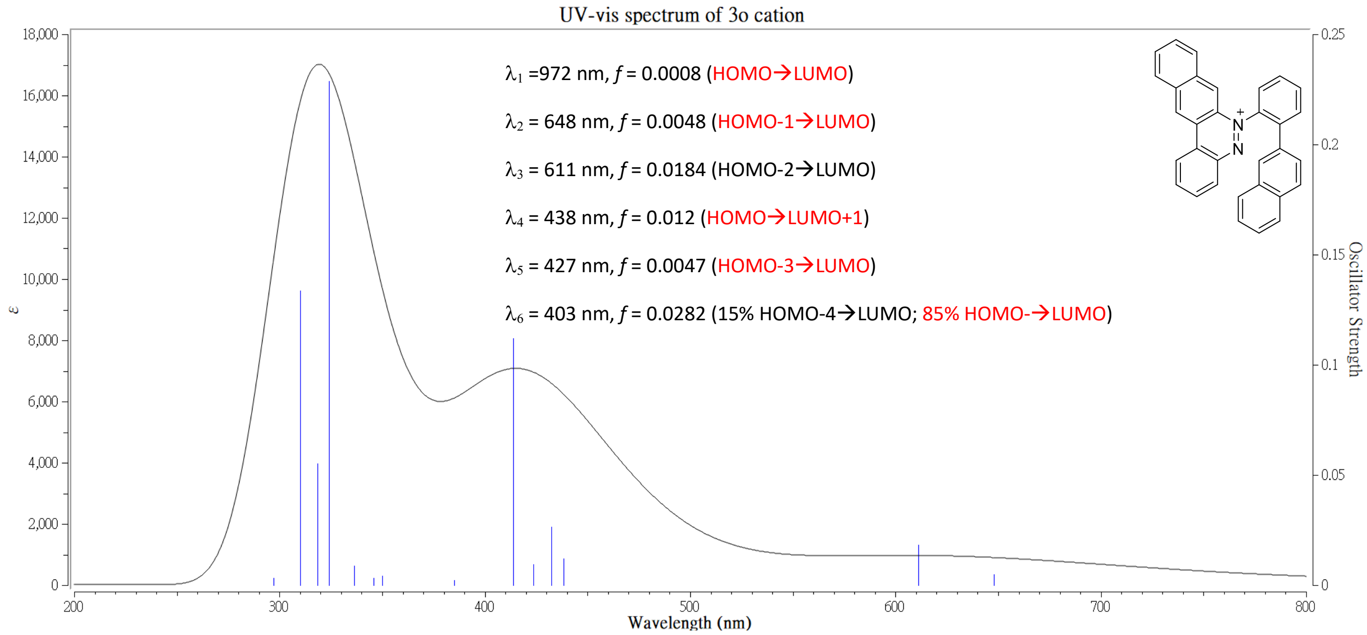

(b)

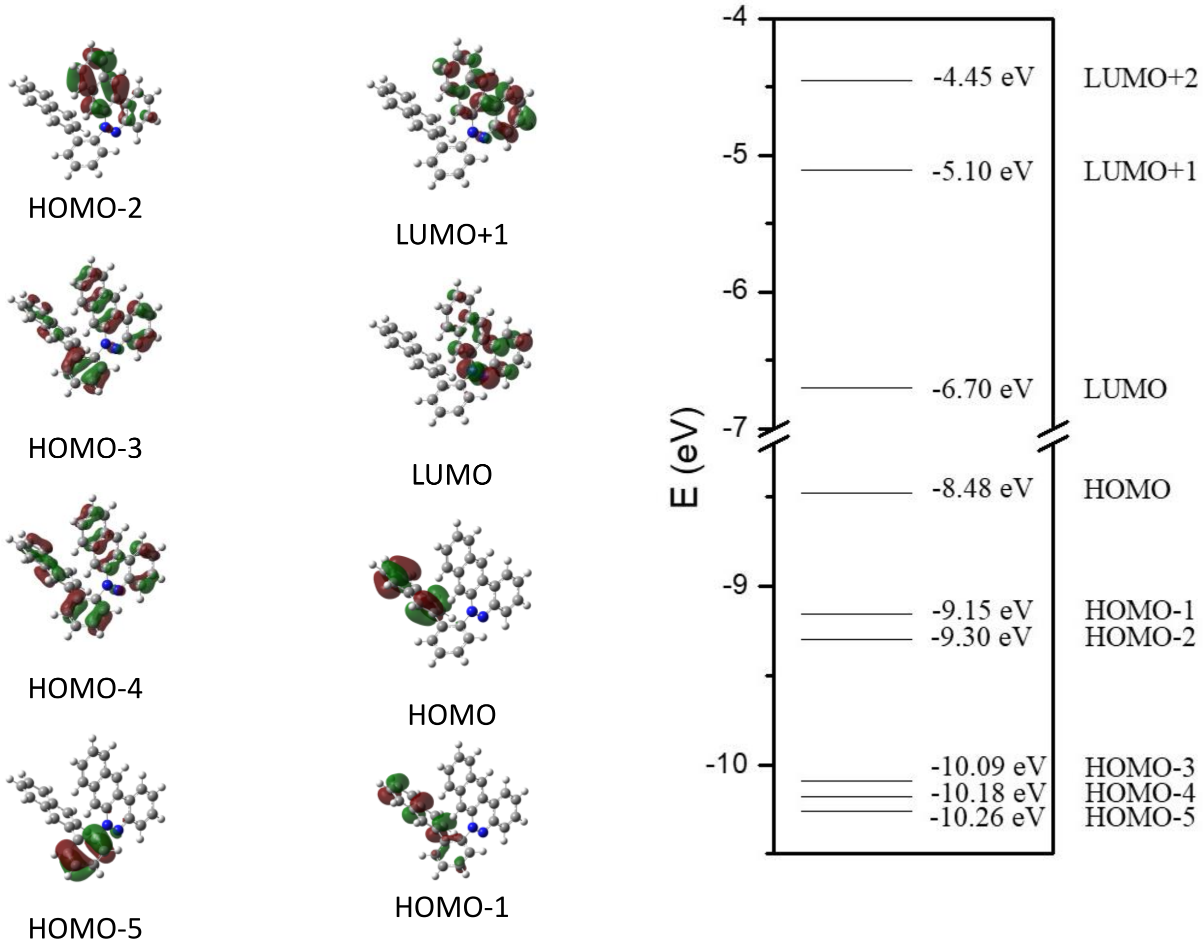

Figure S21. (a) Absorption spectrum of **3o** cation and oscillator strength calculated at B3LYP/6-311G++(2df, 2p) level basis set. (b) MOs of HOMO, HOMO−1, HOMO−2, HOMO−3, HOMO−4, HOMO−5, LUMO, LUMO+1 (iso value = 0.04) and their corresponding energy levels.

(a)

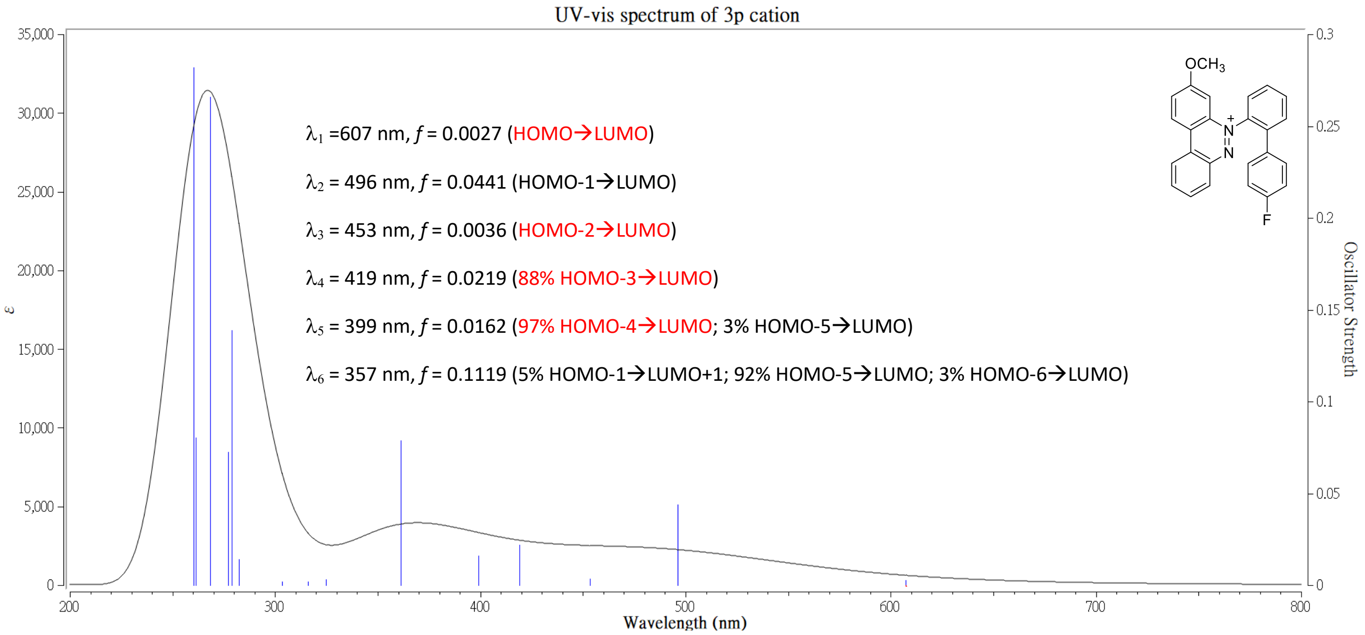

(b)

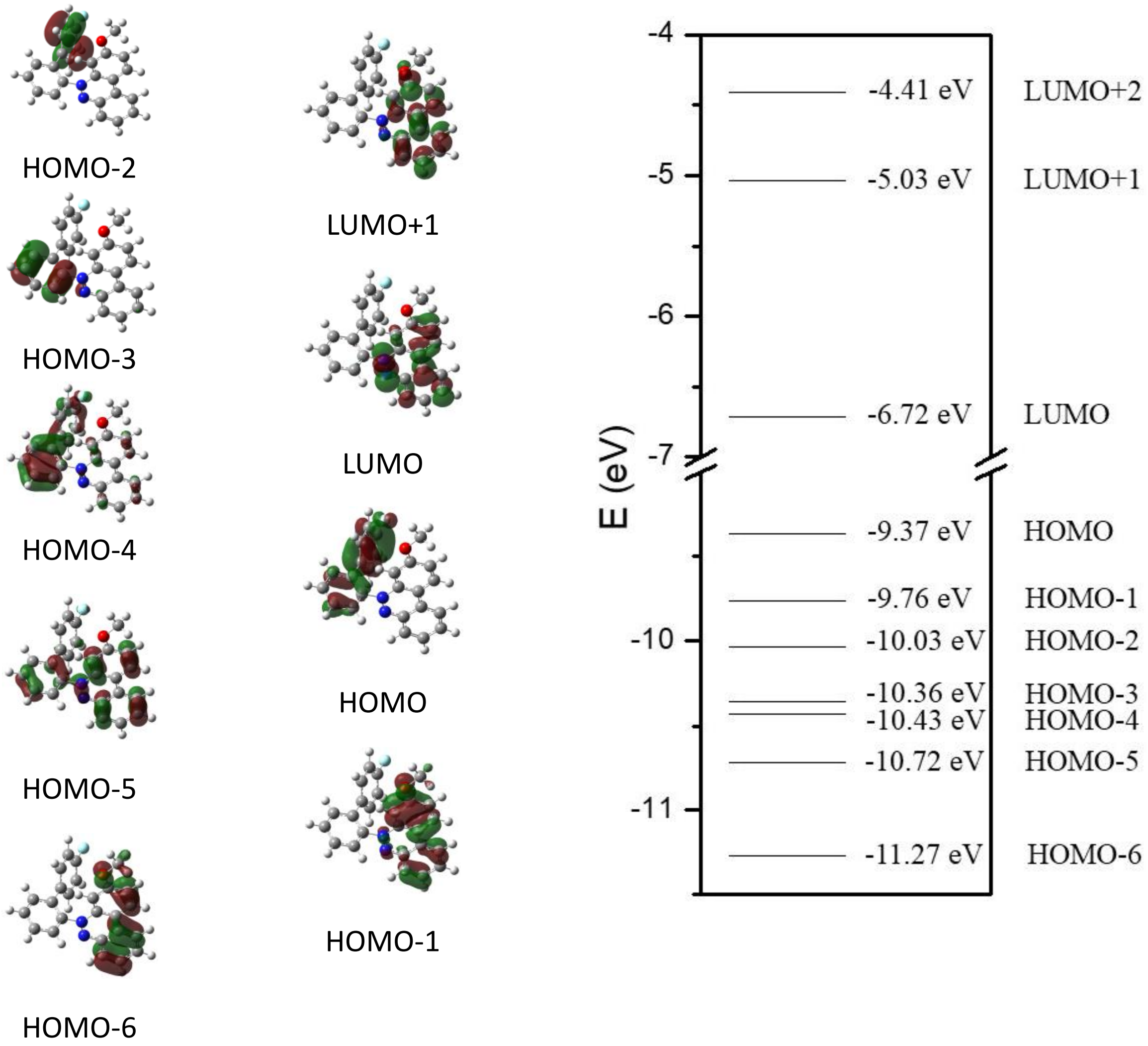

Figure S22. (a) Absorption spectrum of **3p** cation and oscillator strength calculated at B3LYP/6-311G++(2df, 2p) level basis set. (b) MOs of HOMO, HOMO−1, HOMO−2, HOMO−3, HOMO−4, HOMO−5, HOMO−6, LUMO, LUMO+1 (iso value = 0.04) and their corresponding energy levels.

(a)

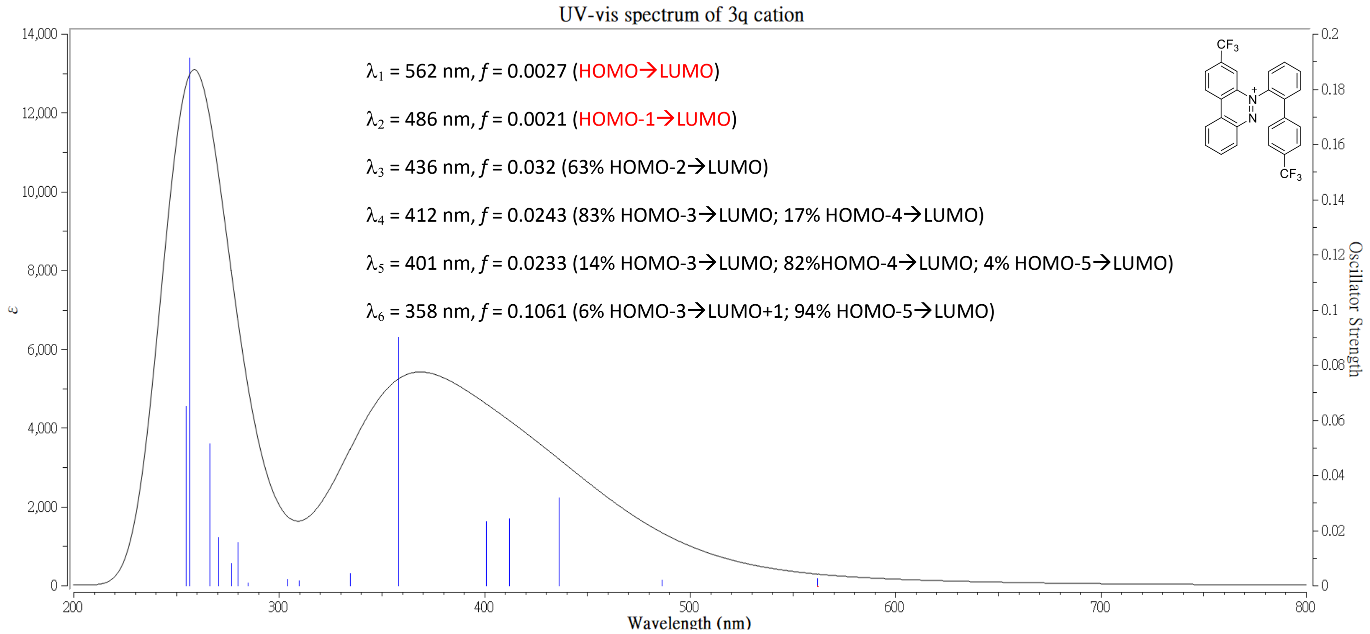

(b)

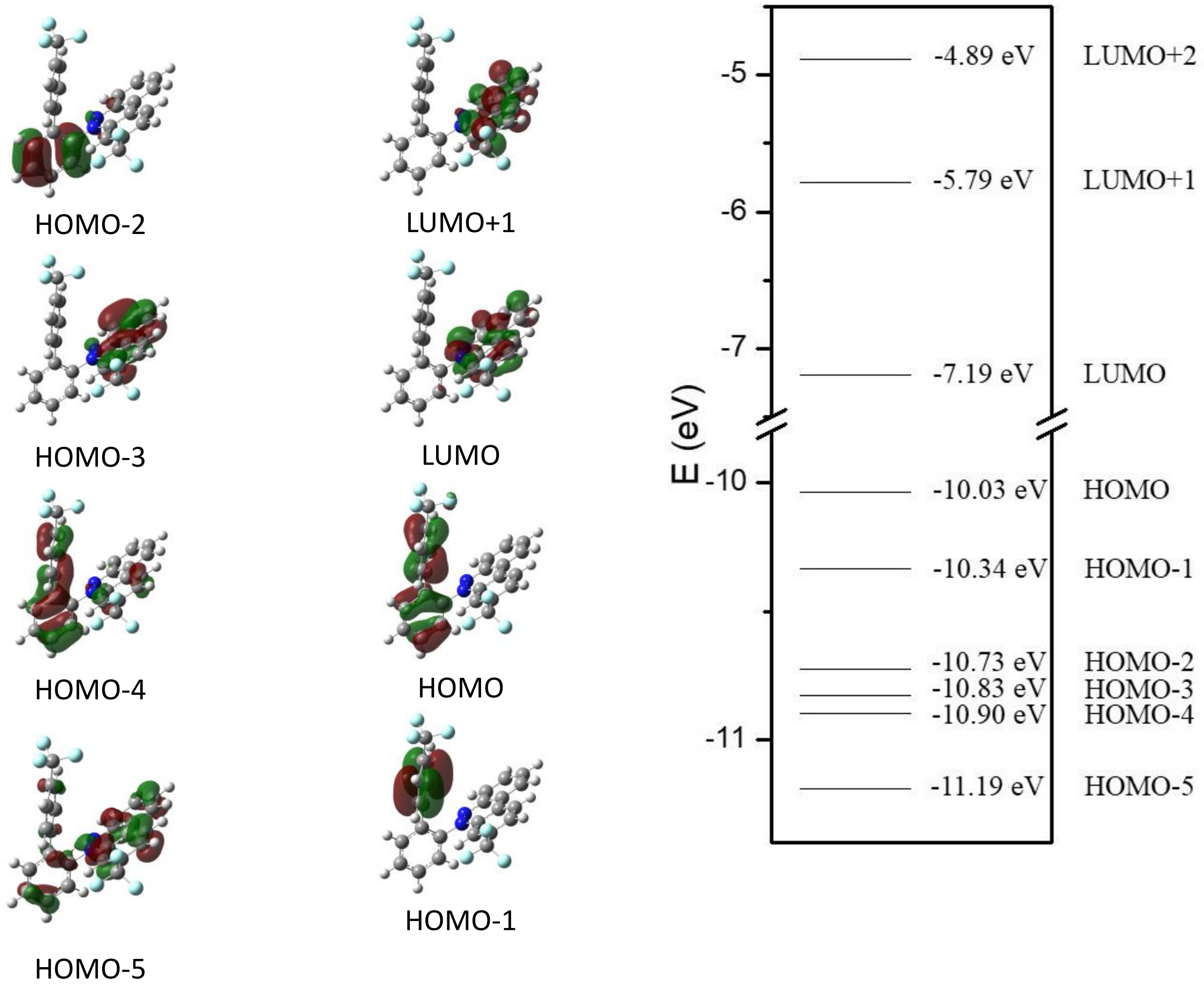

Figure S23. (a) Absorption spectrum of **3q** cation and oscillator strength calculated at B3LYP/6-311G++(2df, 2p) level basis set. (b) MOs of HOMO, HOMO−1, HOMO−2, HOMO−3, HOMO−4, HOMO−5, LUMO, LUMO+1 (iso value = 0.04) and their corresponding energy levels.

(a)

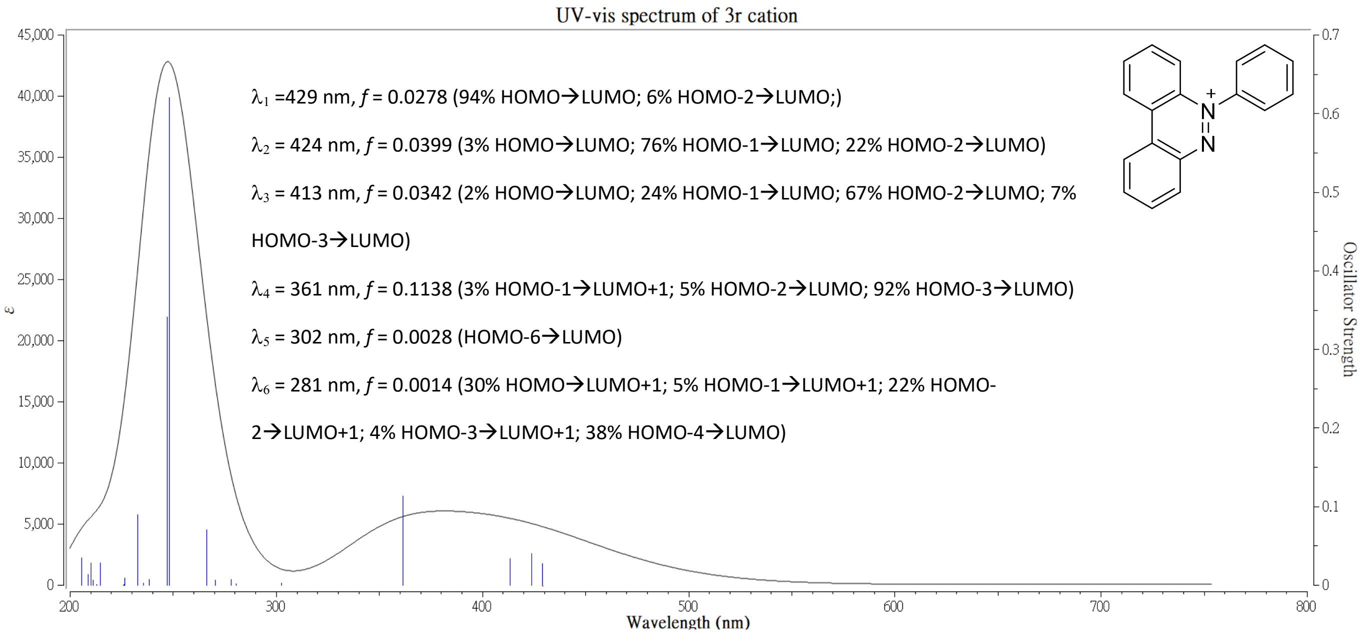

(b)

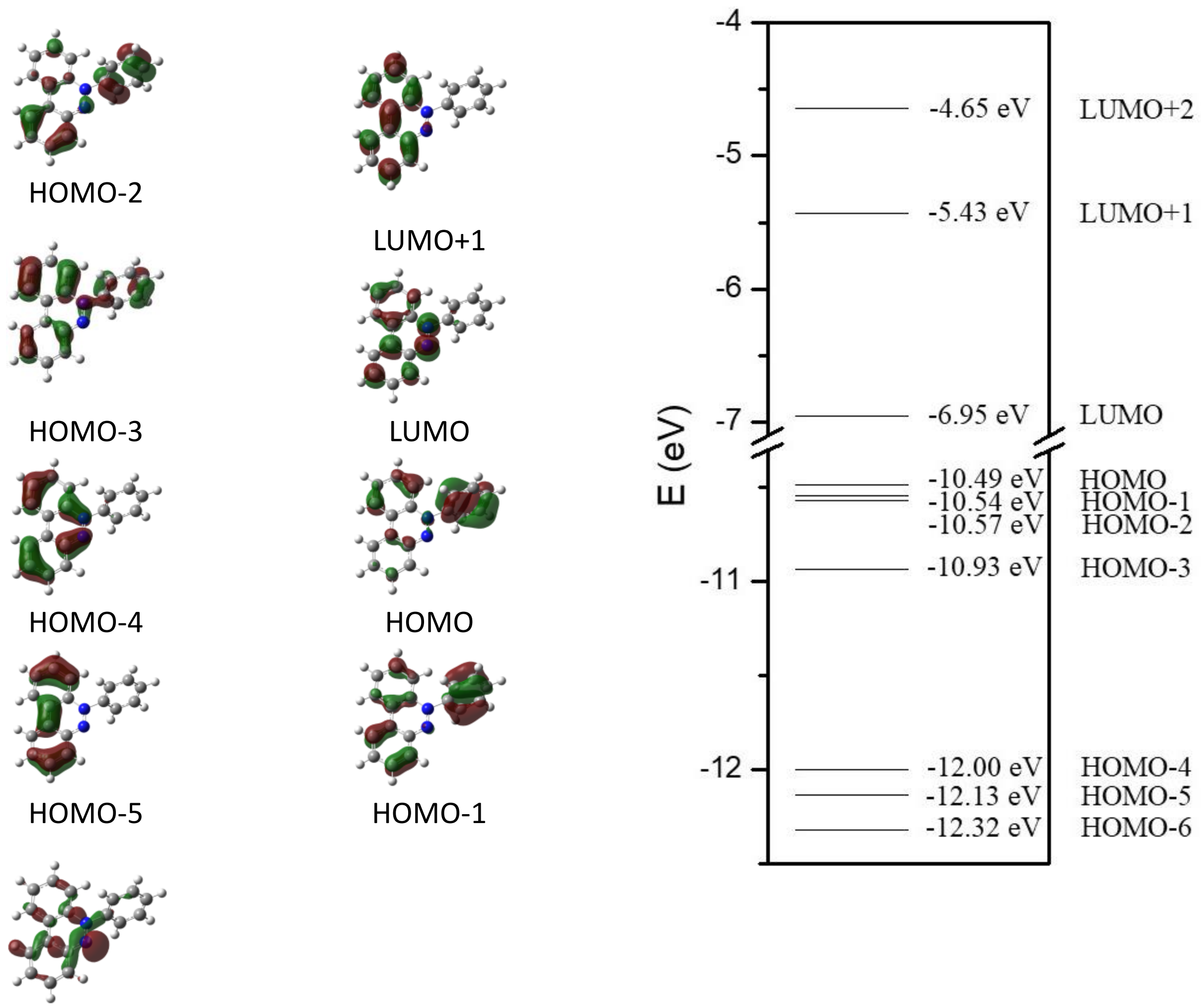

Figure S24. (a) Absorption spectrum of **3r** cation and oscillator strength calculated at B3LYP/6-311G++(2df, 2p) level basis set. (b) MOs of HOMO, HOMO−1, HOMO−2, HOMO−3, HOMO−4, HOMO−5, HOMO−6, LUMO, LUMO+1 (iso value = 0.04) and their corresponding energy levels.

(a)

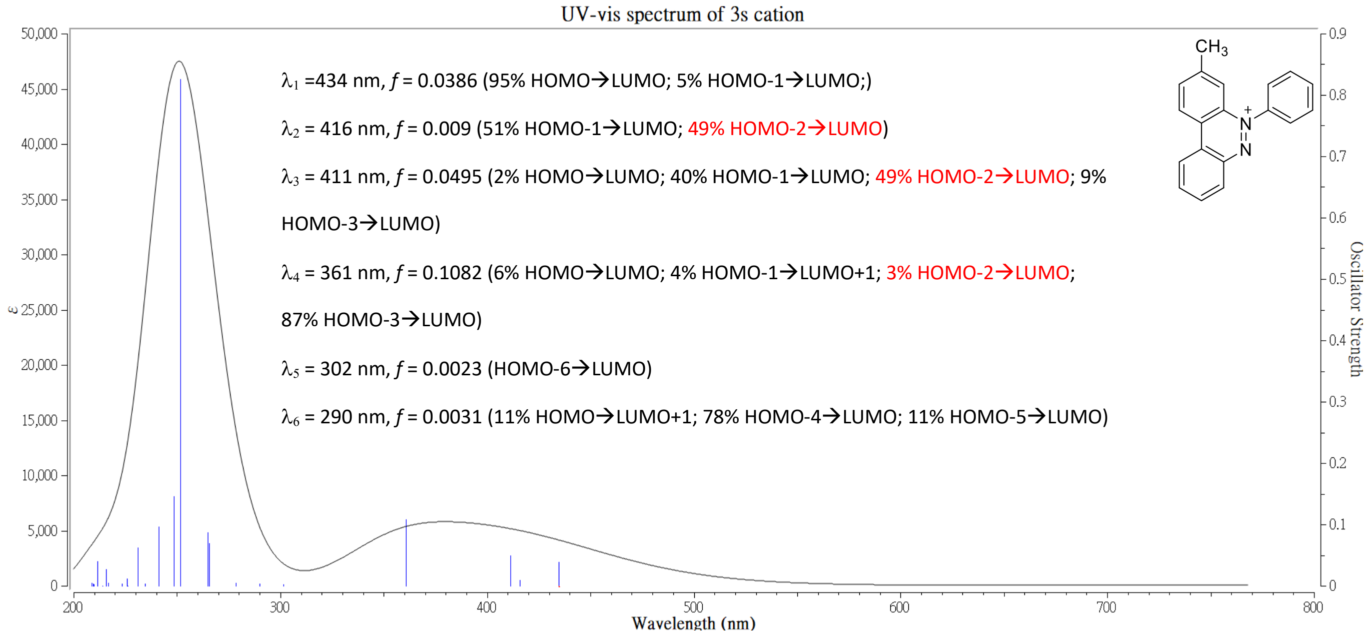

(b)

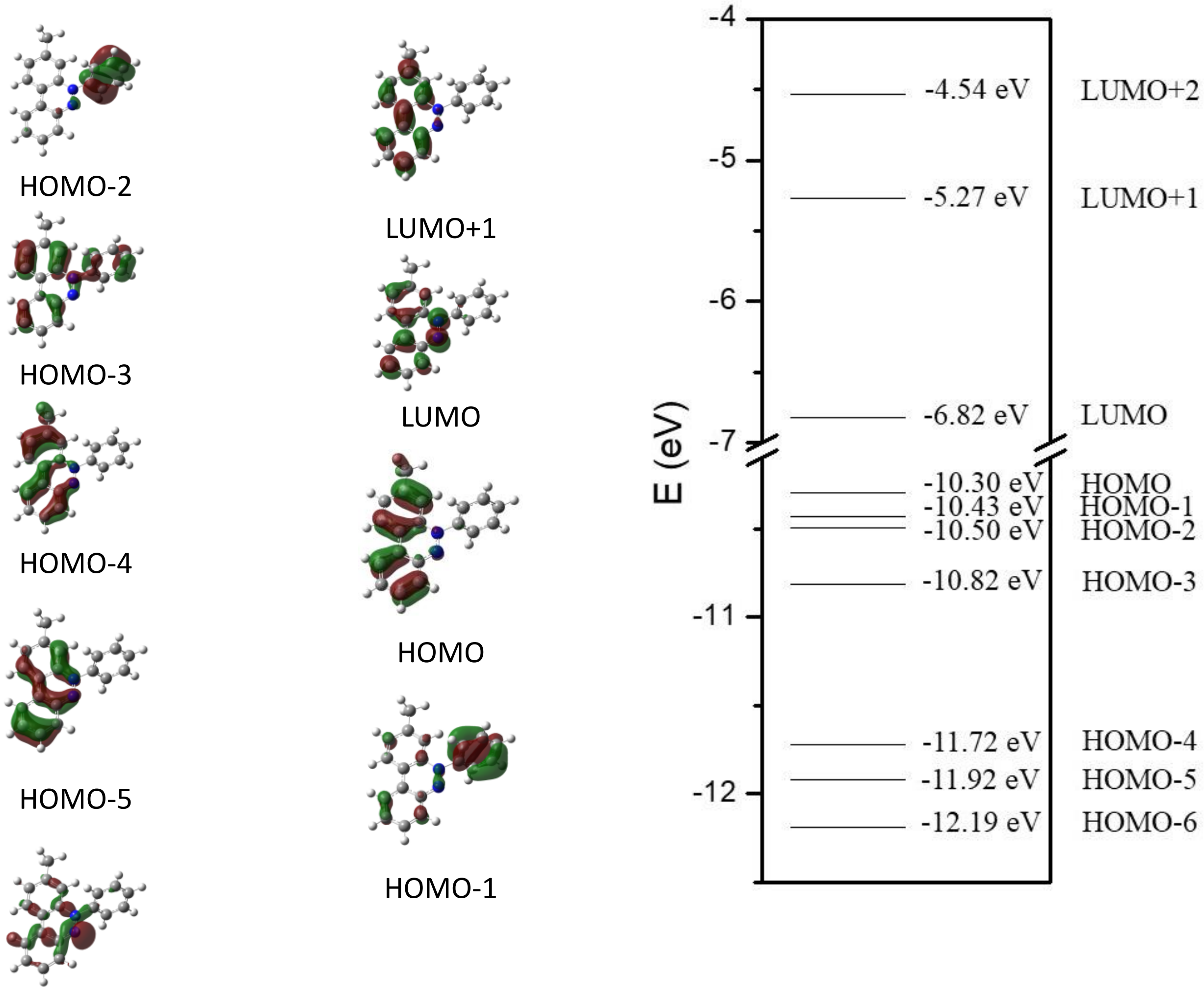

Figure S25. (a) Absorption spectrum of **3s** cation and oscillator strength calculated at B3LYP/6-311G++(2df, 2p) level basis set. (b) MOs of HOMO, HOMO−1, HOMO−2, HOMO−3, HOMO−4, HOMO−5, HOMO−6, LUMO, LUMO+1 (iso value = 0.04) and their corresponding energy levels.

(a)

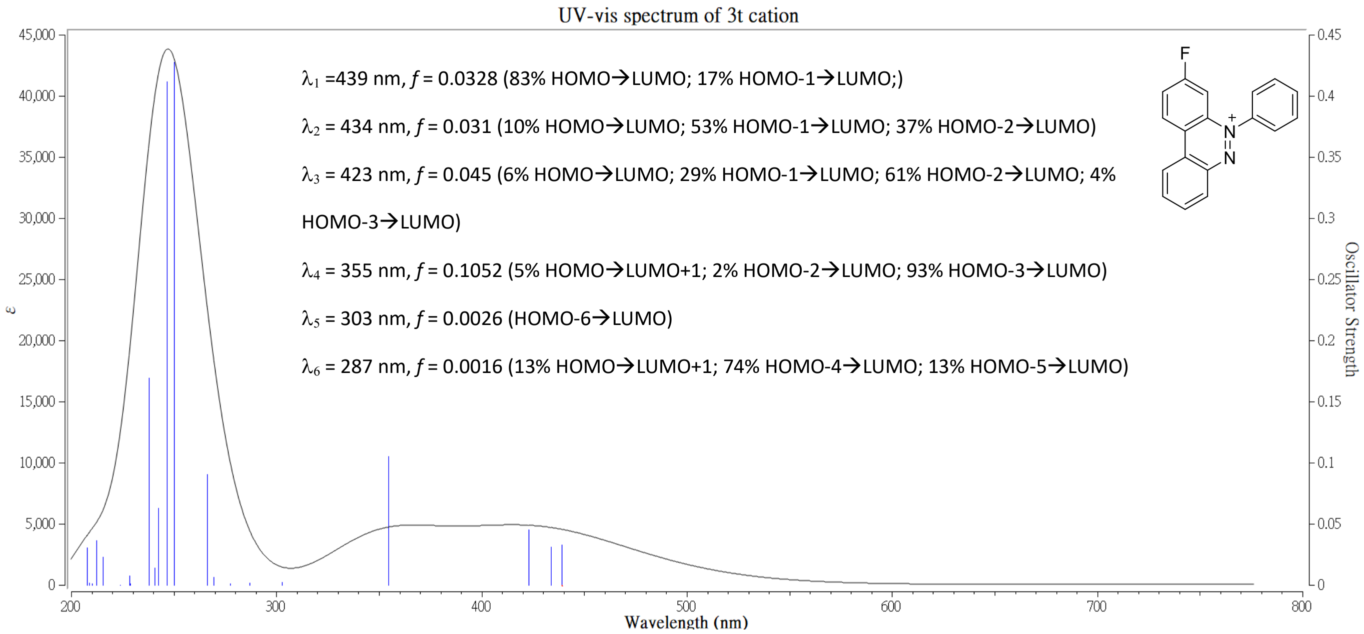

(b)

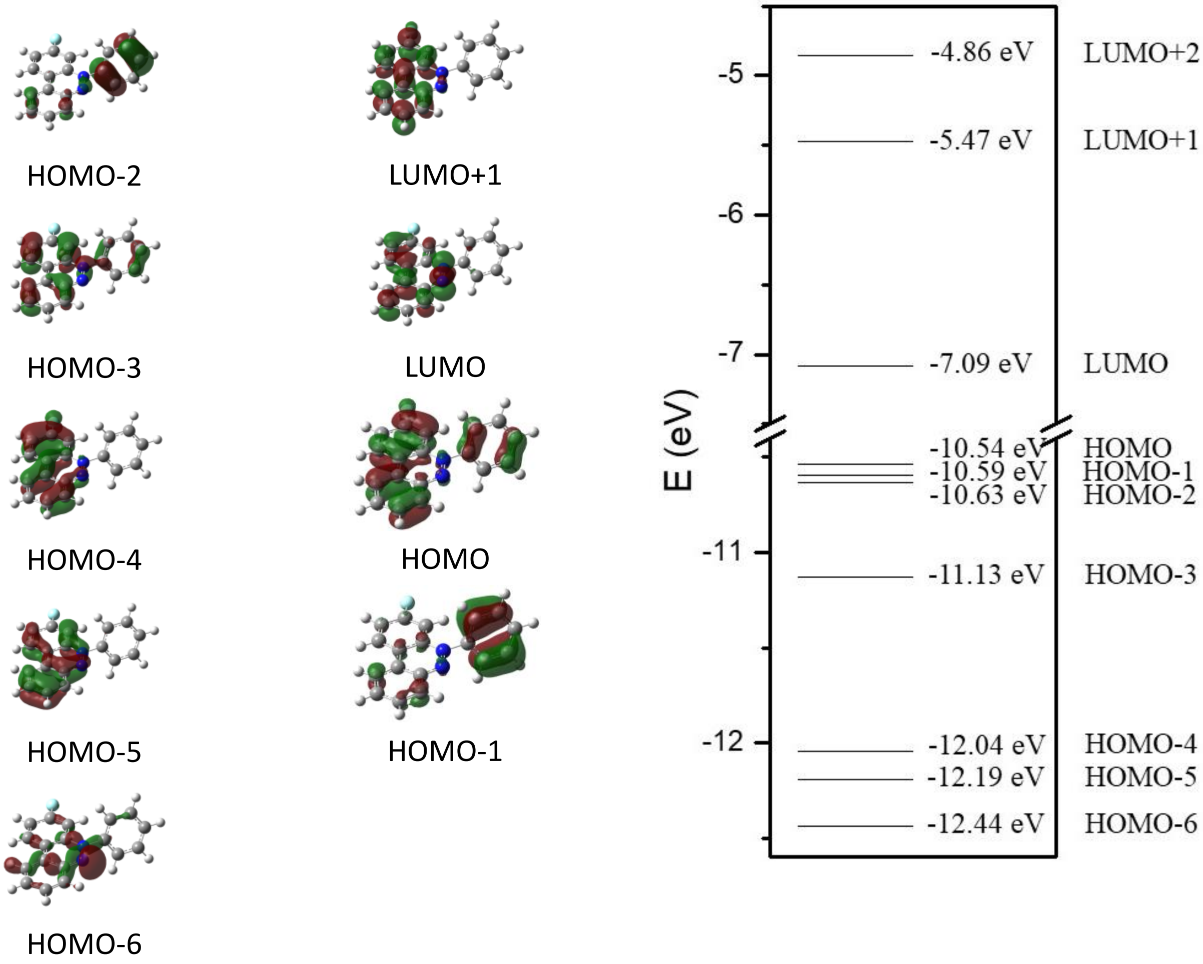

Figure S26. (a) Absorption spectrum of **3t** cation and oscillator strength calculated at B3LYP/6-311G++(2df, 2p) level basis set. (b) MOs of HOMO, HOMO−1, HOMO−2, HOMO−3, HOMO−4, HOMO−5, HOMO−6, LUMO, LUMO+1 (iso value = 0.04) and their corresponding energy levels.

Atomic coordinates of optimized structures

Atomic coordinates of optimized **2a** (N_{imag} = 0)

0 1

C	-4.54760100	-0.06425900	0.85602900
C	-3.49056800	-0.15264000	-0.06564500
C	-3.31326700	0.89904700	-0.98128300
C	-4.16551100	2.00403600	-0.97260400
C	-5.20872600	2.08361700	-0.04500000
C	-5.39617400	1.04506800	0.87025800
H	-4.69094800	-0.86121200	1.58028200
H	-4.01722100	2.80210400	-1.69477300
H	-6.20010300	1.09827600	1.59900400
C	-2.62601900	-1.36421600	-0.09256500
C	-3.23000700	-2.63288700	-0.15084600
C	-1.21047400	-1.30254000	-0.07795800
C	-2.47002200	-3.79942000	-0.20503700
H	-4.31376200	-2.69414100	-0.17918100
C	-0.44717600	-2.48431100	-0.14096400
C	-1.07092600	-3.72363900	-0.20497800
H	-2.96547400	-4.76469500	-0.25631300
H	0.63335800	-2.40014600	-0.12487700
H	-0.47388600	-4.62989200	-0.25034500
N	0.62550800	0.01922900	-0.07189400
N	-0.62551200	-0.01919300	0.07186400
C	1.21048300	1.30256200	0.07799200
C	2.62602900	1.36422100	0.09259900
C	0.44719900	2.48433800	0.14105400
C	3.23003200	2.63288200	0.15093800
C	1.07096300	3.72365600	0.20512500
H	-0.63333600	2.40018700	0.12496500
C	2.47006000	3.79942200	0.20518600
H	4.31378700	2.69412300	0.17927300
H	0.47393400	4.62991400	0.25053600
H	2.96552400	4.76468800	0.25650700
C	3.49056200	0.15263500	0.06561700
C	4.54759600	0.06428900	-0.85605800
C	3.31324200	-0.89909900	0.98119700
C	5.39615200	-1.04504900	-0.87034500
H	4.69095600	0.86127900	-1.58026900
C	4.16547000	-2.00410000	0.97246100

H	2.51269700	-0.84046300	1.71102900
C	5.20868600	-2.08364500	0.04485600
H	6.20008300	-1.09823100	-1.59909100
H	4.01716500	-2.80220500	1.69458600
H	-5.86963700	2.94557300	-0.03761000
H	5.86958600	-2.94561100	0.03742200
H	-2.51272400	0.84038300	-1.71111500

Atomic coordinates of optimized **2b** (N_{imag} = 0)

0 1

C	-4.45800600	-0.97868800	0.84011500
C	-3.39106500	-0.84577600	-0.06253200
C	-3.42380700	0.23442700	-0.96118100
C	-4.48088300	1.14297100	-0.95074200
C	-5.54182500	1.01818900	-0.04051900
C	-5.51022900	-0.06088200	0.85225900
H	-4.45539700	-1.79798900	1.55372100
H	-4.48502800	1.96079600	-1.66787700
H	-6.31746200	-0.18645800	1.57000200
C	-2.30276600	-1.85944300	-0.09147900
C	-2.64041800	-3.22375100	-0.15155300
C	-0.92746700	-1.51708600	-0.07699800
C	-1.66322700	-4.21512900	-0.20632400
H	-3.69005900	-3.50020500	-0.18128400
C	0.05624400	-2.52295200	-0.14208400
C	-0.30722600	-3.86179000	-0.20667400
H	-1.95621300	-5.25983400	-0.25836400
H	1.09818000	-2.22448700	-0.12632600
N	0.60848900	0.14411300	-0.07709800
N	-0.60848600	-0.14402700	0.07723100
C	0.92749500	1.51715500	0.07724900
C	2.30280000	1.85948500	0.09156500
C	-0.05618900	2.52302900	0.14259700
C	2.64049100	3.22377700	0.15175000
C	0.30731800	3.86185400	0.20728400
H	-1.09813300	2.22458700	0.12695300
C	1.66332600	4.21516800	0.20677200
H	3.69014400	3.50019800	0.18135200
H	1.95633800	5.25986200	0.25888300
C	3.39107700	0.84579800	0.06232500
C	4.45774100	0.97864300	-0.84051800

C	3.42391200	-0.23453600	0.96095100
C	5.50995100	0.06068500	-0.85292000
H	4.45500400	1.79788300	-1.55419300
C	4.48084800	-1.14310000	0.95026500
H	2.62440000	-0.35058600	1.68514100
C	5.54172100	-1.01828900	0.03982100
H	6.31695200	0.18612400	-1.57092800
H	4.48500600	-1.96103800	1.66729200
H	-2.62424400	0.35045800	-1.68531600
H	0.45863200	-4.63069200	-0.25284900
C	-6.66636000	2.02714300	-0.01298100
H	-7.56177000	1.61487200	0.46190200
H	-6.37891100	2.92398100	0.55061300
H	-6.93730200	2.35188500	-1.02299700
H	-0.45851900	4.63076500	0.25365600
C	6.66650500	-2.02699500	0.01309900
H	6.37140400	-2.93387100	-0.53003800
H	7.55436000	-1.62250700	-0.48211800
H	6.95284000	-2.33378100	1.02457000

Atomic coordinates of optimized **2c** (N_{imag} = 0)

0 1

C	-4.58670200	-0.33823000	0.48174500
C	-3.45334100	-0.38599800	-0.34676600
C	-3.26557300	0.64034500	-1.28675800
C	-4.19218700	1.67835300	-1.38564300
C	-5.30923800	1.71325900	-0.54758300
C	-5.51899300	0.70447800	0.40294200
H	-4.73289600	-1.12381400	1.21932500
H	-4.04711700	2.46070600	-2.12562200
C	-2.51595600	-1.53878800	-0.25388800
C	-3.03026200	-2.84617800	-0.31640300
C	-1.11392900	-1.38256500	-0.12190900
C	-2.19571100	-3.96060200	-0.26180300
H	-4.10130600	-2.98007100	-0.43479200
C	-0.27412300	-2.51207700	-0.07501700
C	-0.80966600	-3.79174200	-0.14500400
H	-2.62226700	-4.95798100	-0.31856100
H	0.79323300	-2.35513200	0.03072900
H	-0.15452700	-4.65718100	-0.10404700
N	0.62626200	0.05957900	-0.03225400

N	-0.62626900	-0.05969000	0.03217700
C	1.11388300	1.38246200	0.12185000
C	2.51590400	1.53874400	0.25385700
C	0.27403400	2.51193800	0.07489100
C	3.03015400	2.84616000	0.31631300
C	0.80952200	3.79162700	0.14483900
H	-0.79331300	2.35494500	-0.03088800
C	2.19555900	3.96054700	0.26165500
H	4.10119000	2.98010400	0.43471500
H	0.15434900	4.65703800	0.10382800
H	2.62207300	4.95794600	0.31837700
C	3.45334900	0.38600600	0.34680200
C	4.58670900	0.33825300	-0.48172100
C	3.26567000	-0.64027300	1.28687500
C	5.51906300	-0.70438900	-0.40286200
H	4.73284100	1.12379100	-1.21936000
C	4.19235200	-1.67822000	1.38581700
H	2.40724000	-0.61169500	1.94933200
C	5.30938400	-1.71312200	0.54773800
H	4.04734600	-2.46052600	2.12585900
H	-6.02506200	2.52719000	-0.63460200
H	6.02525900	-2.52700300	0.63480200
H	-2.40712200	0.61176500	-1.94918900
C	-6.70398400	0.75510000	1.34079200
H	-6.48327000	1.37032000	2.22229900
H	-7.58175000	1.18984000	0.85191200
H	-6.97471500	-0.24305500	1.69840500
C	6.70406200	-0.75501200	-1.34070100
H	7.58214000	-1.18873100	-0.85146900
H	6.48375200	-1.37115100	-2.22166600
H	6.97410200	0.24301900	-1.69917700

Atomic coordinates of optimized **2d** (N_{imag} = 0)

0 1

C	-3.69961500	-0.61999300	-0.97765200
C	-3.46600000	0.23120100	0.12591200
C	-4.12432300	-0.01454100	1.34015700
C	-4.99454000	-1.09668900	1.48716400
C	-5.22252000	-1.94430900	0.40291600
C	-4.58088100	-1.69767700	-0.81317900
H	-5.48814400	-1.27180200	2.43871700

C	-2.58019900	1.43394300	0.02555900
C	-3.15825900	2.71352400	0.03927100
C	-1.17141000	1.34010800	-0.04781900
C	-2.37352400	3.86437600	-0.03886200
H	-4.23932000	2.79720200	0.10171500
C	-0.38166200	2.50220000	-0.13076100
C	-0.97977700	3.75656400	-0.12899700
H	-2.84673000	4.84214900	-0.03478300
H	0.69504700	2.39347700	-0.19426400
H	-0.36571800	4.64992300	-0.19729400
N	0.62796100	-0.02915800	0.03018800
N	-0.62795600	0.02908200	-0.03019500
C	1.17144700	-1.34017600	0.04783800
C	2.58023800	-1.43397000	-0.02553800
C	0.38173700	-2.50229100	0.13082200
C	3.15834100	-2.71353300	-0.03919500
C	0.97989200	-3.75663600	0.12910200
H	-0.69497500	-2.39360100	0.19432800
C	2.37364300	-3.86440700	0.03897500
H	4.23940500	-2.79717700	-0.10163300
H	0.36586200	-4.65001200	0.19743200
H	2.84688100	-4.84216500	0.03493300
C	3.46599800	-0.23120100	-0.12592900
C	3.69953200	0.62006800	0.97759200
C	4.12435100	0.01450100	-1.34016600
C	4.58075500	1.69778200	0.81308900
C	4.99452700	1.09667700	-1.48720400
H	3.93968000	-0.64830300	-2.18127100
C	5.22243100	1.94436900	-0.40299600
H	5.48815800	1.27175700	-2.43874900
H	-5.89960100	-2.78862000	0.49762700
H	5.89947800	2.78870500	-0.49773100
H	-3.93959600	0.64820700	2.18129300
H	4.77025600	2.35291400	1.66000100
H	-4.77044800	-2.35274700	-1.66012300
C	-3.02950900	-0.39007400	-2.31190600
H	-3.11493800	0.65336000	-2.63356300
H	-1.96008100	-0.62329900	-2.25837300
H	-3.47524800	-1.02207000	-3.08524800
C	3.02936300	0.39021000	2.31182500
H	1.95992500	0.62337500	2.25821600

H	3.11482700	-0.65319600	2.63356300
H	3.47502400	1.02228600	3.08514700

Atomic coordinates of optimized **2e** (N_{imag} = 0)

0 1

C	4.39782400	-1.31207900	-0.71342900
C	3.31157000	-1.10910200	0.14771200
C	3.39244300	-0.03614900	1.05764500
C	4.50591600	0.79257300	1.09936500
C	5.57957600	0.57898700	0.22072100
C	5.52330300	-0.48104200	-0.69131700
H	4.36071500	-2.12099300	-1.43767700
H	4.56911900	1.61090300	1.80953000
H	6.33272100	-0.66782100	-1.38698900
C	2.14882100	-2.03469600	0.12220200
C	2.37627500	-3.42286000	0.16103900
C	0.80464600	-1.58600300	0.07349600
C	1.32401500	-4.33555500	0.16122800
H	3.39955000	-3.78169900	0.21860800
C	-0.25575300	-2.51317100	0.08635900
C	-0.00004500	-3.87742100	0.12953500
H	1.53331300	-5.40088700	0.19753700
H	-1.27049200	-2.13410400	0.04699300
N	-0.59797100	0.18981100	0.05970400
N	0.59797500	-0.18978600	-0.05975100
C	-0.80465200	1.58602200	-0.07359200
C	-2.14883000	2.03470500	-0.12227900
C	0.25574200	2.51319600	-0.08652300
C	-2.37629400	3.42286600	-0.16116500
C	0.00002400	3.87744300	-0.12974600
H	1.27048400	2.13413700	-0.04716900
C	-1.32404000	4.33556700	-0.16142000
H	-3.39957400	3.78169600	-0.21871800
H	-1.53334500	5.40089700	-0.19776700
C	-3.31157400	1.10910300	-0.14771700
C	-4.39780200	1.31210500	0.71345000
C	-3.39246500	0.03611200	-1.05760500
C	-5.52327500	0.48105800	0.69140400
H	-4.36067700	2.12104900	1.43766400
C	-4.50593300	-0.79262000	-1.09925700
H	-2.57664800	-0.13764800	-1.75094000

C	-5.57956800	-0.57900600	-0.22058900
H	-6.33267500	0.66786000	1.38709300
H	-4.56915100	-1.61098000	-1.80938800
H	2.57660800	0.13758900	1.75096500
H	-0.82455200	-4.58452900	0.13457800
H	0.82452600	4.58455500	-0.13484200
O	6.62594900	1.45186600	0.33455500
O	-6.62593900	-1.45189600	-0.33435900
C	-7.74757800	-1.28276700	0.52315600
H	-8.44806700	-2.07516400	0.25659100
H	-7.46433200	-1.38755800	1.57812300
H	-8.22583600	-0.30727000	0.36867800
C	7.74762300	1.28274800	-0.52291600
H	7.46442800	1.38759100	-1.57789100
H	8.44811700	2.07511800	-0.25628500
H	8.22585200	0.30723400	-0.36845800

Atomic coordinates of optimized **2f** (N_{imag} = 0)

0 1

C	4.54988900	-0.68630700	-0.34103300
C	3.39706900	-0.66780200	0.44989400
C	3.25652200	0.33799800	1.42617500
C	4.25568100	1.29227900	1.58834000
C	5.40778000	1.28192700	0.79113100
C	5.54980200	0.28371200	-0.17908000
H	4.68350500	-1.43832100	-1.11202700
H	4.14754000	2.06036300	2.34913900
C	2.37664200	-1.74029700	0.28599100
C	2.78611900	-3.08481200	0.32200500
C	0.99711300	-1.46946800	0.11335700
C	1.86967000	-4.12773100	0.20337400
H	3.83902400	-3.30545400	0.46904400
C	0.07413000	-2.52708100	0.00122900
C	0.50610000	-3.84649400	0.04647400
H	2.21521500	-5.15687200	0.24028800
H	-0.97322500	-2.28313700	-0.13514200
H	-0.21239000	-4.65603000	-0.04491700
N	-0.62046500	0.10786100	0.01187900
N	0.62047700	-0.10774900	-0.01182000
C	-0.99713000	1.46954200	-0.11361500
C	-2.37667300	1.74030000	-0.28624600

C	-0.07416700	2.52720300	-0.00176700
C	-2.78618500	3.08479600	-0.32254400
C	-0.50617200	3.84659500	-0.04729200
H	0.97320000	2.28331500	0.13461500
C	-1.86975600	4.12776500	-0.20419400
H	-3.83910300	3.30537900	-0.46958100
H	0.21230300	4.65616900	0.04388400
H	-2.21532700	5.15688800	-0.24132400
C	-3.39707700	0.66773900	-0.44985100
C	-4.54987300	0.68641000	0.34110700
C	-3.25652400	-0.33831000	-1.42587400
C	-5.54976100	-0.28368100	0.17943000
H	-4.68349000	1.43861600	1.11191300
C	-4.25565800	-1.29266400	-1.58776500
H	-2.37643600	-0.35583700	-2.05858900
C	-5.40773700	-1.28214000	-0.79052900
H	-4.14751200	-2.06094300	-2.34836600
H	6.16863800	2.03916700	0.93715100
H	-6.16857500	-2.03944100	-0.93633300
H	2.37642100	0.35538500	2.05887500
O	6.62338800	0.16688400	-1.02037800
O	-6.62332700	-0.16667000	1.02073000
C	-7.67091600	-1.12235900	0.92034500
H	-7.30818000	-2.13746100	1.12610000
H	-8.40416600	-0.84046400	1.67706700
H	-8.14294200	-1.09730400	-0.07010500
C	7.67100300	1.12251500	-0.91972000
H	8.40426700	0.84079000	-1.67649200
H	7.30830400	2.13768100	-1.12522700
H	8.14299800	1.09719400	0.07073800

Atomic coordinates of optimized **2g** (N_{imag} = 0)

0 1

C	4.46215100	-0.87382300	-0.85729000
C	3.40720800	-0.77211700	0.06542500
C	3.42101000	0.29803300	0.97726100
C	4.45037900	1.23962800	0.97091100
C	5.46858000	1.10347200	0.03494600
C	5.49751700	0.06318100	-0.88380800
H	4.46395500	-1.68427600	-1.57997300
H	4.47263500	2.06179100	1.67822400

H	6.30764600	-0.00383400	-1.60190100
C	2.34139300	-1.81047200	0.09571500
C	2.70993800	-3.16650800	0.15055500
C	0.95929900	-1.49710400	0.08404300
C	1.75449800	-4.17905200	0.20396900
H	3.76533200	-3.42055900	0.17757100
C	-0.00170300	-2.52467400	0.14808900
C	0.39119200	-3.85529300	0.20822700
H	2.07017300	-5.21714000	0.25283000
H	-1.05008200	-2.24999400	0.13703800
N	-0.61379200	0.12957400	0.05898300
N	0.61380300	-0.12947600	-0.05894400
C	-0.95932400	1.49717300	-0.08419000
C	-2.34142700	1.81050100	-0.09586500
C	0.00164700	2.52476100	-0.14840800
C	-2.71001200	3.16651800	-0.15088400
C	-0.39128800	3.85536000	-0.20872000
H	1.05003400	2.25011200	-0.13735000
C	-1.75460300	4.17908100	-0.20446800
H	-3.76541400	3.42053500	-0.17790000
H	-2.07030800	5.21715400	-0.25346400
C	-3.40720600	0.77211600	-0.06539100
C	-4.46213900	0.87393600	0.85732300
C	-3.42097600	-0.29819000	-0.97704400
C	-5.49746900	-0.06310400	0.88401000
H	-4.46396500	1.68451000	1.57987000
C	-4.45030800	-1.23982400	-0.97052300
H	-2.62553900	-0.38692300	-1.70862300
C	-5.46850400	-1.10354800	-0.03456900
H	-6.30759200	0.00400100	1.60210200
H	-4.47253900	-2.06210800	-1.67769500
H	2.62557200	0.38667100	1.70884900
H	-0.35745800	-4.64072500	0.25509400
H	0.35733900	4.64080700	-0.25571800
F	6.47350800	2.02098100	0.02081600
F	-6.47339500	-2.02109400	-0.02027200

Atomic coordinates of optimized **2h** (N_{imag} = 0)

0 1

C	4.56954300	-0.33539900	-0.55358600
C	3.45725600	-0.37987900	0.30317400

C	3.29073200	0.64695300	1.24902900
C	4.21144000	1.69210300	1.33421800
C	5.31548400	1.74534400	0.47771600
C	5.46315600	0.72224600	-0.44891100
H	4.73077900	-1.09758800	-1.30837200
H	4.07333700	2.47235000	2.07678600
C	2.52048300	-1.53498700	0.22954600
C	3.04065100	-2.83978100	0.28536100
C	1.11659400	-1.38036100	0.12134500
C	2.20732500	-3.95592800	0.24977300
H	4.11401100	-2.97146300	0.38325900
C	0.27910700	-2.51197200	0.09311200
C	0.81916200	-3.78996800	0.15801300
H	2.63687700	-4.95208600	0.30171100
H	-0.79036100	-2.35840800	0.00677600
H	0.16548500	-4.65698200	0.13303800
N	-0.62684400	0.05740800	0.02315300
N	0.62684200	-0.05744700	-0.02318500
C	-1.11658500	1.38033700	-0.12127800
C	-2.52047200	1.53498500	-0.22948000
C	-0.27908700	2.51193800	-0.09295300
C	-3.04062600	2.83978900	-0.28520200
C	-0.81912900	3.78994400	-0.15776400
H	0.79037800	2.35835700	-0.00661900
C	-2.20728900	3.95592400	-0.24952300
H	-4.11398400	2.97148800	-0.38310200
H	-0.16544300	4.65694900	-0.13271800
H	-2.63683100	4.95209000	-0.30139200
C	-3.45725800	0.37989300	-0.30320600
C	-4.56954800	0.33535400	0.55354700
C	-3.29074900	-0.64685900	-1.24915100
C	-5.46317500	-0.72226900	0.44878000
H	-4.73077500	1.09748100	1.30839800
C	-4.21147100	-1.69198800	-1.33443200
H	-2.44538900	-0.61497800	-1.92710400
C	-5.31551600	-1.74528900	-0.47793500
H	-4.07337900	-2.47217200	-2.07706900
H	6.04296600	2.54833400	0.52253400
H	-6.04300900	-2.54826600	-0.52282400
H	2.44537200	0.61512100	1.92698400
F	6.52845500	0.75499200	-1.29647700

F	-6.52847600	-0.75507400	1.29634100

Atomic coordinates of optimized **2i** (N_{imag} = 0)

0 1

C	4.55780900	0.00349500	-0.85495300
C	3.49785900	-0.10962100	0.06102400
C	3.31002800	0.92514200	0.99397800
C	4.15397200	2.03648800	1.00751000
C	5.20031300	2.14006200	0.08574900
C	5.39855800	1.11883000	-0.84661000
H	4.70975400	-0.77949500	-1.59259600
H	3.99755300	2.82038500	1.74346900
H	6.20495100	1.19026300	-1.57113400
C	2.64374400	-1.32812400	0.06424800
C	3.25462000	-2.59493700	0.09523100
C	1.22965000	-1.28650200	0.05394500
C	2.50512800	-3.76620600	0.12829300
H	4.33883100	-2.65198100	0.11988700
C	0.48095900	-2.47931900	0.09491700
C	1.09812500	-3.72627500	0.13413800
H	3.01581000	-4.72589600	0.15981300
H	-0.60066800	-2.39941800	0.08340400
N	-0.62662400	0.00877900	0.06621400
N	0.62662400	-0.00876400	-0.06624000
C	-1.22965700	1.28651700	-0.05394900
C	-2.64375000	1.32812900	-0.06424800
C	-0.48097500	2.47934000	-0.09492100
C	-3.25463800	2.59493600	-0.09523400
C	-1.09815200	3.72629100	-0.13413600
H	0.60065300	2.39944800	-0.08341900
C	-2.50515500	3.76621100	-0.12829600
H	-4.33884900	2.65196900	-0.11989500
H	-3.01584500	4.72589700	-0.15982300
C	-3.49785200	0.10961600	-0.06102500
C	-4.55780100	-0.00351400	0.85495100
C	-3.31000300	-0.92514500	-0.99397700
C	-5.39853500	-1.11886000	0.84660600
H	-4.70975800	0.77947500	1.59259300
C	-4.15393200	-2.03650300	-1.00751000
H	-2.50727000	-0.84773900	-1.71954600
C	-5.20027400	-2.14009000	-0.08575200

H	-6.20492800	-1.19030500	1.57112900
H	-3.99750000	-2.82039900	-1.74346700
H	2.50729800	0.84774600	1.71955100
C	-0.29062700	5.00317500	-0.17571700
H	-0.49009500	5.62811900	0.70295200
H	0.78199100	4.79376700	-0.20322300
H	-0.53952400	5.60071200	-1.06050300
C	0.29059100	-5.00315200	0.17575200
H	-0.78203500	-4.79374000	0.20293100
H	0.49029800	-5.62827300	-0.70273500
H	0.53924800	-5.60050900	1.06072800
H	-5.85527200	-3.00656700	-0.09635000
H	5.85532300	3.00653000	0.09634600

Atomic coordinates of optimized **2j** (N_{imag} = 0)

0 1

C	4.54704200	0.08452300	-0.85982500
C	3.49736900	-0.05003700	0.06473300
C	3.29361400	0.97952800	0.99981200
C	4.11193100	2.10982300	1.00559500
C	5.14796800	2.23541600	0.07526900
C	5.36265500	1.21822700	-0.85780600
H	4.70984900	-0.69428700	-1.59962200
H	3.94230000	2.89185700	1.74023100
H	6.16117200	1.30776300	-1.58880300
C	2.66662500	-1.28468300	0.07145100
C	3.30400600	-2.53869400	0.10339700
C	1.24994200	-1.26316400	0.05927000
C	2.58456100	-3.73050300	0.13327400
H	4.38844800	-2.57366400	0.12851600
C	0.51089300	-2.46153300	0.10009400
C	1.19266800	-3.66027200	0.13612700
H	3.07941100	-4.69511600	0.16397300
H	-0.57185700	-2.43269800	0.08953800
N	-0.62679700	-0.00376800	0.06126300
N	0.62679500	0.00375600	-0.06128400
C	-1.24994000	1.26315700	-0.05926200
C	-2.66662300	1.28468200	-0.07144300
C	-0.51088700	2.46152400	-0.10006100
C	-3.30399800	2.53869600	-0.10336300
C	-1.19265600	3.66026700	-0.13606800
H	0.57186400	2.43268500	-0.08950500

C	-2.58455000	3.73050300	-0.13321400
H	-4.38844000	2.57367100	-0.12848200
H	-3.07939600	4.69511800	-0.16389300
C	-3.49737300	0.05003900	-0.06475400
C	-4.54704400	-0.08453800	0.85980300
C	-3.29362500	-0.97950400	-0.99985800
C	-5.36266300	-1.21823800	0.85775800
H	-4.70984600	0.69425500	1.59962000
C	-4.11194800	-2.10979400	-1.00566700
H	-2.49989200	-0.88420700	-1.73336000
C	-5.14798400	-2.23540500	-0.07534200
H	-6.16118000	-1.30778800	1.58875500
H	-3.94232300	-2.89181200	-1.74032300
H	2.49988000	0.88424500	1.73331400
H	-5.78187900	-3.11726100	-0.07901600
H	5.78185900	3.11727500	0.07892300
F	-0.48500500	4.82100500	-0.17030900
F	0.48502100	-4.82101300	0.17039300

Atomic coordinates of optimized **2k** (N_{imag} = 0)

0 1

C	-4.41133500	-1.19657500	0.85162300
C	-3.34967600	-1.01866000	-0.04982000
C	-3.44023300	0.04410500	-0.96533700
C	-4.54687800	0.89181400	-0.97228100
C	-5.60307400	0.72152900	-0.06395400
C	-5.51397100	-0.33989300	0.84591300
H	-4.36541400	-2.00315200	1.57817100
H	-4.59465700	1.69636500	-1.70284500
H	-6.31581400	-0.49995600	1.56292100
C	-2.20744100	-1.97081700	-0.06005800
C	-2.46564200	-3.35367100	-0.09388100
C	-0.85272400	-1.56208800	-0.05322500
C	-1.43681700	-4.28871000	-0.13145800
H	-3.49737500	-3.69183400	-0.11713800
C	0.18121400	-2.51830600	-0.10021900
C	-0.08895900	-3.88295800	-0.14092700
H	-1.67952900	-5.34839100	-0.16424700
H	1.20446900	-2.15856600	-0.09145500
N	0.60204500	0.17216500	-0.07189500
N	-0.60204100	-0.17218400	0.07166800

C	0.85272000	1.56206700	0.05299000
C	2.20743700	1.97080500	0.05996300
C	-0.18123000	2.51828100	0.09979400
C	2.46562000	3.35366400	0.09372500
C	0.08892600	3.88293800	0.14044700
H	-1.20448100	2.15853300	0.09091700
C	1.43678300	4.28869700	0.13111500
H	3.49734600	3.69184000	0.11709000
H	1.67948500	5.34838300	0.16385800
C	3.34968100	1.01866100	0.04992400
C	4.41145800	1.19654400	-0.85137800
C	3.44012800	-0.04407100	0.96550400
C	5.51410500	0.33986800	-0.84548200
H	4.36562400	2.00307800	-1.57797900
C	4.54677600	-0.89176400	0.97263500
H	2.64527800	-0.19364300	1.68859400
C	5.60310100	-0.72150400	0.06444400
H	6.31603700	0.49989800	-1.56239600
H	4.59445500	-1.69628800	1.70323700
H	-2.64547700	0.19370000	-1.68852700
C	-6.78370600	1.66477700	-0.05668300
H	-7.65789800	1.20725400	0.41640100
H	-6.55318300	2.58319800	0.49836900
H	-7.06482700	1.96173300	-1.07248800
C	6.78377000	-1.66470800	0.05746400
H	6.55271000	-2.58406100	-0.49581300
H	7.65743400	-1.20790700	-0.41728000
H	7.06598600	-1.95998100	1.07346400
C	-1.02373300	4.90479100	0.18797400
H	-0.99177700	5.56828200	-0.68447800
H	-2.00463600	4.42245000	0.20811900
H	-0.94224400	5.53847700	1.07894600
C	1.02368900	-4.90481500	-0.18865600
H	2.00458900	-4.42247600	-0.20900500
H	0.99190700	-5.56828700	0.68381700
H	0.94202100	-5.53852000	-1.07959800

Atomic coordinates of optimized **2l** (N_{imag} = 0)

0 1

C	4.42358000	-1.07659900	-0.86682700
C	3.37118900	-0.93563400	0.05388300

C	3.43760700	0.11779800	0.98291500
C	4.51440000	1.00487200	0.99481200
C	5.52810600	0.83104300	0.06024400
C	5.50668600	-0.19468100	-0.87485600
H	4.38627400	-1.87428700	-1.60268400
H	4.57680800	1.81292200	1.71590800
H	6.31522800	-0.29255700	-1.59122000
C	2.25354600	-1.91752200	0.06410400
C	2.54712100	-3.29292600	0.09195100
C	0.88946400	-1.54150900	0.05964700
C	1.54157300	-4.25314900	0.12743300
H	3.58683000	-3.60602400	0.11273200
C	-0.12001900	-2.52338100	0.10489700
C	0.18397300	-3.88115200	0.14071300
H	1.81051000	-5.30643700	0.15673900
H	-1.15204300	-2.18998400	0.10063300
N	-0.60810100	0.15681500	0.05374300
N	0.60812900	-0.15666600	-0.05375600
C	-0.88949500	1.54165500	-0.05962700
C	-2.25359700	1.91758000	-0.06410800
C	0.11992200	2.52359300	-0.10489200
C	-2.54727100	3.29296200	-0.09202200
C	-0.18416200	3.88134400	-0.14073700
H	1.15196800	2.19026600	-0.10064400
C	-1.54178600	4.25325100	-0.12751500
H	-3.58700100	3.60598500	-0.11285300
H	-1.81079400	5.30651900	-0.15688700
C	-3.37116200	0.93560400	-0.05388700
C	-4.42361500	1.07653600	0.86675700
C	-3.43742200	-0.11790500	-0.98284200
C	-5.50664200	0.19452200	0.87478300
H	-4.38642300	1.87428100	1.60255800
C	-4.51413200	-1.00507900	-0.99473700
H	-2.64452700	-0.23606500	-1.71294500
C	-5.52791400	-0.83127100	-0.06024600
H	-6.31523400	0.29237400	1.59109300
H	-4.57642000	-1.81319300	-1.71577200
H	2.64477700	0.23596900	1.71308600
F	-6.57934300	-1.69600000	-0.06418700
F	6.57961800	1.69567200	0.06418800
C	0.90215300	4.93084400	-0.18767200

H	0.85959700	5.58566700	0.69073700
H	1.89496500	4.47441600	-0.21926000
H	0.79756800	5.56973300	-1.07229500
C	-0.90240500	-4.93057900	0.18779000
H	-1.89524000	-4.47409900	0.21785800
H	-0.85897200	-5.58646800	-0.68977200
H	-0.79872400	-5.56839600	1.07330100

Atomic coordinates of optimized **2m** (N_{imag} = 0)

0 1

C	-2.73957200	1.12757300	0.14954500
C	-3.44712600	2.34572500	0.19732700
C	-1.32115000	1.18588400	0.09187800
C	-2.78891900	3.57123100	0.20412100
H	-4.53061700	2.31996300	0.25838300
C	-0.66441900	2.43180400	0.12081700
C	-1.38813700	3.61495000	0.17503000
H	-3.36468800	4.49112100	0.24609000
H	0.41873300	2.43895100	0.08676700
H	-0.86957100	4.56906000	0.19162500
N	0.62625500	0.03676200	0.06606500
N	-0.62627100	-0.03685300	-0.06600700
C	1.32116600	-1.18594600	-0.09191500
C	2.73958300	-1.12757600	-0.14955700
C	0.66448200	-2.43188800	-0.12099900
C	3.44718900	-2.34569100	-0.19747100
C	1.38824900	-3.61500000	-0.17533000
H	-0.41866900	-2.43908200	-0.08697500
C	2.78903000	-3.57122300	-0.20440500
H	4.53068000	-2.31987600	-0.25851000
H	0.86972100	-4.56912800	-0.19203700
H	3.36483500	-4.49108500	-0.24647000
C	-3.47938700	-0.14279300	0.18624800
C	-3.20790900	-1.29684600	0.88883900
S	-4.98250400	-0.32255500	-0.70394400
C	-4.19276100	-2.31332400	0.72210600
H	-2.33619200	-1.39909300	1.52247700
C	-5.21205000	-1.93376800	-0.11238700
H	-4.14808100	-3.28250100	1.20611100
H	-6.07789300	-2.50480500	-0.41870000
C	3.47933000	0.14283300	-0.18610200

C	3.20754700	1.29710100	-0.88822000
S	4.98272700	0.32239000	0.70365700
C	4.19240400	2.31357500	-0.72149400
H	2.33560200	1.39951100	-1.52151900
C	5.21199500	1.93380200	0.11253300
H	4.14751000	3.28290700	-1.20517000
H	6.07790900	2.50478700	0.41874200

Atomic coordinates of optimized **2n** (N_{imag} = 0)

0 1

C	2.39905600	1.59471300	-0.64245300
C	2.87430100	2.90613800	-0.80527200
C	1.04428200	1.41233200	-0.28440300
C	2.03591500	4.00556600	-0.62557200
H	3.91263200	3.05536900	-1.08666800
C	0.19920800	2.52458500	-0.10581300
C	0.69273500	3.81203100	-0.27503800
H	2.42615200	5.01025500	-0.76041900
H	-0.83383100	2.34954400	0.17202000
N	-0.62026100	-0.07369900	0.07661500
N	0.62025900	0.07369700	-0.07661400
C	-1.04428400	-1.41233400	0.28440400
C	-2.39905700	-1.59471500	0.64245300
C	-0.19921000	-2.52458600	0.10581400
C	-2.87430300	-2.90614100	0.80527200
C	-0.69273700	-3.81203300	0.27503800
H	0.83383000	-2.34954600	-0.17201900
C	-2.03591600	-4.00556900	0.62557200
H	-3.91263400	-3.05537200	1.08666800
H	-2.42615300	-5.01025700	0.76041800
H	0.03742200	4.66648300	-0.13228300
H	-0.03742300	-4.66648400	0.13228300
C	3.31394400	0.43975100	-0.89807000
C	4.37356400	0.11953400	0.01393900
C	3.16410300	-0.30524800	-2.05478800
C	4.57102600	0.81504600	1.24100300
C	5.26458000	-0.96086000	-0.30646500
C	4.04181100	-1.37056600	-2.36513400
H	2.35933500	-0.06007100	-2.74142200
C	5.59896300	0.47615600	2.09508900
H	3.89349500	1.61975200	1.50545500

C	6.31728500	-1.28069300	0.59547900
C	5.07361500	-1.68975700	-1.51129000
H	3.89774700	-1.93015700	-3.28487700
C	6.48587200	-0.57920600	1.76890900
H	5.72812300	1.01897500	3.02705400
H	6.98882700	-2.09707900	0.34153900
H	5.75484600	-2.50364300	-1.74608000
H	7.29352700	-0.83615800	2.44819900
C	-3.31394600	-0.43975400	0.89807200
C	-4.37356300	-0.11953300	-0.01393900
C	-3.16410800	0.30524100	2.05479300
C	-4.57102100	-0.81503900	-1.24100800
C	-5.26457900	0.96086100	0.30646600
C	-4.04181700	1.37055800	2.36514000
H	-2.35934200	0.06006100	2.74142800
C	-5.59895500	-0.47614500	-2.09509600
H	-3.89349000	-1.61974500	-1.50546100
C	-6.31728100	1.28069800	-0.59548100
C	-5.07361700	1.68975300	1.51129400
H	-3.89775600	1.93014600	3.28488500
C	-6.48586500	0.57921500	-1.76891400
H	-5.72811200	-1.01896100	-3.02706400
H	-6.98882400	2.09708300	-0.34154000
H	-5.75484900	2.50363900	1.74608400
H	-7.29351700	0.83617100	-2.44820500

Atomic coordinates of optimized **2o** (N_{imag} = 0)

0 1

C	2.16418100	1.99711200	-0.29345700
C	2.41641500	3.37896600	-0.35756800
C	0.81918100	1.57258700	-0.15726000
C	1.38159700	4.31047700	-0.30037200
H	3.44098200	3.71702500	-0.47942900
C	-0.22345800	2.51772800	-0.10843900
C	0.05463500	3.87668100	-0.18040500
H	1.60660600	5.37146700	-0.35976600
H	-1.24041900	2.15801700	-0.00206200
N	-0.60396000	-0.17823600	0.01723900
N	0.60393100	0.17822500	-0.01724000
C	-0.81920500	-1.57260600	0.15719200
C	-2.16419800	-1.99714900	0.29340900

C	0.22343900	-2.51773600	0.10826800
C	-2.41641700	-3.37901100	0.35742700
C	-0.05464000	-3.87669500	0.18014900
H	1.24039400	-2.15801000	0.00187400
C	-1.38159500	-4.31051000	0.30012900
H	-3.44097600	-3.71708700	0.47930600
H	-1.60659500	-5.37150600	0.35945800
H	-0.75554500	4.59905800	-0.14112600
H	0.75554400	-4.59906400	0.14078900
C	3.30634000	1.04644700	-0.38217300
C	4.41207700	1.20606300	0.43560100
C	3.31334200	-0.00840700	-1.34299300
C	5.53839500	0.34381000	0.34864600
C	4.39260100	-0.85294000	-1.45978000
H	2.45675700	-0.12944700	-1.99705300
C	5.53291200	-0.71071100	-0.62115100
H	4.38561200	-1.64376600	-2.20582700
C	-3.30636700	-1.04650600	0.38224000
C	-4.41197200	-1.20586200	-0.43576500
C	-3.31353200	0.00803900	1.34339900
C	-5.53830500	-0.34363900	-0.34872000
C	-4.39281200	0.85253300	1.46027900
H	-2.45706000	0.12886600	1.99764400
C	-5.53298400	0.71057300	0.62141600
H	-4.38594800	1.64311600	2.20658500
H	-4.42007000	-1.99532400	-1.18319300
H	4.42030000	1.99576500	1.18277400
C	6.66178700	-1.56948700	-0.71123100
C	6.67275300	0.49301100	1.19414600
C	7.74777500	-1.39806000	0.12120000
H	8.60419300	-2.06150100	0.04272900
C	7.75265900	-0.35686900	1.08377100
H	8.61267000	-0.23155900	1.73527000
C	-6.67252300	-0.49257000	-1.19445400
C	-6.66187600	1.56931600	0.71158500
C	-7.74772900	1.39815000	-0.12107700
C	-7.75245200	0.35726900	-1.08398300
H	-8.61235400	0.23216400	-1.73566500
H	-6.67466300	-1.29085400	-1.93244700
H	-8.60416100	2.06156600	-0.04253000
H	-6.65593500	2.36685700	1.45040400

H	6.65571900	-2.36726500	-1.44979400
H	6.67501800	1.29153200	1.93188200

Atomic coordinates of optimized **2p** (N_{imag} = 0)

0 1

C	4.11777800	1.20074500	0.73571900
C	3.03757100	1.03925600	-0.14175000
C	3.08743500	-0.04038200	-1.04606900
C	4.16544400	-0.91535200	-1.06653900
C	5.23312600	-0.74305500	-0.17151200
C	5.20718600	0.32282400	0.73519200
H	4.10366900	2.01414000	1.45573800
H	4.20579900	-1.73850600	-1.77276900
H	6.01275600	0.47871800	1.44281500
C	1.91499400	2.01325800	-0.13891000
C	2.20170300	3.39040700	-0.18039800
C	0.55198300	1.62285500	-0.10936900
C	1.18936500	4.34671600	-0.20304600
H	3.24000900	3.70531200	-0.22277900
C	-0.46770700	2.59422800	-0.14552900
C	-0.15365300	3.94586300	-0.19199500
H	1.44438000	5.40190800	-0.24182700
H	-1.49818700	2.25902800	-0.12253300
N	-0.92542000	-0.09257100	-0.08997200
N	0.28525800	0.23780500	0.02645700
C	-1.18641800	-1.47995700	0.04739800
C	-2.54641900	-1.87657000	0.08254800
C	-0.16445500	-2.44835400	0.08104800
C	-2.83187300	-3.25273200	0.13122400
C	-0.47565400	-3.80091600	0.13472000
H	0.86509000	-2.11080200	0.05065700
C	-1.81658300	-4.20657300	0.15493300
H	-3.86944900	-3.56995300	0.17667500
H	-2.06832600	-5.26224300	0.19887000
C	-3.67298400	-0.90396900	0.08313300
C	-4.73986500	-1.06257100	-0.81755100
C	-3.73078500	0.15810200	1.00265000
C	-5.82977100	-0.18911300	-0.81498700
H	-4.70886600	-1.86701000	-1.54628300
C	-4.81428800	1.03667100	1.02521100
H	-2.92594300	0.29022300	1.71718100

C	-5.84283600	0.84547800	0.11048100
H	-6.64982400	-0.29994400	-1.51619800
H	-4.86963900	1.85222100	1.73833400
H	2.27647700	-0.18240500	-1.75221800
H	-0.94674200	4.68757200	-0.21639900
H	0.31996700	-4.53999600	0.15680100
O	6.24298400	-1.65953900	-0.26507600
C	7.35855400	-1.53308000	0.60801200
H	7.05602800	-1.62067900	1.65913400
H	8.02888600	-2.35538900	0.35503000
H	7.87925600	-0.57923900	0.45593100
F	-6.90102000	1.70156100	0.12459700

Atomic coordinates of optimized **2q** (N_{imag} = 0)

0 1

C	4.15442600	-1.78695500	-0.87461000
C	3.15794500	-1.48220200	0.06602100
C	3.41055200	-0.46082000	0.99898300
C	4.61683500	0.23331500	0.98923600
C	5.59658200	-0.07854000	0.03854600
C	5.36292000	-1.08968800	-0.89589300
H	3.97545600	-2.56498200	-1.61045400
H	4.80143700	1.00972500	1.72466700
H	6.11802600	-1.33319100	-1.63505700
C	1.89574000	-2.27088500	0.09622800
C	1.96957400	-3.67367500	0.14423200
C	0.61355100	-1.66850200	0.08982000
C	0.81972400	-4.45881500	0.20018400
H	2.94667300	-4.14684300	0.16390200
C	-0.54418500	-2.46733600	0.15232000
C	-0.44277700	-3.85140400	0.20914400
H	0.90641000	-5.54037800	0.24578800
H	-1.51034600	-1.97648200	0.14487900
N	-0.57349800	0.25727300	0.04102300
N	0.57350500	-0.25733200	-0.04092000
C	-0.61353200	1.66844400	-0.08969200
C	-1.89571400	2.27084600	-0.09612800
C	0.54421700	2.46726200	-0.15212300
C	-1.96952300	3.67363900	-0.14409200
C	0.44283300	3.85133400	-0.20891100
H	1.51037200	1.97639400	-0.14465700

C	-0.81966000	4.45876300	-0.19998100
H	-2.94661400	4.14682200	-0.16378700
H	-0.90632700	5.54032800	-0.24555700
C	-3.15793400	1.48218400	-0.06598500
C	-4.15443900	1.78693300	0.87462200
C	-3.41053600	0.46083400	-0.99898600
C	-5.36295000	1.08969000	0.89584900
H	-3.97547600	2.56493500	1.61049200
C	-4.61683300	-0.23327400	-0.98929400
H	-2.66132000	0.22176500	-1.74539500
C	-5.59660300	0.07857200	-0.03862300
H	-6.11807200	1.33318600	1.63499800
H	-4.80143000	-1.00965900	-1.72475400
H	2.66135100	-0.22174200	1.74540500
H	-1.34144300	-4.45927300	0.25707700
H	1.34150900	4.45919200	-0.25679100
C	6.87479100	0.71369800	0.00927900

Atomic coordinates of optimized **2r** (N_{imag} = 0)

0 1

C	3.00772800	1.41339800	-0.78950200
C	2.12290600	0.74507100	0.07483800
C	1.39677300	1.50778400	1.00613900
C	1.55105600	2.89345300	1.06909900
C	2.42962400	3.54624300	0.19956300
C	3.15729700	2.80043900	-0.73133000
H	3.56455300	0.84290800	-1.52745600
H	0.98591700	3.46319100	1.80136200
H	3.83829400	3.29742200	-1.41649500
C	2.00998400	-0.73735300	0.01900400
C	3.18151100	-1.51549600	0.02258200
C	0.76575900	-1.41419600	-0.02234100
C	3.13258300	-2.90757700	-0.01214100
H	4.14210500	-1.01228600	0.07718800
C	0.72188000	-2.82055300	-0.05592400
C	1.89548500	-3.56376000	-0.05012200
H	4.05539700	-3.48042200	-0.00565700
H	-0.24747700	-3.30299500	-0.10703300
H	1.85182700	-4.64851800	-0.08343500
N	-1.48209700	-1.21044500	0.14228400
N	-0.40216000	-0.61658300	-0.11452700

C	-2.65124600	-0.41902600	-0.01030900
C	-3.85272900	-1.04271200	0.35592500
C	-2.66368600	0.90203400	-0.49527000
C	-5.06187400	-0.35428300	0.24687200
C	-3.87396100	1.58073400	-0.60295800
H	-1.72769300	1.36859800	-0.78041100
C	-5.07482100	0.95819400	-0.23246800
H	-5.98987200	-0.84020900	0.53323600
H	-3.88743900	2.59977800	-0.97926400
H	-6.01438700	1.49591100	-0.32148700
H	2.54678800	4.62501700	0.24818300
H	0.72091500	1.00966500	1.69272900
H	-3.81472600	-2.06323200	0.72431100

Atomic coordinates of optimized **2s** (N_{imag} = 0)

0 1

C	-3.05152600	-0.42883600	-0.81143500
C	-2.02948300	0.01062800	0.04607700
C	-1.49371200	-0.91780300	0.95535700
C	-1.96282900	-2.22986600	0.99946800
C	-2.97635500	-2.67237300	0.13610800
C	-3.51219600	-1.74568200	-0.76821000
H	-3.47663700	0.26178300	-1.53440100
H	-1.53651800	-2.92123800	1.72273700
H	-4.29876600	-2.05686400	-1.45161300
C	-1.58229300	1.42798700	0.01058400
C	-2.54557500	2.45322700	0.01953000
C	-0.21633700	1.80548300	-0.01555100
C	-2.18118200	3.79781700	0.00367800
H	-3.59587800	2.18156700	0.06385000
C	0.14651500	3.16547100	-0.02835800
C	-0.82697100	4.15620500	-0.01852800
H	-2.94970800	4.56537800	0.01344900
H	1.20063800	3.41512900	-0.06771000
H	-0.53753100	5.20293400	-0.03642700
N	1.92515100	1.09414100	0.15932700
N	0.74119000	0.76587800	-0.11582400
C	2.88547700	0.06068000	-0.00392200
C	4.19324300	0.38666300	0.38364300
C	2.60276800	-1.21813600	-0.51887300
C	5.21532900	-0.55645400	0.26625700

C	3.62818200	-2.15209800	-0.63469900
H	1.58837400	-1.45324300	-0.82023800
C	4.93495400	-1.82718700	-0.24284800
H	6.22627000	-0.30048400	0.56927200
H	3.41387100	-3.13938500	-1.03426200
H	5.72857700	-2.56262800	-0.33854700
H	-0.71827400	-0.60393300	1.64574600
H	4.38413800	1.38118700	0.77487800
C	-3.45521400	-4.10518200	0.16692200
H	-4.46077900	-4.19992800	-0.25402600
H	-2.79199700	-4.75598300	-0.41723500
H	-3.47692900	-4.49769100	1.18881100

Atomic coordinates of optimized **2t** (N_{imag} = 0)

0 1

C	-3.03544000	-0.54428800	-0.81913400
C	-2.03620600	-0.07446200	0.05118400
C	-1.47294800	-0.98041700	0.96740400
C	-1.88880900	-2.31129700	1.01682800
C	-2.87497000	-2.73184400	0.13311900
C	-3.46015700	-1.87399200	-0.78868000
H	-3.47200800	0.13280900	-1.54711000
H	-1.46383900	-3.01251700	1.72691300
H	-4.22196400	-2.24393900	-1.46635300
C	-1.63365700	1.35696000	0.01518700
C	-2.62863200	2.35085300	0.02461800
C	-0.27989800	1.77469900	-0.01546400
C	-2.30561900	3.70607300	0.00423300
H	-3.67013600	2.04752100	0.07279700
C	0.04064600	3.14500600	-0.03351100
C	-0.96312100	4.10519600	-0.02328800
H	-3.09715700	4.44972300	0.01463100
H	1.08639700	3.42706500	-0.07603800
H	-0.70595900	5.16019800	-0.04451800
N	1.88338400	1.13057100	0.14797700
N	0.70755600	0.76243700	-0.11118700
C	2.87468800	0.12663600	-0.00902600
C	4.17178700	0.49599700	0.37586400
C	2.63207000	-1.16309700	-0.51733100
C	5.22253900	-0.41567200	0.26429400
C	3.68593200	-2.06532100	-0.62793500

H	1.62616900	-1.43160600	-0.81912000
C	4.98169600	-1.69743000	-0.23735000
H	6.22500200	-0.12672000	0.56565100
H	3.50270700	-3.06072800	-1.02254800
H	5.79783300	-2.40829500	-0.32878900
H	-0.71179700	-0.63836700	1.65938200
H	4.33179000	1.49842200	0.76072700
F	-3.28255800	-4.02958000	0.17342000

Atomic coordinates of optimized **3a cation** (N_{imag} = 0)

1 1

C	2.21733200	1.41768600	2.16267300
C	1.83499700	0.49114700	1.16379400
C	0.49996300	-0.00818100	1.19377900
C	-0.40350900	0.38675100	2.20603000
C	0.01219100	1.28638000	3.16514500
C	1.32450300	1.80993000	3.13946300
H	3.22345500	1.81892400	2.16633300
H	-1.40629800	-0.01869800	2.22602400
H	1.63456400	2.51930000	3.89973900
C	2.71527300	0.00525000	0.13772200
C	4.06214100	0.39036400	-0.03342600
C	2.17614000	-0.96040400	-0.77005700
C	4.82222200	-0.16373800	-1.04924600
H	4.51253400	1.12050100	0.62822100
C	2.97200700	-1.52100300	-1.80772200
C	4.28186300	-1.12412900	-1.94070000
H	5.85686700	0.14377800	-1.16525300
H	2.51620900	-2.25014500	-2.46875300
H	4.90443000	-1.54045700	-2.72533400
N	0.10925300	-0.91494600	0.20781300
N	0.89063000	-1.38323800	-0.71396100
C	-1.25015900	-1.46040000	0.17755500
C	-2.31829400	-0.67856100	-0.29687400
C	-1.41160300	-2.76560600	0.64308600
C	-3.59113800	-1.27548300	-0.25254300
C	-2.68683600	-3.32764600	0.66444400
H	-0.54943000	-3.32610100	0.99013500
C	-3.77687600	-2.57442400	0.22091300
H	-4.43905700	-0.71084400	-0.62680200
H	-2.82470700	-4.34196700	1.02355400

H	-4.77360400	-3.00395200	0.23047900
C	-2.15638300	0.70174500	-0.83385600
C	-1.27084200	0.97289300	-1.89147400
C	-2.93754900	1.75096600	-0.31978200
C	-1.16425600	2.26365800	-2.41365400
H	-0.69754500	0.16464400	-2.33571600
C	-2.82551100	3.04162900	-0.84002900
H	-3.63141300	1.55620900	0.49330000
C	-1.93691000	3.30202000	-1.88658400
H	-0.49133800	2.45395800	-3.24442000
H	-3.43505900	3.84143100	-0.43075100
H	-0.67531700	1.59166500	3.94683100
H	-1.85606900	4.30397500	-2.29648500

Atomic coordinates of optimized **3b cation** (N_{imag} = 0)

1 1

C	2.12114200	0.84213600	2.42379900
C	1.90776600	0.21759900	1.17098300
C	0.68945300	-0.49992300	0.99645900
C	-0.25298600	-0.60846700	2.04273300
C	-0.01846500	0.00440600	3.26109400
C	1.18697400	0.74067600	3.43021800
H	3.03267200	1.40115300	2.59774100
H	-1.16035600	-1.17744900	1.88694400
H	1.37657600	1.22686500	4.38294600
C	2.84384600	0.24147400	0.08570200
C	4.09309800	0.90105800	0.09631100
C	2.47851100	-0.47900800	-1.09591100
C	4.92494900	0.83543600	-1.00698800
H	4.40972800	1.46081000	0.96825000
C	3.34846500	-0.53703300	-2.22133500
C	4.55793200	0.11383500	-2.17177600
H	5.88192100	1.34736200	-0.98092500
H	3.02564500	-1.09726500	-3.09213800
H	5.23407700	0.08090200	-3.01931000
N	0.45683800	-1.11287600	-0.23448500
N	1.29631100	-1.12447300	-1.22420800
C	-0.77667700	-1.86085700	-0.48727000
C	-1.98106700	-1.18096600	-0.74237900
C	-0.68188100	-3.25239900	-0.45358600
C	-3.11753300	-1.98757500	-0.93705200

C	-1.82737000	-4.01913800	-0.65895500
H	0.27677800	-3.72313900	-0.26033800
C	-3.04754300	-3.37988400	-0.89403600
H	-4.06290900	-1.50133800	-1.15555100
H	-1.76506300	-5.10191500	-0.63574700
H	-3.94534500	-3.96704600	-1.05936900
C	-2.09411700	0.30122800	-0.81852600
C	-1.28166200	1.05987500	-1.67745100
C	-3.06773600	0.97489100	-0.06143800
C	-1.43557900	2.44407000	-1.76596100
H	-0.55294900	0.56378200	-2.31168500
C	-3.21188100	2.35876500	-0.15167400
H	-3.71126700	0.41297900	0.61014200
C	-2.40122900	3.12111400	-1.00676200
H	-0.80562700	3.00521800	-2.45135700
H	-3.96986700	2.85403800	0.44956800
C	-1.00297700	-0.09593600	4.39561300
H	-0.53309100	-0.54075400	5.27978200
H	-1.36015700	0.89834800	4.68665300
H	-1.86905800	-0.70444800	4.12711000
C	-2.59151700	4.61316300	-1.13405300
H	-3.37920100	4.84298300	-1.86203600
H	-2.88882500	5.06200300	-0.18164800
H	-1.67723100	5.10713500	-1.47472800

Atomic coordinates of optimized **3c cation** (N_{imag} = 0)

1 1

C	-2.27220300	2.03332100	-1.08560800
C	-1.94837000	0.75261600	-0.58259800
C	-0.70412500	0.17575200	-0.96803700
C	0.16167000	0.86059500	-1.85171000
C	-0.20053900	2.10191500	-2.32109700
C	-1.42455800	2.71913800	-1.94060800
H	-3.21268700	2.49113900	-0.80197000
H	1.09573500	0.40735900	-2.15614700
C	-2.80658100	-0.01567000	0.27650800
C	-4.06860400	0.40010000	0.75374100
C	-2.34141200	-1.31243300	0.66126900
C	-4.81984800	-0.43586200	1.56142400
H	-4.45972100	1.37552100	0.49059800
C	-3.12765500	-2.15987900	1.49052200

C	-4.35401600	-1.72196500	1.93247800
H	-5.78831100	-0.09937000	1.91826100
H	-2.73045000	-3.13442700	1.75290600
H	-4.96685200	-2.35456400	2.56562300
N	-0.37036000	-1.08152600	-0.47298700
N	-1.13625800	-1.80424200	0.28391300
C	0.89733900	-1.72294900	-0.82986200
C	2.09928700	-1.30502100	-0.23164600
C	0.83538300	-2.74414400	-1.77835100
C	3.26674800	-1.96012500	-0.66432900
C	2.01172400	-3.37611300	-2.17724600
H	-0.12243600	-3.03059800	-2.20076700
C	3.22889600	-2.97505400	-1.62032200
H	4.21264700	-1.67896800	-0.21265300
H	1.97540200	-4.17189700	-2.91360800
H	4.15066300	-3.46322500	-1.92056200
C	2.17891200	-0.24250300	0.80950400
C	1.41408600	-0.31319700	1.98415400
C	3.07436300	0.82904500	0.64410400
C	1.54456300	0.67754600	2.96048800
H	0.75528000	-1.15873700	2.15589600
C	3.21346900	1.82897600	1.61406200
H	3.67116200	0.88616700	-0.26304200
C	2.42959600	1.73950300	2.77570400
H	0.96496000	0.61029200	3.87649400
H	2.52393800	2.50130800	3.54515400
H	0.46468300	2.62404200	-3.00251800
C	-1.77359300	4.08136800	-2.47637400
H	-1.80943200	4.07030000	-3.57155900
H	-2.74139200	4.42692700	-2.10691900
H	-1.01467900	4.81728000	-2.18758500
C	4.19069600	2.96608300	1.42925600
H	3.70256000	3.93673600	1.56767300
H	5.00301600	2.90657800	2.16262500
H	4.64086900	2.95225600	0.43306000

Atomic coordinates of optimized **3d cation** (N_{imag} = 0)

1 1

C	2.38008600	-1.88277000	-1.35765400
C	1.91173100	-0.64283200	-0.80194400
C	0.55618600	-0.26595900	-1.09593800

C	-0.28104900	-1.03691900	-1.92747000
C	0.20619800	-2.20855700	-2.45733200
C	1.51607300	-2.62026600	-2.15950500
H	-1.28435200	-0.69954700	-2.14631400
C	2.67723600	0.28207100	0.00922000
C	4.02161400	0.14207300	0.43252000
C	2.01498100	1.48909500	0.42302300
C	4.64605600	1.12414400	1.18554500
H	4.59364800	-0.73222500	0.17764200
C	2.67114000	2.48822200	1.19376100
C	3.98002400	2.30888500	1.57041700
H	5.67858300	0.97308700	1.48526000
H	2.10316900	3.37155000	1.46466500
H	4.49643600	3.06021300	2.15789900
N	0.04344000	0.91616100	-0.56061200
N	0.72172800	1.76221200	0.14061600
C	-1.34863500	1.32294800	-0.78671200
C	-2.37999300	0.76878400	-0.00858100
C	-1.56877000	2.29108300	-1.76670400
C	-3.67735200	1.23842500	-0.27811900
C	-2.86933600	2.73032900	-2.00749000
H	-0.73173100	2.69080300	-2.33037400
C	-3.92317200	2.19919800	-1.25911200
H	-4.49969500	0.85285700	0.31563400
H	-3.05418100	3.48310200	-2.76647500
H	-4.93843700	2.54198200	-1.43182700
C	-2.13494900	-0.22493900	1.08181700
C	-1.36342100	0.17072500	2.18998200
C	-2.69441300	-1.52355800	1.04835900
C	-1.13025000	-0.69704400	3.25570600
H	-0.97202700	1.18323200	2.22674300
C	-2.43630700	-2.38378200	2.12717700
C	-1.66718800	-1.98544500	3.22015400
H	-0.54710900	-0.36562000	4.10942500
H	-1.49586700	-2.67446200	4.04159900
H	-0.41807200	-2.81528700	-3.10469700
H	-2.85509600	-3.38646800	2.10719800
H	1.87514700	-3.55349100	-2.58152500
C	-3.57226300	-2.01592400	-0.08241900
H	-3.50482700	-3.10328400	-0.17563700
H	-4.62668600	-1.77505300	0.09877400

H	-3.30752500	-1.57431000	-1.04633100
C	3.76117000	-2.45962700	-1.14370000
H	4.54754400	-1.81119400	-1.54347500
H	3.97148300	-2.64792200	-0.08609900
H	3.84105800	-3.41674700	-1.66212600

Atomic coordinates of optimized **3e cation** (N_{imag} = 0)

1 1

C	1.87244100	-1.45848700	-2.20820800
C	1.92000800	-0.73721200	-0.99192300
C	1.00794800	0.35294900	-0.84536100
C	0.12366000	0.71431400	-1.86964400
C	0.11087400	-0.01712900	-3.05345100
C	0.99480900	-1.11896800	-3.21486300
H	2.54336900	-2.29505400	-2.36195300
H	-0.55292800	1.55083400	-1.76212000
H	0.99181200	-1.69545600	-4.13157900
C	2.83242400	-1.01193100	0.07491500
C	3.79563200	-2.04693500	0.08703600
C	2.76663100	-0.14962000	1.21730000
C	4.63991900	-2.20089100	1.17086500
H	3.88048700	-2.72636500	-0.75269600
C	3.64724600	-0.32342300	2.32394400
C	4.57256600	-1.33786700	2.29596200
H	5.37342400	-3.00122400	1.16154100
H	3.55615200	0.35716100	3.16352900
H	5.25110300	-1.48620400	3.12903700
N	1.03261500	1.06974700	0.35494400
N	1.86751800	0.85268200	1.32557700
C	0.12433700	2.19432400	0.58068700
C	-1.23174300	1.96496300	0.87849000
C	0.67387800	3.47247400	0.47391800
C	-2.03091300	3.11290500	1.03676100
C	-0.14734300	4.58524000	0.64390200
H	1.72965000	3.58773000	0.25024800
C	-1.50501000	4.39891400	0.91802100
H	-3.07813900	2.98031800	1.28890900
H	0.26949300	5.58346500	0.56277000
H	-2.15389600	5.25798800	1.05646400
C	-1.82475300	0.61077100	1.03328000
C	-1.26728000	-0.34853600	1.90319800

C	-3.00326600	0.27136100	0.35305100
C	-1.85725100	-1.59366600	2.07244700
H	-0.38744000	-0.10069300	2.48913300
C	-3.60684800	-0.97855600	0.51048900
H	-3.45856500	0.98913700	-0.32372300
C	-3.03223800	-1.92249500	1.37409400
H	-1.44144300	-2.32287100	2.75996800
H	-4.51461600	-1.20060600	-0.03723800
O	-0.76544300	0.38850400	-3.98779600
O	-3.52991000	-3.16265300	1.60698800
C	-0.83847300	-0.29051900	-5.25130900
H	-1.13300200	-1.33629600	-5.11665600
H	-1.60768900	0.23586900	-5.81418900
H	0.11509200	-0.22627700	-5.78521700
C	-4.75731900	-3.54613200	0.98503300
H	-4.66223800	-3.55502000	-0.10718800
H	-4.96322800	-4.55559100	1.33986800
H	-5.57512300	-2.87975700	1.28211600

Atomic coordinates of optimized **3f cation** (N_{imag} = 0)

1 1

C	2.08248900	2.25550500	0.49899000
C	1.98221000	0.88708200	0.21799200
C	0.92836200	0.14036100	0.83017000
C	0.03244600	0.77217300	1.72151200
C	0.15651700	2.11727500	1.98680400
C	1.18449900	2.87791500	1.36537300
H	2.86247500	2.86371700	0.05870100
H	-0.75072900	0.19878600	2.19950100
C	2.89408600	0.17001900	-0.63824100
C	3.98885400	0.74280100	-1.31738000
C	2.67093100	-1.23192300	-0.79004800
C	4.81697500	-0.04549700	-2.09977700
H	4.19214900	1.80361300	-1.23073800
C	3.53010900	-2.02757500	-1.59502800
C	4.59236600	-1.43582500	-2.24062900
H	5.65582600	0.41264500	-2.61435400
H	3.31969600	-3.08829600	-1.67868200
H	5.25752500	-2.02953900	-2.85850500
N	0.81558900	-1.21458200	0.54537200

N	1.63373000	-1.88578100	-0.20331300
C	-0.25394700	-2.02316100	1.13484300
C	-1.56974900	-1.92272900	0.65060400
C	0.10682200	-2.87341100	2.18021100
C	-2.53345900	-2.71491800	1.29995600
C	-0.87307600	-3.65038200	2.79563500
H	1.14067000	-2.91675500	2.50743900
C	-2.19619400	-3.56281400	2.35501100
H	-3.55753100	-2.67660400	0.94277100
H	-0.60372700	-4.31576500	3.60902100
H	-2.96638200	-4.16620100	2.82494500
C	-1.96317500	-1.04806300	-0.49033900
C	-1.33200300	-1.15819200	-1.74434700
C	-3.01181600	-0.13800100	-0.32744100
C	-1.76030800	-0.35866200	-2.80226600
H	-0.55129300	-1.89511600	-1.90051200
C	-3.43310200	0.67003100	-1.39595700
H	-3.52102700	-0.03364700	0.62556400
C	-2.80231800	0.56055500	-2.64293100
H	-1.28941400	-0.45824400	-3.77578900
H	-3.11555900	1.16617600	-3.48443300
H	-0.53752400	2.58461800	2.67385300
O	1.36849700	4.19183900	1.56275100
O	-4.45368900	1.52301400	-1.11535500
C	0.50117600	4.92134100	2.44652000
H	0.86512200	5.94712300	2.42044900
H	-0.53362800	4.88869000	2.09132100
H	0.56963200	4.53295600	3.46757000
C	-4.98676100	2.33138700	-2.16587800
H	-5.80002100	2.89968000	-1.71514700
H	-4.23087500	3.02235500	-2.55727100
H	-5.38068800	1.71177100	-2.97943600

Atomic coordinates of optimized **3g cation** (N_{imag} = 0)

1 1

C	2.14270200	1.01143700	2.32942800
C	1.89749600	0.27530200	1.14250600
C	0.65637500	-0.41877600	1.03428200
C	-0.28782000	-0.40412000	2.08131300
C	0.01656200	0.32607000	3.20576100
C	1.21877000	1.04515400	3.35062100

H	3.07284200	1.55410300	2.44367300
H	-1.22186800	-0.94521100	2.01800800
H	1.39713200	1.60314300	4.26326900
C	2.82976700	0.16809700	0.05798300
C	4.09847600	0.78497700	0.00422500
C	2.43557200	-0.64741300	-1.05224600
C	4.92216600	0.58972300	-1.09063200
H	4.43911900	1.41374500	0.81807300
C	3.29779700	-0.83809600	-2.16944400
C	4.52753500	-0.22492000	-2.18270400
H	5.89480700	1.07132400	-1.11400300
H	2.95286200	-1.46615000	-2.98363500
H	5.19850800	-0.35794300	-3.02446200
N	0.39674700	-1.13401000	-0.13529700
N	1.23302300	-1.26114900	-1.11954900
C	-0.86352400	-1.85830600	-0.31345400
C	-2.04639900	-1.15926600	-0.61443400
C	-0.81794800	-3.24426100	-0.16015100
C	-3.21399000	-1.93517900	-0.72715500
C	-1.99381500	-3.98179500	-0.28705400
H	0.12494600	-3.73254500	0.06443300
C	-3.19316100	-3.32050700	-0.56389900
H	-4.14443300	-1.43516000	-0.97664900
H	-1.97100200	-5.05994800	-0.16966600
H	-4.11377600	-3.88597500	-0.66707300
C	-2.10114000	0.31510700	-0.81819000
C	-1.28761700	0.95064100	-1.77316600
C	-3.01612200	1.09355500	-0.08717000
C	-1.37043700	2.32719500	-1.98540500
H	-0.61286300	0.36194100	-2.38697500
C	-3.10818900	2.47151100	-0.28412900
H	-3.65913000	0.62084400	0.64950400
C	-2.27708500	3.06249200	-1.22938800
H	-0.76357600	2.82551900	-2.73352800
H	-3.80775900	3.08087600	0.27744100
F	-0.86376200	0.35763800	4.21536300
F	-2.35735200	4.39634100	-1.42641800

Atomic coordinates of optimized **3h cation** (N_{imag} = 0)

1 1

C	-2.27356400	1.97892600	-1.19349600

C	-1.93420400	0.72324700	-0.64624400
C	-0.67653500	0.14932500	-0.99822900
C	0.19684400	0.80522500	-1.89867900
C	-0.15941400	2.02641900	-2.42466800
C	-1.39218600	2.59628500	-2.05187500
H	-3.21175500	2.46681900	-0.96239900
H	1.13629800	0.34610800	-2.17591400
C	-2.79474500	-0.02501400	0.22963800
C	-4.06713200	0.39163000	0.67219600
C	-2.31635800	-1.29964100	0.66744800
C	-4.81923300	-0.42543500	1.50003500
H	-4.46791800	1.35119300	0.36771100
C	-3.10219400	-2.12688500	1.51618000
C	-4.34136300	-1.68977800	1.92420000
H	-5.79728500	-0.08968200	1.83025500
H	-2.69629800	-3.08566000	1.82005700
H	-4.95479900	-2.30753400	2.57123500
N	-0.33212400	-1.08367300	-0.44846400
N	-1.09790200	-1.78611800	0.32587200
C	0.95200200	-1.71761500	-0.75819500
C	2.13553800	-1.25332500	-0.15800100
C	0.92653500	-2.77786100	-1.66421000
C	3.32368600	-1.90024800	-0.54089800
C	2.12268500	-3.40210200	-2.01463400
H	-0.01718100	-3.10060900	-2.09207300
C	3.32185400	-2.95496200	-1.45418200
H	4.25605300	-1.58073400	-0.08638900
H	2.11520700	-4.22788200	-2.71804500
H	4.25795600	-3.43626800	-1.71862200
C	2.17261900	-0.14706100	0.84035300
C	1.41487500	-0.20348700	2.02292100
C	3.01900400	0.95218000	0.62143100
C	1.49903300	0.82448400	2.96583200
H	0.79636400	-1.07139800	2.22739300
C	3.07233900	1.96180500	1.57489600
H	3.62615600	1.03409200	-0.27405400
C	2.32827000	1.92698700	2.74857700
H	0.92652500	0.76027800	3.88585800
H	2.41169200	2.73504800	3.46683100
H	0.48450600	2.55531000	-3.11839200
F	-1.71588900	3.78680200	-2.57133400

F	3.88194600	3.01996200	1.34381500

Atomic coordinates of optimized **3i cation** (N_{imag} = 0)

1 1

C	-1.80866400	-1.61029400	2.55525300
C	-1.54471900	-0.68963400	1.51236300
C	-0.19592300	-0.26927400	1.31941800
C	0.83992500	-0.73435100	2.16165100
C	0.53817900	-1.62485600	3.16994900
C	-0.78952200	-2.07057200	3.36328500
H	-2.82236200	-1.95217900	2.72541800
H	1.85431500	-0.38885900	2.01338900
H	-1.00877000	-2.77418100	4.15970100
C	-2.55159300	-0.13633500	0.65382000
C	-3.93034900	-0.43600500	0.70277200
C	-2.12066500	0.81171600	-0.32529800
C	-4.80941400	0.18064500	-0.16672800
H	-4.31214600	-1.15011800	1.42279000
C	-3.04469700	1.43464700	-1.20875800
C	-4.38916100	1.13286400	-1.14209800
H	-5.86562800	-0.06821700	-0.10617400
H	-2.65458700	2.14621900	-1.92927000
N	0.07595400	0.62874500	0.28847000
N	-0.82090600	1.15791900	-0.48509500
C	1.44161800	1.09162200	0.02949200
C	2.36319500	0.25448300	-0.61789400
C	1.75611500	2.37893200	0.46799200
C	3.65647400	0.78527700	-0.78024400
C	3.04381700	2.89226800	0.28718500
H	0.99230200	2.97541000	0.95817900
C	3.98879100	2.06320200	-0.33923200
H	4.40159900	0.18261300	-1.28997600
H	4.99941900	2.43037900	-0.49601300
C	2.03854200	-1.10901900	-1.12149900
C	0.96698400	-1.32574700	-2.00540400
C	2.84166900	-2.20249500	-0.75396100
C	0.70085300	-2.60502500	-2.49819100
H	0.36690500	-0.48539300	-2.34147100
C	2.57206400	-3.48113600	-1.24531600
H	3.67706700	-2.05143200	-0.07586800
C	1.49893800	-3.68673700	-2.11643500

H	-0.11876900	-2.75305800	-3.19505700
H	3.20195200	-4.31472700	-0.94998700
H	1.32776700	-1.98408700	3.82140500
H	1.29370700	-4.67975000	-2.50410500
C	-5.40182300	1.76983700	-2.05692100
H	-5.92939400	1.00980500	-2.64380500
H	-6.15739600	2.31563600	-1.48095900
H	-4.93161100	2.47000900	-2.75055100
C	3.40419400	4.28697300	0.73672500
H	4.31031200	4.28187800	1.35114400
H	3.60030200	4.93511200	-0.12498800
H	2.60071000	4.74237900	1.32101200

Atomic coordinates of optimized **3j cation** (N_{imag} = 0)

1 1

C	1.87231900	1.56826600	2.52261200
C	1.58369300	0.65367600	1.48101800
C	0.22819900	0.23807800	1.31651600
C	-0.78820100	0.70140700	2.18332400
C	-0.46160200	1.58504900	3.18929700
C	0.87195200	2.02590800	3.35512700
H	2.88957700	1.90789700	2.67430000
H	-1.80684200	0.35998000	2.05627800
H	1.11072600	2.72414400	4.15060800
C	2.56945400	0.10211800	0.59759600
C	3.95080300	0.40508500	0.62602400
C	2.11213200	-0.84138000	-0.37699000
C	4.82096800	-0.20056800	-0.25869700
H	4.34363200	1.11498500	1.34346200
C	3.00774700	-1.46592900	-1.28587900
C	4.33419300	-1.13214900	-1.20317600
H	5.88260000	0.02327400	-0.24544100
H	2.63806300	-2.17816300	-2.01413800
N	-0.06708400	-0.65406200	0.28890700
N	0.80745000	-1.18349100	-0.50829700
C	-1.43948200	-1.11132000	0.05486800
C	-2.36965500	-0.27201400	-0.58125500
C	-1.74437900	-2.39778000	0.49932100
C	-3.66956100	-0.79180400	-0.73246900
C	-3.04270300	-2.85316500	0.31324000
H	-1.00469000	-3.02704100	0.98109800

C	-4.01746400	-2.06721000	-0.29188700
H	-4.41403000	-0.18260400	-1.23432000
H	-5.02018900	-2.45845700	-0.42446200
C	-2.04178600	1.09002300	-1.08866800
C	-0.99636800	1.29372400	-2.00617700
C	-2.81794300	2.19217200	-0.69102300
C	-0.72863600	2.57107800	-2.50319000
H	-0.42099000	0.44586100	-2.36609400
C	-2.54620600	3.46867400	-1.18691100
H	-3.63399900	2.05022600	0.01224300
C	-1.49893900	3.66204500	-2.09167700
H	0.06864700	2.71028900	-3.22713500
H	-3.15495500	4.30976400	-0.86964300
H	-1.23500000	1.94257900	3.86071600
H	-1.29313400	4.65330700	-2.48332700
F	5.21713600	-1.69451000	-2.03939200
F	-3.35748900	-4.09183000	0.73671700

Atomic coordinates of optimized **3k cation** (N_{imag} = 0)

1 1

C	1.64189400	1.12352600	2.78291100
C	1.55021400	0.37057300	1.58618900
C	0.28164600	-0.19106800	1.26137700
C	-0.83201400	-0.02691300	2.11544300
C	-0.71557100	0.70664000	3.28237200
C	0.54493000	1.28714000	3.59792400
H	2.58740000	1.57111800	3.06485800
H	-1.77801600	-0.48133500	1.85094600
H	0.64240600	1.86696600	4.51137800
C	2.64766400	0.12080300	0.70213600
C	3.96778800	0.59565800	0.87215100
C	2.38515800	-0.69310700	-0.44392300
C	4.94920500	0.26801800	-0.04195300
H	4.22150200	1.21888200	1.72155900
C	3.41453300	-1.01794400	-1.37115900
C	4.69756600	-0.54897300	-1.18512200
H	5.95632300	0.64587200	0.11358300
H	3.15175200	-1.64038900	-2.22039900
N	0.17058400	-0.92582600	0.08284700
N	1.15556400	-1.18351000	-0.72409300
C	-1.10398400	-1.52588300	-0.31770500

C	-2.12192400	-0.73068400	-0.86653800
C	-1.23554900	-2.90032100	-0.11198500
C	-3.31745100	-1.40588900	-1.17778500
C	-2.42718100	-3.55389900	-0.43853700
H	-0.40421200	-3.45608700	0.31176000
C	-3.46826800	-2.77366200	-0.96785400
H	-4.12993400	-0.83838000	-1.62058200
H	-4.40871500	-3.24889600	-1.23359400
C	-1.99226100	0.72901800	-1.12497400
C	-0.93394700	1.25329300	-1.88538600
C	-2.96989800	1.62031600	-0.65029100
C	-0.85551500	2.62124400	-2.15111800
H	-0.18831000	0.58562500	-2.30679200
C	-2.88284800	2.98604800	-0.91687100
H	-3.80038400	1.24283500	-0.05976800
C	-1.82573200	3.51426800	-1.67392200
H	-0.03477000	2.99806800	-2.75606800
H	-3.65109100	3.65219900	-0.53276200
C	5.81695100	-0.86981300	-2.14038800
H	6.24399300	0.04652400	-2.56284200
H	6.62817700	-1.39742200	-1.62644500
H	5.47277300	-1.49687300	-2.96555900
C	-2.58734100	-5.04161200	-0.24206700
H	-3.50017800	-5.27026800	0.31752500
H	-2.66101400	-5.55631800	-1.20685100
H	-1.74074700	-5.46973500	0.30060900
C	-1.75998500	4.98774700	-1.99583200
H	-2.38587800	5.22393100	-2.86512600
H	-2.12093700	5.59666200	-1.16144400
H	-0.73951700	5.30204900	-2.23205300
C	-1.88375600	0.89720300	4.21298100
H	-1.65039700	0.51621000	5.21341300
H	-2.12261800	1.96123000	4.32122300
H	-2.77659100	0.38251300	3.85171300

Atomic coordinates of optimized **3l cation** (N_{imag} = 0)

1 1

C	-1.67631800	-1.23348200	2.70937200
C	-1.55113800	-0.42086900	1.55306400
C	-0.26761800	0.12830700	1.26077800
C	0.83890000	-0.09971200	2.10567300

C	0.64537300	-0.89249600	3.21125100
C	-0.59744500	-1.47419000	3.53050300
H	-2.63562500	-1.67003600	2.95833300
H	1.81063700	0.32930100	1.90350800
H	-0.68209200	-2.09198800	4.41775800
C	-2.63714400	-0.10095100	0.67645100
C	-3.96852400	-0.55145100	0.81411600
C	-2.34715900	0.76280300	-0.42764900
C	-4.93511600	-0.15449900	-0.08909400
H	-4.24533800	-1.21028400	1.62865600
C	-3.36196100	1.15856200	-1.34376400
C	-4.65717200	0.71226900	-1.18886900
H	-5.95173600	-0.51585200	0.04160300
H	-3.07810600	1.81588100	-2.15922400
N	-0.12950100	0.91543700	0.11890800
N	-1.10490500	1.23382000	-0.67800200
C	1.16457700	1.49712300	-0.24408700
C	2.16670600	0.69460000	-0.81147100
C	1.33324500	2.85770300	0.01865700
C	3.38591400	1.34370500	-1.08055100
C	2.54896800	3.48669900	-0.26608200
H	0.51354900	3.42139400	0.45432600
C	3.57374000	2.69696100	-0.81309800
H	4.18768900	0.77029400	-1.53520300
H	4.53172500	3.15362900	-1.04626600
C	1.99176900	-0.74932900	-1.12992200
C	0.94132300	-1.19422500	-1.95238300
C	2.91559700	-1.69425400	-0.64889100
C	0.80342300	-2.54467200	-2.27555600
H	0.24718400	-0.47634700	-2.37829200
C	2.79043900	-3.04846200	-0.96002400
H	3.73827800	-1.37101800	-0.01776300
C	1.73014600	-3.44757400	-1.76598900
H	0.00916100	-2.89651000	-2.92492700
H	3.49592600	-3.78453700	-0.59044100
C	-5.76227500	1.10627400	-2.13274200
H	-6.20662600	0.22202100	-2.60292400
H	-6.56480700	1.62511800	-1.59679600
H	-5.39884200	1.76620500	-2.92315100
C	2.75263400	4.95871500	-0.00447800
H	3.65159800	5.13146000	0.59610500

H	2.88024000	5.50703200	-0.94462600
H	1.90221100	5.39566000	0.52475800
F	1.68034400	-1.12599700	4.03105200
F	1.59860200	-4.75750800	-2.07139300

Atomic coordinates of optimized **3m cation** (N_{imag} = 0)

1 1

C	2.75425800	0.03868700	0.03629400
C	4.10074900	0.42493800	-0.16552300
C	2.22931600	-1.07401200	-0.71439400
C	4.88225000	-0.26414100	-1.06948200
H	4.51294200	1.25998400	0.39174600
C	3.06373100	-1.76661200	-1.64033300
C	4.36544400	-1.36382300	-1.80988100
H	5.91433900	0.03625300	-1.22036200
H	2.63929400	-2.60002200	-2.18941500
H	5.00928800	-1.88315000	-2.51167600
N	0.15013200	-0.91390600	0.23410400
N	0.95683700	-1.51344900	-0.60301700
C	-1.19252800	-1.47849200	0.32829300
C	-2.29990800	-0.76722800	-0.17442300
C	-1.30912700	-2.72277200	0.94795700
C	-3.56177400	-1.36133100	0.02418800
C	-2.57280800	-3.28503700	1.11715300
H	-0.41924000	-3.23293600	1.30202400
C	-3.69869400	-2.59288500	0.66192400
H	-4.44003500	-0.85546400	-0.36332000
H	-2.67505900	-4.25032300	1.60122800
H	-4.68762000	-3.02179300	0.78803600
C	-2.17073600	0.51155500	-0.89169300
C	-1.30068700	0.85194600	-1.90706600
S	-3.25939000	1.84160700	-0.55137700
C	-1.50438000	2.17047500	-2.40800600
H	-0.57495300	0.16051000	-2.32024800
C	-2.52497000	2.82617400	-1.76987200
H	-0.93006400	2.60630200	-3.21708300
H	-2.89259300	3.82808700	-1.94595400
C	1.84061000	0.63310600	0.92952100
C	0.52390400	0.13874200	1.03351000
S	2.08519800	1.94990900	2.03470100
C	-0.27168300	0.81443100	2.00972000

C	0.44381700	1.80347300	2.61337900
H	-1.30102700	0.57789500	2.23949600
H	0.10229700	2.47871500	3.38715300

Atomic coordinates of optimized **3n cation** (N_{imag} = 0)

1 1

C	-1.97532300	0.20090400	-0.38998700
C	-0.74344900	-0.30940000	-0.89305500
C	0.05356400	0.43177800	-1.81282400
C	-0.33704300	1.68668000	-2.17291300
H	0.94434600	-0.01550100	-2.23120400
C	-2.84718200	-0.74511200	0.24490700
C	-4.22865700	-0.56566000	0.52111200
C	-2.28952100	-2.03149900	0.56481200
C	-4.95850300	-1.55259200	1.15431300
H	-4.73697600	0.32755500	0.18825100
C	-3.04844400	-3.01991000	1.25628900
C	-4.36408300	-2.77529300	1.55952700
H	-6.01740700	-1.39288200	1.33271200
H	-2.56140100	-3.95915900	1.49470000
H	-4.95982700	-3.52289100	2.07220400
N	-0.33575600	-1.57635500	-0.50712600
N	-1.03915100	-2.39661900	0.22018200
C	0.97005500	-2.11295000	-0.89745200
C	2.13821600	-1.66823600	-0.25459500
C	0.97600700	-3.09494200	-1.88909900
C	3.34302100	-2.25965500	-0.67511500
C	2.18866800	-3.65639600	-2.28357100
H	0.04172100	-3.41009600	-2.34254300
C	3.37268400	-3.23460800	-1.67209900
H	4.26419600	-1.95674300	-0.18771200
H	2.20602600	-4.41955000	-3.05443800
H	4.32117900	-3.67439400	-1.96387100
H	0.24379400	2.24243300	-2.90263200
C	2.12443000	-0.67228700	0.85906400
C	2.80333300	0.58921200	0.74198200
C	1.48523700	-1.00012200	2.04500000
C	3.47925800	1.01008700	-0.43902800
C	2.79480500	1.47987800	1.86980900
C	1.48286700	-0.11787500	3.15032800
H	1.00435400	-1.96944600	2.13993500

C	4.11906000	2.23028800	-0.49896000
H	3.50107400	0.35652600	-1.30443200
C	3.46374700	2.73166600	1.77271400
C	2.12455600	1.09658900	3.06288800
H	0.98745600	-0.41343300	4.06997300
C	4.11466500	3.10174400	0.61718600
H	4.63555000	2.52416200	-1.40795700
H	3.45553200	3.39300500	2.63480000
H	2.13522400	1.77640600	3.91038300
H	4.62704800	4.05699800	0.55925300
C	-1.46224600	2.32493800	-1.56280800
C	-2.26011200	1.60669300	-0.61476900
C	-1.75381300	3.67796800	-1.84829200
C	-2.76302100	4.34156700	-1.17536400
H	-2.97354100	5.38343000	-1.39399300
H	-1.15172500	4.19783800	-2.58751200
C	-3.24127700	2.33606000	0.10432700
C	-3.48527200	3.67120700	-0.16989900
H	-3.77049000	1.87409600	0.92612200
H	-4.22914600	4.20813700	0.40999400

Atomic coordinates of optimized **3o cation** (N_{imag} = 0)

1 1

C	1.85582700	2.47084600	-0.29259400
C	2.01187700	1.08581900	-0.29554900
C	1.24362100	0.31832700	0.64953200
C	0.39305000	0.93779500	1.56399700
C	0.24959400	2.33245100	1.56371500
C	0.99199500	3.11935400	0.60599600
H	2.41860200	3.07865900	-0.99223400
H	-0.16083800	0.34483200	2.28071200
C	2.91354700	0.36979600	-1.16574800
C	3.74140100	0.95635500	-2.14270500
C	2.97807100	-1.04753900	-1.00599500
C	4.58914500	0.16983300	-2.90877900
H	3.72503500	2.02784700	-2.30321600
C	3.85191100	-1.84101300	-1.79677300
C	4.65154100	-1.23289100	-2.73883800
H	5.21970300	0.64307100	-3.65515600
H	3.86141300	-2.91336100	-1.63388900
H	5.32521700	-1.82241500	-3.35118700

N	1.38528000	-1.07537400	0.63884400
N	2.20483200	-1.72715800	-0.11790100
C	0.61919700	-1.91829500	1.55928700
C	-0.74608200	-2.16677100	1.32797700
C	1.31151100	-2.44083700	2.65174500
C	-1.40097400	-2.96431900	2.28417600
C	0.63094300	-3.23138700	3.57621400
H	2.36707800	-2.22109400	2.77579100
C	-0.73040600	-3.48541100	3.39089800
H	-2.44929600	-3.19777700	2.12836500
H	1.16009700	-3.64373900	4.42874600
H	-1.26923800	-4.10414500	4.10153300
C	-1.49078300	-1.64426100	0.14953500
C	-1.02457400	-1.88636500	-1.17682100
C	-2.67744600	-0.95176400	0.32768600
C	-1.73447200	-1.43089900	-2.26501200
H	-0.12570200	-2.47580300	-1.32835100
C	-3.42801400	-0.46373900	-0.77486600
H	-3.04972800	-0.76303300	1.33182300
C	-2.94562700	-0.70397100	-2.10333000
H	-1.38094500	-1.64296800	-3.27062400
C	-4.64457600	0.25289600	-0.60232100
C	-5.35132500	0.70832800	-1.69413500
H	-6.28129000	1.25025600	-1.55221300
H	-5.01301800	0.43160200	0.40437500
C	-4.87448100	0.46903000	-3.00813700
H	-5.44417900	0.83027900	-3.85874600
C	-3.69865100	-0.22193900	-3.20801500
H	-3.33579300	-0.41051800	-4.21474800
C	-0.61245800	2.99749400	2.48832200
C	-0.73612800	4.36359900	2.46192800
H	-1.39294900	4.86410600	3.16612000
C	-0.00658200	5.14013600	1.51397600
H	-0.12064500	6.21948300	1.51167300
H	-1.16679300	2.40455400	3.20960100
C	0.83463400	4.53714300	0.61171000
H	1.39076200	5.13107300	-0.10742200

Atomic coordinates of optimized **3p cation** (N_{imag} = 0)

1 1

C	1.97200600	2.48901700	-0.22736000

C	1.84941900	1.11397500	0.08395300
C	0.63045200	0.69214300	0.69989600
C	-0.38966100	1.60053000	1.00924100
C	-0.23001700	2.94575500	0.68883600
C	0.96637800	3.38636300	0.05866900
H	2.87720900	2.85358200	-0.69783500
H	-1.30288200	1.28141100	1.49240000
H	1.10005400	4.43052800	-0.19547200
C	2.86673900	0.13756500	-0.15485400
C	4.12593300	0.39005800	-0.74644500
C	2.58098100	-1.20581700	0.25589500
C	5.03836000	-0.63577400	-0.90839500
H	4.38623400	1.38889600	-1.07585800
C	3.53584500	-2.24936000	0.08106400
C	4.74958200	-1.96260800	-0.49347700
H	6.00044000	-0.42192100	-1.36370700
H	3.27018400	-3.24803000	0.41046400
H	5.48933000	-2.74321300	-0.63441200
N	0.48611200	-0.66531200	1.00095600
N	1.40302900	-1.56563500	0.80906600
C	-0.73506700	-1.17295300	1.62697900
C	-1.90233400	-1.35483000	0.86400400
C	-0.67307900	-1.44795500	2.99319600
C	-3.03617900	-1.81058400	1.56030900
C	-1.81420200	-1.90596600	3.64961500
H	0.25655200	-1.29561700	3.53217200
C	-2.99820500	-2.07953100	2.92854100
H	-3.95130900	-1.98103900	1.00217600
H	-1.77648300	-2.12376400	4.71168500
H	-3.89174000	-2.43855100	3.42927400
C	-1.97684100	-1.09759900	-0.60101800
C	-1.09494500	-1.72368800	-1.49951900
C	-2.97921400	-0.25725100	-1.11698500
C	-1.19726800	-1.50900500	-2.87453200
H	-0.34618000	-2.41630200	-1.12820400
C	-3.09302500	-0.02931600	-2.48850900
H	-3.67364200	0.23169900	-0.44009900
C	-2.19356300	-0.65893100	-3.34153300
H	-0.53557000	-2.00205200	-3.57835200
H	-3.86106800	0.61919400	-2.89553900
F	-2.29476100	-0.44276800	-4.67212400

O	-1.25243400	3.75381000	1.01172600
C	-1.17669700	5.16194100	0.73394700
H	-1.08473800	5.34303500	-0.34166400
H	-2.11742400	5.57455800	1.09481500
H	-0.34301100	5.62157800	1.27419200

Atomic coordinates of optimized **3q cation** (N_{imag} = 0)

1 1

C	-1.71524300	0.06870800	-2.70477800
C	-1.84671900	0.61420300	-1.40442400
C	-1.52498200	-0.22128400	-0.29855400
C	-1.12281600	-1.56228400	-0.48733600
C	-1.01509200	-2.05258700	-1.76998900
C	-1.30375100	-1.23313900	-2.88736500
H	-1.94483600	0.67762900	-3.57035700
H	-0.90781400	-2.19489300	0.36254000
H	-1.20752300	-1.63739400	-3.88957600
C	-2.30483200	1.94898000	-1.13282600
C	-2.66664300	2.90240800	-2.10631200
C	-2.41246200	2.32336200	0.24500100
C	-3.11748900	4.15445800	-1.72077700
H	-2.59729800	2.66541700	-3.16118500
C	-2.87928700	3.61266300	0.62307200
C	-3.22878200	4.51483100	-0.35495000
H	-3.39268900	4.87634800	-2.48337700
H	-2.94412700	3.84793700	1.67985600
H	-3.58693900	5.50294100	-0.08683100
N	-1.63015600	0.30647700	0.98960400
N	-2.06069600	1.49834700	1.25990700
C	-1.28918500	-0.49516000	2.16781700
C	0.05715200	-0.74423900	2.48926900
C	-2.35261100	-0.98750400	2.92411800
C	0.29089800	-1.55325700	3.61455200
C	-2.08376400	-1.77898600	4.03988900
H	-3.37361500	-0.76016700	2.63482100
C	-0.75886700	-2.06516600	4.37811900
H	1.31751900	-1.75494400	3.90325000
H	-2.90255100	-2.16818600	4.63553600
H	-0.54030200	-2.67955400	5.24559200
C	1.20066900	-0.19092100	1.71021700
C	1.34089100	1.19347000	1.51092200

C	2.18518200	-1.05084900	1.19702100
C	2.42648000	1.70255800	0.80045900
H	0.62135800	1.87895300	1.94791300
C	3.27029600	-0.54546300	0.48059400
H	2.10054300	-2.12262000	1.35044000
C	3.38749300	0.83179000	0.27696900
H	2.53683100	2.77391300	0.67031200
H	4.02237100	-1.22002700	0.08648900
C	-0.58899800	-3.48610900	-2.01283700
C	4.52311200	1.38668400	-0.54907000
F	5.59207100	0.56282000	-0.56765800
F	4.13854900	1.56961900	-1.84240900
F	4.93844000	2.59013800	-0.09212400
F	0.51777100	-3.53098600	-2.78669200
F	-0.31484500	-4.13482400	-0.86195400
F	-1.55963800	-4.17256000	-2.65414100

Atomic coordinates of optimized **3r cation** (N_{imag} = 0)

1 1

C	-1.98292400	2.44264200	-0.05358500
C	-1.43112800	1.14007600	-0.01545400
C	-0.01076300	1.01702300	-0.05283400
C	0.81726000	2.15786000	-0.16269200
C	0.23689300	3.40891900	-0.20439900
C	-1.16719200	3.55292400	-0.14013200
H	-3.05794300	2.57133100	-0.02160200
H	1.89193900	2.04731900	-0.22032100
H	-1.60727700	4.54425400	-0.17023800
C	-2.21420500	-0.06408900	0.03342800
C	-3.62317300	-0.12125900	0.07385700
C	-1.49515300	-1.30169100	0.02878400
C	-4.27080900	-1.34508200	0.09992600
H	-4.21001600	0.78935500	0.08283700
C	-2.17661400	-2.55038900	0.05593000
C	-3.55126400	-2.56605000	0.08896900
H	-5.35570200	-1.36972500	0.12978900
H	-1.58503200	-3.45935300	0.05379500
H	-4.08893300	-3.50782600	0.11049300
N	0.54339200	-0.26454800	-0.00203100
N	-0.14289600	-1.36470200	0.02418200
C	1.99073600	-0.46748000	0.01459700

C	2.71089500	-0.13023200	1.16103600
C	2.59536300	-1.03801400	-1.10421700
C	4.08646500	-0.36815400	1.17661200
C	3.97314800	-1.26336300	-1.07486400
H	2.00312600	-1.30045900	-1.97461100
C	4.71598500	-0.92941900	0.06110700
H	4.66184700	-0.12216100	2.06304400
H	4.46190600	-1.70302600	-1.93802600
H	5.78568700	-1.11119500	0.07989000
H	0.86646700	4.28802800	-0.29218300
H	2.20934000	0.29288000	2.02565400

Atomic coordinates of optimized **3s cation** (N_{imag} = 0)

1 1

C	-1.77986300	2.39484800	0.00647600
C	-1.39101000	1.03322000	0.01697400
C	0.00573600	0.75197300	-0.01527100
C	0.96117900	1.79067600	-0.08869500
C	0.55498600	3.11425100	-0.10658500
C	-0.83786500	3.39777500	-0.04857700
H	-2.83143100	2.65361500	0.03581000
H	2.01476200	1.54746100	-0.13760700
H	-1.16404500	4.43384100	-0.05755100
C	-2.30736500	-0.06960200	0.03573100
C	-3.71521300	0.03677100	0.06400900
C	-1.73648300	-1.38288800	0.01290800
C	-4.49987700	-1.10294000	0.06379200
H	-4.19153500	1.00964700	0.08457400
C	-2.55997600	-2.54428100	0.01243200
C	-3.92648500	-2.40002100	0.03633300
H	-5.58059300	-1.00234500	0.08507100
H	-2.07827500	-3.51583700	-0.00334600
H	-4.57056300	-3.27275100	0.03711500
N	0.40691500	-0.58493300	0.00812800
N	-0.40198300	-1.60110900	0.01064300
C	1.82099300	-0.95490900	0.02071300
C	2.56028600	-0.77069700	1.18913700
C	2.37085400	-1.52408800	-1.12643000
C	3.89949400	-1.16544200	1.19894200
C	3.71328300	-1.90840600	-1.10234600
H	1.76350500	-1.66557300	-2.01436300

C	4.47491500	-1.72929100	0.05589800
H	4.48790100	-1.03948400	2.10189800
H	4.15918400	-2.35118900	-1.98688700
H	5.51628600	-2.03432300	0.07034700
H	2.09891500	-0.34290600	2.07359700
C	1.54905500	4.24164000	-0.18979200
H	1.45682500	4.90402000	0.67822000
H	2.57643300	3.87391200	-0.23103200
H	1.36824700	4.85267000	-1.08114600

Atomic coordinates of optimized **3t cation** (N_{imag} = 0)

1 1

C	-1.84578700	2.35639100	-0.00493500
C	-1.40602900	1.00842000	0.01448900
C	-0.00049400	0.76654600	-0.01881400
C	0.92606100	1.82736500	-0.10235300
C	0.42921800	3.10993100	-0.12316400
C	-0.94779700	3.39921900	-0.06803800
H	-2.90569200	2.57650800	0.02414600
H	1.99266800	1.65683100	-0.15528400
H	-1.27435700	4.43321800	-0.08610600
C	-2.28885700	-0.12229600	0.03938400
C	-3.69840000	-0.06039300	0.07282000
C	-1.67711000	-1.41804500	0.01733400
C	-4.44677000	-1.22496400	0.07643700
H	-4.20707100	0.89590600	0.09419800
C	-2.46364600	-2.60506200	0.02146400
C	-3.83387100	-2.50364800	0.04876600
H	-5.52999400	-1.15775500	0.10146400
H	-1.95133200	-3.56082900	0.00681200
H	-4.45059400	-3.39586500	0.05299800
N	0.44322300	-0.55774100	0.01114200
N	-0.33759100	-1.59523600	0.01608800
C	1.86807600	-0.88172700	0.02373500
C	2.61382300	-0.62191100	1.17417200
C	2.42354700	-1.48190700	-1.10522300
C	3.96554700	-0.97084300	1.18328400
C	3.77838700	-1.81903200	-1.08166100
H	1.81173600	-1.68121000	-1.97883500
C	4.54654600	-1.56388200	0.05791200

H	4.55971700	-0.78565200	2.07206400
H	4.22945200	-2.28313500	-1.95248700
H	5.59812800	-1.83146300	0.07159700
H	2.14932100	-0.17324800	2.04661700
F	1.28710400	4.13472900	-0.20460500

Atomic coordinates of optimized excited state **3p cation**

1 1

C	-3.11868300	0.96300900	1.20861900
C	-2.33554000	0.12828000	0.39612600
C	-1.13538900	0.67705700	-0.13524500
C	-0.76246700	1.99669400	0.14842900
C	-1.57222000	2.79670900	0.96098600
C	-2.76231300	2.27604600	1.49825300
H	-4.04241500	0.58063000	1.62715000
H	0.14185200	2.43565200	-0.25155500
H	-3.40524000	2.87674400	2.12810400
C	-2.68111000	-1.24607000	0.05435100
C	-3.83679600	-1.90745500	0.49979600
C	-1.77799100	-1.95562900	-0.80489300
C	-4.11185200	-3.21748200	0.12209800
H	-4.53704300	-1.39362900	1.14897100
C	-2.07805600	-3.29709000	-1.18449200
C	-3.22493000	-3.91163600	-0.72745500
H	-5.01354900	-3.70310900	0.47929400
H	-1.38569300	-3.80564000	-1.84673900
H	-3.44832000	-4.93103100	-1.02544300
N	-0.33335100	-0.14643100	-0.93085100
N	-0.63613400	-1.43246000	-1.30352600
C	0.76938300	0.38783300	-1.68688800
C	2.12598200	0.32143500	-1.23927100
C	0.47052900	1.02644000	-2.87717200
C	3.12078300	0.93676200	-2.05246700
C	1.47797000	1.58999900	-3.67899700
H	-0.56687000	1.08613700	-3.18648400
C	2.80948200	1.54126400	-3.26182700
H	4.16009400	0.87889500	-1.74961600
H	1.21411300	2.06488400	-4.61837000
H	3.59618200	1.96765600	-3.87466100
C	2.57442500	-0.34511900	-0.02270600
C	1.98346300	-1.56572500	0.44085600

C	3.67777700	0.20239100	0.71812200
C	2.46175700	-2.19876900	1.56486600
H	1.18130000	-2.01081900	-0.13154700
C	4.14551700	-0.40623500	1.85832300
H	4.11821200	1.14073500	0.40386000
C	3.53217900	-1.60861000	2.26568400
H	2.04938300	-3.13754900	1.91598200
H	4.95485500	0.00842300	2.44816700
F	3.98696500	-2.21083300	3.35132900
O	-1.11901400	4.05786400	1.17082500
C	-1.90313400	4.95507100	1.96297900
H	-2.01369700	4.58356000	2.98771100
H	-1.35028500	5.89380700	1.97385300
H	-2.88964400	5.11645000	1.51494300

X-ray Crystallographic Analysis

General Crystal Growing Conditions: X-ray quality single crystals of **3i** and **3m** were grown from the solvent of $CHCl_3$ at room temperature by slow evaporation.

ORTEP diagram of compound **3i** (CCDC 2299966)

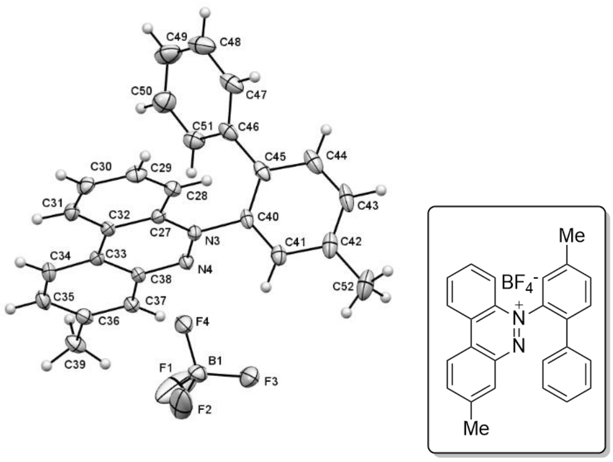

Table S4. Crystal data and structure refinement for compound **3i**

Empirical formula	$C_{53}H_{43}B_2Cl_3F_8N_4$	
Formula weight	1015.88	
Temperature	100(2) K	
Wavelength	0.71073 Å	
Crystal system	Monoclinic	
Space group	$P2_1/c$	
Unit cell dimensions	a = 19.5685(8) Å	α= 90°.
	b = 28.1537(12) Å	β= 97.555(2)°.
	c = 8.8507(4) Å	γ = 90°.
Volume	4833.7(4) $Å^3$	
Z	4	
Density (calculated)	1.396 Mg/m^3	
Absorption coefficient	0.263 mm^{-1}	
F(000)	2088	
Crystal size	0.20 x 0.15 x 0.06 mm^3	
Theta range for data collection	1.050 to 26.386°.	
Index ranges	-24<=h<=24, -34<=k<=35, -11<=l<=11	
Reflections collected	41689	
Independent reflections	9869 [R(int) = 0.0348]	
Completeness to theta = 25.242°	99.9 %	
Absorption correction	Semi-empirical from equivalents	
Max. and min. transmission	0.7454 and 0.6915	
Refinement method	Full-matrix least-squares on F^2	
Data / restraints / parameters	9869 / 180 / 681	
Goodness-of-fit on F^2	1.052	
Final R indices [I>2sigma(I)]	R1 = 0.0619, wR2 = 0.1364	
R indices (all data)	R1 = 0.0836, wR2 = 0.1512	
Extinction coefficient	n/a	
Largest diff. peak and hole	1.919 and -1.593 e.$Å^{-3}$	

ORTEP diagram of compound **3m** (CCDC 2299967)

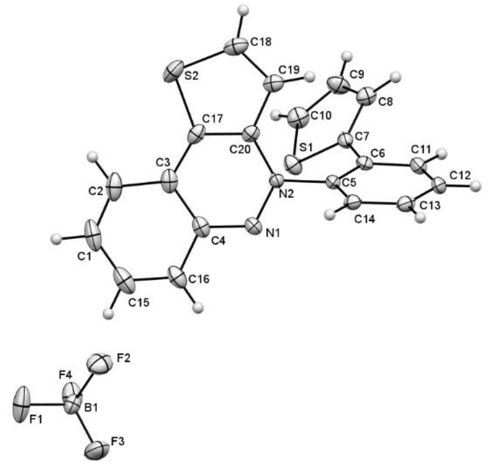

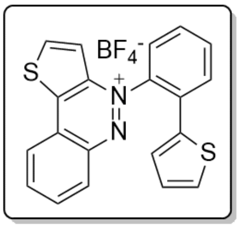

Table S5. Crystal data and structure refinement for compound **3m**

Empirical formula	$C_{20}H_{13}BF_4N_2S_2$	
Formula weight	432.25	
Temperature	100(2) K	
Wavelength	0.71073 Å	
Crystal system	Orthorhombic	
Space group	Pbca	
Unit cell dimensions	a = 12.5077(13) Å	α= 90°.
	b = 15.1838(15) Å	β= 90°.
	c = 20.313(2) Å	γ = 90°.
Volume	3857.7(7) $Å^3$	
Z	8	
Density (calculated)	1.489 Mg/m^3	
Absorption coefficient	0.322 mm^{-1}	
F(000)	1760	
Crystal size	0.12 x 0.09 x 0.02 mm^3	
Theta range for data collection	2.005 to 26.387°.	
Index ranges	-15<=h<=15, -18<=k<=18, -25<=l<=25	
Reflections collected	52342	
Independent reflections	3955 [R(int) = 0.0685]	
Completeness to theta = 25.242°	100.0 %	
Absorption correction	Semi-empirical from equivalents	
Max. and min. transmission	0.7454 and 0.6754	
Refinement method	Full-matrix least-squares on F^2	
Data / restraints / parameters	3955 / 0 / 262	
Goodness-of-fit on F^2	1.021	
Final R indices [I>2sigma(I)]	R1 = 0.0369, wR2 = 0.0838	
R indices (all data)	R1 = 0.0621, wR2 = 0.0953	
Extinction coefficient	n/a	
Largest diff. peak and hole	0.335 and -0.328 e.$Å^{-3}$	

Scheme S1. Control experiments.

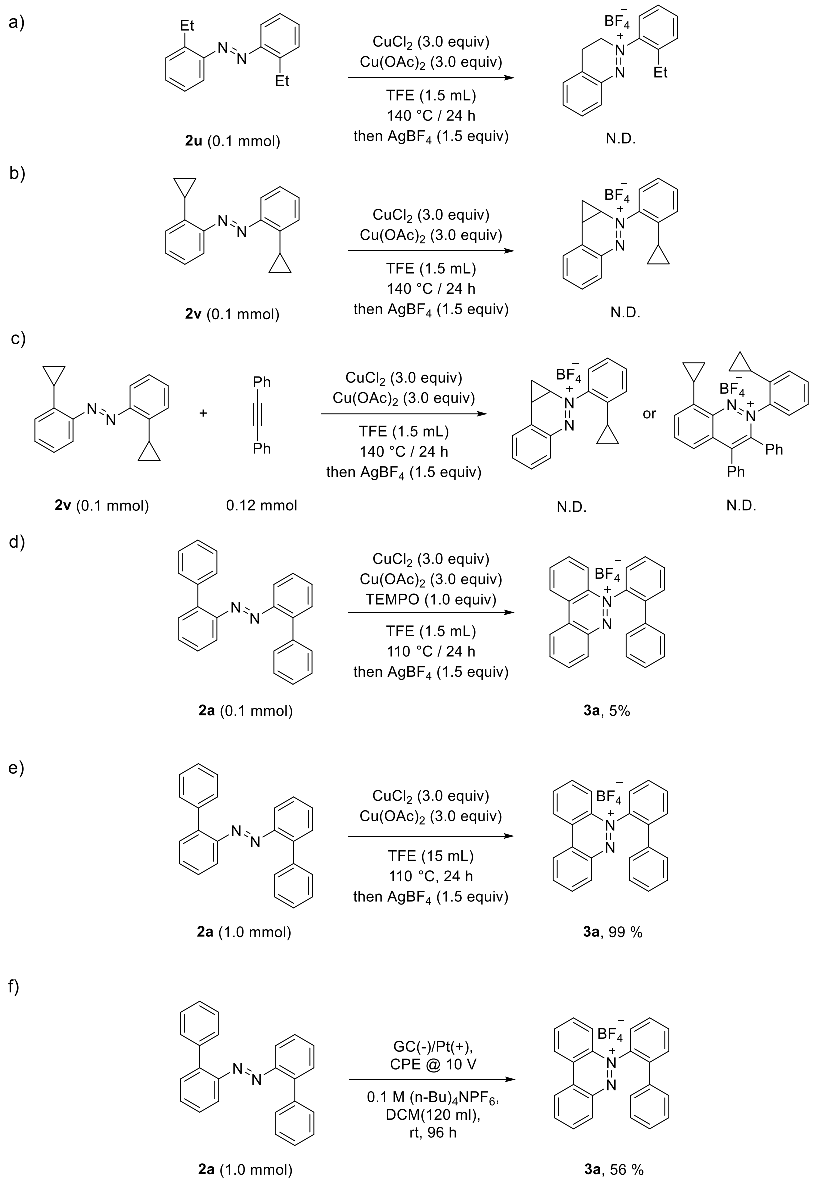

Reference

[1] V. Rajeshkumar, F.-W. Chan, S.-C. Chuang, *Adv. Synth. Catal.* **2012**, *354*, 2473-2483.

[2] J. Jayakumar, G. Vedarethinam, H.-C. Hsiao, S.-Y. Sun, S.-C. Chuang, *Angew. Chem. Int. Ed.* **2020**, *59*, 689-694.

[3] N. Khatun, A. Modi, W. Ali, B. K. Patel, *J. Org. Chem.* **2015**, *80*, 9662-9670.

[4] J. Hubrich, T. Himmler, L. Rodefeld, L. Ackermann, *ACS Catal.* **2015**, *5*, 4089-4093.

[5] M. Li, Y. Ye, *ChemCatChem* **2015**, *7*, 4137-4142.

[6] Gaussian 16, Revision B.01, M. J. Frisch, G. W. Trucks, H. B. Schlegel, G. E. Scuseria, M. A. Robb, J. R. Cheeseman, G. Scalmani, V. Barone, G. A. Petersson, H. Nakatsuji, X. Li, M. Caricato, A. V. Marenich, J. Bloino, B. G. Janesko, R. Gomperts, B. Mennucci, H. P. Hratchian, J. V. Ortiz, A. F. Izmaylov, J. L. Sonnenberg, D. Williams-Young, F. Ding, F. Lipparini, F. Egidi, J. Goings, B. Peng, A. Petrone, T. Henderson, D. Ranasinghe, V. G. Zakrzewski, J. Gao, N. Rega, G. Zheng, W. Liang, M. Hada, M. Ehara, K. Toyota, R. Fukuda, J. Hasegawa, M. Ishida, T. Nakajima, Y. Honda, O. Kitao, H. Nakai, T. Vreven, K.

Throssell, J. A. Montgomery, Jr., J. E. Peralta, F. Ogliaro, M. J. Bearpark, J. J. Heyd, E. N. Brothers, K. N. Kudin, V. N. Staroverov, T. A. Keith, R. Kobayashi, J. Normand, K. Raghavachari, A. P. Rendell, J. C. Burant, S. S. Iyengar, J. Tomasi, M. Cossi, J. M. Millam, M. Klene, C. Adamo, R. Cammi, J. W. Ochterski, R. L. Martin, K. Morokuma, O. Farkas, J. B. Foresman, and D. J. Fox, Gaussian, Inc., Wallingford CT, 2016.
